# A Stochastic Dynamic Operator Framework That Improves the Precision of Analysis and Prediction Relative to the Classical Spike-Triggered Average Method, Extending the Toolkit

**DOI:** 10.1523/ENEURO.0512-23.2024

**Published:** 2024-11-05

**Authors:** Trevor S. Smith, Maryam Abolfath-Beygi, Terence D. Sanger, Simon F. Giszter

**Affiliations:** ^1^Neurobiology and Anatomy, and Marion Murray Spinal Cord Research Center, Drexel University College of Medicine, Philadelphia, Pennsylvania 19129; ^2^Department of Electrical Engineering and Computer Science, University of California Irvine, Irvine, California 92697

**Keywords:** interneuron, SDO, spike-triggered average, spinal cord, STA, stochastic dynamic operator

## Abstract

Here we test the stochastic dynamic operator (SDO) as a new framework for describing physiological signal dynamics relative to spiking or stimulus events. The SDO is a natural extension of existing spike-triggered average (STA) or stimulus-triggered average techniques currently used in neural analysis. It extends the classic STA to cover state-dependent and probabilistic responses where STA may fail. In simulated data, SDO methods were more sensitive and specific than the STA for identifying state-dependent relationships. We have tested SDO analysis for interactions between electrophysiological recordings of spinal interneurons, single motor units, and aggregate muscle electromyograms (EMG) of major muscles in the spinal frog hindlimb. When predicting target signal behavior relative to spiking events, the SDO framework outperformed or matched classical spike-triggered averaging methods. SDO analysis permits more complicated spike–signal relationships to be captured, analyzed, and interpreted visually and intuitively. SDO methods can be applied at different scales of interest where spike-triggered averaging methods are currently employed, and beyond, from single neurons to gross motor behaviors. SDOs may be readily generated and analyzed using the provided *SDO Analysis Toolkit*. We anticipate this method will be broadly useful for describing dynamical signal behavior and uncovering state-dependent relationships of stochastic signals relative to discrete event times.

## Significance Statement

Here the authors introduce new tools and demonstrate data analysis using a new probabilistic and state-dependent technique, which is an expansion and extension of the classical spike-triggered average, the stochastic dynamic operator (SDO). SDO methods extend application into domains where classical spike-triggered averages fail, capture more information on spike correlations, and match or outperform the spike-triggered average when generating predictions of signal amplitude based on spiking events. A data and code package toolkit for utilizing and interpreting SDO methods is provided together with example simulated and physiological data analyses. Both the method and the associated toolkit are expected to be broadly useful in research domains where the spike-triggered average is currently used for analysis, and beyond.

## Introduction

Standard methods of examining the association of a neural spike and an external or physiological signal, despite variability in environment and organismal state, involve averaging perievent segments of the signal over multiple spiking events (i.e., “spike-triggered” averaging; Kirkwood and Sears, 1975). Under various assumptions (e.g., noise is additive and independent, the spike effect is stationary), such averaging reduces the magnitude of stochastic noise and isolates the correlation of a particular spike train and signal relative to other neurons or neural signals. This relationship between spike and signal may vary: A spike may be affected by a signal, a spike may affect a change of a signal, or a neuron may be both sensitive to and evoke a change in signal. Coarsely stated, analysis of individual neurons often seeks to characterize when a neuron is active and what happens when the neuron fires.

Here we consider the role of the STA in predicting changes in signal amplitude. STA methods have identified significant structural and functional relationships from cortical neurons (Fetz and Cheney, 1980; Buys et al., 1986), midbrain (Cheney, 1980), and spinal interneurons (Maier et al., 1998; Hart and Giszter, 2010; Takei et al., 2017) to muscle activations. The STA has also been instrumental in characterizing receptive fields of neurons within sensory systems. The related stimulus*-*triggered averaging method has similarly been valuable for interpreting stimulation-driven behavioral output (Cheney and Fetz, 1985) and is standard procedure for neural analysis. Importantly, classical triggered averaging necessarily presumes that signal behavior and evoked spike/stimulus effects are comparable across all spiking events (i.e., can be averaged together). In practice, such relationships are often dynamic, such as the current length of a muscle affecting the tension generated by a motoneuron spike (Gordon et al., 1966).

**Figure 1.**
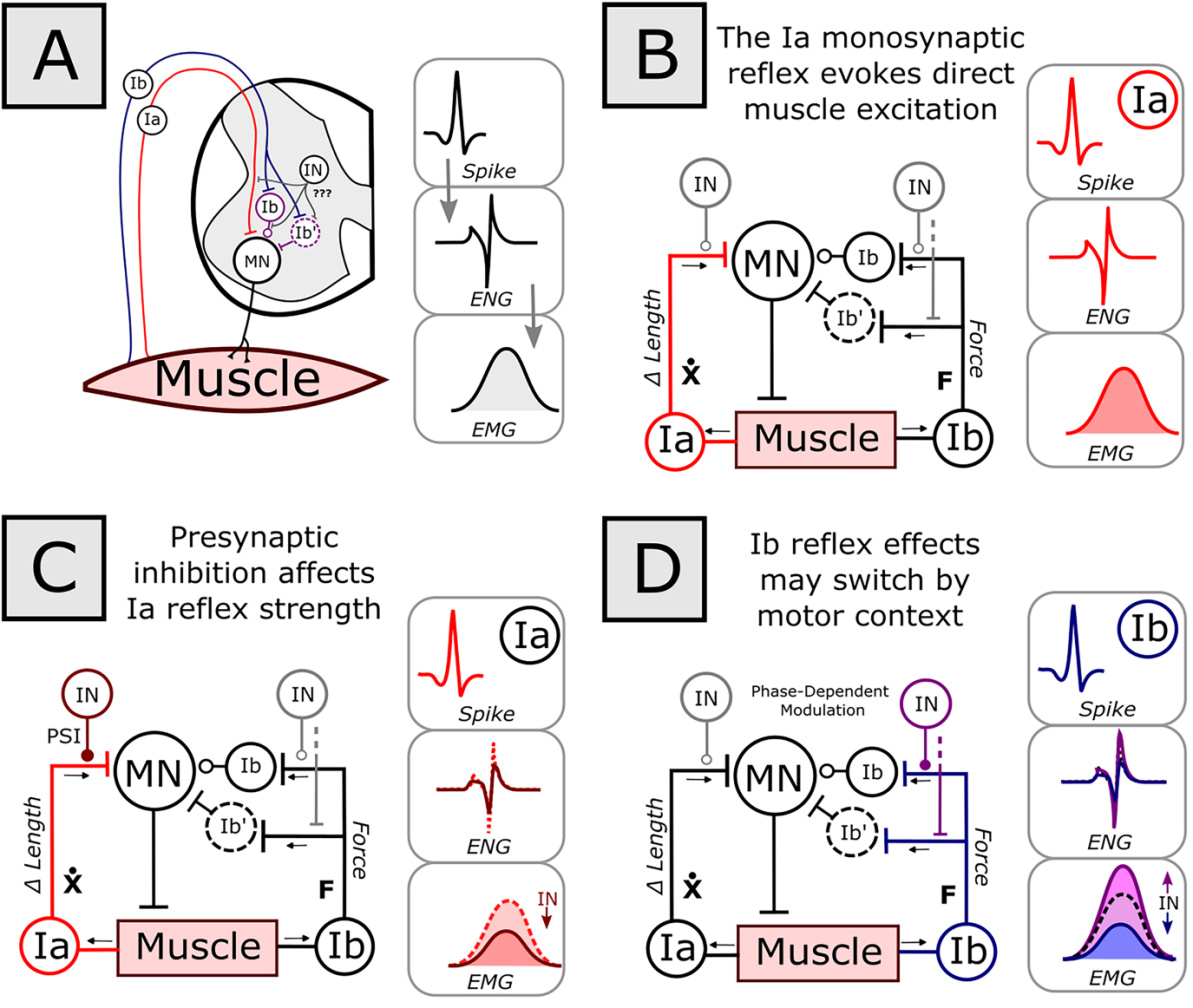
The nervous system generates state-dependent behavior, altering the effects of single spikes in the spinal reflex systems. ***A***, Due to the presence of monosynaptic and disynaptic reflexes provided by the Ia and Ib afferent systems, there is a parametric relationship between activity of these neurons and muscle behavior. One method of interrogating the effects of a neuron's spike on the spinal output would be to average the electroneurogram (ENG) or electromyogram (EMG) signal traces at time of a spike (i.e., “Spike-triggered Average”, STA). Here, the state of other spinal interneurons may influence this circuit but are often not directly known. ***B***, Within a simplified circuit model, through the monosynaptic reflex, the Ia afferent detects changes in muscle length and provides excitation to the homonymous motor pool. Under these conditions, the average effect of a spike in the Ia afferent will be to increase the ENG and EMG of this muscle. However, the Ia afferent is only one of many inputs into the system. ***C***, Presynaptic inhibition (PSI) of the Ia afferent may directly attenuate the amplitude of the spike effects of the Ia afferent. Determining an average spike effect by averaging EMG relative to the spikes of the Ia afferent, without additional consideration, will necessarily average over both conditions with and without presynaptic inhibition, which may obscure dynamical behavior. ***D***, The effects of the Ib afferents are known to be selectively modulated by spinal state/activity. While during muscle tension, the Ib afferent may attenuate ENG or EMG levels to the homonymous motor pool (blue), in other conditions, such as during initial muscle loading in the early stance phase of locomotion, the Ib afferent spike effects may enhance ENG or EMG (interneurons mediating this switching behavior are stylized in purple). Clearly, use of the simple spike-triggered average across these very different conditions would be ill-equipped to handle this behavior. MN, motoneuron; IN, interneuron; PSI, presynaptic inhibition.

Even under similar experimental recording conditions, neurons may demonstrate variable post-spike effects due to underlying neural system state or dynamics. To illustrate, consider the simplified spinal reflex pathway with monosynaptic spindle and disynaptic Golgi tendon organ (GTO) effects (Fig. 1*A*). Here, some of the mechanical parameters of the muscle are encoded by muscle spindles and GTOs and relayed to the spinal cord via the Ia and Ib afferent, respectively. The Ia afferent then monosynaptically excites motoneurons within the homonymous motor pool, evoking muscle contraction in a textbook fashion (Fig. 1*B*). Spikes recorded at the Ia afferent can predict motor outflow, measured by electroneurogram (ENG) or electromyogram (EMG). [Indeed, direct stimulation of this pathway induces the Hoffmann reflex (Hoffmann, 1910), comprising a clinical measure of spinal excitability.] However, the precise parametric relationship between Ia afferent and muscle response also depends on other factors, e.g., muscle length (Shiavi and Negin, 1975) and presynaptic inhibition of the Ia afferent in the spinal cord (Devanandan et al., 1964; Clark and Cope, 1998; Fig. 1*B*). Presynaptic inhibition (modeled by IN in Fig. 1*C*) or other inhibitory drives may cause Ia afferent spikes to reduce excitatory postsynaptic potentials on motoneurons, thereby lessening observed ENG or EMG responses. The effective strengths of Ia reflexes are regulated and can differ among spinal conditions, such as posture and locomotion, and between locomotor phases. The correlation between a spike of the Ia afferent (itself only one of many inputs to the motoneurons) and motor behavior may thus depend upon the state of these ongoing background spinal dynamics. Indeed, the Ib pathway's state-driven disynaptic modulation is still more influential and may switch effects between inhibition to excitation based on locomotor phase (Fig. 1*D*; Prochazka et al., 1997). Aspects of these background dynamics are also reflected in signal “state” (here, the level of ENG or EMG activity). Spike-triggered averaging the effect of the Ia or Ib afferent, without considering signal state, also averages over these intrinsic dynamics and potential state-dependent modulations.

Our objective here is to present a flexible and scalable data modeling and analysis framework which can better capture dynamical signal behaviors in the central nervous system relative to spiking events, where state dependence exists, and where responses may be stochastic. Previously, the “Stochastic Dynamic Operator” (SDO) was developed and demonstrated as a theoretical model accounting for stochastic state-dependent effects of individual and population neural firing on signal dynamics (Sanger, 2010, 2011, 2014). Here, for the first time, we demonstrate the utility of SDOs for analysis and interpretation of real neural datasets and validate the SDO methods’ performance with simulated data examples. We briefly explore examples of SDO analysis for interpreting and stochastically predicting biological signals using the correlated activity between interneurons, single motor units, and muscles in spinal motor behaviors.

## Materials and Methods

### Humane treatment and institutional animal care and use committee approvals

All experimental procedures complied with the guidelines of the National Institutes of Health Guide for Care and Use of Laboratory Animals and received approval from the Institutional Animal Care and Use Committee of Drexel University.

### Biological rationale and choice of test data framework

We selected the well-worked and robust spinal frog model to test the SDO framework with biological data. The spinal frog exhibits a well-characterized hindlimb-to-hindlimb wiping reflex (Giszter et al., 1989; Schotland and Rymer, 1993a,b; Kargo and Giszter, 2000) to rid the skin of a noxious stimulus. The role of spinal interneurons contributing to muscle activations within this model has previously been investigated using the STA (Hart and Giszter, 2010). Furthermore, because the frog lacks gamma motor neurons, proprioceptive feedback from the musculature is dependent upon muscle activations, lengths, and forces, which may be experimentally measured or estimated, and is not modified by independent control of muscle spindles. This anatomical arrangement is ideal for minimizing trial-to-trial variation in reflexive wiping, representing an ideal case for the use of the classical STA within the system, and to compare the STA in the best light with the new SDO approaches.

### Surgical methods

We anesthetized adult bullfrogs using 1 ml/kg of 5% tricaine and incubated them on ice to quicken the anesthetic effect. The skin on the skull was incised along the dorsal midline between the eyes and ears and then reflected away to reveal the musculature. We incised the midline musculature fascia behind the skull and deflected the musculature using a retractor to expose the foramen magnum. After opening the foramen, we cauterized the vascular dura over the fourth ventricle and cut an opening along the midline with iridectomy scissors. We then laterally transected the spinal cord with fine vacuum aspiration immediately below the medulla of the frog, taking care not to rupture any of the remaining dura or large blood vessels along the sides of the incision or underlying the spinal tissue. Following spinal transection, we filled the cavity with a piece of Gelfoam and closed the incision. We next made a small opening over the brain and decerebrated the frog at a low level through repeated applications of heat cautery to the tectum and rostral structures. After packing the cavity with Gelfoam, the incision was closed with wound clips and sealed with Vetbond (cyanoacrylate). We inserted bipolar intramuscular stainless steel electromyography (EMG) electrodes in 11 muscles of the frog right hindlimb: the rectus anterior (RA), rectus interior (RI), adductor magnus (AD), semimembranosus (SM), gluteus (GL), vastus internus (VI), biceps (BI), sartorius (SA), vastus externus (VE), gastrocnemius (G), and tibialis anterior (TA). For recording from the fragile sartorius (SA), a silicon patch electrode was placed under the muscle belly. To expose the spinal cord for extracellular recording, we made an incision on the mid-to-lower back of the frog's skin and musculature and reflected the skin back. We incised fascia, separated the back musculature with blunt dissection, and kept the muscles deflected via retractors. Other vertebral musculature was cleared using blunt dissection and iridectomy scissors until the bone of the vertebral arches was cleanly exposed. After clearing connective tissue, we used rongeurs to cut away the spinal arch and spinous processes, revealing the dura beneath. We removed three arches, exposing the L2/L3 border region of the frog spinal cord. We used an electrocautery to cauterize small patches of blood vessels in the vascular dura and expanded these holes until it was possible to deflect the dura and the attached blood vessel back, revealing the spinal cord pia mater for a length of three spinal segments. This allowed full access to half the width of the spinal cord, from the midline to the right lateral extreme of the cord. We covered the dura with moistened Gelfoam and a cotton ball. The frog was allowed to recover overnight. We opened a small hole in the pia mater using microincision on the day of recording to provide access directly to the white and gray matter.

### Reflex preparation

During experimental recordings, the frog was placed on a molded stand that supported the body in the horizontal plane. The pelvis and vertebral column were immobilized using custom-made clamps. The wiping limb was secured into an ankle restraint connected to a six-axis force/torque transducer (ATI 310) to record isometric forces generated at the ankle. Hindlimb-to-hindlimb wiping was evoked by electrically stimulating the dorsolateral surface of the heel of the target (left, contralateral) limb. The stimulation was delivered via bipolar leads (2–3 mm separation) and consisted of a 350 ms train of 3–8 V, 1 ms biphasic pulses applied at 40 Hz. This stimulus evoked either bilaterally coordinated hindlimb wiping attempts, between the contralateral limb (containing EMG electrodes) and ipsilateral limb, or ipsilateral flexion withdrawals from the stimulated limb. We adjusted the bipolar leads as necessary to evoke wiping motor responses from the right (contralateral) limb. Trials were composed of 60 s intervals, with stimuli applied 5–10 s after trial recording. Stimulus applications were spaced at least 2 min apart to avoid habituation.

### EMG recording

**Figure 2.**
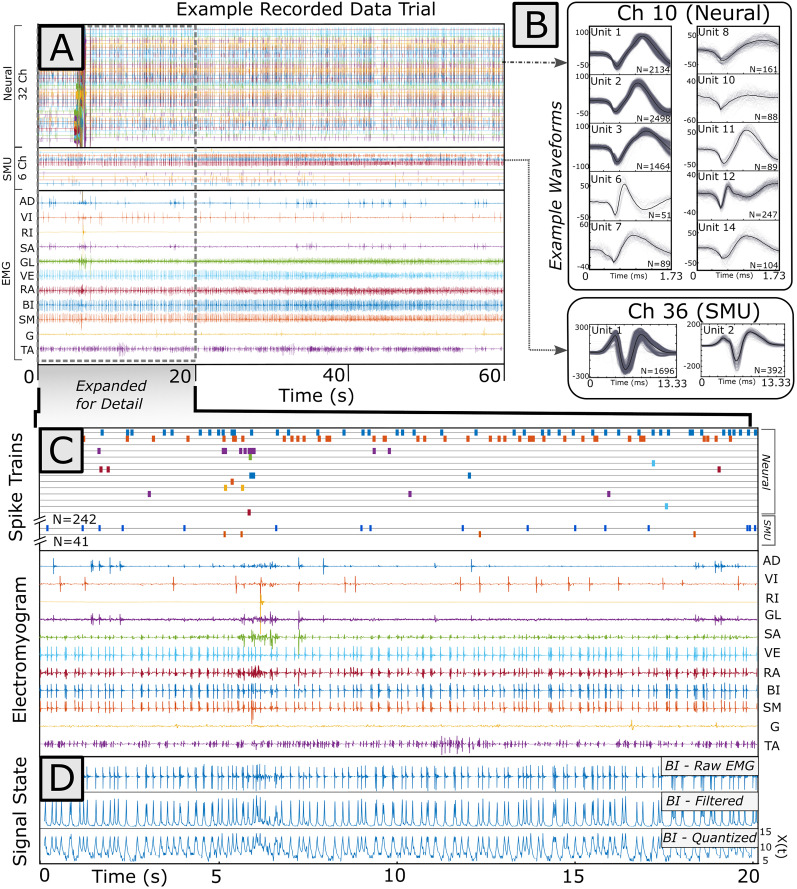
Example 60 s data trial. ***A***, Spiking data were recorded on 32 channels (neurons) and 6 data channels (single motor units, SMU). Analog EMG was recorded across 11 muscles. Both sensory-elicited and ongoing motor behavior were recorded in this trial. ***B***, Example waveforms from a single neural and motor unit channel within the greater dataset are plotted. Waveforms from both neural spikes and motor units, collected across all trials, were sorted in *Plexon Offline Sorter*. (The 13 ms single motor unit waveforms displayed here were extracted from stainless steel vastus externus EMG signal recorded 3.33 ms before to 10 ms after motor unit spike time.) Spiking events in sorted clusters were treated as point processes. ***C***, Expanded signal and sorted point processes from a single neural and motor unit channel. The total number of spikes and their correlated occurrence relative to EMG signal could be investigated through different analytical methods, including the spike-triggered average or the SDO. ***D***, Example raw EMG from the biceps femoris muscle, which was rectified and filtered and then quantized to derive the time series of signal “state” used for later analysis. Here higher signal “state” corresponds to a higher signal amplitude of the rectified and filtered EMG signal amplitude. A logarithmic scheme was used for quantization of state from signal amplitude.

EMG signals were recorded from the right, isometrically fixed, hindlimb using implanted wire pairs. Signals were sampled at 2 kHz, differentially amplified by a gain of 10,000 by a Ripple Grapevine EMG front-end amplifier, and bandpass filtered (15–375 Hz) online by a Ripple Grapevine neural interface processor (Fig. 2*A*). Spinally organized wiping movements were elicited through a cutaneous stimulation electrode applying a 250 ms train of 40 Hz biphasic 1 ms electrical pulses to the ankle dorsum.

### Neural recording

We recorded extracellular neural activity using a flexible braid (Kim et al., 2013, 2019a,b) of 24-channel microwires (each wire 9.6 μm diameter) forming a multielectrode, ∼240 μm total in diameter. Recording site impedances on the microwire were set between 0.2 and 0.5 MΩ by controlled platinum nanograss plating. We targeted the L1–L3 region of the spinal cord, <500 μm lateral of the spinal cord midline, a region of the spinal cord with a high yield of motor responses to microstimulation and high yield of wipe-active neurons (Bizzi et al., 1991; Giszter et al., 1993; Hart and Giszter, 2010). The neural multielectrode was advanced until action potentials were apparent (Fig. 2*A*). Continuous signals were filtered using 0.3 Hz high-pass, and 7.5 kHz low-pass filters, and digitized at 30 kHz using a Ripple Grapevine neural interface using *Nano2* Front Ends. Neural events were detected using a threshold of 4.5× the root mean square (RMS) of each channel's background activity. Each event was sampled as a 1.73 ms (52-point) waveform for offline clustering and analysis.

### Motor unit recording

Motor unit action potentials were recorded in muscle using a pair of 6-channel braided microwire (9.6 μm diameter) electrodes, with a seventh wire acting as reference, also in the braid assembly. Microwires were of sufficient length to reach a Ripple Grapevine Neural Front-End Amplifier without tension during frog movement (∼8–10 cm). Motor unit data was recorded as with neural data, above. We exposed a single recording site (∼50 μm) on each 9.6 μm Nickel–Chromium wire in the braid using laser microablation, with site impedance controlled to 0.2–0.5 MΩ via gold electroplating, like the neural probe. Single motor unit probes were inserted into the proximal vastus externus muscle, oblique to the fiber pennation angle, via a curved suture needle. Electrodes were also grounded through a 7-strand stainless steel wire (AM Microsystems) into the belly of the same muscle.

### Data processing methods

#### Data preprocessing

Off-line cluster cutting of both interneuron and motor unit spikes was performed using Plexon Neurotechnology Research System's Offline Sorter software. The first three principal components of 1.73-ms-long waveforms, centered on each recorded spike, were clustered using an automated, expectation–maximization sorting algorithm (Figueiredo and Jain, 2002; Shoham et al., 2003) followed by manual curation (Fig. 2*B*). Sorted spike trains and EMG signals were imported into MATLAB for further analysis (Fig. 2*C*). EMG signals were further processed off-line using a series of zero-phase filters: (1) a 60 Hz notch filter, (2) a 10 Hz high-pass filter (fourth order Butterworth), and (3) a 20-point root mean square (RMS) filter, which smoothed and rectified the EMG signal. We assigned EMG signal “state” based on signal amplitude. Since the smoothed and rectified EMG signal amplitude distribution fell into a roughly exponential distribution, we quantized EMG amplitude into signal states using logarithmic binning to allocate probabilities of state closer to a normal distribution: We defined 20 signal states for each EMG channel, representing equal intervals of the log-transformed EMG signal amplitude (Fig. 2*D*).

### Algorithms developed and tested

#### Theoretical consideration of when the spike-triggered average may fail

Biological systems are dynamic and incompletely observed. Consequently, signals measured within the system are often stochastic and could be spuriously correlated from an outside perspective. When determining a neurons’ contribution or sensitivity to a measured covariate signal, various assumptions are made regarding the structure of the relationship. The STA supports different causal models. It provides one method to reverse-correlate a neuron's spikes to preceding changes in signal amplitude. The STA also provides the means to predict future signal state based on a spike occurrence. The latter is the focus of the methods compared here. When spiking events (or at least the result of neural spikes) are considered independent and the stochastic component of the signal is uncorrelated with a spike, averaging signal amplitude around spiking events provides a straightforward method of isolating a single “response” of the signal corresponding to spiking events.

**Figure 3.**
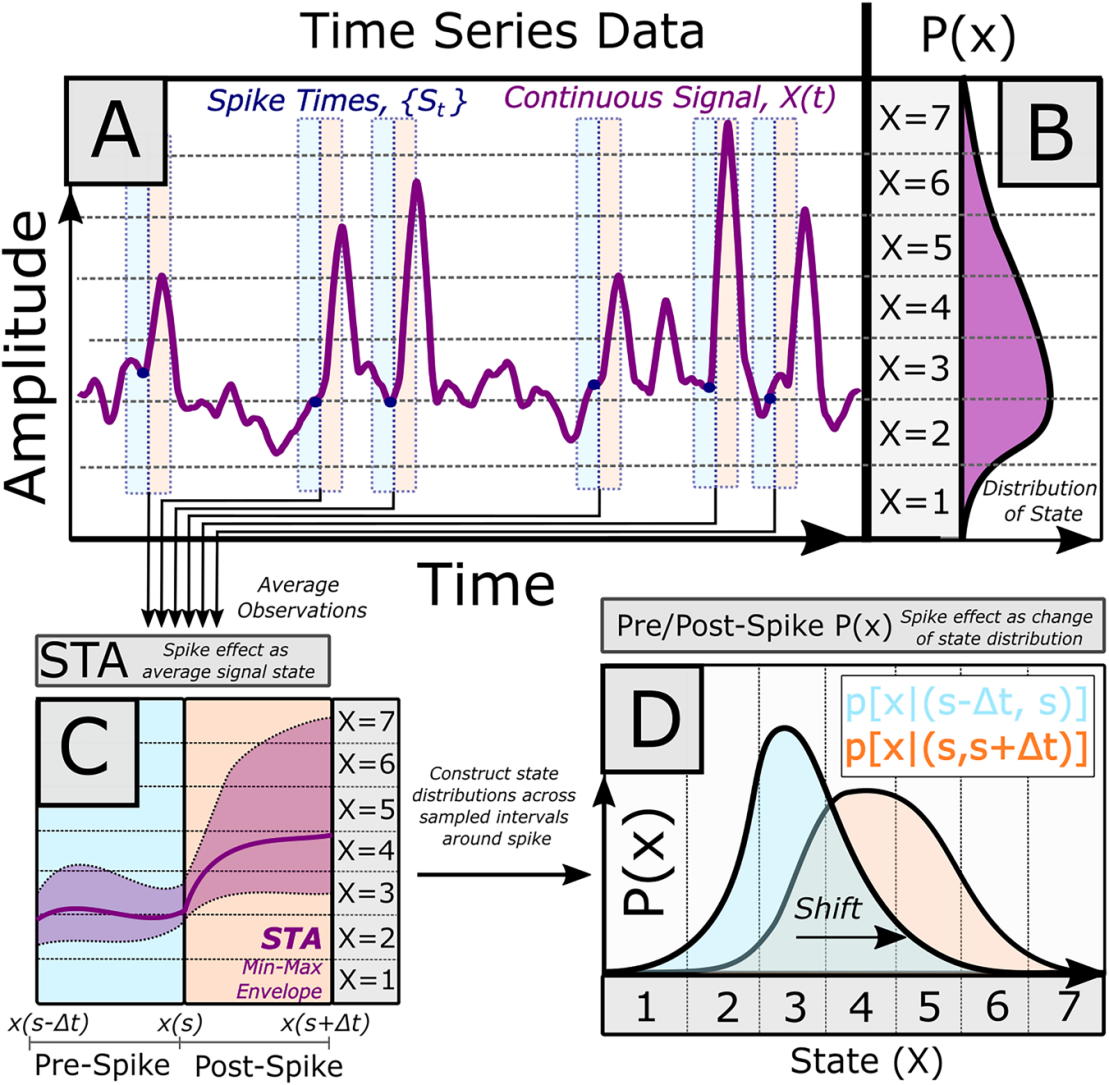
***A***, Electrophysiological recordings presented as time series data: Time-varying amplitude values collected over a duration. The relationship of a point process, such as a spike train, and a time series is often evaluated as the spike-triggered average (STA). Here, intervals of the continuous signal are sampled around spiking events, with samples averaged together. ***C***, Under the STA framework, if a spike–signal relationship is significant, an “impulse response” may be present, showing a difference in the average signal behavior in the pre-spike versus post-spike averaged interval. ***B***, A complementary approach is to treat the electrophysiological signal as a discrete random variable. By applying a quantization scheme, continuous signal amplitudes may be converted into a discrete time series of finite signal “states.” This permits signal behavior over an interval to then be described as a probability distribution of signal state. ***D***, As with the classical spike-triggered average (***C***), the relationship between a spike and signal may be discerned from intervals of the discrete signal before and after spike. The analogous spike-triggered average probability distributions describe signal state prior to, and after, a spike. The correlated effect of a spike event on signal behavior can thus be described as the shift in the spike-triggered pre-spike and post-spike signal distributions.

The time series data used in the STA comprises signal amplitude, *x*, indexed at time *t*, i.e., *x*(*t*) (generally, *t* is collected at the limit of digital signal sampling in the recording system; Fig. 3*A*). To mechanistically compute the STA, the pre-spike and post-spike signal levels are sampled over some finite interval, Δ*t*, relative to spike time, *s*. For every time point relative to a spike, $t_{i}\in (s-\Delta t,s+\Delta t)$, the concurrent signal amplitude, *x*(*t_i_*), is averaged over all spiking events to find the mean, *x̄*(*t_i_*) (Fig. 3*C*). These signal waveforms may be variously preprocessed prior to this averaging operation (e.g., mean-leveling) to further isolate spike-associated correlations. Performing this operation on all relative time points thus generates a mean waveform, *x̄*(*t*), which is effectively the signal impulse response to the spiking impulse.

In this framework, the neuron's contribution to the correlated signal (or the signal's effective stimuli, if testing how signal facilitates a spike in reverse correlation) is the convolution of the spiking impulses with the expected impulse response (i.e., the STA). Here, spikes are implicitly considered as independent and equivalent events with the spike rate reflecting neural excitation (i.e., the neuron behaves within the assumptions of the linear–nonlinear Poisson model; Schwartz et al., 2006). If these assumptions are upheld, then the STA should provide the maximum likelihood estimation of the spike–signal correlation (Paninski, 2004).

These assumptions bear consideration. Is a neural spike equally potent in predicting future signal state across all conditions? An initial exploration of this question may be directed at the covariate signal itself: Does the spike–signal correlation depend on signal amplitude? It is important to recognize that the operation for computing the STA enforces a type of dimensionality reduction: For each position in time relative to spike, *t*, the ensemble of observed signal responses forms a distribution of amplitudes, *X*(*t*). By definition, the classical STA merely utilizes the mean of this distribution, a single value, when reporting the “average” response at *t*. Further, this distribution is often assumed to be Gaussian (Normal) for statistical significance. If the STA is calculated from the change in signal amplitudes (i.e., Δ*x* instead of *x*), then any consideration of amplitude-dependent spike effect correlations is also lost. In estimating a single response, the STA thus contains less complete information about signal behavior at time *t* compared with the full distribution of signal values around the spike.

#### The spike-triggered average distribution of signal states

**Figure 4.**
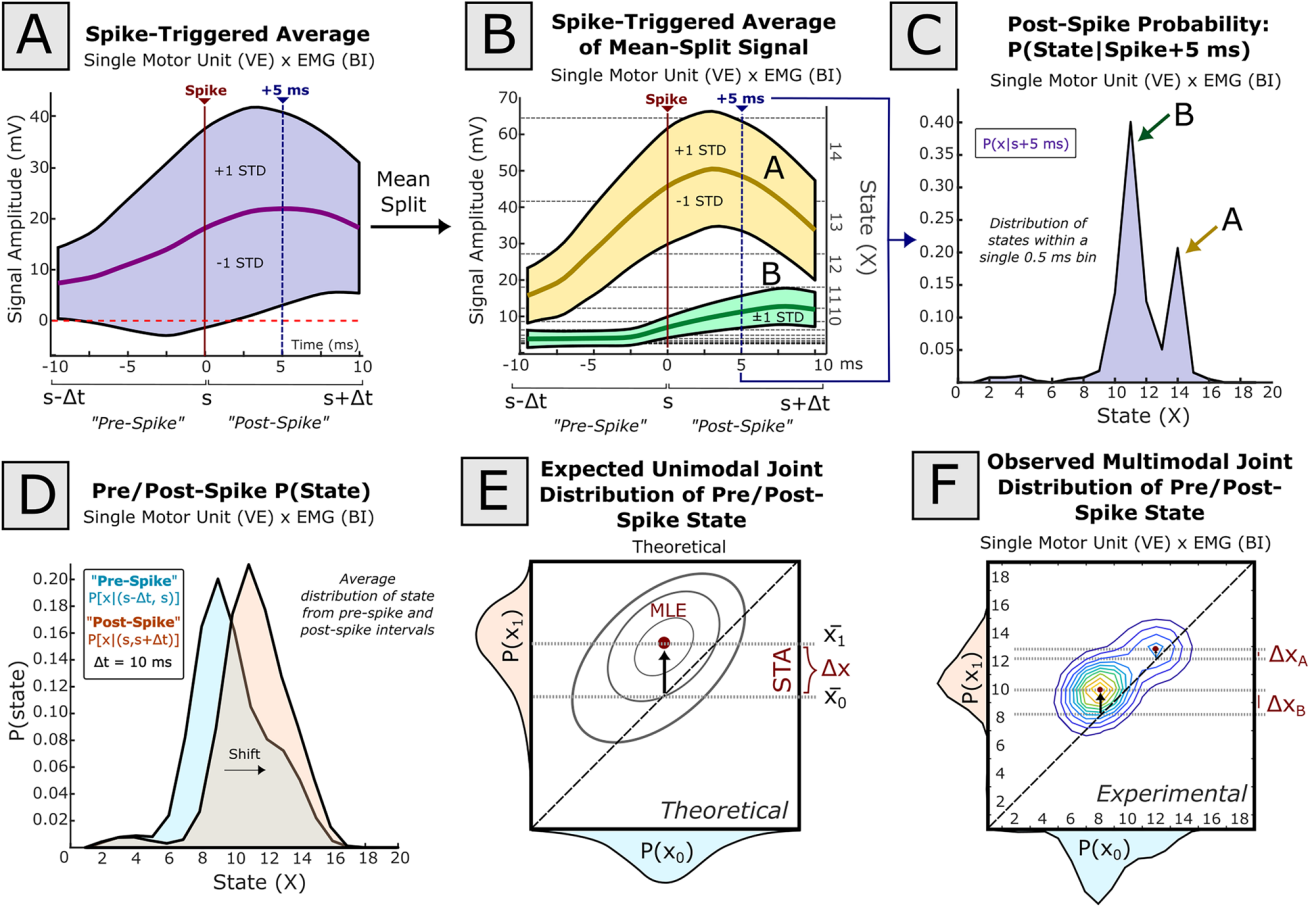
The spike-triggered average (STA) of a signal may vary based on background signal behavior. In this example, spiking events from a single motor unit recorded in the vastus externus (VE) muscle were compared against the aggregate EMG signal recorded from the biceps femoris (BI). A window 10 ms before spike to 10 ms after spike was used for identifying spike-triggered effects. ***A***, The simple spike-triggered average of BI EMG at spike (red line) within this window has wide variability. The mean STA signal is given as a thick purple line, while ±1 standard deviation of the windowed signal is described by the breadth of the colored envelope. ***B***, Separating the aggregate STA by whether the signal is above or below the mean aggregate STA (***A***) at time of spike provides two STAs (STA-A, STA-B) which exhibit different behavior. ***C***, Signal amplitudes were converted to discrete signal states using a logarithmic binning scheme. By taking a histogram of binned signal amplitudes (“states”) at an arbitrary single time point (here, 5 ms after spike; position indicated by dashed blue vertical lines in ***A*** and ***B***), this bimodality corresponding to STA-A and STA-B is also seen in state space. ***D***, If signal amplitude over the entire perispike interval is quantized into amplitude states, the signal amplitude in the pre-spike and post-spike intervals may be represented as a pre-spike and post-spike distribution of state. ***E***, When pre-spike and post-spike distributions are described using a joint probability distribution, the theoretical peak value corresponds to the maximum likelihood estimate (MLE) of the post-spike state given pre-spike state. Here, in theoretical data, the STA effect is the migration of the peak from the matrix diagonal (i.e., the average change in state). ***F***, If, however, the distribution of pre-spike or post-spike signal distributions are multi-peaked, as in this physiological example (***A–D***), transitions to the global peak (*X_B_*; STA-B) may be suboptimal to describe transitions around local peaks (*X_A_*; STA-A). In this example, the best prediction of post-spike state is contingent on whether pre-spike state is closer to 8 or 12.

We here propose a method of utilizing the complete spike-triggered state probability distribution to describe spike-triggered effects. Our process is as follows: We first apply an amplitude binning scheme (i.e., quantization) to our recorded time series data (e.g., EMG), such that each observation of the recorded signal amplitude may be assigned to a single discrete amplitude bin, *x_i_*. [The number of such bins can be chosen for the signal based on signal range and precision required (Fig. 3*A*,*B*).] Here we define signal “state” as a binned range of signal amplitudes, resulting from the quantization scheme applied to the original, continuous, time series data. (Hence, “higher states” correspond to higher levels of original signal amplitude.) Here, the frequency of observing state over the entire time series is described by the distribution *P*(*x*) (Fig. 3*B*). Next, we infer the spike-triggered effect, classically inferred from the change in the amplitude (waveform) before and after a spike as the change in the distributions of signal amplitude around each spike (Fig. 3*D*). As with the classical STA, signal is sampled over a short interval (*s *− Δ*t*, *s *+ Δ*t*) relative to the spike event, *s* (Fig. 4*A*). By quantizing signal into discrete states, potentially non-normally distributed signal amplitudes in the STA (Fig. 4*B*) can be described explicitly for any time point, or interval of time, as a distribution of state (Fig. 4*C*). We define the pre-spike distribution as the distribution of discrete signal states over the entire sampled interval leading to a spike p[x,(s−Δt,s)], and the post-spike distribution as the distribution of states in the entire chosen sample interval after a spike, p[x,(s,s+Δt)] (Fig. 4*D*). For brevity, we will represent the discrete pre-spike and post-spike distributions as $p(x_{0})$ and $p(x_{1})$, respectively. Thus, the effect of a spike, classically captured in the STA as the change of mean signal amplitude in time, may be equivalently described as the change of the probability distributions relative to a spike, Δp(x) (Eq. 1).

$$p(x_{1})-p(x_{0})=\Delta p(x).$$

Practically, because the signal has been quantized into a finite number of states, the pre-spike, post-spike, and difference probability distributions are each captured in finite length vectors. The average pre-spike, post-spike, and difference distributions vectors could then be averaged across spiking events, as with the STA.

In this formulation, the maximum likelihood estimation of signal state around a spike in quantized state space is given as the global maximum of the joint distribution of the pre-spike and post-spike state distributions (Fig. 4*E*). By this description, the STA corresponds to the movement of the global mean off the joint state diagonal (i.e., the difference in the average pre-spike and post-spike state), which assumes a stationary and unimodal outcome. If, however, $p(x_{0})$ or $p(x_{1})$ are not unimodal (as in this example taken from physiological data), local maxima may exist in the joint distribution, independent of the global maximum (Fig. 4*F*). In this case, clearly, consideration of the pre-spike state can better inform predictions of post-spike state distributions than the average of Δp(x) alone. In these instances, the classical STA may perform as a suboptimal description for spike–signal prediction.

#### Stochastic dynamic operator framework

To capture state-dependent relationships in spike–signal correlations more precisely, measures of the changes from pre-spike to post-spike signal distributions should also be state sensitive. Specifically, we desire to know $p(x_{1})=\underset{x}{\sum }\;p(x_{1}|x_{0})p(x_{0})$. That is, we seek the post-spike distribution, given the conditional probability of signal state after a spike and the prior pre-spike signal state distribution.

To map the state-dependent shift in pre-spike signal state distribution, we define a linear operator, *L*. *L* is implemented as a matrix which uses the pre-spike state distribution $p(x_{0})$ to generate a column vector providing the change in the state distribution, Δp(x), given an observed pre-spike probability distribution (Eq. 2).

$$\Delta p(x)=Lp(x_{0}).$$

The relationship between the pre-spike and post-spike distributions can be described via the simple update $p(x_{1})=p(x_{0})+\Delta p(x)$. By combining and rearranging Equations 1 and 2, the post-spike state distribution may be predicted from a given pre-spike state distribution by operator *L* (Eq. 3).

$$p(x_{1})=Lp(x_{0})+p(x_{0}).$$

Next, we need to derive *L*. Theoretically, *L* may be recovered as a standard linear estimate across all observed pre-spike to post-spike distributions. Here, we further elaborate on this process. Ultimately, Equation 14 is the main result of this derivation. For a set of *k* spikes, we may define the sampled ensemble of pre-spike distributions as the vector concatenation $P(x_{0})=[\;p{\left(x_{0}\right)}_{1}\ldots p{\left(x_{0}\right)}_{k}]$, the post-spike distributions as $P(x_{1})=[\;p{\left(x_{1}\right)}_{1}\ldots p{\left(x_{1}\right)}_{k}]$, and the change in state distributions as $\Delta P(x)=[\;p{\left(\Delta x\right)}_{1}\ldots p{\left(\Delta x\right)}_{k}]$. For *n* total states and *k* spiking events, this produces *n*-by-*k* matrices. *L* is then estimated as follows:

$$\Delta P(x)\approx LP(x_{0}).$$

Postmultiplying by the transpose of the pre-spike distribution establishes the following:

$$\Delta P(x)P(x_{0}{)}^{T}=LP(x_{0})P(x_{0}{)}^{T}.$$

This could, in principle, be simplified to a standard linear regression problem as follows:

$$R_{xy}=LR_{xx},$$

where $R_{xy}=\Delta P(x)P(x_{0}{)}^{T}$and $R_{xx}=P(x_{0})P(x_{0}{)}^{T}$ and are square matrices of order *n*. *L* could thus be derived as follows:

$$L\;=\;R_{xy}\;R_{xx}^{-1}.$$

However, the linear estimation of the SDO derived in Equation 7 is often ill-posed because the covariance matrix of pre-spike state distribution (*R_xx_* in Eq. 7) may be noninvertible. Because this matrix often could not be defined in our datasets via Equation 7, we developed an alternative linear estimation method, which we will describe below. To introduce that method, we must consider how *L* behaves. In practice, *L* is a square matrix whose order equals the number of signal state levels used for analysis. It provides the averaged spike-triggered change in probability of state, given pre-spike state, as estimated from the observed data (Eq. 4). Thus, *L* is effectively a kind of state-dependent spike-triggered average, albeit for state distributions rather than waveforms. Because *L* operates on probability distributions to describe stochastic signal dynamic changes, *L* is termed as SDO (see Sanger, 2011 for development and definitions). SDOs were originally developed to describe and manipulate continuous-time stochastic dynamics governed by differential equations. Here, we use the properties of the discrete time SDO to create a difference equation which describes how the spike-triggered average change in probability of state depends on the prior state.

The matrix operator, *L*, is not uniquely defined. The SDO matrix represents a “probability flow” from the pre-spike to the post-spike distribution. Probability flows out from each state *j*, to other states *i*, i≠j. The total outward and inward flow component from each state must sum to 0 to conserve the total probability, and states cannot increase their own probability by flowing into themselves. Four conditions are necessary and sufficient for an SDO matrix to behave as a linear operator (Sanger, 2011): (1) The matrix diagonal must contain nonpositive elements, (2) off-diagonal elements must be non-negative, (3) each column must sum to 0 (if the SDO is modeled as a left-stochastic matrix, as used here), and (4) the sum of positive elements within a column must be no greater than 1. From the perspective of a single initial state, *x*_0_ = *j*, these constraints mean that the change of probability of maintaining state at *x*_1_ must be nonpositive $\left(\Delta p(x_{1}=j|x_{0}=j)\leq 0\right)$, while the change of probability of transitioning to any other subsequent state must be non-negative; $\Delta p(x_{1}\neq j|x_{0}=j)\geq 0$ (i.e., one may only increase, or sustain, the probability of transitioning to another state and only decrease, or sustain, the probability of maintaining state). A valid estimation of the SDO must thus uphold these constraints.

#### SDO matrix linear estimation method

Having identified how the SDO matrix should behave, here we describe an alternative equation which efficiently generates compliant SDO matrices regardless of input state probability distributions and bypasses the invertibility problem faced by the classical least-squares method in Equations 6 and 7. In Equation 2, the SDO is a matrix that provides the change in the state probability, $\Delta p(x)=Lp(x_{0})$ while upholding the listed constraints. If *R_xx_* cannot be directly inverted to derive *L* via Equation 7, let us instead consider when *R_xx_* is not necessary. When the pre-spike state distribution is binary, a compliant SDO matrix (*L*) can always be estimated from the outer product of the change in state distribution and the binary pre-spike state distribution (Eq. 8). This corresponds to *R_xy_* given in Equation 6.

$$L=\Delta p(x)p(x_{0}{)}^{T}.$$

In Equation 1, this can alternatively be represented as the difference of the initial distributions:

$$L=\;[\;p(x_{1})-p(x_{0})]\;p(x_{0}{)}^{T}.$$

However, when *p*(*x*_0_) is not binary and |Δp(x)|>0 (as is usually the case), Equations 8 and 9 produce a matrix with positive elements occurring on the diagonal and negative elements elsewhere than the diagonal. Hence while the vector Δp(x) can be predicted from the resultant transformation matrix (i.e., upholds Eq. 2), it is not an SDO. However, a compliant SDO can be generated by the sum of SDO matrices generated for each of *n* discrete states in the pre-spike distribution (Eq. 10). (This is equivalent to iteratively and independently populating each column of the SDO in Eq. 9.)

$$L=\;\overset{n}{\underset{\;j=1}{\sum }}\;[\;p(x_{1})-p(x_{0}=j)]\;p(x_{0}=j{)}^{T}.$$

Here, the probability of state *j* in the pre-spike state distribution is given as $p(x_{0}=j)$, and this distribution contains zeros everywhere except at element *j*. (Alternatively, this can be thought of as the summation of *n* SDO matrices generated by Eq. 8 via binary pre-spike distributions, with the *j*th SDO weighted by the probability of observing state *j* in the pre-spike interval.) Because vector $p(x_{0}=j)$ contains a single nonzero element, the *j*th term only populates column *j* of the SDO matrix in the net summation, thus each term is effectively independent. This technically permits a special case where the outer product can be distributed to the minuend and subtrahend. Thus, Equation 9 may be rewritten as follows (Eq. 11):

$$L=\;\overset{n}{\underset{\;j=1}{\sum }}\;p(x_{1})\;p(x_{0}=j{)}^{T}-p(x_{0}=j)\;p(x_{0}=j{)}^{T}.$$

The sum of the differences may be rewritten as the difference of two sums (Eq. 12):

$$L=\;\overset{n}{\underset{\;j=1}{\sum }}\;p(x_{1})p(x_{0}=j{)}^{T}-\overset{n}{\underset{\;j=1}{\sum }}\;p(x_{0}=j)p(x_{0}=j{)}^{T}.$$

Here, because the *j*th term of the left sum of outer products again independently contributes to the *j*th column of the matrix, the summation is equivalent to the outer product of the pre-spike and post-spike distributions directly, $p(x_{1})p(x_{0}{)}^{T}$. Similarly, the *j*th term generated via the outer product operation of binary vectors in the right sum only operates on a single element on the array diagonal at *L_jj_*. Hence, the right summation produces a diagonal matrix, whose diagonal contains the pre-spike distribution. By employing these two simplifications, we can then derive *L* as follows (Eq. 13):

$$L=\;p(x_{1})p(x_{0}{)}^{T}-\mathrm{diag}(\;p(x_{0})).$$

This form utilizes the original pre-spike and post-spike state probability distributions directly without any further requirements. Finally, we average the SDO matrix across all spikes. For *k* spiking events, if the ensemble of pre-spike distributions is provided via the vector concatenation $P(x_{0})=[\;p{\left(x_{0}\right)}_{1}\ldots p{\left(x_{0}\right)}_{k}]$, and the ensemble of post-spike distributions is the similar concatenation $P(x_{1})=[\;p{\left(x_{1}\right)}_{1}\ldots p{\left(x_{1}\right)}_{k}]$, the SDO is computed as follows:

$$L=\;\frac{1}{k}\left(P\left(x_{1}\right)P{\left(x_{0}\right)}^{T}-\text{diag}\sum P\left(x_{0}\right)\right).$$

Equation 14 is the important final result that serves as the basis of the SDO analysis used below and produces compliant SDOs which uphold both Equations 2 and 3. It is direct and algorithmically straightforward once formulated this way. The prior development equations are important to understand how Equation 14 was derived, but Equation 14 is the main one for the reader less interested in the derivation methods.

A further technical point can be made. Due to the structural assumptions made in Equation 10, and consistent with Sanger, by this formulation of Equation 14, for each spike observed, every state in pre-spike distribution probabilistically flows to all states in the observed post-spike distribution. Consequently, when calculated for a single spike, the columns of the SDO matrix are substantially identical, save for the matrix diagonal, with the *j*th column scaled according to the probability of observing state *j* in the pre-spike distribution, $p(x_{o}=j)$. State-dependent behavior of the final SDO matrix, averaged over these per-spike effects, is thus not derived from the sum of state-dependent transitions of pre-spike and post-spike distributions by individual spiking events, but rather emerges from differences in sampled pre-spike and post-spike distributions of data across the entire spike train.

Next, we elaborate SDO analysis as a flexible tool to describe stochastic behavior correlations within simulated and physiological data. The SDO methods and analyses described below, including the implementation of Equation 14 for SDO matrix estimation, were generated using the *SDO Analysis Toolkit*, a package we wrote and assembled. Analyses presented were generated in MATLAB 2023a, running on a Dell Precision Tower 5810 using an RTX3060 GPU with a Windows 10 operating system.

##### Model hypotheses

To compare the spike-triggered SDO with classical methods, we explored trying to fit the different test data under several model method hypotheses, including the STA. We tested both real physiological and model “physiological data” (in which the ground truth generator mechanisms originating the data were known). Each combination of spiking unit and stochastic signal in our datasets was fit using seven different SDOs, describing seven statistical models (below). Each of the seven method hypotheses explored was embodied as a matrix operator predicting the change in the predicted post-spike signal state distribution, $\Delta \hat{\;p}(x)$, given the pre-spike state distribution. (Further, all seven hypothesis matrices also satisfy the constraints to behave as SDOs.) The predicted post-spike state distribution was then estimated with the observed pre-spike distribution via the update equation $\hat{\;p}(x_{1})=p(x_{0})+\Delta \hat{\;p}(x)$. For each model, we tested the predicted post-spike state distribution, and most probable post-spike state, and compared these predictions with the observed post-spike distributions and states. Prediction matrix method hypotheses were as follows:

1. [H1] Constant $\Delta \hat{\;p}(x)=0$: This is the null hypothesis of no effect and that the data has no statistical drift. The hypothesis matrix is empty (all zeros), hence the predicted post-spike state distribution, $\hat{\;p}(x_{1})$, matches the pre-spike distribution, $p(x_{0})$, exactly. This may occur when signal state changes at relatively low frequency (e.g., no change over the windowed sample), or completely at random (e.g., white noise), and the spike has no correlation to signal behavior. In terms of the classical STA, this would be equivalent to no expected “effect.” This may occur with a flat average waveform in the pre-spike and post-spike interval but can describe any waveform where the average state does not differ between the pre-spike and post-spike interval.
2. [H2] Diffusing in time $\Delta \hat{\;p}(x)=(G-I)p(x_{0})$: Here, *I* is the identity matrix and *G* is a matrix generated by convolution of a Gaussian kernel with the identity matrix. In this model, the predicted post-spike distribution reflects diffusion of the pre-spike distribution. This is the null hypothesis of no effect but that the data also has a random statistical drift. The predicted post-spike distribution has increased variance relative to the pre-spike distribution but no change of mean. This may occur if spike is correlated with an unpredictable or randomly fluctuating polymodal spike impulse response, which increases the variance of the post-spike distribution when accumulated across time. In both cases, there is no change in the “average” state in the interval. If spike effects were consistent, there would instead be a clear impulse response in the time domain (e.g., the STA).
3. [H3] Predicted by a spike time alone $\Delta \hat{\;p}(x_{1})=(\bar{P}(x_{1})-I)p(x_{0})$: Here, *I* is the identity matrix and $\bar{P}(x_{1})$ is the average sample post-spike distribution. This hypothesis is the classical STA, expressed within state probability space. Here, prior state is assumed to have no effect on the resulting spike effect. This matrix causes the post-spike distribution to become the average post-spike distribution. Equivalently, this is the change in the post-spike distribution generated from the average post-spike waveform.
4. [H4] Predicted only by prior state independent of spike $\Delta \hat{\;p}(x_{1})=\;L_{B}p(x_{0})$: Here, $L_{B}$ is a “background” SDO estimated over all time points in the signal, rather than only at time indices associated with spike. The same “pre-spike” and “post-spike” interval durations are used for “spike-triggered” responses (although here, all time indices are treated as a “spike”). This hypothesis matrix permits a signal to have intrinsic (“background”) dynamics independent of spiking effects and hence demonstrates state-dependent behavior. It can thus be applied to any arbitrary time point of a dynamic signal. This condition may arise, for example, if the system stabilizes a signal around a fixed point, converging onto a value from multiple potential initial states. In this model state dynamics are partly determined by prior state, but spike occurrence has no effect. This also forms the “background SDO” in statistical tests used below, as deviation of the spike-triggered SDO (model H7) from the “background” SDO (model H4) indicates a difference in the local system dynamics specifically near the spike occurrence.
5. [H5] Predicted by prior state dynamics in the pre-spike interval $\Delta \hat{\;p}(x_{1})=(M_{0}^{\Delta t}-I)p(x_{0})$: Here, $M_{0}$ is a first-order Markov matrix describing successive state transitions between the *n* discrete time points in the pre-spike interval. Matrix *M*_0_ is estimated using only data from the pre-spike intervals from all spiking events of the sampled neuron. In model H5, we estimate local probability dynamics prior to spike and assume these are conserved after spike. Here, the sequential state transitions composing the observed time evolution of state are treated as independent and independent of spiking. To predict the post-spike state distribution over an interval Δ*t* composed of *T* discrete time points, the results of the iterative application of the Markov transition matrix *M*_0_ over 1 to *T* steps are averaged together: $M_{0}^{\Delta t}=\frac{1}{T}\sum_{t=1}^{T}\;M_{0}^{t}$. The Matrix $M_{0}^{\Delta t}$ then serves as a left transition matrix predicting the post-spike state distribution over the entire interval Δ*t*. For consistency with other methods, the change in the pre-spike probability distribution provided by the transition matrix is returned by subtracting the identity matrix (*I*) from the averaged Markov matrix $\left(M_{0}^{\Delta t}-I\right)$. In model H5 we thus estimate the Markov probability dynamics between time points in the interval prior to spike, and we assume these dynamics are conserved after spike. The state transitions composing the time evolution of state in the predicted post-spike interval are treated as independent Markov transitions determined by *M*_0_ and are therefore independent of spiking.
6. [H6] Predicted by prior state, plus a constant effect following a spike $\Delta \hat{\;p}(x)=(L_{B}+\Delta \bar{P}(x))p(x_{0})$: Here, $L_{B}$ is an SDO estimated over all time points in the signal (i.e., H4), and $\Delta \bar{P}(x)$ is the average change in the pre-spike to post-spike distribution, across all samples (i.e., the spike-triggered change). This hypothesis combines the independent state-dependent dynamics from H4 with a STA. Here, the STA effect is treated as state-independent after the spike, integrated into the existing, spike-independent, background dynamics.
7. [H7] Predicted by prior state and spike (spike-triggered SDO) $\Delta \hat{\;p}(x_{1})=Lp(x_{0})$: In this model, the prior state and spike occurrence have interacting effects and are captured using the spike-triggered SDO matrix, *L* (Eq. 2). In H7, spikes are hypothesized to change the local background signal dynamics, which may manifest as different spike-correlated shifts of the post-spike distribution and may be state dependent. For example, a spike may be associated with transitions toward a given state, with directional shifts in the pre-spike distribution conditional on whether the signal is ascending or descending toward this state or with multimodal increased probability of multiple states.

Each model hypothesis method examined here assumes a different relationship between a spike source and signal. For example, the H4 method assumes that the signal state is functionally independent of spiking behavior. The H6 method assumes that signal state behavior correlates with spike sampling times in a state-dependent fashion but that spike effects themselves are additive and are not state dependent. The H5 method assumes that the spike is not a causal agent, although spike occurrence is used to determine epochs where dynamics are sampled. Insofar as the relationship between randomly sampled interneurons and electromyograms may vary, we expected that arbitrary combinations of spike sources and signals in our recorded data could likely be best-modeled by different hypotheses.

#### Validation of SDO methods in simulated data

**Figure 5.**
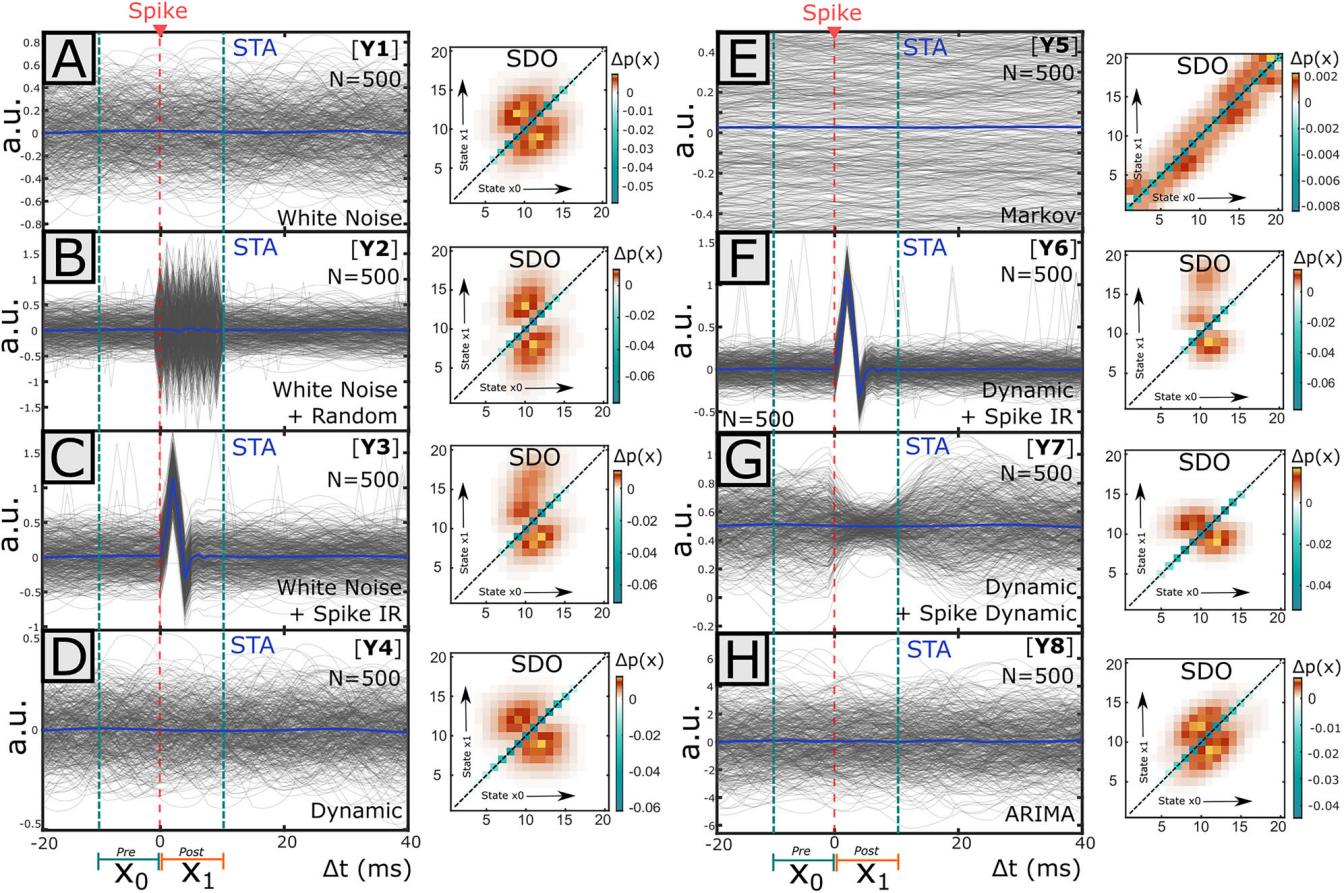
Comparison of the STA impulse response and SDO matrices obtained from simulated stochastic control data. Time series and point process data were simulated with different stochastic processes and spike–signal control relationships (or lack thereof). For each simulated spiking event, a sample of time series data was extracted in the window 10 ms before spike to 10 ms after a spike. The STA impulse response was estimated as the average signal amplitude across all spike-triggered samples. The pre-spike window was defined as 10 ms before spike until the time of spike, and the post-spike interval as the 10 ms following the spiking event. In each case, the SDO was generated using the distribution of states extracted across each interval. Twenty states were linearly defined from the minimum and maximum of the observed signal values. Time series were stationary about a mean value (usually zero), with similar amplitude ranges on either side of the mean. Hence, the average signal level corresponds to an intermediate signal state (i.e., 10–11), and SDOs had values around the middle of the matrix. Representative examples of STAs and SDOs from simulated data are plotted here. The 500 spike-triggered window signals are overlaid as gray traces in time. ***A***, [Y1] Time series was generated using low-pass filtered white noise. Spikes have no effect. STA is flat and SDO is symmetrical about the diagonal. ***B***, [Y2] Spikes evoke increased noise level impulses in otherwise white noise. STA is flat, while the SDO is elongated in the horizontal dimension (corresponding to the increased signal variance after spike). ***C***, [Y3] Spikes have consistent effects within a white noise signal. STA extracts the impulse response. The SDO is elongated above the matrix diagonal, showing an increase in transition toward higher states (amplitudes). ***D***, Signal is generated by a stabilizing dynamic equation. Spike has no effect and STA is flat. The SDO demonstrates more stabilizing effects than in white noise (Y1); above-average input states (11–15) are pushed toward lower states (5–10) while below-average input states (5–10) are pushed toward higher states (10–15), as indicated by SDO elements. ***E***, [Y5] Markov process. Subsequent signal amplitude transitions to similar amplitude as prior amplitude. Spike has no effect. STA is flat. The SDO indicates that changes in signal distribution at spike time are minor and bidirectional (indicated by the limited band on the matrix diagonals). ***F***, [Y6] Spike has consistent effect within a process described by a stabilizing dynamical equation. As with Y3, STA extracts the impulse response and SDO is elongated. ***G***, [Y7] Spike induces dynamic effects, while signal is also generated by a stabilizing dynamic equation. STA is flat. SDO is elongated in the horizontal dimension, indicating pre-spike signal variance is less than post-spike variance. ***H***, [Y8] Signal is generated from an ARIMA(3,2) process. STA is flat. As with the white noise (Y1), the SDO is relatively symmetrical about the matrix diagonal.

We first tested our inference that the classical spike-triggered average method may be insufficient to consistently detect state-dependent or paradoxical interactions within the nervous system using computational models of different stochastic processes in silico. We generated data via well-characterized systems and assessed the statistical specificity and sensitivity of the STA and SDO methods to detect these relationships. Eight methods of generating stochastic time series data (Generators Y1–Y7), corresponding roughly to the seven hypotheses (above) and an additional ARIMA process (Y8), were used. To correlate spike-triggered effects, one putative spike train was drawn from random indices for each simulation, in common for all the time series methods (Generators Y1–Y8). If a simulated stochastic time series had a spike-triggered effect (Generators Y2, Y3, Y6, Y7), the associated spike effects were applied at these time indices. We describe these stochastic generator processes as follows:

1. [Y1] Low-pass filtered white noise: Pre-spike and post-spike distributions of state should not systematically vary. Spike has no effect (Fig. 5*A*).
2. [Y2] Low-pass filtered white noise with spike-triggered random effects: This control is similar to Y1, but at time of spike, there is an added higher amplitude random (white noise) response added during the 10 ms duration after spiking event. The effect of spike increases signal variance (Fig. 5*B*).
3. [Y3] Low-pass filtered white noise with consistent spike-triggered control effects: This is the same as Y1, but with an added consistent impulse response during the 10 ms following time of spike. The spike has a consistent effect (Fig. 5*C*).
4. [Y4] Low-pass filtered white noise with stabilizing dynamics independent of spikes: Here, the signal was iteratively synthesized using the update equation $Y_{4}[t]=Y_{4}[t-1]+\Delta Y_{4}[t]$. To maintain consistency between generator models, for every time *t*, we utilized the differential of Y1 to derive the update to Y4 $\left(\Delta Y_{4}[t]\right)$. This generator model was stabilized toward a fixed point using the dynamic equation: $\Delta Y_{4}[t]=k(x_{0}-Y_{4}[t])+\Delta Y_{1}[t]$. Here *X*_0_ is a fixed point (0), and *k* is the stabilizing coefficient (1 > *k* > 0). Essentially, this equation biases future signal toward zero, with an effect magnitude proportional to its current distance from zero. The differential of the Y1 signal $\left(\Delta Y_{1}[t]\right)$ is thus used here as an added random noise term. Spike has no effect (Fig. 5*D*).
5. [Y5] Markov process independent of spikes: Here, a sequence was generated via a random walk of a Markov matrix. The Markov transition matrix was composed of a 100 × 100 identity matrix convoluted with a Gaussian kernel, biasing transitions to nearby states. The signal was mean-leveled around zero. Spike has no effect (Fig. 5*E*).
6. [Y6] Stabilizing dynamics with consistent spike-triggered effects: This is the same as Y4, with the spike impulse responses of Y3 added. Spike has a consistent effect (Fig. 5*F*).
7. [Y7] Stabilizing dynamics with spike-triggered dynamics: This is the same as Y4; however, in the 10 ms duration after the spike, a second stabilizing equation is additionally active (of the same form in Y4). For the spike-triggered dynamics, the equilibrium of the control is positive, *X*_0_ > 0, such that background dynamics and spike-triggered dynamics stabilize toward different points. A spike activates a consistent change in dynamics (Fig. 5*G*).
8. [Y8] Autoregressive Integrated Moving Average (ARIMA) stationary stochastic process: ARIMA models are commonly used to parsimoniously describe and forecast stochastic time series data. Here, a stationary ARIMA(3,2) model was used. The signal was mean leveled around zero. Spike has no effect (Fig. 5*H*).

We compared the STA and SDO methods of analyzing data from these various generators’ simulations of model systems in which spikes were independent of dynamics or participated in the control of dynamics.

#### Computational implementation of the STA methods

We used three methods of calculating the spike-triggered average between a spike and (simulated) rectified EMG and one measure of spike tuning. A spike-triggered effect was considered “significant” if any test of effect returned a positive result using *α* = 0.05. To avoid spurious correlations, only spike trains with a minimum of 500 spikes were included for analysis.

##### Simple effects

This is a simple test of correlation in signal amplitude relative to spike correlations. The average signal amplitude in each 20 ms pre-spike interval and the 20 ms post-spike interval was captured for all spiking events. We then tested the null hypothesis that the difference in the distribution of average signal amplitude in the pre-spike and post-spike interval was normally distributed around zero (i.e., a paired *t* test).

##### Detrended effects

The increment-shifted average (ISA) technique attempts to isolate spike-correlated effects in signal from potentially slower curvilinear trends in signal amplitude preceding and following spike (Davidson et al., 2007). In this framework, for every spike, *s*, a segment −40 ms prior to and 100 ms after the spike is extracted from the signal amplitude. Then, for every discrete time increment t∈[s−20ms,s+40ms] relative to a spike, *s*, a 60 ms subsample of this segment was extracted [*t *− 20 ms, *t *+ 40 ms] (e.g., sample 1 is taken as [*t *− 40 ms, *t* + 20 ms], sample 2 is [*t *− 39 ms, *t* + 21 ms], etc.) The average of these waveforms is the ISA. Subtraction of the increment-shifted average waveform from the spike-triggered signal in the window 20 ms prior to and 40 ms after spike may better isolate spike-correlated events relative to slower-resolving background behavior. The mean of the pre-spike and post-spike expected baseline windows (20–0 ms before the spike; 20–40 ms after the spike) is then subtracted from the mean of the ISA-subtracted window in the test interval (0–20 ms after spike), isolating the average change of signal specific to this interval (Poliakov and Schieber, 1998). We then tested the null hypothesis that the resultant distribution of spike effects is normal and centered around zero.

##### Bootstrapped deviation

The Chronux MATLAB package (Mitra and Bokil, 2008; Bokil et al., 2010) similarly tests for significant spike-triggered average relationships by collecting pre-spike (20–0 ms before the spike) and post-spike (0–20 ms after the spike) windows. The standard deviation of relative signal amplitude across the population of *N* spikes at every relative time index, *t*, is estimated using the average waveforms from 20 bootstrapped populations of *N* spike indices (i.e., resampling with replacement). A spike-triggered average relationship was considered significant if the rectified, mean-leveled, STA waveform had 1 or more points above threshold anywhere within the interval *t* = [−20 ms, +20 ms] relative to a spike. Threshold was defined by calculating the inverse *t* statistic after Bonferroni’s correction to the familywise error rate for the number of points tested. (This corresponded to ∼2.99 standard deviations for 40 points.)

#### Computational implementation of SDO methods

To generate, visualize, and analyze SDOs, we developed the *SDO Analysis Toolkit*, written in the MATLAB programming language. For each time series signal (Generators Y1–Y8 in simulated data, or rectified aggregate EMG in the physiological data), signal states were defined as 20 intervals between the channel-wise minimum and maximum values of signal amplitude observed over the entire dataset. (Note that simulated data used a linear scale while in the collected EMG data we used a logarithmic scale, based on observed signal amplitude distributions.) Under the SDO model, each spike was treated as an independent event. For each spike, we extracted signal states in a short interval (10 ms) before (“pre-spike”) or after (“post-spike”) the spike event. The frequency histogram of states within these observed intervals relative to spike comprised the pre-spike and post-spike probability distributions. The pre-spike distribution included the time bin containing the timestamp as the final element. SDO matrices were then generated for each combination of spiking unit (i.e., neuron) and signal. Note that as a first demonstration of the SDO methods explored here, we consistently used 20 states for quantizing signal amplitude, and pre-spike and post-spike durations of 10 ms each, although these parameters are among those which may be readily modified within the *SDO Analysis Toolkit*.

#### Linearly estimated SDOs were used for analysis

In addition to the linear estimation method described above (Eq. 14), we also included and tested a constrained optimization method utilizing the MATLAB *Optimization Toolbox* applied within the *SDO Analysis Toolkit* which currently uses the *fmincon* solver. Here, the SDO is sought as a matrix which minimizes the least mean-squared prediction error of the observed change of the pre-spike distribution, Δ*p*(*x*), given the pre-spike distribution as in Equation 4, while also upholding SDO matrix constraints. However, we found that the SDOs generated by linear estimation had significantly more stable matrix element values than SDOs generated via constrained optimization, measured by element-wise distances between matrices, across different numbers of spikes (250, 500, 1,000, 2,000) within the same stochastic realizations (Table 1). We found this result across all types of simulated data (Table 2). This suggested that the optimization algorithm was potentially overfitting to the data or was following different exploitations of matrix redundancies within a dataset rather than converging onto a single and more generalizable solution. Indeed, prediction errors of the constraint-optimized SDOs were often higher than those derived from the linear-estimated SDOs generated from the same simulated data, indicating that the solver is not identifying the globally optimal SDO.

**Table 1.** Average distances between SDO matrices generated by the direct linear or constrained optimization methods, by number of spikes used

#Spikes	#Spikes	250	500	1,000	2,000	250	500	1,000	2,000
	SDO method	Direct linear				Constrained optimization			
250	Direct linear		−8.21	−8.45	−8.56	−4.18	−4.17	−4.21	−4.29
500				−9.17	−9.37	−4.15	−4.20	−4.22	−4.29
1,000					−10.27	−4.16	−4.19	−4.24	−4.30
2,000						−4.17	−4.19	−4.23	−4.31
250	Constrained optimization						−4.33	−4.43	−4.50
500								−4.68	−4.86
1,000									−5.32
2,000									

The averaged log-normalized summed element-wise distance between SDO matrices estimated using the direct linear equation or constrained optimization methods. Here, for each of the eight stochastic generator models (Y1–Y8), 100 realizations of the stochastic time series and 2,500 spikes were generated. For every realization, for every stochastic process, an SDO was generated using subsets of 250, 500, 1,000, or 2,000 spike training fraction using either the linear estimation equation or via constrained optimization. We tested the similarity of SDO matrices by both the number of spikes used from the same realization (250–2,000) and between methods of SDO estimation. Similarity was calculated as the log-normalized sum of squared differences between matrix elements. (Note that due to this log operation, a more negative value indicates less distance than a less negative value.) To evaluate the consistency of the linear-estimated SDOs and the constrained-optimized SDOs across diverse data, we then averaged the distances of SDO matrices between subsets of data (defined by method and number of spikes). These distances were averaged across all realizations (100) and stochastic generators (Y1–Y8). (That is, 100 realizations × 8 models were averaged together for each measurement.) Linear-estimated SDOs were more like each other than those estimated via constrained optimization across all measured combinations, indicated by a smaller average distance.

**Table 2.** Average distances between SDO matrices generated by the direct linear or constrained optimization methods, by type of modeled data

Data model	Fractions used	Direct linear	Constrained optimization	Δ*x̅*	Δ*x̅* (95% CI)	*p* value
Y1	Pooled	−8.53	−4.26	−4.28	(−4.36, −4.19)	0
Y2		−9.75	−3.90	−5.85	(−5.96, −4.57)	0
Y3		−9.24	−4.40	−4.84	(−4.94, −4.73)	0
Y4		−8.69	−4.27	−4.42	(−4.51, −4.33)	0
Y5		−8.02	−7.22	−0.80	(−0.89, −0.72)	5.10 × 10^−67^
Y6		−10.07	−5.29	−4.78	(−4.91, −4.65)	0
Y7		−9.14	−3.99	−5.15	(−5.27, −5.04)	0
Y8		−8.61	−4.17	−4.44	(−4.54, −4.35)	0
Y1	Adjacent	−8.72	−4.32	−4.40	(−4.53, −4.26)	2.08 × 10^−268^
Y2		−9.95	−4.00	−5.95	(−6.11, −5.78)	2.76 × 10^−293^
Y3		−9.44	−4.53	−4.91	(−5.06, −4.75)	2.38 × 10^−261^
Y4		−8.92	−4.30	−4.61	(−4.75, −4.47)	5.46 × 10^−272^
Y5		−8.22	−7.35	−0.88	(−1.01, −0.74)	4.55 × 10^−32^
Y6		−10.29	−5.46	−4.84	(−5.03, −4.64)	1.19 × 10^−211^
Y7		−9.37	−4.02	−5.35	(−5.53, −5.17)	7.93 × 10^−251^
Y8		−8.83	−4.25	−4.58	(−4.73, −4.43)	9.68 × 10^−257^

The differences in average distances between SDO matrices generated by direct linear equation versus constrained optimization methods, broken down by stochastic generator model (Y1–Y8). Here, for each of the eight stochastic generator models (Y1–Y8), 100 realizations of the stochastic time series and 2,500 spikes were generated. For every realization, for every stochastic process, an SDO was generated using subsets of 250, 500, 1,000, or 2,000 spike training fractions. We then tested the similarity of SDO matrices by both the number of spikes used from the same realization (250–2,000) and between methods of SDO estimation. Similarity was calculated as the log-normalized sum of squared differences between matrix elements. (Note that due to this log operation, a more negative value indicates less distance than a less negative value.) For each type of stochastic generator (Y1–Y8), we tested the average distance between matrices generated using the linear estimation or constrained optimization methods. For this measurement, the average distances by estimation method and generator model (Y1–Y8) were pooled across all spike fractions (i.e., the similarity between all matrices, regardless of number of spikes used to generate them), or only between adjacent training fractions (i.e., distances between matrices generated with 250 and 500 spikes, 500 and 1,000 spikes, 1,000 and 2,000 spikes). We tested the null hypothesis of no difference in the mean distances of SDOs from the linear estimate and constrained optimization methods for all stochastic generator models (i.e., *t* test). The 95% confidence intervals for the differences of means are provided. For all stochastic models, the distances between the linear-estimated SDOs were significantly less than those generated via constrained optimization, indicating that SDO matrix generated via the linear estimation equation was less variable by number of spikes than those generated via constrained optimization, across all modeled data.

We infer that although the four SDO matrix constraints are sufficient to define an SDO, for constraint-optimized estimation there may be additional uncharacterized constraints, preferable optimization techniques, or else much longer datasets than tested here (with concomitantly slower computation) may be necessary to derive a stable and generalizable SDO. We mention the optimization method for completeness in describing the *SDO Analysis Toolkit* and include it as a method of deriving the SDO but caution that significant care will be needed for its use with physiological data. Since the direct linear equation (Eq. 14) seems to be the faster and perhaps better approach in our estimation, we used Equation 14 for deriving and characterizing the SDO throughout results, as described below.

#### Generating spike-independent SDOs for significance testing

Spike-triggered SDOs were tested for significant spike–signal correlations against null hypotheses generated using Monte Carlo sampling. We used the following process: First, we generated 1,000 shuffled-spike trains, for each observed spiking unit (e.g., neuron). We included two methods of shuffling spike times, both included within the *SDO Analysis Toolkit*. The first is to directly shuffle the observed interspike intervals on a trial-wise basis, thus preserving the durations between impulses. The second method is to estimate the rate process underlying the observed spike train, assuming spikes were generated by a renewal process, then to resample this process 1,000 times. (The *SDO Analysis Toolkit* allows for various filtering kernels for this purpose.) In both cases, the 1,000 “shuffled” spike trains from the tested spiking unit were then used to produce 1,000 “shuffled” spike train SDOs, using the same signal time series as used for the tested experimental SDO. Hence, in the null hypotheses we are testing if the changes in signal dynamics measured at a spike are a result of sampling (interspike intervals) or dynamics near a spike, but not directly associated with it (renewal process). The number of shuffles used for significance testing is a tunable parameter within the *SDO Analysis Toolkit*.

#### SDO matrix normalization

**Figure 6.**
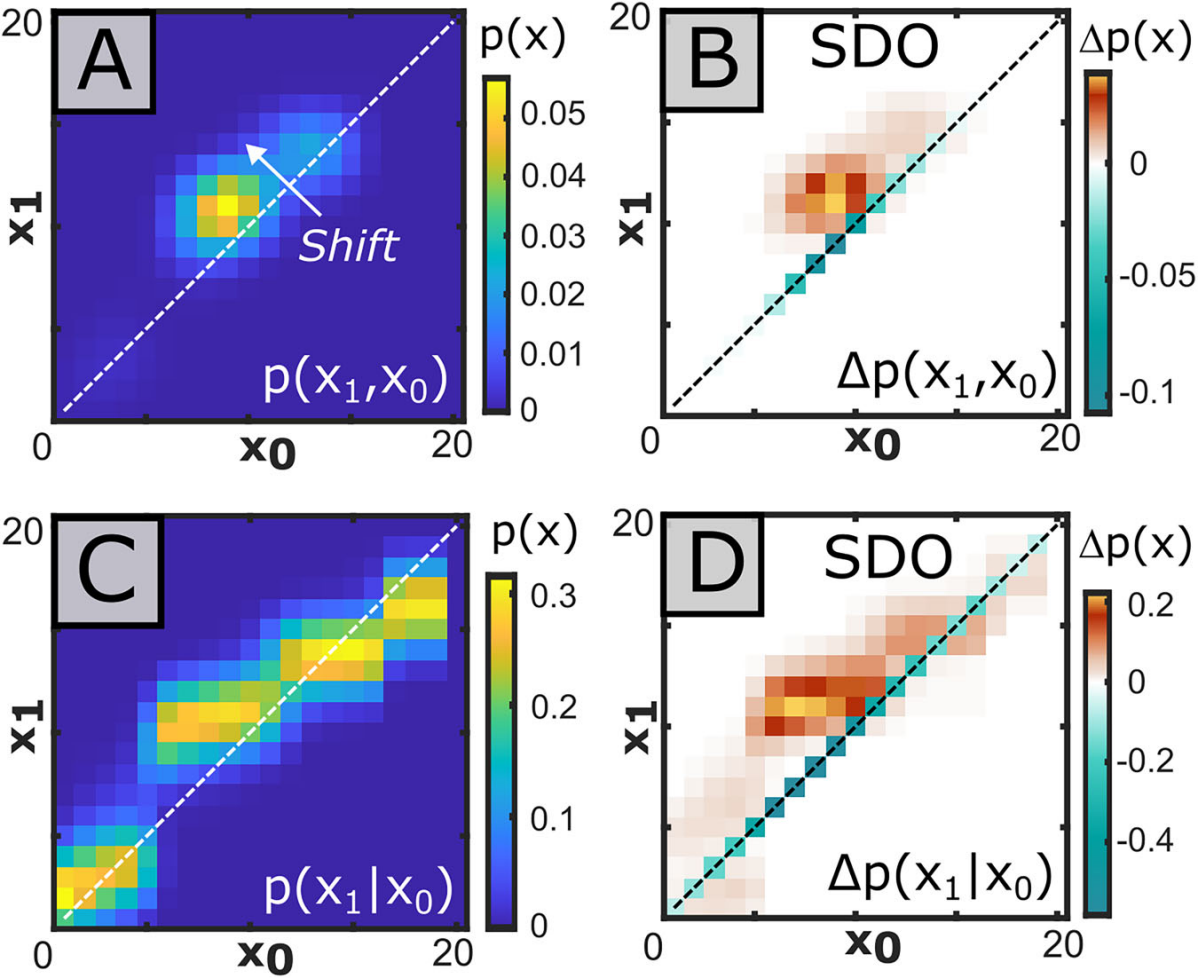
SDOs alter background state transition matrices to compose spike-triggered transition matrices. In this example, pre-spike and post-spike distributions of signal state were generated from the spikes of a single motor unit in the vastus externus against the analog EMG signal recorded in the biceps femoris, using a time interval of 10 ms for both the pre-spike and post-spike distributions. ***A***, The spike-triggered average joint distribution of the pre-spike and post-spike state distributions behaves as the description of state transitions around spike. ***B***, A spike-triggered SDO captures the change of state probability across the 10 ms pre-spike and post-spike distribution of states, given an occurrence of spike. This SDO shows strong directional effects, evidenced by the asymmetrical banding relative to the diagonal. ***C***, Normalization of each column of the joint distribution to 1 creates a left transition matrix, representing the expected probability of post-spike state distribution given a pre-spike state distribution. ***D***, Normalizing the columns of the SDO by the same factor used in the joint distribution (***C***) creates the normalized SDO. This form is used to predict the conditional change of state distributions.

The joint probability of the pre-spike and post-spike state distributions describes the overall probability of observing state transitions in the pre-spike and post-spike intervals (Fig. 6*A*). The column sum of the joint distribution provides *P*(*x*_0_), the average pre-spike state distribution from the ensemble, and the row sum of the joint distribution provides *P*(*x*_1_), the average post-spike state distribution from the ensemble. As estimated in Equation 4, the SDO matrix (Fig. 6*B*) describes the change in state probability distributions across the ensemble and will reflect the probability distributions used to construct it. Specifically, $\Delta p(x_{1}=i,\;x_{0}=j)$ depends on both the conditional change in probability given an initial state $\Delta p(x_{1}=i|\;x_{0}=j)$ and the probability of observing the initial state, $p(x_{0}=j)$, in the pre-spike distribution. Both the value of Δp(x) and confidence in this value are affected by the probability of observing it in the data. This latter quantity may differ between the spike-triggered (method H7), spike-shuffled (null), and background (all time points, method H4) SDOs.

To account for differences in the sampling of pre-spike state (column scaling) between the spike-triggered, background, and “spike-shuffled” SDOs, the columns of the SDO matrices are first normalized to the conditional form, $\left(\Delta p(x_{1}|\;x_{0})\right)$. When normalizing an SDO matrix, each column *j* of the SDO is scaled by the reciprocal of the probability of state *j* in the pre-spike distribution, $\frac{1}{\;p(x_{0}=j|s)}$. [When applied to the joint distribution of state $p(x_{1}|x_{0})$, this is equivalent to normalizing each column to 1 (Fig. 6*C*), producing a transition matrix.] In this normalized form, the SDO represents the conditional change of the probability of state, given an initial state distribution, $\Delta p(x_{0}|x_{0})$, and hence emphasizes magnitude of correlated signal change, by state (Fig. 6*D*). The normalized (conditional) SDO matrix is the form that is used for generating predictions of Δ*p*(*x*) as in Equation 2 for all H1–H7. However, it is not ideal to directly compare between normalized SDOs or use these to estimate significance here because this form may distort the contributions of very rarely sampled states to the measures of significance.

Instead of normalization, for comparison and testing significance, the conditional SDOs of the spike-triggered (method H7), “shuffled”, and background (method H4) SDOs are rescaled by the average pre-spike state distribution of the observed spike-triggered ensemble, *P*(*x*_0_), simulating what the “shuffled” and background SDOs would resemble if they had been taken from the same pre-spike state distributions as the spike-triggered SDO (method H7). [Note that this process produces the same method H7 spike-triggered SDO as originally sampled (Fig. 6*B*).] These rescaled SDOs can then be used for evaluating the matrix similarity-based measures of significance (as mentioned above). Both the normalized and rescaled SDOs can be readily plotted and examined within the *SDO Analysis Toolkit*.

#### Identifying significant SDOs

We first identified significant SDOs by looking for differences in the spike-triggered (method H7) and spike-shuffled SDO matrices. [Differences between spike-triggered SDOs (method H7) and background SDOs (method H4) were also measured but were not used to directly determine significance of spike effects]. In the rescaled spike-triggered and shuffled-spike SDOs, we measured four features of SDO effect and one feature of state tuning:

1. Matrix element values: The sum of squares error (SSE) calculated between each element of the spike-triggered SDO matrix versus the distribution of values for that element in the shuffled distribution.
2. Matrix similarity: Overall cumulative SSE of the spike-triggered SDO matrix and the shuffled SDO matrices or between the respective joint distributions.
3. State-wise post-spike distribution directional shift/bias: For each input state (SDO column), the sum of the elements above the matrix diagonal versus the elements below the matrix diagonal. (This corresponds to the expected change in mean imposed by the SDO, conditional on input state.)
4. Array-wise total post-spike distribution directional shift/bias: The sum of all SDO matrix elements above the diagonal versus the elements below the diagonal (i.e., does the spike coarsely facilitate or inhibit signal?).
5. Pre-spike state tuning: The Kullback–Leibler divergence (KLD) between the observed state at spike, p(x|s), versus shuffled-spike times. (Note that this is not a measure of the spike effect but can be used to identify significant relationships between signal level and a spike occurrence.)

The significance of the spike-triggered SDO (method H7) was determined by comparing the spike-triggered test statistic versus the null distribution of test statistics from the 1,000 shuffled-spike SDOs. Here, the difference between the spike-triggered SDO statistic and the mean of the shuffled-spike SDO statistics was compared with the internal variance of the population of 1,000 “shuffled” SDOs (i.e., is the test result from the spike-triggered SDO significantly different than would be predicted from the shuffled distribution?) A *p* value of <0.05, with Bonferroni’s correction for the number of states (here, 20), was used to determine significance. Within the *SDO Analysis Toolkit*, the number of shuffles and the alpha value for significance testing can be readily modified.

#### Visualization of SDO and SDO effect predictions

Visualization of the correlation between spikes and signal is important for intuition. The spike impulse response of the STA may be readily visualized and interpreted from the average time-varying waveform of sampled signal amplitude across all spiking events. In contrast, the SDO describes the change in state probability distributions without prescribing a specific temporal waveform structure. Thus, any time effects would not be immediately apparent from the pre-spike and post-spike distributions for the SDO. Nonetheless, considering the underlying state transition probability and signal behavior over time is important.

**Figure 7.**
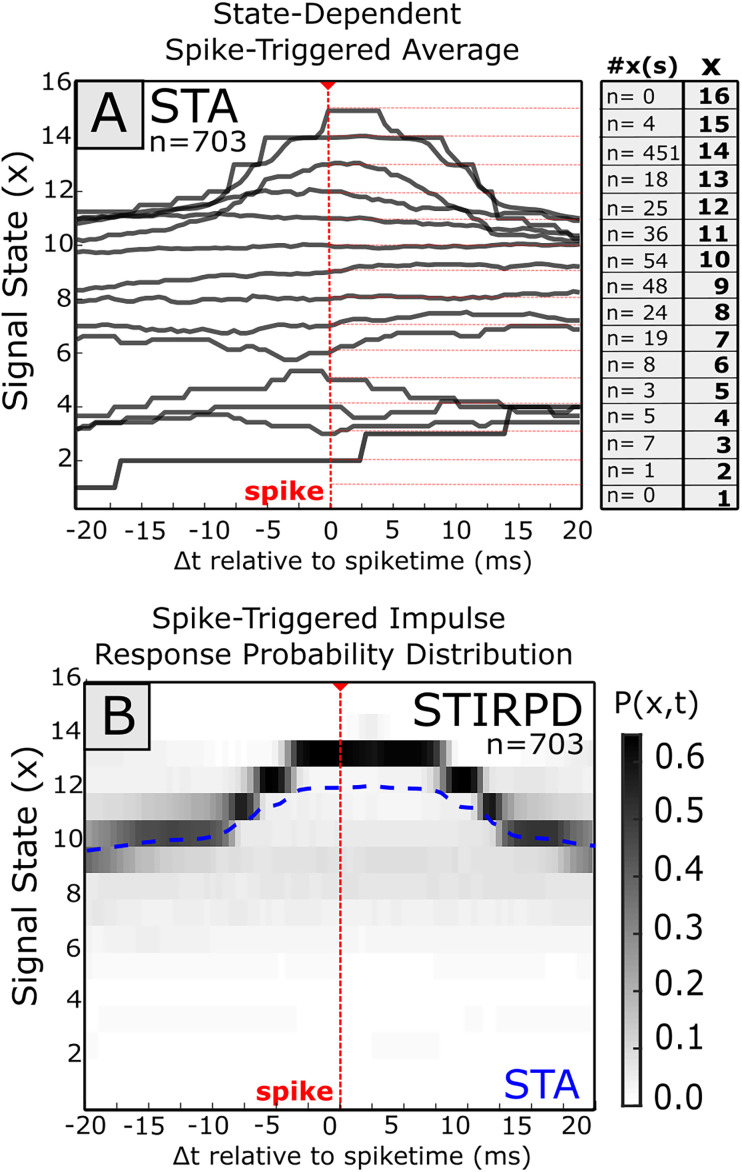
Expressing STAs and probability distributions. The spike-triggered impulse response probability distribution (STIRPD) is a combination of the classical spike-triggered average and state probability distributions. ***A***, The relationship between the spike train of an interneuron is compared against EMG (filtered acausally). In the state-dependent STA, a set interval [*s *− Δ*t*, *s *+ Δ*t*] of signal behavior is extracted for every spiking event (*s*), sampled in discrete time. A state, *x*, is assigned to every observed time point, *t*, (t∈[s−Δt,sΔt]) within this interval to provide a sequence of states, x(t). When segregating the STA by state at time of spike, x(s), state-dependent behavior may be observed. ***B***, The spike-triggered impulse response probability distribution extends the STA by calculating the distribution of states at each measured time point p(x,t) across all spiking events. Because both states and time are discrete, the STIRPD is a finite matrix and may be displayed as an image. Here, the “arching” signal behavior present at the higher states in the STA is present as a dark band, but the STIRPD nonetheless captures the relatively invariant signal behavior in states 6–10 in the perispike interval. Horizontal separation of state mass in the STIRPD (i.e., banding) will result in multimodal pre-spike or post-spike distributions elements of the joint probability matrix and may be indicative of cases where the STA is insufficient. Here, the STA is overlaid on the STIRPD in blue.

We observed that the spike-triggered response may vary by signal state at time of a spike (Fig. 7*A*). To visualize and identify such variations of signal behavior relative to spiking events in the SDO framework, we developed the spike-triggered impulse response probability distribution (STIRPD) description (Fig. 7*B*). The STIRPD is effectively a transformation of the classical STA into the probability state space but has the advantage of more robustly describing the relationship between the spiking source and signal behavior in time. To calculate the STIRPD, as with the STA, for each spiking event, *s*, the state-quantized signal is sampled on an interval around spike from (s−Δt,s+Δt). Subsequently, the distribution of signal states at every time point *t*, *p*(*x*,*t*), is calculated from the same relative time bin across all sampled signals. The value of each element represents the probability of observing a given state (as opposed to another state) at a given time after a spike, $\mathrm{STIRP}D_{ij}=p(x=i|t=j)$.

Because both time and state are defined discretely, the STIRPD may be displayed as a raster image and interpreted qualitatively. The familiar spike-triggered average (in state space: mean state) is laid over the probability distribution. The probability distribution of state at time of a spike, *p*(*x|s*), is captured as a single column vector for the time bin containing the spike on the STIRPD and thus can reveal if the distribution is expected to be unimodal or multimodal or if there is a coarse signal behavior change around the spike. The summation of state probability over time in the STIRPD during the pre-spike interval, [s−Δt,s] provides $p(x_{0})$, while summation of the STIRPD over a given post-spike interval provides $p(x_{1})$, used in the calculation of a specific SDO. The STIRPD description thus links the SDO and STA visualization methods.

**Figure 8.**
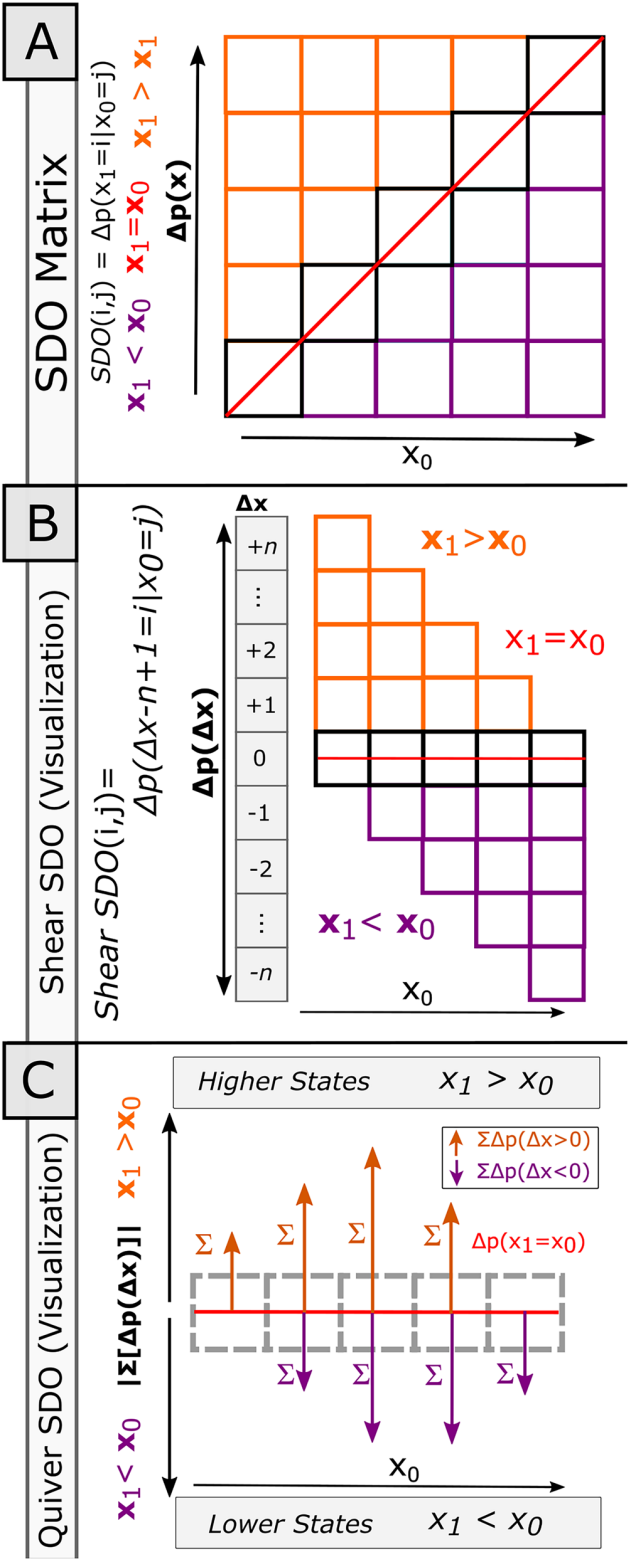
Alternative visualizations of the SDO matrix. The effect of a spike-triggered SDO on the probability of signal state between two observations *x*_0_ and *x*_1_ may be qualitatively inferred from the SDO directly. ***A***, The SDO matrix may be divided into three regions, the primary diagonal (red line), representing the probability of maintaining state; the “above-diagonal” (orange) region, representing the probability of increasing state; and the “below-diagonal” (purple) region, representing the probability of decreasing state. ***B***, The square SDO matrix may be sheared to orient the prior matrix diagonal to the horizontal. The shear SDO visualization emphasizes the change in probability of state, Δ*p*(*x*), relative to the directional change of state, Δ*x*. As SDO operators are sparse matrices and usually concentrated around the diagonal when time intervals are short, the shear SDO may be trimmed to a few rows to compactly display the effects of an SDO. ***C***, The magnitude and directional effects of an SDO can be coarsely described by summing Δ*p*(*x*_1_|*x*_0_) separately for the regions above (orange) and below (purple) the SDO matrix diagonal. In the quiver SDO visualization, these column sums are represented as vertical vectors oriented above or below the abscissa, respectively. The length of each vector for state *x* is the magnitude of the summed change in probability. The vector heading projecting from the abscissa corresponds to whether the change of state, Δ*x*, is positive (orange) or negative (purple). The elements of the diagonal are plotted as a line within the same axes. The orientation and magnitude of vectors for each state, *x*, provide a coarse measure of influence of Δ*p*(*x*_1_|*x*_0_).

As with the STIRPD, we could also qualitatively infer the relationship between a target signal and the spike source neuron for the pre-state (*x*_0_) to the post-spike (*x*_1_) state change using visualizations of the SDO matrix directly (Fig. 8*A*). When inspecting the SDO, we also found it is intuitively helpful to plot a shear of the SDO matrix such that the prior matrix diagonal, corresponding to (i=j), is aligned horizontally (Fig. 8*B*). This transformation emphasizes the direction of the SDO effects, Δp(Δx,t): Elements above the shear SDO horizontal correspond to transitions toward higher states $\left(x_{1}>x_{0}\right)$, while below-diagonal corresponds to transition toward lower states $\left(x_{1}<x_{0}\right)$. The sign and magnitude of these elements may be used to interpret SDO effect for a given input state. [For example, for an input state which has positive elements (increased probability) above the shear-horizontal “toward higher states” and negative elements (decreased probability) below the shear-horizontal “toward lower states,” the net effect for that input state is a shift toward the higher states].

A third visualization of the SDO we explored is the “Quiver” representation of an SDO (Fig. 8*C*), which emphasizes the magnitude and coarse directional biases of Δp(x) for a given input state. Here, in the quiver representation, each column of the shear SDO matrix is summed over (1) the above-horizontal elements and (2) the below-horizontal elements, and each represented by a vector whose magnitude is proportional to this sum, originating at the abscissa, and projecting up and down, respectively. The elements of the reference horizontal row (the original SDO matrix diagonal), corresponding to $\Delta p(x_{1}=x_{0})$, are coplotted by a simple line (because the SDO matrix diagonal is defined to be nonpositive, this trace is negative). These vectors indicate the coarse directional effect of the SDO for a given pre-spike state: If both vectors above and below have an equivalent magnitude for a state, then the change in post-spike state overall is not directional (i.e., there is no change in the mean of the post-spike state distribution contributed by this input state), while unbalanced vectors indicate a coarse direction of change in the post-spike predicted state distribution.

#### Assessing the prediction utility of significant SDOs

Finally, in comparing methods, we tested the capability of the SDO (method H7) to predict post-spike signal behavior within our datasets. We assessed two types of prediction: (1) predictions of the post-spike state *distributions*, $\hat{\;p}(x_{1})$, and (2) predictions of most likely single state within the same post-spike interval, ${\hat{x}}_{1}=\mathrm{argma}x_{x_{i}}:\hat{\;p}(x_{1}=x_{i})$. Predictions of the post-spike state distribution (1) capture overall signal dynamics, while predictions of single post-spike states (2) reflect utilization of such a prediction toward directing an exclusive action or decision (e.g., “go”/”no-go”, tracking-target, etc.). (Note that even in instances where the spike-triggered SDOs did not significantly differ from shuffled-spike or background, SDOs could still be used to predict signal behavior, but we have limited our scope here to significant cases to compare the STA vs the SDO directly.)

When evaluating predictions of an SDO (i.e., the correlation between a particular spiking unit and signal), the observed pre-spike and post-spike distributions of signal state were extracted for each spiking event. The predicted post-spike state distribution, $\hat{\;p}(x_{1}|H)$, were generated on an event-wise basis for each of the seven matrix model hypotheses (methods H1–H7) described above (including the spike-driven SDO as method H7), in accordance with Equation 14. The “single best state” of each predicted post-spike distribution was defined as the most likely state (i.e., peak). When evaluating model-predicted post-spike distributions, the experimentally observed post-spike distribution, $p(x_{1})$, served as ground truth. Hypotheses which minimized the reconstruction error of the post-spike distributions with the ground truth model were considered the model of best fit ( $\mathrm{argmi}n_{H}:p(x_{1})-\hat{\;p}(x_{1}|H)$ for H1–H7). (Note that here, the H3 matrix is used to predict the effects of the classical STA represented in the probability space, corresponding to the average change in signal distributions which would otherwise be predicted from the time-varying amplitude.) Spiking events, and associated errors, were treated as independent observations. The difference between predicted and observed distributions was quantified using the KLD and log-likelihood. The difference between predicted and observed single states was quantified using the error frequency (*e*0), absolute error (*e*1), and squared (*e*2) error. Because the spike-wise error rate for these predictions to single states are integer values, and the population of errors is not expected to resemble a continuous distribution (and hence may be ill-suited for tests of means or medians), we instead calculate the cumulative error and bootstrap this statistic to evaluate the confidence intervals. If the distribution of two model hypothesis errors did not overlap at the 95% confidence interval, they were considered significantly different. Observed error rates were considered significantly different if there was no overlap of the estimated 95% confidence intervals, corresponding to a *p* value of <0.05. We used Cohen's *D* statistic (Cohen, 1988) to measure the effect size between bootstrapped cumulative error distributions. We hypothesized and expected that, by capturing information of the spike effect more effectively, the spike-triggered SDO (H7) predictions would be the best most of the time.

## Results

### SDO methods were more sensitive to state-dependent relationships than the STA in simulated data

We compared the statistical sensitivity and specificity of SDO (method H7) and classical (i.e., time domain) STA methods by comparing false-positive and false-negative rates for detecting spike effects in model simulated data. Within the model simulated data, the white noise (Generator Y1), background dynamics (Generator Y4), Markov (Generator Y5), and ARIMA (Generator Y8) stochastic processes contained no spike-triggered effects. We would thus consider the detected significance of spike-triggered tests applied against these processes as false positives. Similarly, when spiking events induced a change in signal amplitude or dynamics (Generators Y3, Y4, Y6, Y7), we considered the failure to detect significance as false negatives. Significance tests of the STA and SDO had different performance by condition (Table 3). Three STA methods were compared. The STA *t* test method reported Generator Y2 (random spike-driven impulses) as significant 100% of the time, while both the ISA subtraction and Chronux-based STA methods never reported Generator Y2 as significant (the desirability of this outcome for physiological analysis may vary by circumstance). Only SDO measures could reliability detect significance of Generator Y7 spike effects, while STA did not.

**Table 3.** Performance of the different significance tests of spike-triggered effects for the STA and SDO methods over 1,600 simulations

Basis	Test	Data generator model type								Average (Y1–Y8)
		Y1	Y2	Y3	Y4	Y5	Y6	Y7	Y8	
STA	Simple *t* test	0.95	1	1	0.95	0.94	1	0.09	0.96	0.86
	ISA-subtracted	0.75	0	1	0.81	0.88	1	0.33	0.83	0.7
	Chronux	1	0	1	0.99	1	1	0.07	1	0.76
SDO	Element similarity	1	0.9	0.86	1	1	0.89	0.38	1	0.88
	Matrix similarity	0.99	0.9	1	0.98	1	1	0.9	1	0.97
	*P*(*x*_0_,*x*_1_) similarity	0.99	0.9	0.91	0.89	0.9	1	1	0.9	0.94
	Total coarse bias	0.97	0.8	0.88	0.97	1	0.9	0.89	0.99	0.92

Test performance was evaluated as the proportion of “correct” tests to the total number of tests, by method and condition. Here, Y1 (white noise), Y4 (background dynamics), Y5 (Markov process), and Y8 (ARIMA) were not influenced by spike; hence failure to reject the null hypothesis (i.e., no spike effect) was treated as “correct”. For Y2 (spike-triggered random impulses), Y3 (spike-triggered consistent impulses), Y6 (spike-triggered consistent impulses on a dynamic background), and Y7 (spike-triggered dynamics), a significant spike-triggered effect was treated as “correct.” The average performance is the average value across each row. Here, SDO methods are highly sensitive and specific across all tested data modalities. Tests of the entire SDO matrix (“Matrix Similarity”) or the joint distributions of pre/post-spike state (“*P*(*x*_0_,*x*_1_) Similarity”) had the highest accuracy.

We assessed the relative sensitivity (i.e., the probability that a “True” effect is classified as “True”) and specificity (i.e., the probability that a “False” effect is classified as “False”) of the STA and SDO methods in the simulated data. We again assessed the significance of the STA and SDO by combining the results of tests across all measures: i.e., an STA or SDO was considered “significant” if any of the tests of significance of spike effect returned significance (as in an initial assay for significant relationships in experimental data). When describing simple relationships between spike and signal (e.g., Generator Y3; noise convolved with consistent impulse responses), STA and SDO methods were equally capable of identifying consistent state-independent spike-triggered responses (Generators Y3, Y6) throughout simulated data. This sensitivity was upheld even when spike effects represented random impulses (Generator Y2). The classical STA method had a higher false-positive rate for time series data generated from a Markov generator process (Generator Y5) than the SDO method. However, in the case where spiking events caused a change in local system dynamics (Generator Y7), the STA was significantly less sensitive than the SDO method (H7), detected as 39.3 ± 4.9% [STA]^a^ versus 99.8 ± 0.4% [SDO]^b^ (one-tailed *t* test, *p* < 0.00001).

We also observed a difference in specificity with the STA and SDO significance measures depending on the origin of stochastic signals across models (Generators Y1–Y8). When evaluating the grand mean rates across all conditions, the STA produced a significantly higher number of false-positive tests in simulation for white noise signals (Y1), 27.7 ± 3.1% [STA]^c^ versus 15.4 ± 6.8% [SDO]^d^ (one-tailed *t* test, *p* < 0.00001), signals with only background dynamics (Y4), 22.3 ± 3.3% [STA]^e^ versus 17.9 ± 8.4% [SDO]^f^ (one-tailed *t* test, *p* = 0.029), and Markov processes (Y5), 17.1 ± 5.6% [STA]^g^ versus 2.7 ± 3.4% [SDO]^h^ (one-tailed *t* test, *p* < 0.00001). The false-positive rate between the STA and SDO methods for the ARIMA(3,2) process (Y8) did not significantly differ 19.1 ± 4.4% [STA]^i^ versus 15.4 ± 9.8% [SDO]^j^ (one-tailed *t* test, *p* = 0.12).

We next tested the importance of the number of spikes versus the number of bootstrapped “shuffles” used for SDO significance estimation. (Note that in this case, the number of shuffles, used for evaluating SDO significance, is expected to have no effect on the STA.) For each simulation, eight stochastic signals (Generators Y1–Y8) and the associated common spike train were generated, as described above. One hundred simulations were performed for every combination of an analyzed number of spikes (250, 500, 1,000, 2,000) and number of shuffles (250, 500, 1,000, 2,000). For these simulations, other variables (e.g., filtering, number of states, length of pre-spike/post-spike intervals, signal transformations, amplitude/shape of the spike-triggered impulse response) were kept consistent. The battery of SDO significance measures remained highly sensitive with all tested parameters (Table 4) and, unsurprisingly, became more specific as additional bootstraps were used for estimating the distribution of the null statistic for SDO (Table 5). As expected, measures of the STA specificity did not vary by number of shuffles used for SDO significance estimation (Table 6). For all combinations, except 250 spikes with 250 bootstraps, SDO methods outperformed STA measures of significance (Table 7). Having observed that the SDO could provide additional sensitivity and specificity compared with the STA in simulated data, we next sought to test the predictive capacities of the SDO in simulated data.

**Table 4.** Sensitivity of SDO significance tests by number of shuffles and spikes

SDO	#Spikes				
#Shuffles		250	500	1,000	2,000
	2,000	1	1	1	1
	1,000	1	1	1	1
	500	1	1	1	1
	250	1	1	1	1

Sensitivity of SDO significance tests by number of shuffles and spikes. Proportion of 100 simulations (per condition) where any of the four SDO significant tests reported a significant spike–signal relationship, out of all simulations with a true spike–signal relationship (Y2, Y3, Y6, Y7), by number of spikes and bootstraps (i.e., true positive rate). SDO significance methods were highly sensitive. Here, the number of spikes and bootstraps did not affect the sensitivity of the SDO significance measurements across the simulated data.

**Table 5.** Specificity of SDO significance tests by number of shuffles and spike

SDO	#Spikes				
#Shuffles		250	500	1,000	2,000
	2,000	0.08	0.1	0.08	0.05
	1,000	0.14	0.11	0.07	0.06
	500	0.15	0.18	0.05	0.11
	250	0.26	0.19	0.14	0.18

The proportion of false-positive rates for 100 simulations per combination of spike number and shuffle. We considered false positives as simulations where any of the four SDO significance tests reported a significant relationship for stochastic models where the spike had no effect (Y1, Y4, Y5, Y8). Here, unsurprisingly, the specificity of the SDO methods increase with a higher number of shuffles used for generating the null distribution for significance tests.

**Table 6.** Sensitivity of STA significance tests by number of shuffles and spike

STA	#Spikes				
#Shuffles		250	500	1,000	2,000
	2,000	0.84	0.86	0.84	0.84
	1,000	0.83	0.82	0.85	0.87
	500	0.85	0.86	0.85	0.85
	250	0.86	0.85	0.85	0.85

The proportion of 100 simulations (per condition) where any of the three STA significant tests reported a significant spike–signal relationship, out of all simulations with a true spike–signal relationship (Y2, Y3, Y6, Y7). Here, only the number of spikes vary in the STA significance calculations (shuffles counts are only used for coestimated SDO significance). In this instance, the sensitivity of STA significance methods does not vary by spike count.

**Table 7.** Specificity of STA significance tests by numbers of shuffles and spikes

STA	#Spikes				
#Shuffles		250	500	1,000	2,000
	2,000	0.18	0.21	0.15	0.21
	1,000	0.23	0.21	0.22	0.22
	500	0.23	0.22	0.22	0.22
	250	0.22	0.21	0.23	0.22

Proportion of false-positive rates for 100 simulations per combination of spike number and shuffle. We considered false positives as simulations where any of the three STA significance tests reported a significant spike–signal relationship, out of all stochastic models where spike had no effect (Y1, Y4, Y5, Y8). Here, the only the number of spikes vary in the STA significance calculations (shuffles counts are only used for coestimated SDO significance). Specificity of the STA significance methods are more variable than sensitivity across simulation. STA significance specificity is lower than for the SDO.

### The spike-triggered SDO is generally more predictive of post-spike state than other matrices

To evaluate the performance of the SDO matrices estimated from the linear equation (Eq. 14) versus other hypothesized model estimation methods, including the constrained optimization, we generated 100 simulations (i.e., pairs of neuron and time series) of 2,500 spikes, for each of the eight stochastic generator simulated models (Y1–Y8). For each simulation, we estimated an SDO with 1,000 spikes and tested the prediction performance on the remaining subset of 1,500 spikes. We evaluated the average spike-wise prediction error for both post-spike state (absolute error, *e*_1_) and post-spike state distributions (KLD) across the range of model estimation methods (H1–H7), including the SDO as method H7. Generally, the stochastic process generators (Y1–Y8) did not directly correspond to the estimation method (H1–7) in terms of best prediction, especially when spike-driven dynamics were involved in the simulated data.

For predictions of post-spike state, the spike-triggered SDO (method H7) provided the lowest absolute error or did not significantly differ from the best fit method (Table 8). The linear-estimated SDO also had lower absolute error than the constrained-optimized SDO for all test data except the Markov model generator (Y5). However, for measures of predicted post-spike state distribution (KLD), the results were mixed. Of the two methods of estimating the SDO (H7), the linearly estimated SDO provided better predictions for model-generated data with spike-driven effects (Y2, Y3, Y6, Y7) while the constrained optimization had lower prediction error for models without spike effects (here, Generators Y1, Y5, and Y8; Table 9). Together, the results of the absolute error and KLD error indicate that the spike-triggered SDO, as estimated within the simulated data, can usually predict the peak of the post-spike state distribution on a spike-wise basis better than other models but may overestimate the variance when operating over the intervals tested here, where dynamics may be collapsed together. This may be due to limitations of smoothness of individual spike-triggered pre-spike and post-spike distributions: SDOs were originally formulated for fundamentally smooth probability distributions. As these distributions were averaged into the SDO, the SDO and its post-spike predictions become smooth. The coarser post-spike distributions realized in finite discrete data may thus be difficult to replicate with smooth SDOs and the SDO method result in a higher KLD accordingly. Moreover, we found that while Gaussian smoothing pre-spike and post-spike distributions reduced the KLD between predicted and observed post-spike state distributions, it did not change the matrix hypothesis method (H1–H7) of best fit.[Table T10]

**Table 8.** Prediction errors of matrix method hypotheses when predicting post-spike state in simulated data

Matrix fit method		Data generator model								Mean (Y1–Y8)
		Y1	Y2 (S)	Y3 (S)	Y4	Y5	Y6 (S)	Y7 (S)	Y8	
H1	No change	1.997^a,b^	0.977^a,b^	1.298^a,b^	2.751^a,b^	0.408	0.985^a,b^	1.297^a,b^	1.223^a^	1.367
H2	Gaussian	1.932^a,b^	0.967^a,b^	1.266^a,b^	2.647^a,b^	0.409	0.977^a,b^	1.215^a,b^	1.185^b^	1.325
H3	STA	1.854^a^	0.879^b^	1.034^a,b^	1.990^b^	7.252^a,b^	0.677^b^	0.521^b^	1.683^a,b^	1.986
H4	Background SDO	1.706^b^	0.834^b^	0.975^b^	1.980^b^	0.432	0.647	0.637^a^	1.158^b^	1.046
H5	Markov matrix	1.772^a^	0.850^b^	1.518^a,b^	2.299^a,b^	0.423	0.781^a,b^	0.708^a,b^	1.122^a,b^	1.184
H6	Background + STA	1.706^b^	0.834^b^	0.971^b^	1.980^b^	0.432	0.639^a,b^	0.637^a^	1.580^b^	1.097
H7^a^	Direct linear SDO	1.705^b^	0.839^b^	0.970^b^	1.990^b^	0.434	0.663^b^	0.504^b^	1.161^b^	1.033
H7^b^	Constrained Optim. SDO	1.875^a^	1.034^a^	1.153^a^	2.130^a^	0.410	0.713^a^	0.645^a^	1.223^a^	1.148
	Mean (H1–H7)	1.818	0.902	1.148	2.221	1.275	0.760	0.771	1.292	

Magnitude of error between predicted and observed most likely post-spike state by stochastic model, by matrix hypothesis. Using 100 simulations of 2,500 spikes for each stochastic model (Y1–Y8), a matrix hypothesis was generated using 1,000 spikes. H1–H7 matrices, including both the direct linear and constrained optimization SDO estimations, were then used to predict post-spike state on the remaining data. The grand mean of prediction error (averaged over spikes and simulations) by condition (Y# and H#) is provided. An ANOVA was performed on each stochastic model (Y1–Y8), comparing the mean error by hypothesis (H1–H7). For all stochastic generators (Y1–Y8), there were significant differences in mean prediction error *p* < 0.00001. Post hoc comparisons were performed using Dunnett's test. For every stochastic generator (Y1–Y8), significant differences between the linearly estimated SDO and other models are given by ^a^ and significant differences between the constrained SDO and other models are given by ^b^ Generator models which contained spike-driven effects are indicated with (s). Here, the linear-estimated SDO produced the lowest error in predicting post-spike state across all simulated data.

**Table 9.** Prediction errors of matrix method hypotheses when predicting post-spike distributions in simulated data

Matrix fit method		Data generator model								Mean (Y1–Y8)
		Y1	Y2 (S)	Y3 (S)	Y4	Y5	Y6 (S)	Y7 (S)	Y8	
H1	No change	7.682^b^	3.221^a,b^	6.524^a,b^	7.846^a,b^	2.851^a,b^	6.408^a,b^	6.955^a,b^	5.399^a,b^	5.861
H2	Gaussian noise	7.565^a^	3.002^a,b^	5.907^b^	7.518^a,b^	5.908^a,b^	5.947^a,b^	7.330^a,b^	5.829^a,b^	6.126
H3	STA	7.794^a,b^	3.359^a,b^	5.747^a,b^	6.582^a,b^	10.965^a,b^	5.729^a,b^	3.394^a,b^	7.873^a,b^	6.430
H4	Background SDO	7.658	2.973^a,b^	5.714^a,b^	6.794^b^	7.892^b^	5.381^a,b^	6.895^a,b^	6.960^b^	6.283
H5	Markov matrix	7.211^a,b^	2.735^a,b^	6.282^a,b^	6.856^a^	6.418^a,b^	5.709^b^	4.789^a,b^	6.309^a^	5.789
H6	Background + STA	7.658	2.973^a,b^	5.754^a,b^	6.794^b^	7.892^b^	5.393^a,b^	6.895^a,b^	6.96^b^	6.290
H7^a^	Direct linear SDO	7.655	3.883	5.988^b^	6.795^b^	7.896^b^	5.701^b^	5.815	6.957^b^	6.336
H7^b^	Constrained Optim. SDO	7.616	3.998	6.158^a^	6.891^a^	3.835^a^	5.805^a^	5.862	6.268^a^	5.804
	Mean (H1–H7)	7.605	3.268	6.009	7.010	6.707	5.759	5.992	6.569	

Kullback–Leibler divergence between predicted and observed post-spike state distribution by stochastic model, by matrix hypothesis. For 100 simulation of 2,500 spikes for each stochastic model (Y1–Y8), a matrix hypothesis was generated using 1,000 spikes. H1–H7 matrices were then used to predict post-spike state distributions of the remaining data. The grand mean of prediction error by condition is provided. An ANOVA was performed on each stochastic model (Y1–Y8), comparing the mean error by hypothesis (H1–H8). For all stochastic generators (Y1–Y8), there were significant differences in mean prediction error *p* < 0.00001. Post hoc comparisons were performed using Dunnett's test. For every stochastic generator (Y1–Y8), significant differences between the linearly estimated SDO and other models are given by ^a^ and significant differences between the constrained SDO and other models are given by ^b^ Generator models which contained spike-driven effects are indicated with (s).

**Table 10. T10:** Statistical table

	Data structure	Type of test	Confidence intervals
a	Normal	*t* test (1-tailed)	39.31 ± 2.425
b	Normal	*t* test (1-tailed)	99.81 ± 0.198
c	Normal	*t* test (1-tailed)	27.69 ± 1.517
d	Normal	*t* test (1-tailed)	15.00 ± 3.307
e	Normal	*t* test (1-tailed)	22.31 ± 1.637
f	Normal	*t* test (1-tailed)	17.88 ± 4.111
g	Normal	*t* test (1-tailed)	17.06 ± 2.722
h	Normal	*t* test (1-tailed)	2.69 ± 1.635
i	Normal	*t* test (1-tailed)	19.09 ± 2.622
j	Normal	*t* test (1-tailed)	15.36 ± 5.780
k	Bootstrapped normal	*t* test (1-tailed)	357 ± 0.810
l	Bootstrapped normal	*t* test (1-tailed)	287.9 ± 0.775
m	Bootstrapped normal	*t* test (1-tailed)	1,816.8 ± 5.863
n	Bootstrapped normal	*t* test (1-tailed)	487.5 ± 1.964
o	Bootstrapped normal	*t* test (1-tailed)	534.8 ± 2.003
p	Bootstrapped normal	*t* test (1-tailed)	167.9 ± 0.781

Types of tests and measures of confidence intervals used. The row label corresponds to the superscript of the matching test in text and Tables.

Rather, this relative underperformance of the spike-triggered SDO on KLD metrics may represent a structural issue in our test design. While the SDO captures the variance of the average post-spike distribution over all spikes via Equation 2, unlike other matrices, it does not necessarily predict the signal variance borne out of single stochastic realizations of state signal around a spike as tested here. Rather, the predicted post-spike state probabilities, given the same pre-spike state distribution, reflect the expected (i.e., average) post-spike distribution across the post-spike intervals from all spiking events from the same prior distribution. At the same time, the maximum of the post-spike distribution (post-spike “state”) is likely more consistent across realizations. Matrix hypotheses with no change in variance (H1), a consistent change in variance (H2), convergence to a distribution with a set variance (H3), or having variance tied to the rate of transitions between stochastic states (H5), may thus sometimes outperform the spike-triggered SDO (H7) when predicting distributions from single realizations or coarse distributions. Finer grained temporal analyses may overcome these issues. Having identified that SDO methods are more sensitive than the STA, and can be more predictive of post-spike state, and sometimes post-spike distributions in simulated data, we then set about applying SDO methods to physiological data.

### Identifying when SDOs are needed instead of STAs in physiological data

EMG signals (2,000 Hz) were processed off-line using a series of 60 Hz notch (first eight harmonics), 10 Hz high-pass, and 20-point rectified root mean square zero-phase filters. Because the distribution of EMG amplitude in our trials was roughly exponential, we log-transformed the processed EMG before assigning the processed signal to states. The resultant assigned and quantized “state signal” varied smoothly and “continuously” (i.e., subsequent discrete states effectively only transitioned to adjacent states when using a 0.5 ms time step), although this was not essential for any of the development and testing here. Under this definition of signal states, we observed signal behavior of the spike-triggered averaged signal waveform (in state space), often varied by signal state at time of spike (Fig. 6; i.e., demonstrated state dependency).

Similarly, and unsurprisingly, the occurrence of spikes was not homogeneous by signal state, as measured by the probability of state at spike, p(x|s). The distribution of state at spike varied with the spiking source tested. Motor activity evoked in our wiping trial paradigm was usually brief and vigorous, with motor unit spikes generally restricted to higher motor states as motoneurons were increasingly recruited during motor behavior. In contrast, recorded interneurons often spontaneously spiked during both motor-quiescent and motor-active epochs of our recording trials. Accordingly, interneuronal spiking events were recorded during both low and high EMG signal states. We thus set about better identifying and predicting these state-dependent effects using SDO approaches.

### Identifying significant SDO effects

We used 10 ms intervals (20 points at 2,000 Hz) before and after (relative to) the spike to generate the pre-spike $p(x_{0})$ and post-spike state distributions $p(x_{1})$. This interval was selected to examine the immediate post-spike effects on signal behavior (corresponding to 1–3+ chemical synapses). However, arbitrary intervals are possible using the *SDO Analysis Toolkit*.

We proceeded as described in detail in the Materials and Methods. SDOs were exhaustively generated for every combination of spiking source and signal within the dataset [11 EMG × 283 (likely-redundant) spike trains = 3,113 potential SDOs]. We defined a “significant” SDO as a spike-triggered SDO matrix which differed from the respective spike-shuffled SDOs for any of the four tests of SDO effects as described in the methods, at a *p* value <0.05. Significant SDOs were used to predict single signal states and post-spike state distributions.

#### Example SDO analyses in spinal data

We compared the STA and SDO methods with the physiological data we collected from spinal frogs. We found that significant spike-triggered SDO matrices generally matched or improved the accuracy of the STA-based method when predicting both single post-spike states and probability distributions. When SDOs were significant, the confidence intervals of the cumulative prediction errors generated using the SDO [H7] versus STA [H3] matrix hypotheses often had no overlap when using 1,000–5,000 shuffles (i.e., there were no instances where performance of the STA and SDO were comparable), indicating “effect sizes” in the two methods lay beyond the observed distribution tails obtained from the bootstrapping (although specific *p* values are ill-defined in these instances). Other hypothesized matrix models had distributions that were more closely positioned and showed distribution overlaps that allowed estimation of probability values. Overall performance of SDOs was thus often much better than all other tested models including the classical STA.

**Figure 9.**
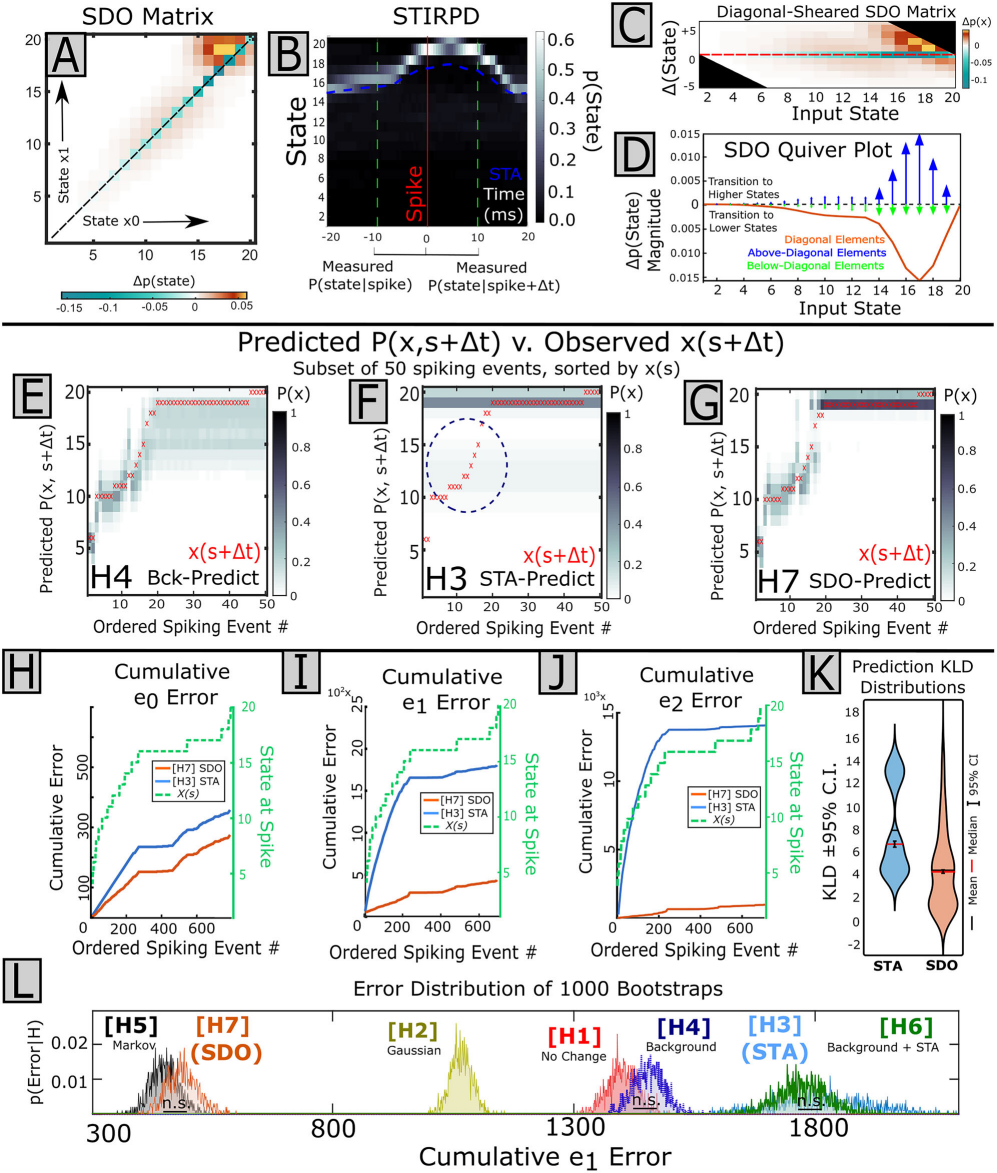
SDO analysis of a spinal interneuron and EMG amplitude: The spikes from a single spinal interneuron were compared against EMG signal amplitude from the vastus externus muscle (filtered zero-phase, acausally). ***A***, The spike-triggered SDO matrix (here, Gaussian smoothed for visualization). Spike-triggered effects are primarily associated with higher states (15–19), with an increased probability of transition toward relatively greater states, as positive elements are above the diagonal. ***B***, The extended STIRPD of the EMG signal shows a coarse relationship between spike time and signal state. After spike time, signal state appears to converge on state 18–19 with a probability ∼0.6. ***C***, The shear SDO shows the positive elements of the matrix are primarily concentrated above the diagonal over states 15–19. This suggests the spike-triggered SDO is consistent with a transition toward higher post-spike states for input states in this region. The slight diagonal orientation of the domains (parallel to the sheared top of the matrix) suggests the post-spike signal state is “stepping-up” to a particular state, rather than broadly increasing state, as first suggested by the STIRPD. ***D***, The SDO quiver plot shows the coarse directional effects of the SDO for each input state. Consistent with the shear SDO, the effect of this spike-triggered SDO is to support a transition toward higher states for input states 15–19, indicated by vectors above and below the abscissa pointing upward for these states. ***E–G***, For a subset of 50 spiking events, the predicted post-spike state distributions were calculated for each spike using the STA or SDO. Each predicted post-spike state distribution was represented as a column vector, ordered according to state at spike, and horizontally concatenated into a matrix, displayed here as a grayscale image. The single observed post-spike state for each spike is overlaid as a red *x* in the respective column. ***E***, The background SDO demonstrates state-dependent predictions independent of spike-triggered effects. Here the background SDO is well-suited to predict post-spike distributions when in a “lower” initial state but makes overly broad predictions at higher states. ***F***, Here, the STA can predict the post-spike state only over a limited range of experimental data (ordered spiking event 25+). The STA fails to accurately predict post-spike state distributions when predicting from a lower pre-spike state (indicated by the blue circle of observed post-spike states not covered by STA-predicted post-spike state distributions) but is accurate at higher states. ***G***, In contrast, predictions of post-spike state by the SDO are valid over the entirety of the dataset. ***H***, When predicting to single states, the SDO reduces both the frequency (*e*0), (***I***) magnitude (*e*1), and (***J***) sum-squared magnitude (*e*2) of prediction errors relative to the STA. This predictive accuracy is state dependent: The SDO and STA have equivalent performance at high input states, but the STA significantly underperforms the SDO's prediction error at lower states, consistent with D. ***K***, When predicting post-spike distributions, the SDO outperforms the STA [as measured by the Kullback–Leibler divergence (KLD) between predicted and observed post-spike states]. The distribution of KLD, calculated for every observed spiking event, is given as a violin plot. Here, lower values indicate less divergence from the observed distribution and hence, a better fit. Here the bimodality of the STA violin plot demonstrates the insufficiency of the STA to predict the post-spike state distribution for spikes occurring at “lower” states. ***L***, Significance of cumulative errors were tested using 1,000 bootstraps of *e*1 errors for all seven matrix hypotheses. Here, the distributions of SDO-predicted and STA-predicted errors do not overlap; *p* values are arbitrarily small.

Significant spike-triggered SDOs generated from interneurons to EMG signal amplitude analyses often displayed high tuning to signal state and strong state-dependent effects. In the example SDO analysis shown in Figure 9, as indicated by the SDO matrix, a strongly stereotyped relationship exists between an interneuron spike and signal behavior during high EMG states (Fig. 9*A*). This relationship could also be extracted as the impulse response from the time-domain STA. The “arch-like” rapid rise and fall of EMG signal state, observed in the STIRPD (Fig. 9*B*), is a consequence of using a zero-phase smoothing filter on relatively isolated motor units that were active and observed to be recruited at high intensities on the aggregate EMG channel. The peak of the filtered signal and the time of the motor unit occurrence on the channel is observed in the STIRPD image at state 20, ∼6 ms after the spike for the recorded interneuron. That is, the interneuron in Figure 9 consistently fires before spiking in the vastus externus (VE) EMG channel. However, this relationship between interneuron and motor unit in the aggregate EMG is state dependent: While the VE is in a signal state >14, a spike from this interneuron reliably predicts an increase in VE signal state (consistent with recruiting additional individual motor units). However, when the VE is in states 1–14, the spiking of the recorded interneuron no longer predicts a change of signal state. The SDO is tuned to this relationship and only demonstrates an effect at these higher states (Fig. 9*C*,*D*). In contrast, the simple classical time-domain STA averages over both conditions. When predicting post-spike states and distributions, pre-spike state dependency is important: The H3 STA matrix is accurate in predicting when signal state at spike is high (e.g., states 18–20) but is inaccurate when signal state at spike is low (Fig. 9*F*). In contrast, the SDO-based prediction captures this state dependency, matching the H3 STA prediction accuracy for high states, also greatly improving predictions for the lower states (Fig. 9*G*). The state-dependent background SDO generates reasonable predictions, independent of spike-triggered effects (Fig. 9*E*), especially when state is <15. This may indicate background, rather than spike-driven, dynamics predominate when state is <15. If cumulative prediction errors are quantified over all occurrences of spike, the SDO significantly, and greatly, reduces both the frequency (*e*_0_; STA = 356.95^k^, SDO = 287.8^l^, Cohen's *D* = −10.4) and the magnitude (*e*_1_; STA = 1,816.8^m^, SDO = 487.5^n^, Cohen's *D* = −15.5) of the associated cumulative prediction errors relative to the STA (Fig. 9*H–J*). The SDO and STIRPD thus capture a richer description of the spike-triggered signal behavior compared with the classical STA alone (Fig. 3). Predicted post-spike distributions deviate less from observed distributions using the SDO rather than the H3 STA, as displayed and measured using a smaller KLD (Fig. 9*K*). [Note that the empirical distributions, Fig. 9*L*, bottom, of these different bootstrapped cumulative errors do not overlap, and hence *p* values cannot be directly estimated (i.e., *p* values that would lie in long tails of the distributions that were never visited or captured in the bootstrapping of distributions would be significant, but the specific *p* values are ill-defined in these instances)]. If use of the classical STA is desired, the SDO and STIRPD can here inform about which spiking events should be used to estimate the STA impulse response and in what conditions it applies. Taken together, the combination of interneuron state tuning and spike-triggered effects can form the basis for the analysis of potential dynamical neural controls of motor behavior.

**Figure 10.**
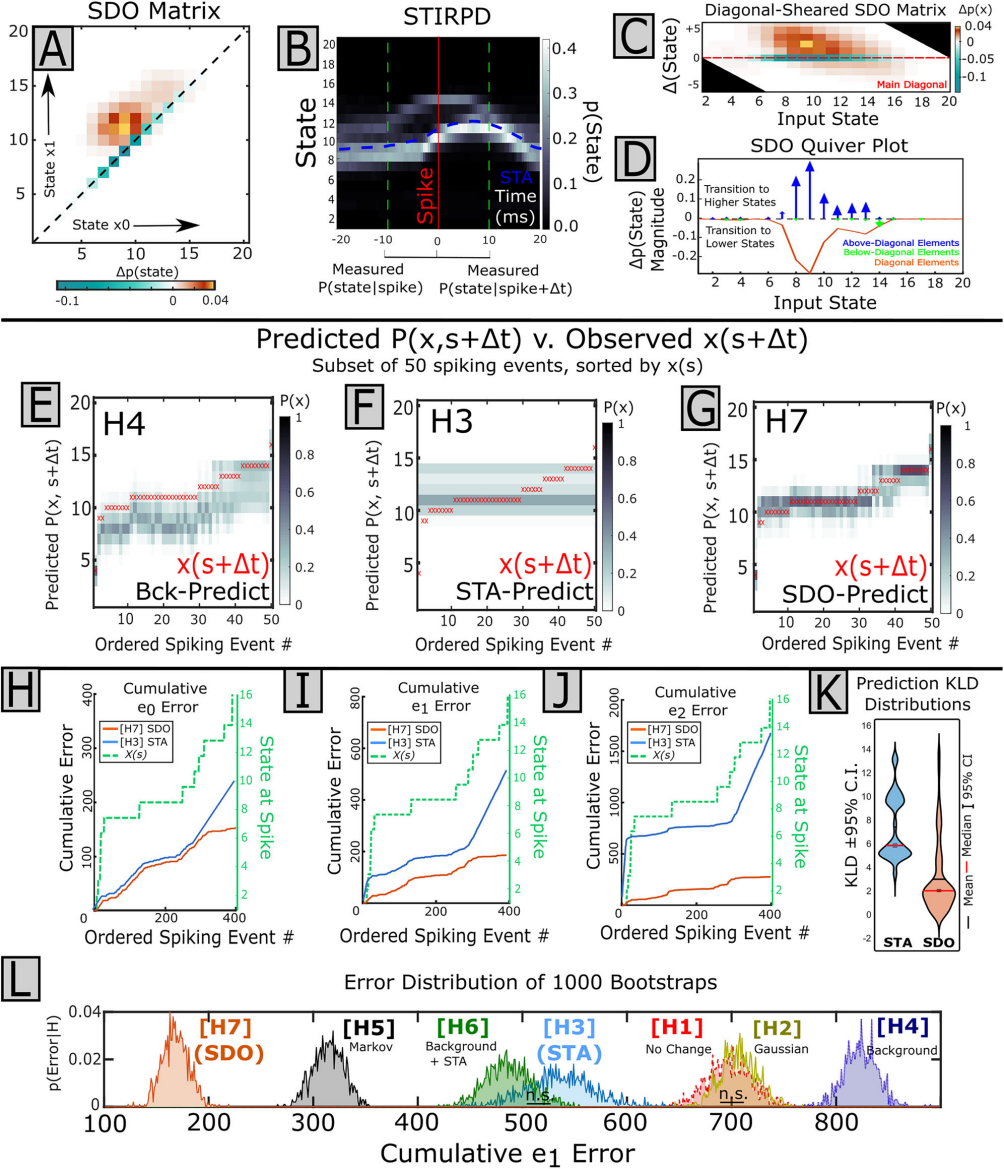
SDO analysis of a single motor unit and synergist muscle EMG: The spike train of a single motor unit (SMU) in the vastus externus muscle was compared against EMG signal amplitude of the biceps femoris (as in Fig. 3). ***A***, The SDO matrix. Effects are localized in two regions, about State 8–10 and 12–14. ***B***, The extended STIRPD of the EMG shows this SMU is tuned to two different states of EMG signal amplitude, as indicated by the bimodal behavior of *p*(state|spike). In the top “arc”, at state 14, the post-spike state distribution appears mostly symmetrical to the pre-spike state. However, in the lower arc (state at spike = 10), the post-spike states are increased relative to spike. ***C***, The shear SDO shows the positive elements of the matrix are primarily concentrated above the diagonal over states 8–12, corresponding to the lower “arc”, but minimal effects outside this region. ***D***, Similarly, the quiver SDO demonstrates coarse directional bias toward higher post-spike states for input states 8–12, corresponding to the “lower arc” on the STIRPD, but minimal effects for input states 13–16, consistent with minimal change to the “upper arc” of the STIRPD. ***E–G***, For a subset of 50 spiking events, the predicted post-spike state distribution was calculated for each spike using the STA or SDO. Each predicted post-spike state distribution was represented as a column vector, ordered according to state at spike, and horizontally concatenated into a matrix, displayed as a grayscale image. The single observed post-spike state for each spike is overlaid as a red x in the respective column. ***E***, Predictions from the background SDO are state dependent although inadequately capture spike effects. ***F***, The bimodal post-spike distribution predicted from the STA suboptimally predicts the observed post-spike state, while (***G***) the SDO-predicted distribution of post-spike state more tightly fits the observed post-spike state, across all signal states. Thus, the SDO provides a more reliable method of predicting signal behavior. ***H–J***, When predicting single post-spike states, the rate of error accumulation for different hypotheses depends on state. Post-spike signal state was predicted for every spiking event, for all hypotheses, and the error between each event-wise prediction was accumulated for all spikes. Spiking events were sorted by state at time of spike to uncover state-dependent error rates. Here the rate of accumulation for the (***H***) frequency (e0), (***I***) magnitude (e1), and (***J***) sum-squared magnitude (e2) of error are comparable for states 7–10 for the STA and SDO as indicated by the parallel traces of the cumulative error over this region), but the STA performs poorly at lower and higher states, whereas the SDO maintains prediction accuracy over all states. ***K***, The similarity between each predicted and observed post-spike distribution was assessed as the Kullback–Leibler divergence (KLD). The distribution of the KLD over all spiking events is displayed as a violin plot. Predicted distributions using the SDO resulted in a better fit than the STA. ***L***, Significance of cumulative errors were tested using 1,000 bootstraps of *e*1 errors (for all 7 matrix hypotheses, below). As suggested by ***E*** and ***F***, the STA results in a better prediction than the background SDO but worse than the spike-triggered SDO. Here, the distributions of SDO-predicted and STA-predicted errors do not overlap; *p* values are arbitrarily small.

In contrast to interneurons, vertebrate single motor units should be causally linked only to a single muscle's aggregate EMG and force. Nonetheless, intermuscular coordination (e.g., Hart and Giszter, 2004) may theoretically permit significant correlations between single motor units in one muscle relative to muscle activity in another. SMUs collected in our dataset demonstrated somewhat stereotyped motor tuning to signal behavior over the experimental interval. Likely unsurprisingly, tuning of aggregate EMG and motor unit was strongest for the parent muscle, although more complicated relationships could also be observed for synergist muscles (Fig. 10). In the example SDO analysis, the SDO matrix shows two regions of spike-triggered effects (Fig. 10*A*,*C*,*D*). Here, aggregate EMG signal behavior collected in a synergist muscle around SMU spike times visibly separates based on signal state at SMU spike within the STIRPD (Fig. 10*B*) and *p*(state|spike) and thus resembles a bimodal distribution. Just as the mean of a bimodal distribution may be a suboptimal description of the distribution, the STA calculation averages over this observed separation in signal behavior when predicting post-spike state in the synergist (Fig. 10*F*). Consequently, the H3 STA matrix predicts overly broad post-spike distributions, while the SDO-predicted post-spike distributions that better resembled the observed distributions (Fig. 10*G*). This improvement of the SDO over the H3 STA is similarly observed for predictions to most likely (singular) post-spike state (Fig. 10*I–K*). In this case, SDO methods resulted in significantly and much lower cumulative *e*_1_ (magnitude) error rate compared with the STA (*e*_1_; STA = 534.8^o^, SDO =167.9^p^, Cohen's *D* = −20.2). As diagrammed in Figure 10*L*, empirical distributions of these bootstrapped cumulative errors do not overlap, (i.e., *p* values that would lie in long tails of the distributions that were never visited or captured in the bootstrapping process would be significant, but specific *p* values are ill-defined in these instances, being beyond the observed distribution tails’ ranges). Similarly, predicted post-spike distributions deviate less from observed distributions using the SDO rather than the H3 STA, measured as a smaller KLD (Fig. 10*K*). Structure of statistical tests are reported in Table 10. 

This improvement in prediction is consistent with SDO methods capturing a richer description of the spike-related state-dependent effects relative to the classical STA. While increasing the number of parameters within a model tends to improve the fit to observed data, the SDO and probability distributions (and H3 STA), as utilized here, are treated as discrete representations of fundamentally smooth and continuous objects. Hence, from an Akaike or Bayes information criterion on model complexity, the exact number of parameters and degrees of freedom (DOF) will vary with chosen quantization of state (e.g., a Gaussian distribution measured within 100 binned intervals may possess only 2 fundamental degrees of freedom.) However, in predicting $p(x_{1}|x_{0})$ instead of $p(x_{1})$, the SDO likely always represents more DOF than the classical STA. Nonetheless, the information gain in using the SDO-predicted post-spike distribution to represent the observed distribution (as indicated by the KLD; Figs. 9*K*, 10*K*) is greater than for the STA predictions, while the efficiency of this information gain depends on SDO matrix dimension choices and data available.

### Classification of SDO types and motifs

**Figure 11.**
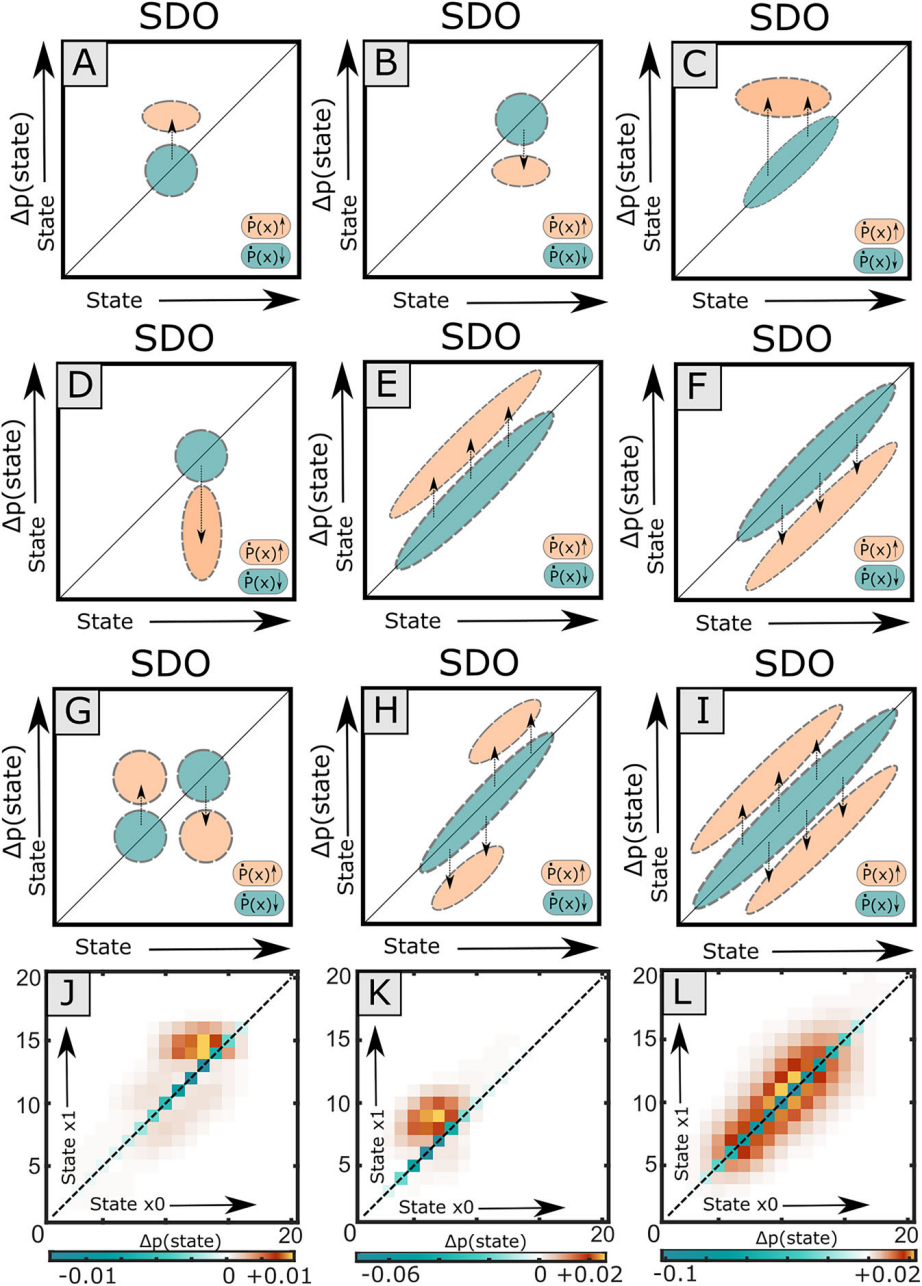
The effect of the SDO in using the pre-spike state distribution, *p*(*x*0), to influence the post-spike state distributions (*px*1) can be characterized and crudely classified from the localization of Δ*p*(*x*) in the SDO matrix. SDO features may have effects arranged along the matrix diagonal or display horizontal or vertical banding. Topologies may be combined to accumulate effects in a state-dependent fashion. The direction of the dashed arrows indicates the general state-dependent directional effects of the SDO conditional on that pre-spike distribution of state. ***A***, Step Up Operator. ***B***, Step Down Operator. ***C***, Convergence Operator (up). ***D***, Divergence Operator (down). ***E***, Increase Operator. ***F***, Decrease Operator. ***G***, Stabilize State Operator. ***H***, Destabilize Operator. ***I***, Diffusion Operator. ***J***, An example “Convergence” SDO. ***K***, An example “Increase” SDO. ***L***, An example “Diffusion” SDO.

When the state-quantized signal is smoothly varying and state distributions are drawn using a short interval, nonzero elements of the SDO are usually concentrated near the matrix diagonal. Accordingly, the SDO is a sparsely sampled matrix, with zero-value elements where state transitions are not observed and nonzero elements reflecting the extent of *P*(state|spike) over all spiking events. Positive elements of the SDO indicate increased probability of the state transition (*j → i*), while negative elements indicate decreased probability of transition (*j *→* i*). This permits the localization of positive and negative domains in the SDO to be descriptors of the correlated “actions” of a spike on signal behavior (Fig. 11).

The state-dependent changes in post-spike distribution enacted by an SDO could be inferred by viewing the matrix directly. We identified nine classes of simple features or “motifs” which represented specific types of changes observed in the data. These motifs are summarized in Figure 11 for sparse matrices. Figure 11 shows important examples of simple sparse SDOs forming elementary “motifs.” When evaluating motifs, both the normalized and parameterized SDOs should be considered: The normalized SDO displays spike-triggered “effects” while the parameterized SDO scales these effects by the frequency of their occurrence. This summary helps illustrate how SDOs can be intuited and interpreted when the SDO matrix is sparse. These SDO motifs can be classified as below.

#### “Step-up/down” operator (Fig. 11A,B)

These elementary SDOs are characterized by a restricted negative domain on the matrix diagonal, with a similarly restricted positive domain above (Fig. 11*A*) or below (Fig. 11*B*) the diagonal. This restriction limits the number of states modified by the SDO and generally reflects motor tuning for the spike–signal relationship. The Step-Up and Step-Down operators decrease the probability of maintaining state and increase the probability of transitioning to a narrow band of post-spike states, from a limited band of input pre-spike states. This elementary operator was the most common from single motor units to EMG which demonstrated significant motor unit tuning.

#### “Convergence” operator (Fig. 11C,J from spinal data)

This elementary SDO is characterized by a horizontal band. The Convergence SDO increases the probability of transitioning to a restricted band of states in the post-spike distribution from a larger band of input (pre-spike) states. (Note that this domain may occur above or below the matrix diagonal.) This SDO usually corresponds with a decreased variance of the post-spike distribution. This operator was commonly extracted in the normalized SDO matrix when there was a strong reversion to a common post-spike state distribution (i.e., converging on a common state distribution).

#### “Divergence” operator (Fig. 11D)

This elementary SDO is characterized by a vertical band. The function of Divergence SDO is opposite of the Convergence SDO: Pre-spike input states within a limited band (defined by the horizontal breadth of this motif) are distributed to a broader output band of states (defined by the vertical breadth of the positive domain of this motif), which may be above, below, or on either side of the matrix diagonal. This motif may emerge if the range of states in the pre-spike distribution is limited (e.g., spike state tuning), but the range of states in the post-spike distribution of states is not as tightly controlled. This bidirectional increase in signal dynamic range can also occur when the spike impulse response is multimodal (such as a single motor unit action potential), and the signal fluctuates stochastically about a mean value (such as unrectified EMG or the simulated white noise process).

#### “Increase/decrease” operator (Fig. 11E,F,K from spinal data)

These elementary operators are characterized by a positive diagonal band either above (Fig. 11*E*) or below (Fig. 11*F*) the main diagonal. Because positive domains run parallel to the matrix diagonal, the effect of the Increase or Decrease operator is broad, describing a shift of the post-spike distribution toward increased or decreased signal state. If these bands are adjacent to the main diagonal, these operators permit a smooth drift of the post-spike distribution. Crudely, these would correspond to excitatory or inhibitory synaptic effects, respectively, if observed neuron-to-neuron or neuron-to-EMG. This SDO motif maps reasonably well onto the classical STA method for the states of origin. Indeed, in simulated data, the H3 matrix (STA) produced this SDO when there was a clear and consistent post-spike facilitation or inhibition of signal amplitude.

#### “Stabilize” operator (Fig. 11G)

This SDO resembles the co-occurrence of the Step-Up and Step-Down operator acting over a clustered band of states. When the input pre-spike distribution trends toward lower states, the Stabilize operator increases probability of transition toward higher states (“step up”). Simultaneously, when the input pre-spike distribution trends toward higher states, the Stabilize operator increases the probability of transition toward lower states (“step down”), the net result stabilizes the probability of state in the center of the motif. This is equivalent to a negative feedback controller around the setpoint state at the center of the motif and was indeed recovered from both the background and spike-triggered SDOs from stabilizing dynamical systems (e.g., Y4, Y7).

#### “Destabilize” operator (Fig. 11H)

This SDO resembles the co-occurrence of an Increase and Decrease Operator concatenated along the diagonal. The function of the Destabilize operator is inverse of the Stabilize operator: Lower input states are pushed toward lower output states and higher input states toward higher output states. Input states near the middle of the Destabilize operator will be “destabilized” and may be pushed toward a bimodal post-spike distribution, away from the initial state.

#### “Diffusion” operator (Fig. 11I,L from spinal data)

This SDO is characterized by a negative region along the diagonal, with positive diagonal bands both above and below the diagonal. The function of the Diffusion SDO is to broaden the distribution of state (i.e., increase the variance of the post-spike probability distribution), similar to diffusion in a fluid. If the magnitude of Δ*p*(*x*) above and below the matrix diagonal is comparable within a column of the SDO matrix, the increase in variance due to this operator does not necessarily change the mean of the post-spike distribution. The Diffusion operator was the most common SDO in our physiological datasets when describing background signal dynamics with shuffled timestamps.

Examples of three such motifs from our physiological data are shown in Figure 11*J–L*.

## Discussion

We have demonstrated that SDO methods capture dynamics and spike-correlated information better than the classical STA method in both real and simulated datasets. While we have demonstrated that the SDO is a useful method, the extra information it offers may not always be necessary. In simulated model data, the tested STA methods of significance were sensitive to both consistent (Generator Y3) and random (Generator Y2) spike-triggered impulse responses within white noise and in signals on a dynamic background (Generator Y6). Detecting random effects (Generator Y2) may be important but are not part of classical circuit-breaking spike-triggered averaging identification techniques. The SDO description makes such stochastic effects explicit. However, the classical STA is robust and computationally simple: Signal is collected around spiking events and averaged together. The extracted waveform can serve as a template for the correlated “effect” of the STA, measured as the change in this average signal. This STA feature is particularly useful for reverse correlation. Similarly, because the average signal amplitude is calculated independently for each aligned time bin in the STA, the duration of the pre-spike and post-spike windows generally do not affect significance of the STA.

However, the STA was incapable of consistently assigning significance when spike-triggered effects were dynamic (Generator Y7). Further, SDO-based tests of significance were both more sensitive (less false negatives) and specific (less false positives) than those of the classical STA for identifying significant state-dependent relationships between a spike and signal across all simulated data. This is likely a function of the state-dependent operations and bootstrapping procedures standard to the SDO method here. When applied to physiological data, our results show that SDO analysis provided superior, more nuanced descriptions of spike-correlated signal behavior than the classic STA impulse response when complex interactions were present. This held true for both spinal interneurons and single motor units’ correlations to homonymous or synergist EMGs. The STA estimates the spike-triggered response amplitude using the means of the distribution of observed amplitudes across all spikes. Thus, the STA depends on assumptions of mean and normality as optimal descriptors of the response distribution. The example data here show these assumptions are violated in some physiological data by multimodal distributions of output state (e.g., STIRPD Fig. 10*B*) and state-dependent output behavior (as in Fig. 9*B*). While these factors may be handled with additional explicit consideration and experimental design with the STA, because SDOs are probabilistic descriptors, SDO methods provide tools that straightforwardly capture these features of neural data automatically.

SDO methods measure changes in distributions of signal state rather than the average amplitude of the signal at every time index. This method trades sensitivity in the time domain for sensitivity to signal variance. When there are strong relationships between signal and time, such as motor units to the homonymous muscle in the data analyzed here, the STA can capture these time effects as mean signal amplitude. The accumulation of state into pre-spike and post-spike distributions for estimating the SDO may compress this time-domain information depending on interval size and binning choices. In these cases, the STIRPD with appropriate binning provides a reasonable compromise, behaving as a quantized, probabilistic, STA. SDO reanalysis with informed parameters for finer time granularity and a chosen latency is then always available and possible.

### Caveats of state definition for stochastic dynamic operators

Because the SDO describes changes in state, the definition of state for original signal amplitude is important. The number of bins, selection of signal levels, filtering, and transformation of the signals (e.g., log-transform) will all change the representation of the state space time series. The definition of state (i.e., the quantization scheme) must be sufficiently sensitive to capture changes in signal state distribution needed to detect significant correlations with the SDO: If signal amplitude before and after the spike is assigned to the same state, there will be no measured change in pre-spike and post-spike distributions (i.e., the SDO matrix will resemble our null hypothesis model H1). This sensitivity can be established a priori because state is often derived from scaled amplitude ranges of signals with real units (e.g., for a signal with a dynamic range of 10 mv, 20 equal-width binned states are sensitive to effects >0.5 mv of original signal). Amplitude quantization and time discretization to state bin width determines the minimum detectable effect in state space. Explicit, intentional, determination of dynamic range will likely be necessary for detecting significant effects with the SDO. As the number of states increases, sampling of state-to-state transitions observed within a finite dataset necessarily decreases. Hence, state numbers used in SDO analyses represents a trade-off between resolution, information, sampling theory, experimental constraints, and resulting statistical power. In our results, 20 states were sufficient to detect significant correlations between spikes and signal behavior, without requiring an inordinate number of spikes.

Similarly, temporal relationships between spikes and distributions constrain spike-triggered SDOs. Shorter time windows are clearly more sensitive to high-frequency (quantized) signal content than longer time windows which effectively filter the high frequency, as with any choice of binning intervals. Latency in determining when to define “pre-spike” and “post-spike” intervals is similarly important (i.e., is effect of spike near-immediate or delayed and by how much?). SDO predictions thus capture spike effects strictly in context of experimenter signal processing and analysis choices. As with the STA, the selection of the pre/post-spike intervals for the SDO will depend on the question and goal. Hence, the duration of the pre/post-spike intervals, Δ*t*, remains as a free parameter within the toolkit, as does the latency between these intervals. We used an interval of 10 ms here, consistent with the expected effects within our experimental model. That said, the SDO describes the change in state probability across the entire pre-spike and post-spike intervals; interpretations of the SDO are contingent on the time intervals chosen for these distributions. In the extreme case, using a single time pre-spike and post-spike time bin for deriving (binary) pre-spike and post-spike distributions, the SDO essentially becomes a Markov description. Alternatively, with excessively long pre-spike and post-spike intervals, the spike-driven changes may be indistinguishable from background, and the potential spike effects may thus be lost. Finally, it is also possible to use short intervals at successive latencies after spike to capture the evolution of the distribution, which is effectively an SDO analysis of the STIRPD. In the examples here, we presented one latency and binning strategy for purposes of demonstration. When tested in simulation, this combination permitted the SDO to be both more sensitive and specific than the STA using only 250 spikes and 1,000 shuffles. To increase utility, we permit these parameter choices to be user defined within the *SDO Analysis Toolkit*.

Predictions of both post-spike state distributions and singular (“best”) states were compared between the STA (H3) and SDO approaches. Experimentally, most arbitrary combinations of spiking source and signal did not produce significant SDOs (or STAs) in physiological data. Nonsignificant SDOs (using our five measures of matrix significance) usually resembled Diffusion operators (hypothesis H2; Fig. 11*I*,*L*), enacting an unbiased passive increase in signal variance over time. Diffusion operator SDOs could, in principle, be statistically significant but were not in our data. Nonetheless, spike-triggered SDOs which were significant generated superior predictions relative to the H3 STA. All seven model hypotheses were implemented as matrices which satisfy the definition of an SDO, albeit generated by different strategies. Hence, even if the spike-triggered SDO matrix (H7) estimation is suboptimal compared with the other prediction model hypotheses, the model of best fit may still be assessed using SDO methods in the *SDO Analysis Toolkit*.

### Interpretations of SDOs and state tuning in examined spinal data

In vertebrates, single neurons usually act as part of larger organizational units. It is thus not unreasonable to assume the effects of a neuron will depend on the activity of covarying units and system state. Spinal interneurons must, to some degree, be both sensitive to ongoing motor state and capable of influencing motor state (Auyong et al., 2011a). The classical STA can show clear interneuron effects (Hart and Giszter, 2010; Takei et al., 2017). Similarly, significant SDOs in our data were also extracted when signals showed such statistically consistent behavior in the post-spike interval, as visualized on the STIRPD (Figs. 9, 10). However, the correlation between interneurons and motoneurons to EMG were also sensitive to pre-spike signal state in our data. SDO methods could detect and capture these pre-spike dynamics and state effects.

In our data, unsurprisingly, significant SDOs were often observed between single motor units (SMU) and homonymous muscle EMG. This is consistent with the motor unit summation constructing the recorded EMG signal following the size principle (Henneman et al., 1965). However, significant SDOs also existed in our data for the same spiking SMU against EMG signals across multiple muscles, with similar motor tuning. For example, the stereotyped “arcing” observed in distributions for the biceps femoris muscle (STIRPD; Fig. 10*B*) corresponded to the likely spiking of correlated motor units, recorded in the aggregate EMG implanted into the biceps muscle. The reliable corecruitment of motor activity in biceps’ aggregate EMG accompanied the single motor unit in the vastus externus, recorded with fine-wire braided probes, is consistent with synergy-based control of motor pools, and also some features of human data reports (Laine et al., 2015; Negro et al., 2016; Del Vecchio et al., 2022; Hug et al., 2023).

Vertebrate SMUs are confined to a single muscle: SMUs thus cannot be directly causal to heteronymous muscle EMG. However, in synergy premotor drives and in the spinal reflexes, activity of single motor units may be tuned to the state not only of the homonymous muscle but also synergists and antagonists. Insofar as muscles have correlated coactivation at (fast) time scales examined here, synergist motor unit spikes and EMG will correlate during observed cocontractions. Common synergist premotor drives could directly induce such correlations (Kargo and Giszter, 2000; Hart and Giszter, 2004). Such correlations may be state dependent: Synchronization between motor units is more frequently observed at low-force levels than during high forces (Basmajian and De Luca, 1985; Semmler et al., 2000). SDO-based methods can identify and estimate stochastic and state-dependent effects, which may provide additional predictive power for analyzing these correlations.

### Ideal SDO estimation

An ideal SDO should minimize prediction error and be generalizable to additional data. As an iterative method, our constrained optimization implementation always required considerably longer compute time than the direct linear method for estimating SDOs. Optimization was expected to meet or exceed performance of linear estimation, but we found that it usually underperformed the linear method when using 250–2,000 spikes within simulated data (Tables 8, 9) and varied more when training across different data fractions (Table 1). This result may indicate that the optimizer used here became trapped in local minima or had insufficient example data. Other optimizer implementations may conceivably converge to the global minima with more spikes, different optimization designs, or additional constraints beyond those strictly defined for the SDO matrix. We used the MATLAB *fmincon* solver with the *interior-point* algorithm to minimize the mean-squared error, but other options are available. Further work may refine the SDO optimization approach and improve convergence but may potentially still require more data than linear approximations. Conversely, the linearly estimated SDO (Eq. 14) is not guaranteed to minimize prediction error and likely can be improved upon. However, our primary objective in the research presented here is to provide a complement and expansion to the STA, one with enhanced sensitivity and which adds description of state-dependent spike effects using modestly sized datasets. The direct linear estimate of the SDO accomplishes this task in a computationally efficient fashion. Further, the direct linear estimate is suitable for iterative real-time applications.

### SDO sensitivity and caveats

When using the STA and SDO as explored here, we treated spiking events and spike–signal correlates as independent, with the spike impulse response as linearly estimated across samples (although the SDO permits for state dependency in response). In circumstances where signal likely facilitates spike (such as stimulus-triggered averaging in the visual system), spike-triggered averaging of the stimulus in the pre-spike interval can identify the average filter (or combination of filters) which are the maximum likelihood estimation of the signal facilitating a response. In these cases, deviation of the neuron from the linear–nonlinear Poisson (LNP) model implicit to the STA becomes particularly important: Estimating the average post-spike facilitation of a bursting neuron may be straightforward but estimating the stimulus triggering the burst (if indeed there is a burst) may not. In these instances, both the classical STA and SDO might sometimes produce spurious correlates. Alternative estimations of the reverse-correlated STA have been proposed to manage deviations of the neuron from the LNP model (Paninski et al., 2004), but these have not currently been implemented for the use of the SDO for forward correlation between spike and signal as presented here.

Neither SDOs nor classical STAs can directly infer causality between a spiking process and a signal nor the mechanism underlying the stochastic process. Determining causality between observed processes requires explicit experimental manipulations (e.g., driving the neuron). Nonetheless, correlative models are useful predictive tools and can indicate the need for further causal experimental tests. (Indeed, in neural encoder/decoder frameworks, the circuit “function” of a neuron may often be divorced from its capacity to reflect or predict network state.) Here, SDO analysis can augment other correlative tools such as blind source separation methods (e.g., independent components analysis and non-negative matrix factorization). SDO frameworks can integrate correlation patterns and recordings of interneurons, as observed in data here with some interneurons predicting signal states across multiple muscles. When neurons are known, or modeled, as differentially contributing to motor behavior in neural circuit configurations (e.g., segregation of rhythm and pattern in locomotion; McCrea and Rybak, 2008; Danner et al., 2017) and spinal “state” (Ausborn et al., 2018), SDO methods may provide useful exploratory tools. As far as different SDO motifs capture significant state-dependent spiking behaviors within the data, different classes of neurons should generate different SDO motifs: SDO matrices are thus likely useful descriptive classifiers.

The SDO framework can be extended. We demonstrated SDO analyses using single-channel EMG time series data and stochastic simulated data, although more complex applications are likely to be straightforward. In the depiction of the SDO presented here, “state” is defined using amplitude of the same signal correlated with spike. The SDO may be extended to incorporate additional orthogonal dimensions of state by evaluating changes to joint probability of the form $\Delta p(x_{1}|x_{0},\ldots \theta )$, where *θ* can represent any number of independent quantizable parameters. (Although in this instance, the dimensionality of the SDO matrix will grow by the number of discrete variables and may no longer be interpretable as a 2D matrix.) SDOs may thus be scaled as necessary to address the experimental question.

SDO analysis may thus be applied to combined or derived signals (e.g., activations of independent or principal components, rate processes, neural ensemble-derived dimensionality reductions). For example, spiking events may be correlated with projection component features obtained from higher-dimensional datasets (Smith and Brown, 2003; Churchland et al., 2008; AuYong et al., 2011b; Raposo et al., 2014; Lindén et al., 2022; McMahon et al., 2022; Thura et al., 2022). The extension of SDOs to oscillatory processes via the definition of a stochastic phase variable may be of particular use for linking single-unit effects with population dynamics (e.g., neurons may be sensitive to network phase, or evoke phase-dependent effects) but will require additional validation beyond what has been presented here. On these frontiers, SDOs provide a new means to visualize and explore predictive influences of specific spike trains in these dynamics and based on state variables.

Our SDO methods described here provide a framework for experimentalists to explore state-dependent and probabilistic relationships within data and to test generative parameters within the *SDO Analysis Toolkit*. A repertoire of statistical tests is used to identify significant SDO matrices and to test SDO-based predictions. SDO matrices are tested for significance using Monte Carlo bootstrapping using our five tests of matrix composition. Predictions of signal behavior are tested using seven matrix hypotheses, including the SDO and the STA, to determine the model of best fit in observed data to both single state and distributions. While we cannot provide an exhaustive survey of potential SDO topologies, Figure 11 provided a rudimentary classification to interpret SDO “function” within recovered SDOs. We believe the combination of visual intuition with mathematical foundations provided by SDO analysis supports more precise and nuanced analyses of a spike effect relative to the classical STA alone.

### Conclusions

Here we introduced and demonstrated SDOs as useful analysis tools in both physiological and simulated data. SDOs extend the classical STA into probabilistic and state-dependent domains, behaving as a state-dependent spike-triggered average which may better capture spike correlations and potentially causal effects. The SDO offers improved sensitivity and specificity for identifying significant relationships between the spike and signal relative to the classical STA. The utility and function of SDO methods can be intuited through motifs on the SDO matrix, the shear and quiver SDO visualizations, and the STIRPDs. Together, these methods capture classic STAs and graphically reveal significant and state-dependent stochastic behaviors. SDO analysis is thus a novel method for both qualitative and quantitative data interpretation. We demonstrated the utility of SDOs as a flexible tool for describing relationships between spinal interneurons, single motor units, and aggregate muscle activations. We showed that using SDOs and STIRPDS in place of the classical STA can better characterize post-spike effects, account for noncorrelated background dynamics, improve sensitivity and specificity of detecting significant spike–signal correlations, and significantly increase prediction accuracy. SDOs provide a statistical model of neural effects, are easily and efficiently obtained from existing data, and can be empirically tested for statistical significance. The SDO may be readily estimated with data collected on multiple time scales and experiment types, including stimulation and interventions, in ways which far exceed applications described here in simulated and spinal motor control data. In summary, SDO analysis is a powerful but accessible method, and we anticipate both the framework and *SDO Analysis Toolkit* here to augment the general Neuroscience toolbox.

## Data Availability

The SDO analysis and data figures were produced using the *SDO Analysis Toolkit*. The code/software described in the paper is freely available online at https://github.com/GiszterLab/SdoAnalysisToolkit.

10.1523/ENEURO.0512-23.2024.d1 SDO Analysis Toolkit Download SDO Analysis Toolkit, ZIP file.

## Synthesis

Reviewing Editor: Arvind Kumar, KTH Royal Institute of Technology Decisions are customarily a result of the Reviewing Editor and the peer reviewers coming together and discussing their recommendations until a consensus is reached. When revisions are invited, a fact-based synthesis statement explaining their decision and outlining what is needed to prepare a revision will be listed below. The following reviewer(s) agreed to reveal their identity: Michael Denker, Arunava Banerjee. Note: If this manuscript was transferred from JNeurosci and a decision was made to accept the manuscript without peer review, a brief statement to this effect will instead be what is listed below.

Synthesis

The two reviewers have appreciated the changes made by the authors in the manuscript. But the analysis has raised some new concerns. Moreover some of the concerns raised in the previous version need further work. Importantly, one the reviewer has identified an issue that he/she missed in the previous round - i.e. finding the operator L is a constrained optimization problem and solution to this problem is not in the n Eq 6.

The detailed comments are appended below to help you revise the manuscript.

Reviewer #1

The manuscript represents a resubmission of a previously submitted work. I acknowledge that the authors have taken the time to make substantial improvements related to points raised in an earlier review. In particular, the authors have added an extensive section on various controlled artificial data that they use to benchmark the method and addressed concerns regarding the method for significance testing.

A few points remain that need clarification, including one serious concern regarding a potential bug affecting the experimental analysis, as outlined below.

Major

-----

1. Both STA and SDO have a time scale $+-\Delta t$. In constructing the distributions for SDO (e.g., line 217) however, this distribution likely heavily depends on this choice due to the aggregation of amplitudes across the observed pre or post intervals. How is \Delta t chosen as a free parameter and how does it affect the analysis?

This is particularly well illustrated in the example presented in Figure 4, where I was confused why the clear bimodal structure seen in B and C is completely wiped out in the post-spike red distribution. Here, it seems that the most likely reason is that the "A" signal rapidly drops off between 5 and 10 ms. A change of \Delta t would likely have strong consequences for the analysis.

2. Something is wrong in Figure 2D: As expected, the quantized trace (bottom) starts out fully synchronized with the filtered trace (middle). However, as time progresses the bottom trace starts to lag behind the filtered trace, which is clearly visible, e.g., at time t=15 s. Mathematically, the quantization should not lead to this problem. Please check this error, and check that it does not systematically propagate to all other results of the paper, and otherwise repair the results. Given that a time shift of the continuous signal could have strong consequences, unless also spike times undergo the same shift, this may have serious consequences for spike-signal correlations.

3. In Figure 4, despite the explanation of how the states are defined in the caption with state 10-11 corresponding to "base line", it's very difficult to relate the signal amplitude (panels A and B) to the state (panel C). In particular, it seems that all the interesting dynamics in the STA is quenched into 4 states (10-14), and all other 16 states are more or less unused. What is the rational for such a coarse graining of the interesting region between 5 and 70 mV?

Also, given the fixed number of 20 states in the implementation, line 429, would this not be something that should be more based on the actual signal?

4. The authors have made a very thorough new analysis aimed at detecting whether STA and/or SDO can detect the "STA effect" in 8 different stochastic model setups. I find this analysis very useful, as it gives the reader a much better intuition on how the method performs under various controlled conditions. One question still remains on my mind though. In the results, e.g., Figure 9L and 10L, the authors estimate the most predictive hypothesis/model (H1-H7) based on the model data. Given that to my understanding, Y1-Y7 are attempting to model H1-H7, can a similar statement be made here, i.e., that Y1 data is best predicted by an H1 model? Given that the STOs of many Y's look very similar, this might be interesting to understand the conclusions that can be drawn from such an analysis.

Minor

-----

- Line 31: either "a spike and a signal" or "spike and signal"

- Line 31/32: a spike may be affected by a signal, a spike may effect

- All over the manuscript: There is a mismatch as STA is defined on line 28 as "spike-triggered averaging", but in some sentences it is used as "spike-triggered average". An example is line 35 or line 172, where the same abbreviation is used twice in a row to mean different things. I believe the community standard would be the latter, i.e., STA=spike triggered average. In that case, line 28 would need to be changed, as well as all places where STA is used to mean spike-triggered averaging (one example is line 35: STA has identified -> The STA has been used to identify). Otherwise, the reader will stumble over these sentences and suspect missing articles in front of STA.

- Line 136: period is missing.

- Line 140-141: High-pass->high-pass Low-pass->low-pass

- Line 179: the signal amplitude

- Line 182: the concurrent signal amplitude

- Line 183: no comma behind "events"

- Line 197-200: The sentence is very convoluted and difficult to read.

- Line 200: It is unclear why the envelope is mentioned here, and what envelope the authors refer to. Do the authors refer to z-scoring the signal itself? Otherwise please explain.

- Line 215: discrete states

- Line 219-220 and line 250 and line 306: The sentences do not need to be put in parenthesis, to improve readability.

- Line 220: effect of a spike

- Line 221: to be more precise, I suggest to clarify that the effect of a spike is "the change in mean amplitude in time"

- Line 234: I think the underlining is not necessary, the point is now made very clear in the text.

- Line 243: Underlined word.

- Line 244: Either "Predictions of ... are made" or "The post-spike distribution ... is predicted"

- Line 246: Equation should be further up to avoid forward reference of Eq 2.

Line 304-305: I am not sure this statement is true. As a counter-example imagine a spike-locked fast oscillation. This would not yield a flat STA, yet, H1 would be satisfied (given Delta t is larger than the period of oscillation.

- Line 306-307: Sentence is broken. Where is the convolution in the formula?

- Line 310: a spike. There are multiple other places, esp. in the methods, where I would think an article "a" before the word spike is missing.

- Line 315-316: Reads very badly.

- Line 327/328: I do not understand the second part of the sentence.

- Line 365: Given that there are 7 H's and 8 Y's, is it possible to make the link between the models and artificial data a bit more explicit for the reader, e.g. something along the lines of: "...7 methods Y1-Y7 corresponding roughly to H1-H7, respectively, and one additional method Y8.."

- Line 458: Given that the 3 types of SDOs are defined all over the Methods part, I think it may help very briefly remind / reference what the background and shuffled SDOs refer to.

- Line 679: What is the interpretation that the background prediction yields similar effects as the spike-triggred SDO? Does this mean that the spike has very little effect for this particular example?

- In all figures that contain text, there is a mixture of capitalization and non-capitalization of words. Please make the figures more consistent.

- Figure 1 caption line 147: The caption refers to the "STA effect", however, this is only defined much later in the text on line 220.

- Figure 1 caption line 150: "average Trigger averaging EMG" is unclear, please rephrase.

- Figure 1 caption line 151: "both conditions" should probably be made explicit (I assume: with and without PSI?).

- Figure 2 caption line 162: The abbreviation SMU is not explained, and in the text is used for the first time only much later.

- Figure 2 caption line 169: Why "EMG envelope"? From the figure, I would assume a high signal state corresponds to a high signal amplitude, not envelope. Otherwise, please show the envelope that was used for classification into states.

- Figure 3 and 4: The word "draw" inside the figures seems strange to me. Are the distributions created by random sampling (drawing) or by simply constructing the distributions from all available samples?

- Figure 4: mv->mV (I assume)

- Figure 9: Should panels E,F,G not have similar labels of H4 H3 and H7, as in Figure 10? This would help the reader in understanding the plot.

Reviewer #2

The authors have verified SDO in simulation data as suggested by this reviewer in a previous submission.

However, there is an important issue that needs to be resolved before this reviewer can sign off on the article. In the paragraph referenced in lines 269-280 the authors rightly point out that the operator L has to satisfy four constraints in order to be a valid SDO operator. This is therefore a constrained optimization problem and the solution for a given dataset is therefore not the one noted in Eq. 6, which corresponds to a standard least squares solution to extract L (this solution does not explicitly satisfy the noted conditions). I could not find where in the paper the authors solve for the constrained optimization problem. This needs to be fixed, and tables and graphs re-computed.

Also, there is a small error on line 257. R_xx and R_xy are flipped.

## Author Response

We thank the reviewers for taking the time to review our manuscript and for their constructive comments. Using these comments we have performed a substantial revision of the paper and added code, further improving and validating the SDO methods, and our presentation of these. We have reorganized the manuscript somewhat in order to allow for more explicit parallels to be drawn between STA and SDO analysis. This permitted us to streamline the material slightly and reduce redundancy in discussing and introducing the STA and SDO methods.

Please find below our detailed responses to the comments; we hope they are to the reviewers' satisfaction.

Concerns Raised Reviewer 1-1 : "The authors provide a very detailed and extensive report on a new analysis method extending the idea of spike-triggered averages (STAs). Only until reading fairly far into the manuscript does it becomes apparent that the proposed method uses the STA to investigate whether the spike time is associated with a state transition in terms of the values assumed by the time series before and after the spike. This process removes the temporal aspect of the time series (except for the few instances where M models the time series as a Markov process). As such, the method seems less adequate in some situations where STAs are used, e.g., when investigating oscillatory time series. It would be useful to discuss in more depth (as early as possible) the type of analysis scenarios and hypotheses SDO can consider and the types of time series adequate for this analysis. For example, STAs may be used in other scenarios than that described, e.g., on line 186-187." In our revision of the manuscript, we introduced this topic earlier during the direct comparisons with the STA. Our method is focused on potentially forward causal effects of spikes, rather than reverse correlation analyses, as often also performed using STA. Our method can be adapted to oscillatory time series, if focusing on forward causal analyses as we do. However, we do not elaborate this aspect here, where it would further complicate descriptions in what is already a complex exposition. When taking a distribution of state across the pre-spike or post-spike window, as with any binning method, we do lose some temporal information using the SDO method, in exchange for better capturing the variability of the state dynamics. However, if desired by narrowing the temporal intervals, granular temporal details can be captured using multiple successive serial SDOs with short duration bins for state, each at increasing latency. When there are strong time effects, we point to the STIRPD as a visualization, permitting visualization of both state dependent behaviors and strong time effects, and when an SDO(t) .

Reviewer 1-2: "In general, I am not convinced of the significance estimation of SDOs. When using random background timings to calculate surrogate SDOs, does this not also alter the pre (prior) distributions and p(x_0)? Why is it valid to compare these surrogate operators against the one of the STA? At the same time, Figure 9 shows that the background SDOs are highly similar to the estimated spike-triggered SDOs, which may indicate that SDOs capture mainly intrinsic dynamics of the signal. One may speculate in how far prediction errors when predicting distributions at the same random spike time used to calculate the background operator are similar to prediction errors used when predicting distributions at spike times (Figure 9L, H7). I think the significance estimation needs further clarification and justification." The goal of significance testing is to examine if the additional gains of information from spike occurrence relative to a background SDO are significant. A significant result indicates that the spike changes distributions beyond the background dynamics. Thus, the idea that SDOs capture prior intrinsic dynamics absent the spike is an explicitly tested (and if significant) and rejected hypothesis. We have further expanded the methods section on significance testing. Based on the concern of potentially different prior distributions of state in experimental vs. shuffled spike trains, we have modified the code such that before significance testing, the surrogate/shuffled SDOs are now first rescaled to have the same prior distribution as the experimentally-derived SDOs. This permits us to detect if there are significant differences in the prediction of dp(x_1), without spurious correlations due to differences in state at time of spike for shuffled vs. experimental spike trains.

It is true that the SDO captures all dynamics around spike, which may be spike-triggered and intrinsic. Our use of Null Hypotheses H4 ('Background SDO') and H6 ('Background SDO + STA') are used to evaluate the' background SDO' as sufficient to describe signal dynamics local to spike, and if any spike effect is state dependent. The 'background SDO' is calculated from all measured timepoints, and hence represents the overall expectation of signal dynamics. When the prediction performance of the background SDO vs. the spike-triggered SDO do not differ significantly, we consider the spike-triggered effect not to be significant. The framework clearly deals with the reviewer's concern, and we demonstrate this now using ground truth model simulated data (see below).

Reviewer 1-3: "Related to this point, given that this study is primarily methodological, I was expecting to see an evaluation of the method based on some type of synthetic ground truth data next to the involved analysis of experimental data in the Results section. While there is an evaluation of how well the individual models fit in that data, it remains unclear in how far the method can actually distinguish between these models if they are implemented by synthetic data. This would help the reader in a number of ways: (i) clarify the data scenarios that the method is intended for, (ii) assess the degree to which the method can distinguish between H1-H7, (iii) assess the approach of significance testing and calibrate the method with respect to number of spikes required, number of surrogates required, and effects of the observation time window." To address this concern, we have added a new section to the manuscript in which we simulate various sorts of causal or null spike-signal relationships with different types of controls (e.g., white noise, white noise with a consistent spike-triggered impulse response, white noise of higher amplitude gated and added by spikes, spikes driving and stabilizing signal toward a state) and evaluate the capability of the STA and the SDO (including matrix hypotheses H1-H7) to detect them, and the false negative and positive error rates. This serves three purposes:

1) We directly compare the sensitivity and specificity of the classical STA (i.e., no probability-based methods) against the SDO to detect significant spike-correlated effects on signal.

2) We validate the significance testing measures of the SDO method directly.

3) We identify cases where the STA is sufficient.

We have evaluated effects of both the number of spikes and shuffles/surrogates on the sensitivity and specificity of STA and SDO methods. While there are a considerable number of independent parameters which could be explored (number of states, pre/post spike intervals, signal transformations, filtering, etc.), our results indicate that the significance testing using prior-normalized SDOs from shuffled spike trains are generally more sensitive and specific than the classical STA alone.

Reviewer 2-1: "The big difference between a general stochastic controller and a biological stochastic controller is the manner in which information is moved around in the system.... that is using spikes. If there is a model mismatch between the theory and the biological application, this will invariably show up as spurious effects in the extracted kernels." Within the spike-triggered SDO (and in some instances, the STA), we do assume that changes in system dynamics (if any) are short-lived on the interval around spike. This does represent a departure from prior uses of the SDO as evoking a smooth change in state probability time evolution. If, however, a neuron spikes at sufficient rate to be adequately captured with a smooth rate parameter, the influence of spike-triggered effects may be reasonably modeled by a smooth scaling of the unit-associated SDO. Further work will be required to establish the performance of individual units' locally-estimated SDOs on predicting longer-scale behavior and dynamics, and when the neuron behavior significantly diverges from the Linear-Nonlinear-Poisson model, particularly when reverse-correlating spikes against stimuli. Instead, our objective here is to introduce a use-case for using the SDO on a local-scale to determine significance (or absence) of forward correlation of spike-signal relationships, as in one commonly used application of the STA.

Reviewer 2-2 "Applying SDO and STA to recorded data and demonstrating that they give different answers does not address this question. This paper needs an entirely new section where simulation data is generated from a well-defined control system (so that the answers are known) and it is then demonstrated that SDO arrives at correct answers where STA does not. The assumed model for the spiking of the neuron then plays a central role in how well the two techniques perform. If this is first demonstrated in simulation data, the technique can then be justifiably applied to recorded data." We thank the reviewer for calling attention to this important aspect of the work. In response, we have revised the introduction, methods, and discussion within the paper to further discuss use-cases of the SDO. More important, we have also included a section on tests with a range of different simulated spike-signal amplitude relationships (or absence) and stochastic processes, and show that there exist clear cases in simulation data where the SDO arrives at the correct answer (i.e., true positives, true negatives) while the forward-correlated STA does not, and there are clear differences in sensitivity with SDO outperforming STA.

We have also addressed minor comments listed by reviewers in the text.
