# Supplementary material for: A Stochastic Dynamic Operator Framework That Improves the Precision of Analysis and Prediction Relative to the Classical Spike-Triggered Average Method, Extending the Toolkit
Source: eNeuro. 2024 Nov 5;11(11):ENEURO.0512-23.2024. doi: 10.1523/ENEURO.0512-23.2024 (PMC11552545; doi:10.1523/ENEURO.0512-23.2024)
Supplement: SDO Analysis Toolkit — Download SDO Analysis Toolkit, ZIP file. [file eneuro-11-ENEURO.0512-23.2024-s001.zip › sdoAnalysis_20240821/sat-master/documentation/SDOAnalysisToolkitSOP.pdf]

SDO Analysis Toolkit

Standard Operating Procedures

Trevor S. Smith

Last Updated May 6, 2023

SDO Analysis Toolkit 1
Installing the SDO Analysis Toolkit: 4
Performing Custom SDO Analysis 11
Class-Method (OOP-Based) SDO Analysis: 11
Class Definitions: 13
xtDataCell() 13
ppDataCell() 15
pxtDataCell() 16
sdoMat() 18
sdoMultiMat() 20
Programmatic Functions 23
Generate and Populate Data holders ppData and xtData 23
Generate ppData: 23
SAT.ppDataHolder_new.m: 23
Populate ppData: 24
Generate xtData: 25
SAT.xtDataHolder_new.m: 25
Populate xtData: 26
Validate Data Structures 26
SAT.validateDataCells.m: 26
Compute SDO Matrix Structure 28
SAT.computeSDO.m 29
Screen for Significant Spike-Triggered SDOs 37
SAT.sdoUtils.findSigSdos.m: 37
Plot SDOs and Visualize Statistics 38
SAT.plotSDO.m: 38
Plot SDOs: 40
SAT.plot.plotDiffSDO.m: 40
SAT.plot.plotJointDiffSDO.m: 41
Plot Alternative SDO Visualizations: 43
SAT.plot.quiverSDO.m: 43
SAT.plot.shearSDO.m 44
Plot SDO Significance Statistics: 45
SAT.plot.risingFallingState.m: 45
SAT.plot.arraySig.m: 47
SAT.plot.px0Sig.m: 48
Use SDO to predict probability distributions of state 49
SAT.predictSDO.m 50
SAT.predict.predictPx.m: 52
Generate Predicted States from Distributions 54
pxTools.getXfromPx.m 54
Measure Prediction Errors Between Predicted and observed states and distributions 55
SAT.predict.calcPredictionError.m 55
Visualize Predicted Distributions and States 57
pxTools.plot.sta_v_x.m 57

pxTools.plot.px_v_x_waterfall.m: 59
pxTools.plot.staPxt.m 60
Plot State Predictions and Errors: 62
SAT.predict.plot.px_v_x.m 62
SAT.predict.plot.error_v_state.m 63
SAT.predict.plot.error_rates.m 64
SAT.predict.plot.relative_error_rates.m 65
SAT.predict.plot.pxDistance.m 66
SAT.predict.testSig.m 68
Supplemental Libraries for manipulating probability distributions and SDOs: 72
pxTools Library: 72
pxTools.getXtStateMap.m 72
pxTools.getPxtFromXt.m 73
pxTools.getXfromPx.m 74
pxTools.getTrialwisePxt.m: 75
pxTools.getCovArrFromJointArr.m 76
pxTools.getSdoFromJointArr.m 77
pxTools.getH0Array.m 77
pxTools.getMarkovFromXt.m 78
pxTools.KLDiv.m 78
pxTools.matrixRandomWalk.m 79
pxTools.splinePx.m 79
pxTools.predictPxtFromPx0.m 80
pxTools.getPxTE.m 80
pxTools.xvect2px.m 81
Auxiliary Functions: 81
SAT.sdoUtils.quicksdo.m 81
SAT.sdoUtils.islinearsdo.m 82
SAT.sdoUtils.normsdo.m 82
SAT.sdoUtils.conformsdo.m 83
SAT.estPxParams.m: 83
trimdatacell.m: 83
ssta_vs_sdo.m: 84

Installing the *SDO Analysis Toolkit*:

1) <u>Add the SDO Analysis toolkit to MATLAB Path</u>

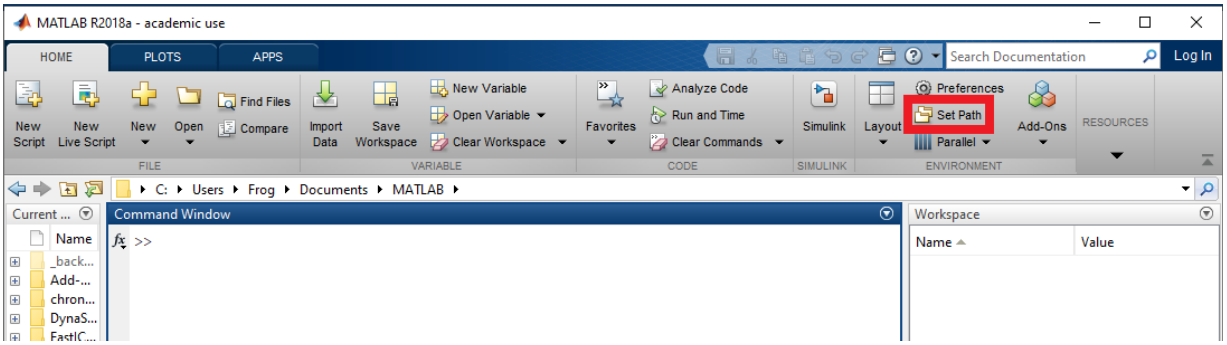

Open the ‘Set Path’ dialog in the MATLAB ‘HOME’ ribbon

This will pop up a user-interface window.

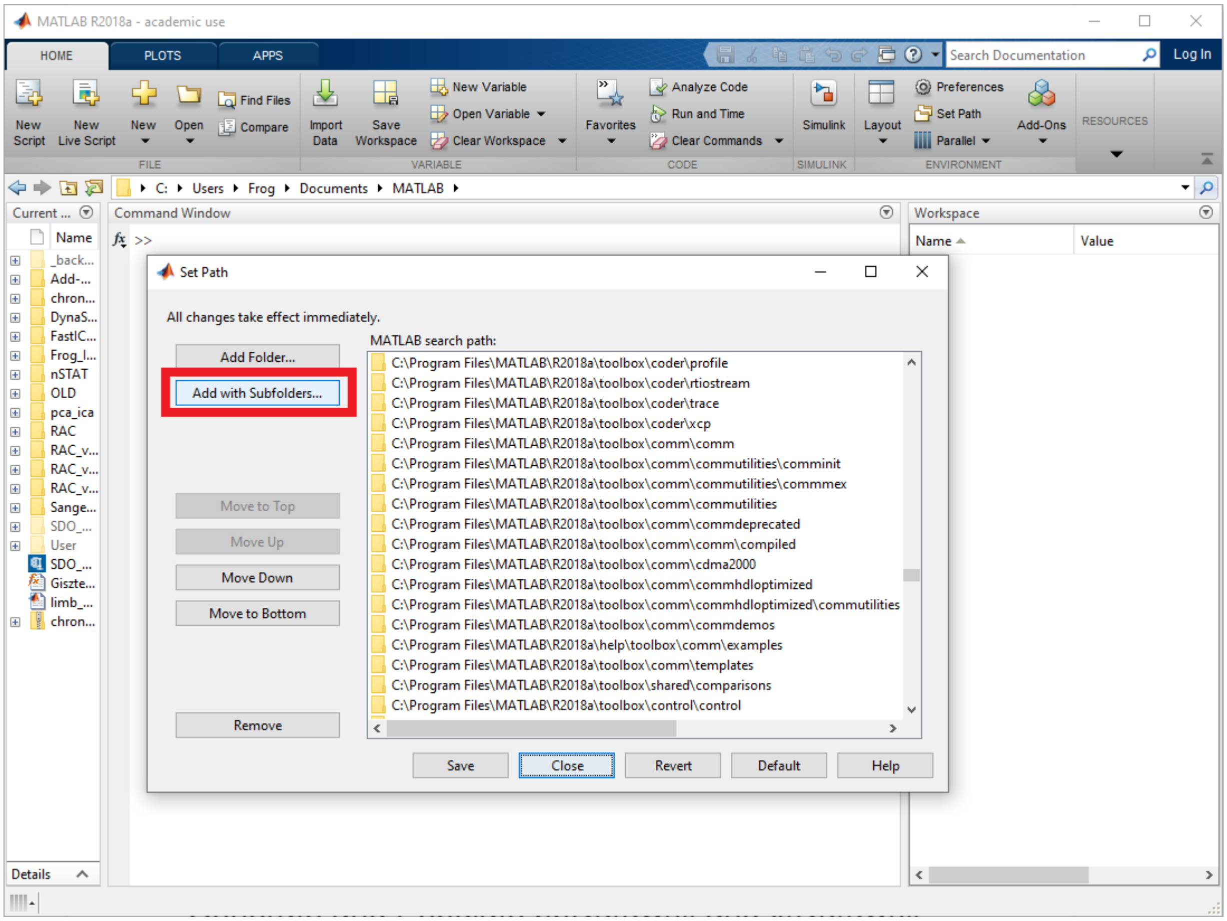

Click ‘Add with Subfolders’

A new window will pop up.

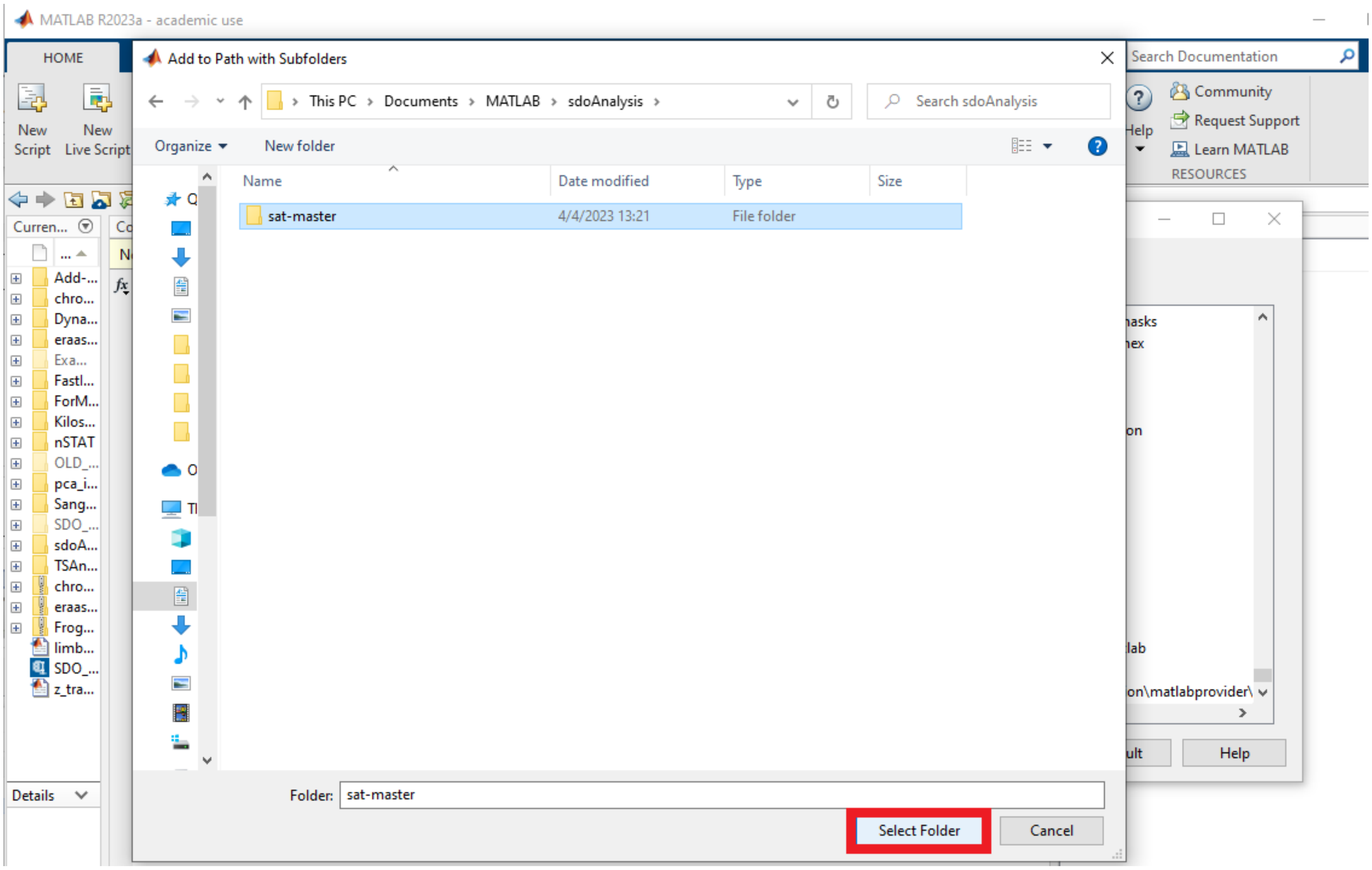

Navigate to the folder containing ‘sdoAnalysis’, from the downloaded repository of the *SDO Analysis Toolkit*. Click on the ‘sat-master’ folder to select it, and then click ‘Select Folder’ in the MATLAB user interface.

The pop-up will close, and return to the *‘Set Path’* window in the MATLAB user interface.

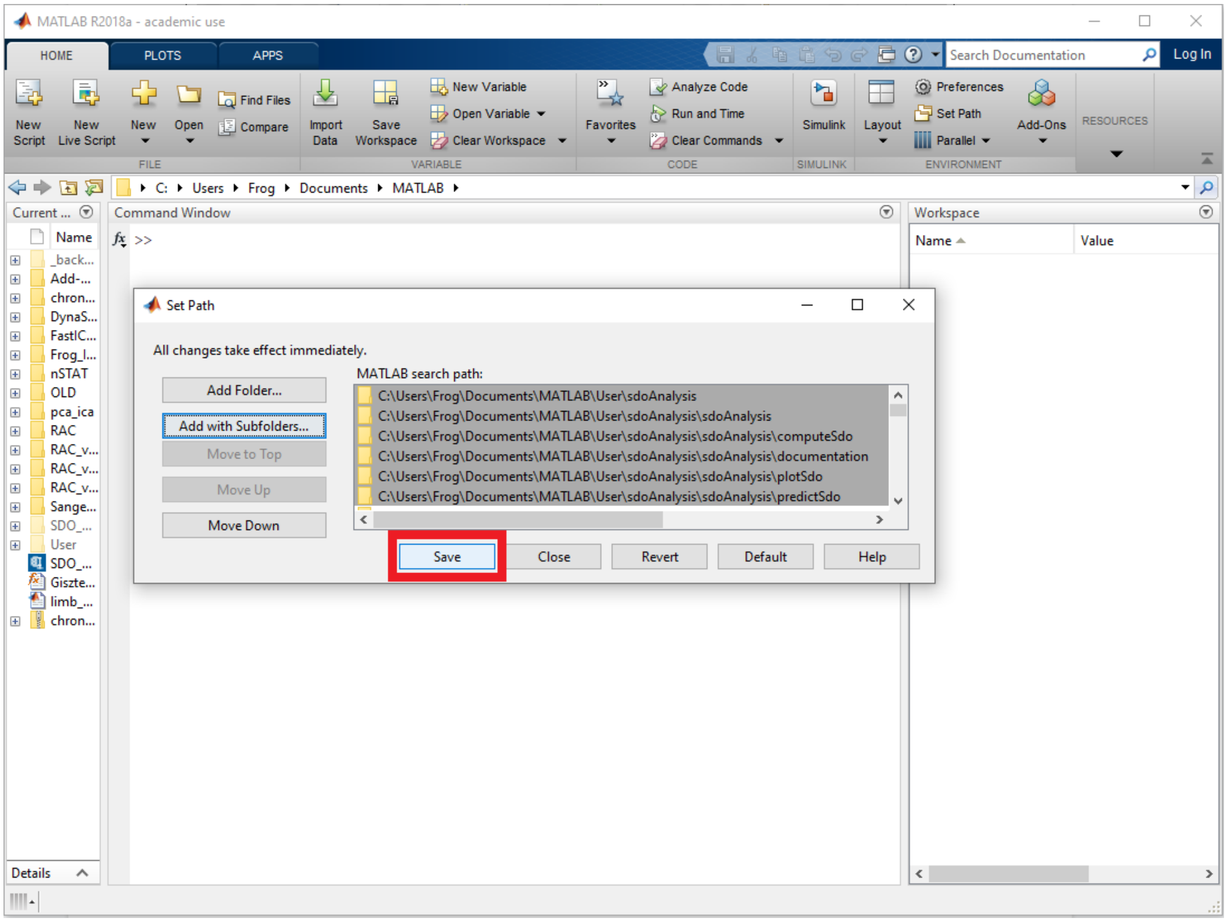

Save the changes to MATLAB path (if desired), for future MATLAB sessions.

The *'Set Path'* pop up window will close, and MATLAB will return to the command window.

2) Download Demonstration Data:

Data used in the demonstration of the *SDO Analysis Toolkit* may be downloaded in parallel. These include '*xtData.mat*' and '*ppData.mat*'.

An empty folder, 'demoData' is contained within the 'sdoAnalysis' directory. 'xtData.mat' and 'ppData.mat' may be placed here. (This is not necessary, because loading of this data is through a graphic user interface, but may be desirable as later screenshots assume this directory organization).

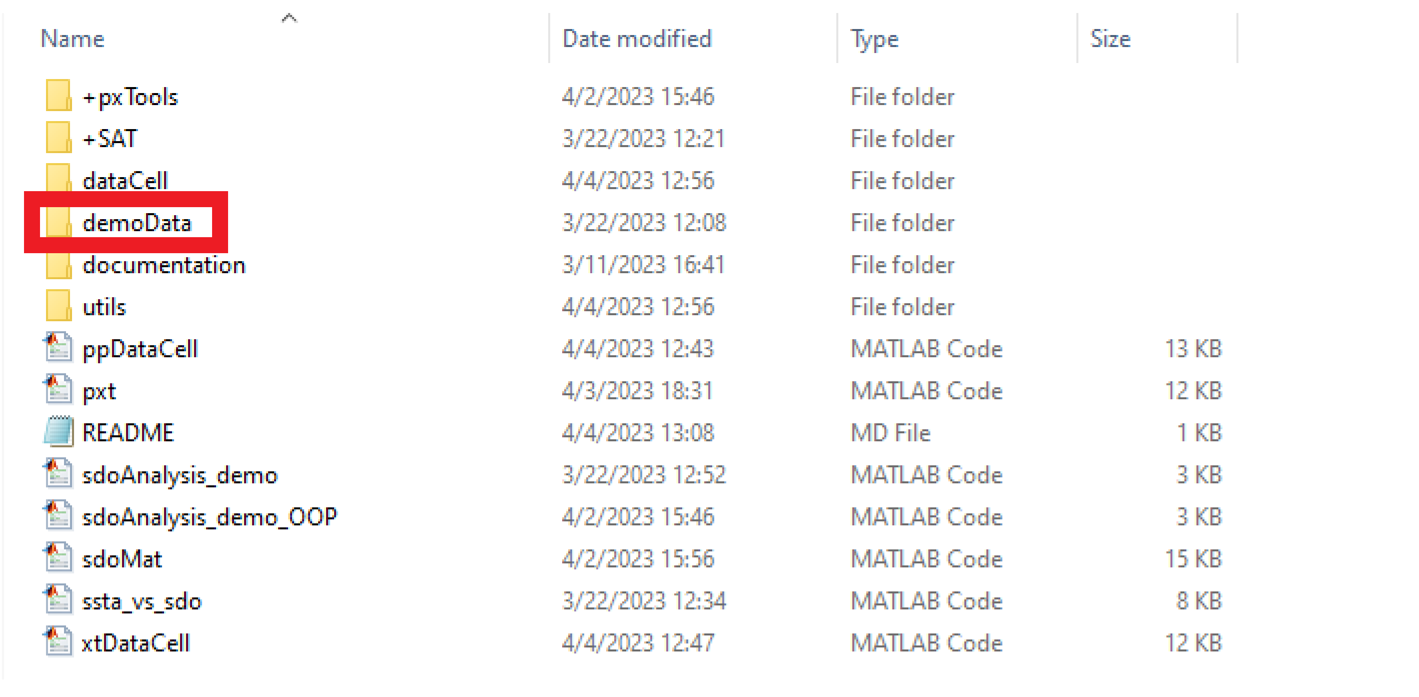

Demonstration data ('xtDataCell.mat' and 'ppDataCell.mat') may be placed into the empty directory '*demoData*' contained within the encompassing '*sdoAnalysis'* directory.

3) <u>Verify Installation and demonstrate the *SDO Analysis Toolkit*'s capabilities</u>:

Once added to MATLAB Path, the functions within the *SDO Analysis Toolkit* can be called from the command interface.

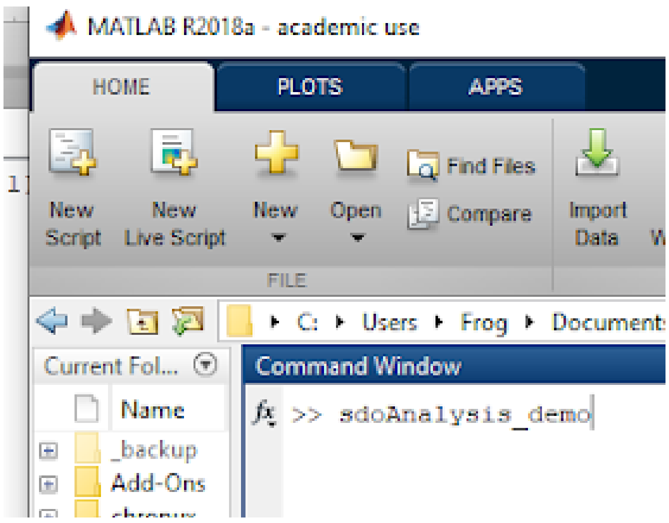

Type 'sdoAnalysis_demo' in the command window to call the demonstration script

A user interface window will pop up.

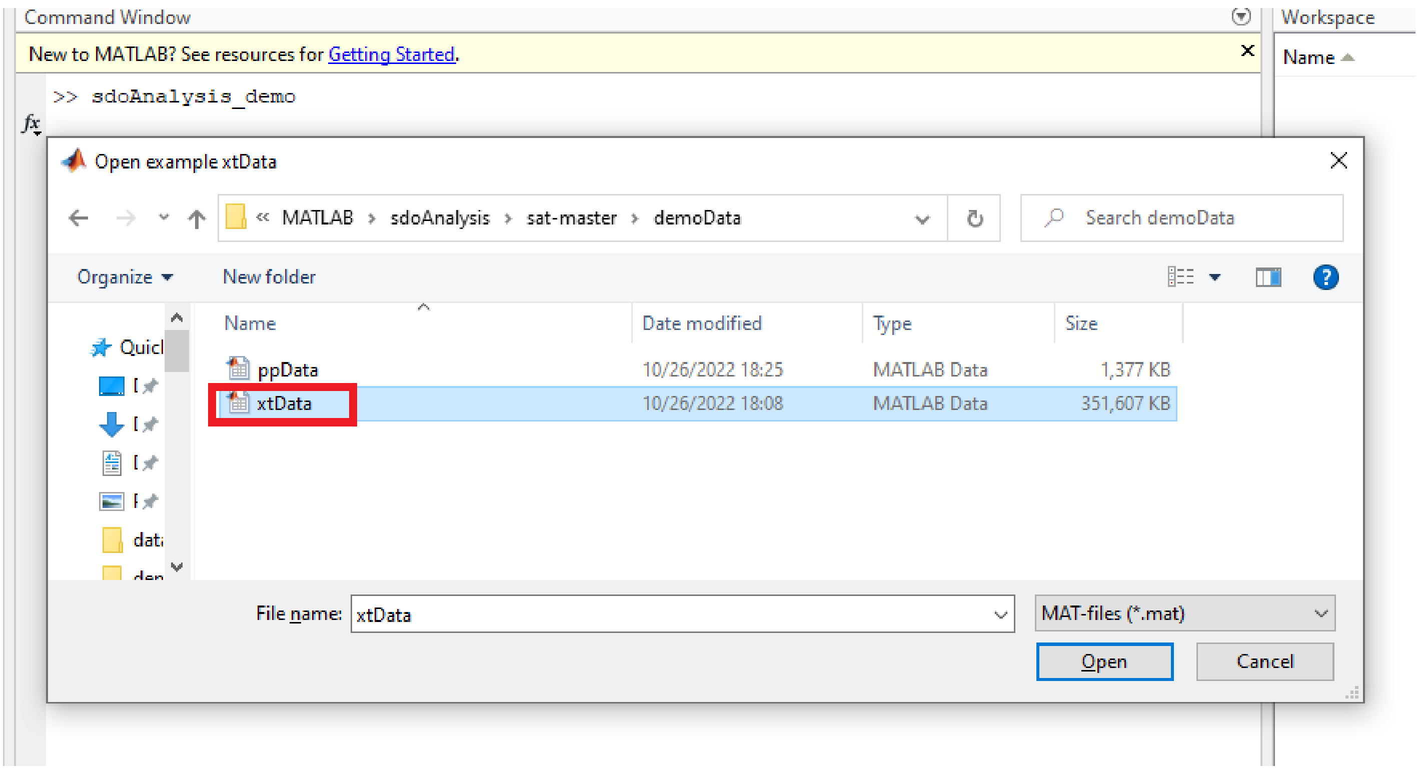

Navigate to the folder containing the included demonstration data and click ‘xtDataCell.mat’, and then ‘Open’ in the MATLAB user interface.

The interface window will close. After a moment, a second user interface window will pop up.

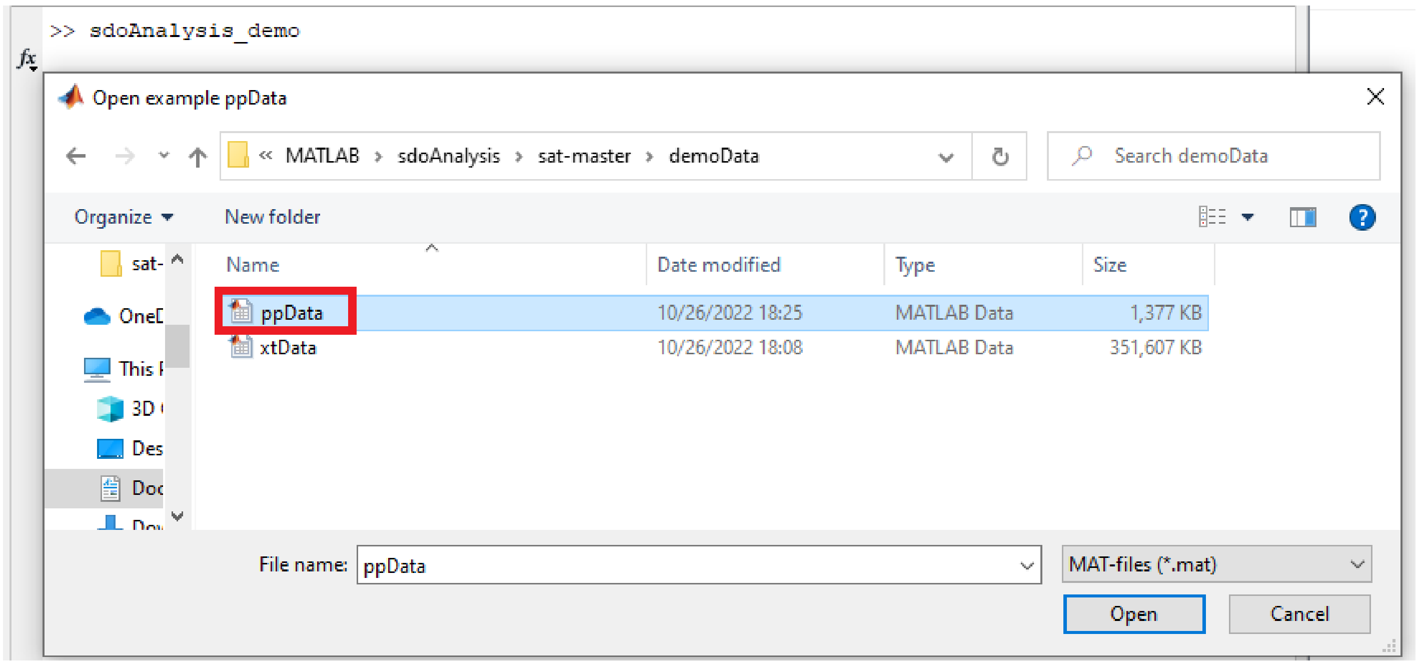

Navigate to the folder containing the included demonstration data and click ‘ppDataCell.mat’, and then ‘Open’ in the MATLAB user interface.

Note: If during the selection process, ‘Cancel’ is hit, or the wrong data is loaded, the command window will demonstrate an error. Run *sdoAnalysis_demo.m* again, and re-select the proper matfiles.

Once demonstration data has finished loading, *sdoAnalysis_demo*.m will automatically construct the SDO matrix structures, and plot a demonstrated spike-muscle pair. SDOs are constructed for every point process unit contained in *ppDataCell* (13) against every analog time series data channel contained in *xtDataCell* (11). An update message will be displayed after finishing each analog channel (i.e. 11 updates).

In our experience, using the provided demo data, each analog channel takes ~40-60 seconds to run (i.e. ~8-10 minutes to complete).

Once the SDOs are constructed, the time used for figure generation should be trivial (2-10 sec).

18 figures should be generated. The first figure should show three colored heatmaps, and should match the below image.

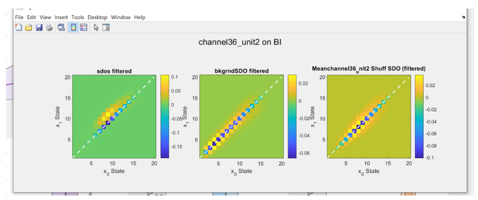

Figure 1, assuming a clean start in MATLAB.

Identification and interpretation of these plotted figures is included in the later documentation. Please refer to these sections for more information.

Successful completion of the *sdoAnalysis_demo* script should also display “COMPLETE!” in the MATLAB command window. This confirms that the *SDO Analysis Toolkit* has been installed successfully, and may be harnessed on custom data.

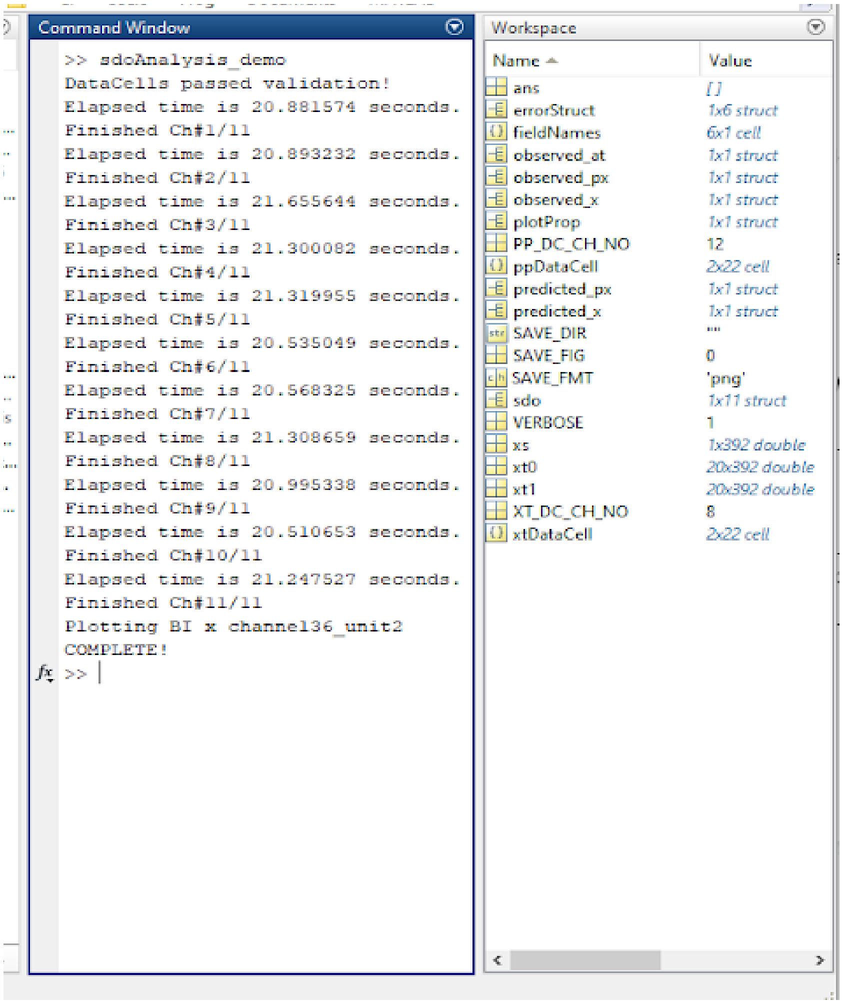

MATLAB command window and workspace variables after a successful execution of *sdoAnalysis_demo*.

If desired, alternative SDO analyses from the demonstration data may be visualized using the *plotSDO* script. (See also later documentation on the *plotSDO* module).

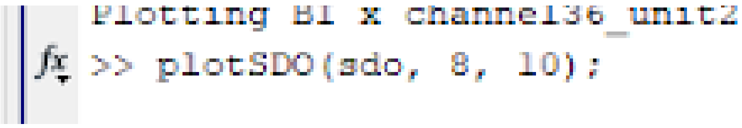

SDOs from other combinations of EMG and Neuron may be similarly plotted from the computed SDO using the SAT.*plotSDO* command.

Performing Custom SDO Analysis

The SDO Analysis Toolkit (SAT) contains both programmatic functions / scripts and class-method based implementations of the SDO. Custom data *classes* are initialized with default parameters, wrap the included functions into objects, from which functions may be called as *methods* of the object directly. This later 'object-oriented programming' (OOP) method allows for standardization of data structures and makes function calls more accessible and efficient (and provides 'guiderails' to minimize the chance of running into errors). Thus, the OOP-based methods are the preferred implementation of the *SDO Analysis Toolkit*. These methods require MATLAB v. 2019a or newer.

Nonetheless, it is important for an investigator to understand the parameters which are being called by the nested functions, to best optimize the parameters for their dataset. Advanced users who would like to reliably adapt the existing methods for their particular data sets may find direct calls to the function library to be better suited. Some standalone methods (such as drawing the waveforms of the simple STA) are not incorporated into the OOP classes. Thus, we present both methods here for convenience.

There is significant interconvertibility between structures generated by the stand-alone functions and the class methods. *xtData* and *ppData* data holder structures may be directly imported into the *xtDataCell* and *ppDataCell* classes as the 'data' field. Particular SDOs selected from the *sdo* data structure generated from the function-based methods may also be directly imported into the *sdoMat* class.

Class-Method (OOP-Based) SDO Analysis:

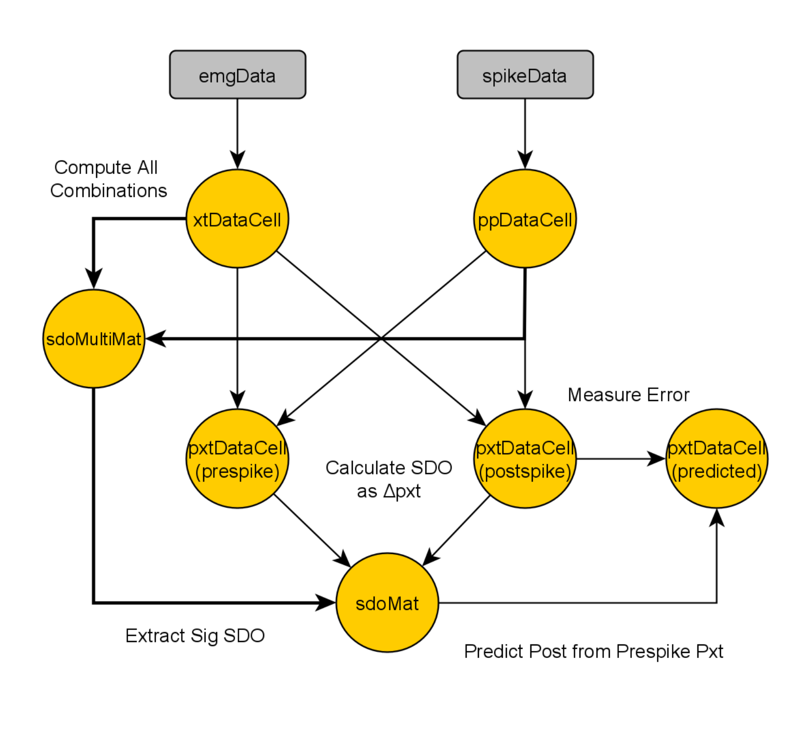

<u>Overview of Interactions of the OOP-based custom data classes</u>. Time series data is imported into *xtDataCell* and point-process data into *ppDataCell*. Probability distributions of state are

generated for the pre-spike and post-spike distribution using the *pxtDataCell* class. The difference between *pxtDataCell* class data is used to generate the *sdoMat* class. The *sdoMat* may then produce a predicted probability distribution *pxtDataCell* class. The difference between the predicted and observed *pxtDataCell* classes is the prediction error. A *sdoMultiMat* class may be used to compute SDOs within a selected range of units within *xtDataCell* and *ppDataCell*, using a common set of parameters, from which individual *sdoMat* objects may be extracted for further analysis.

Classes and Associated Methods

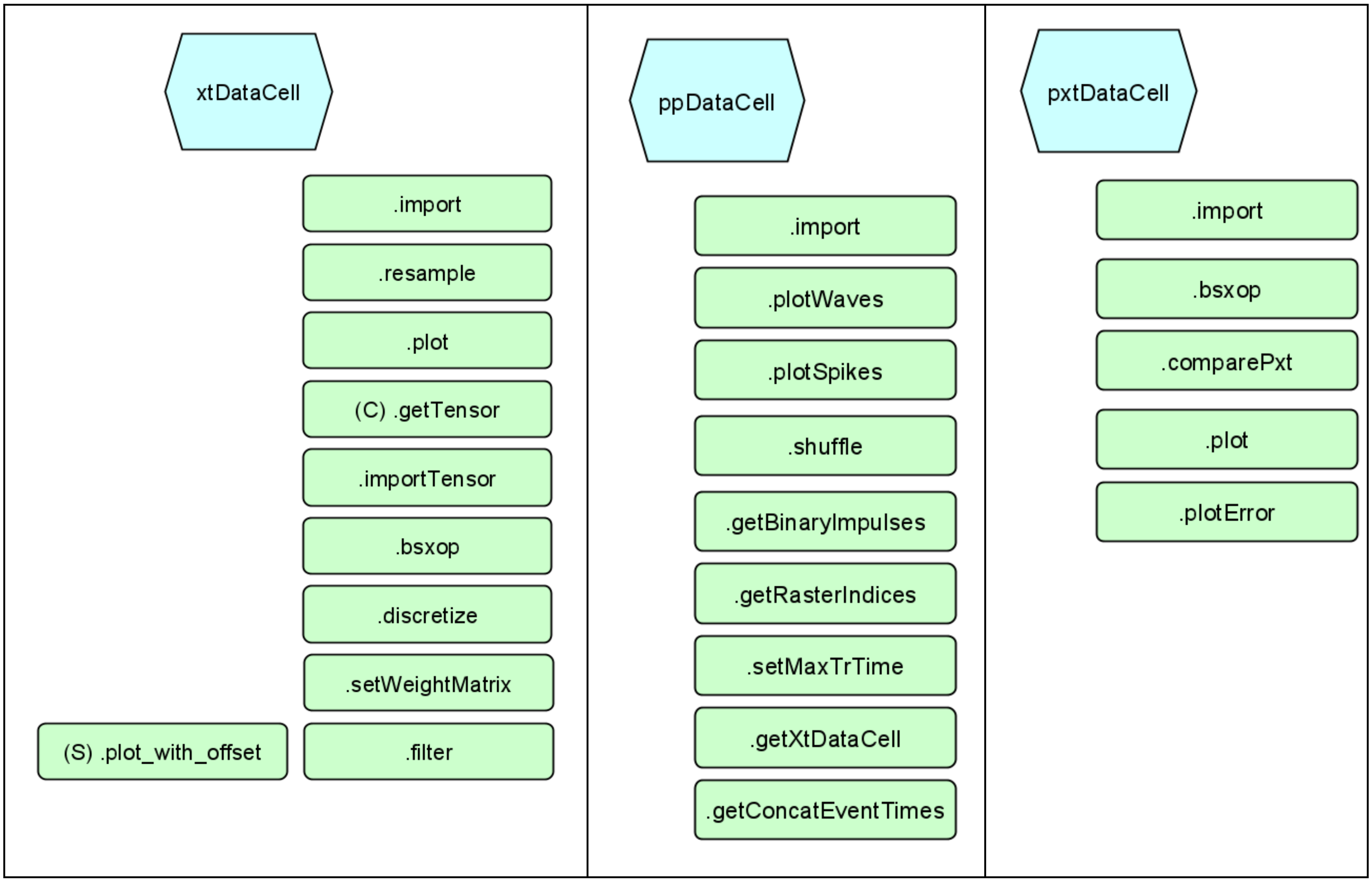

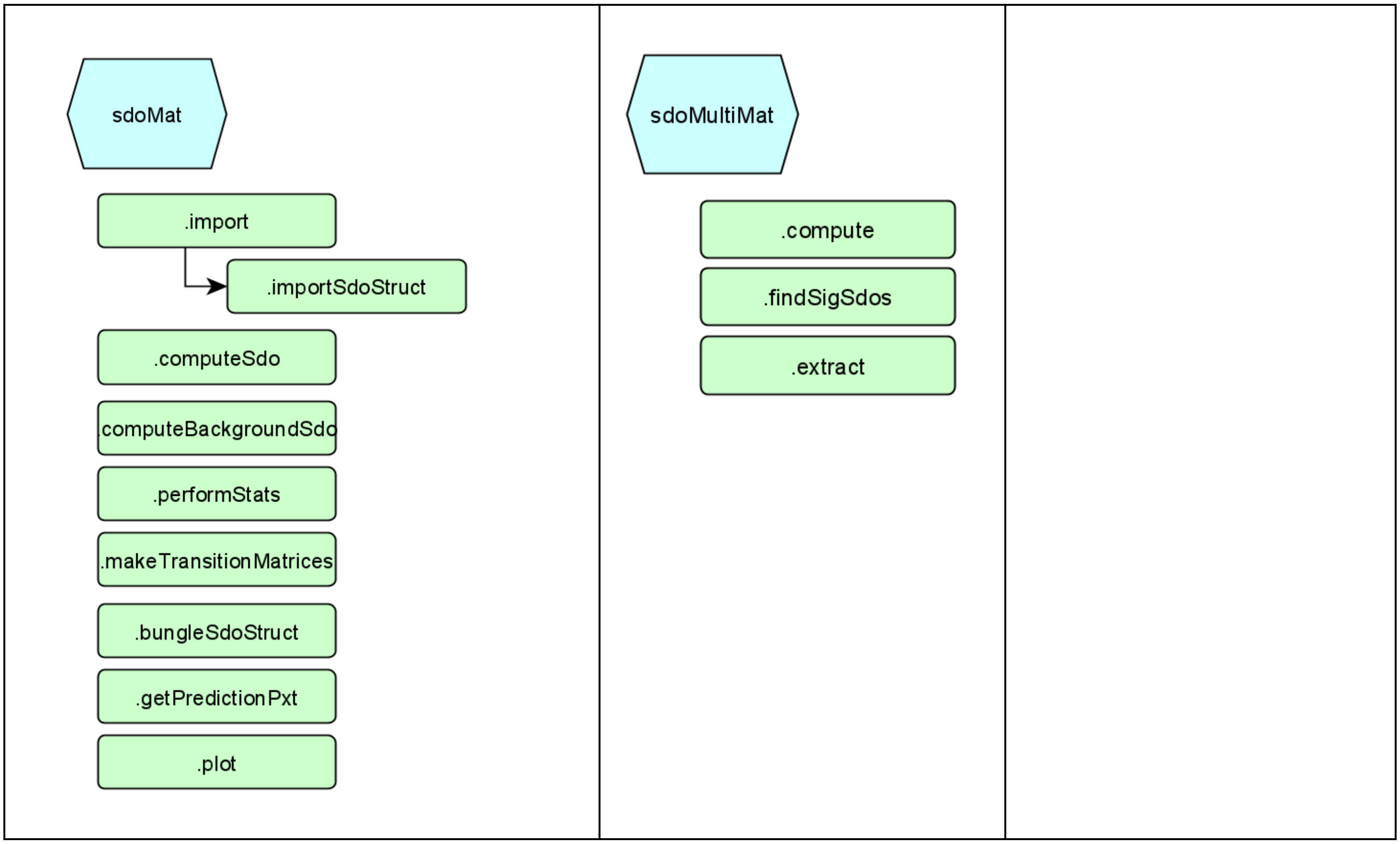

Class Definitions:

NOTE: All custom classes are *handle* classes (i.e. inherit properties from the MATLAB-defined *handle* class, https://www.mathworks.com/help/matlab/handle-classes.html). Briefly, handle classes operate on references *to* a data object (i.e. provide a 'handle' on), such that calls to and manipulations of, the object refer to the same, singular object. If *A = handleClass; B = A;* Then if *A = [ ] → B = [ ]*. If an investigator seeks a (deep)copy of a given data class (e.g. to separately manipulate), they must use the *.copy* method, else the reference variable used for the 'copy' (B, in example) will point to the same object.
NOTE: When calling a method which is part of a class, the method may be called by appending the suffix *.methodName(params)* (i.e. the name of the function/method) to the variable of that data class. Alternatively, these can be called as *methodNames(variableOfClassType, params)*.

---

xtDataCell()

Utility: Custom *Handle* class to Import, manipulate, and subsample trialwise time series [x(t)] data.
Properties:
- **.data**
  - {1 x N_TRIALS} cell containing trialwise timeseries data. See *SAT.xtDataHolder_new.m*
  - Populated by *.import* method.
- **.metadata**:
  - {1 x N_TRIALS} cell containing trialwise meta data. See *SAT.xtDataHolder_new.m*
- .**nTrials**:

- [Integer]. Number of columns in *data*.
- Populated by *.import* method.

- **.nChannels**:
    - [Integer]. Number of rows (data channels) for each recording trial.
    - Populated by *.import* method.
- **.electrode**:
    - {1 x N_CHANNELS} cell containing strings of channel names.
- **.fs**
    - Sample frequency of timeseries data.
    - Populated by *.import* method.
    - Modified by *.resample* method.
- .**trTimeLen**:
    - [1 x N_TRIALS] doubles array, corresponding to the length of each trial in *.data*.
    - Populated by *.import* method.
- **.dataField**:
    - [string]. Corresponds to fieldname in *.data* which contains time series data.
    - Populated by *.import* method.
- **.channelAmpMax**:
    - [N_CHANNELS x N_TRIALS] doubles array, corresponding to maximum observed value.
    - Populated by *.import* method.
- **.channelAmpMin**:
    - [N_CHANNELS x N_TRIALS] doubles array, corresponding to minimum observed value.
    - Populated by *.import* method.
- **.mapMethod**:
    - {'linear', 'log', 'linearsigned', 'logsigned'}. Method for defining state. See *SAT.computeSDO.m*
    - Default = 'log'
- **.maxMode**:
    - {'pTrial', 'xTrialxSeg'}. Method for determining trial min/max. See *SAT.computeSDO.m*
    - Default = 'xTrialxSeg'
- **.nBins**:
    - [Integer]. Number of states to define between min/max amplitude.
    - Default = 20

(Selected) Methods:

- **<u>.import</u>**(*xtData)*:
    - Imports *xtData*, as generated by *SAT.xtDataHolder_new.m*
- **<u>.resample</u>**(DESIRED_HZ, DATAFIELD):
    - Resample time series data in data.{}DATAFIELD to a given frequency (DESIRED_HZ)
    - *Defaults*:
        - *DESIRED_HZ* = obj.fs;
        - *DATAFIELD* = obj.dataField
- **<u>.bsxop</u>**(*xtDataCell*, funcHandle)
    - Perform bitwise operations between two *xtDataCells* of the same size (i.e. sum, multiply)
    - *funcHandle* is a standard MATLAB function handle.
- **<u>.plot</u>**(useTrials, useChannels, OFFSET, DATAFIELD):
    - Co-plot the appended time series data (or subset). Segregate traces by *OFFSET* or autodefine.
    - *Defaults*:
        - *useTrials* = 1:obj.nTrials;
        - *useChannels* = 1:obj.nChannels;
        - *OFFSET* = [];

- *DATAFIELD* = obj.dataField
- **.getTensor**(useChannels, useTrials)
  - Extracts a 3D data array (or subset of) from the *.data* field as a
    - (N_USED_CHANNELS, xtDataLen,N_USED_TRIALS) doubles tensor
  - *Defaults*:
    - *useChannels* = 1:obj.nChannels;
    - *useTrials* = 1:obj.nTrials;
- **.importTensor**(ten):
  - Imports a 3D data tensor (ten) to the *.data* field, in the same format as the *.getTensor* method.
- **.discretize**:
  - Assign state mapping to the xtData using set parameters.

---

ppDataCell()

Utility: Custom *Handle* class to import, manipulate, and subsample trialwise point process (pp) data.
Properties:

- **.data**
  - {1 x N_TRIALS} cell containing trialwise timeseries data. See *SAT.xtDataHolder_new.m*
  - Populated by *.import* method.
- **.metadata**:
  - {1 x N_TRIALS} cell containing trialwise meta data. See *SAT.xtDataHolder_new.m*
- .**nTrials**:
  - [Integer]. Number of columns in *data*.
  - Populated by *.import* method.
- **.nChannels**:
  - [Integer]. Number of rows (data channels) for each recording trial.
  - Populated by *.import* method.
- **.electrode**:
  - {1 x N_CHANNELS} cell containing strings of channel names.
- **.fs**
  - Sample frequency of timeseries data.
  - Populated by *.import* method.
  - Modified by *.resample* method.
- .**trTimeLen**:
  - [1 x N_TRIALS] doubles array, corresponding to the length of each trial in *.data*.
  - Populated by *.import* method.
- **.dataField**:
  - [string]. Corresponds to fieldname in *.data* which contains time series data.
  - Populated by *.import* method.
- **.nShuffles**:
  - [integer]. Number of shuffles to generate
  - Default = 1000
- **.shuffMethod**:
  - {‘isi/’cif’} Method for generating shuffle. See *SAT.computeSDO.m*
  - Default = ‘isi’
- **.shuffTau**:
  - Double. Time constant (in sec) for defining the conditional intensity function from point-process.

        - Default = 0.2
- **.shuffCIF**:
        - {'sg', '-hg', 'expd', 'tb'} Method for constructing the conditional intensity function.
        - See *SAT.computeSDO.m*
        - Default = '-hg'.

Methods:
- **.import**(*ppData*):
        - Imports *ppData*, as generated by *SAT.ppDataHolder_new.m*
- **.plotWaves**(useTrials, useRows):
        - Plots spike waveforms, in *.data*, if supplied.
- **.plotSpikes**(useTrials, useChannels):
        - Plots scatter of trialwise trains.
- **.shuffle**(useChannels, N_SHUFFLES, SHUFF_METHOD):
        - Generates shuffled spiketimes using defined shuffle methods.

---

pxtDataCell()

Utility: Custom *Handle* class to derive, manipulate, and measure difference between probability distributions measured at observed times.

Properties:
- **.xtName**:
        - *xtDataCell.dataField*
        - Populated by *.import*
- **.ppName**:
        - *ppDataCell.dataField*
        - Populated by *.import*
- **.xtChName**:
        - xtDataCell channel name of imported data.
        - Populated by *.import*
- **.ppChName**:
        - ppDataCell channel name of imported ata.
        - Populated by *.import*
- **.pxtNames**:
        - {1x nPxtTypes} cell of of *char*. Corresponds to type of prediction.
        - Populated by *.import*
        - Populated by *.comparePxt*
- **.nPxtTypes**:
        - [int]. Number of dataTypes/predictions contained within the *pxtDataCell*.
- **.nEvents**:
        - [int]. Number of events around which distributions are drawn.
- **.nShuffles**:
        - [int]. Number of shuffles generated in *pxt*.
        - Populated by *.import* from *ppDataCell*.
- **.xtProperties**:
        - Metadata of *xtDataCell*
- **.ppProperties**:

    - Metadata of *ppDataCell*
- **.duraMs**:
    - [double]. Duration of interval over which to derive event-wise distributions.
    - If *.duraMs* < 0, then sample this interval PRIOR to event.
    - If *.duratMs* > 0, then sample this interval AFTER to event.
    - Default = +10
- **.nShift**:
    - Shift in the the start time for pre/post spike interval. See *SAT.computeSDO.m*
    - Default = 1;
- **.zDelay**:
    - Interval between pre/post spike interval. See *SAT.computeSDO.m*
    - Default = 0;
- **.filterWid**:
    - Width of the smoothing filter, in states. See *SAT.computeSDO.m*
    - Default = 0;
- **.filterStd**:
    - Standard deviation of the smoothing filter, in states. See *SAT.computeSDO.m*
    - Default = 0;
- **.nStates**:
    - Number of states used to rasterize signal.
    - Populated by *.import* from *xtDataCell*.
- **.fs**:
    - [Double] Sample frequency
    - Populated by *.import* from *xtDataCell*.
- **.stateMapping**:
    - [1 x N_STATES+1] corresponding to the edges of the state bins.
    - Populated by *.import* from *xtDataCell*.
- **.backgroundPx**:
    - [N_STATES x 1] Probability distribution; average P(x)
    - Populated by *.import*
- **.backgroundMkv**:
    - [N_STATES x N_STATES] Markov Matrix, corresponding to background transitions (over duraMs).
    - Populated by *.import*
- **.markovMatrix**:
    - [N_STATES x N_STATES] Markov Matrix, corresponding to transitions over adjacent states.
    - Populated by *.import*
- **.data**:
    - {1 x nPxtTypes} cell containing [N_STATES x N_EVENTS] doubles cell, containing trialwise probability data.
- **.stateAssignment**:
    - {‘max’, ‘mean’, ‘median’} - Description of assigning single state to a distribution.
    - Default = ‘max’
- **.errorStruct**:
    - Error structure, containing the distance between a reference *pxtDataCell* and second *pxtDataCell*.
    - Populated by *.comparePxt*

Methods:
- **.import**(xtdc, ppdc, XT_CH_NO, PP_CH_NO):

- Populates *pxtDataCell* class using the provided *xtDataCell* (xtdc) and *ppDataCell* (ppdc) class, and provided channel/row indices (XT_CH_NO, PP_CH_NO).
    - Uses Properties in *pxtDataCell* to define classes.
- **.bsxop**(pxtdc, functionHandle, DATAFIELD):
    - Permits bitwise operations in the data between two *pxtDataCell* data classes.
    - *funcHandle* is a standard MATLAB function Handle.
- **.comparePxt**(px1):
    - Compares the differences between this *pxtDataCell* class object and a second *pxtDataCell* class.
    - Populates the *errorStruct* field of reference *pxtDataCell*.
- .**plot**():
    - Plots the probability data using the *imagesc* plotter.
- .**plotError**():
    - Plots the error metrics calculated between the reference *pxtDataCell*and measured *pxtDataCell*.

---

sdoMat()

Properties:
- **.xtName**:
    - *xtDataCell.dataField*
    - Populated by *.import*
- **.ppName**:
    - *ppDataCell.dataField*
    - Populated by *.import*
- **.xtChName**:
    - xtDataCell channel name of imported data.
    - Populated by *.import*
- **.ppChName**:
    - ppDataCell channel name of imported ata.
    - Populated by *.import*
- **.pxtNames**:
    - {1x nPxtTypes} cell of of *char*. Corresponds to type of prediction.
    - Populated by *.import*
    - Populated by *.comparePxt*
- **.nPxtTypes**:
    - [int]. Number of dataTypes/predictions contained within the *pxtDataCell*.
- **.nEvents**:
    - [int]. Number of events around which distributions are drawn.
- **.nShuffles**:
    - [int]. Number of shuffles generated in *pxtDataCell*.
    - Populated by *.import* from *ppDataCell*.
- **.xtProperties**:
    - Metadata of *xtDataCell*
- **.ppProperties**:
    - Metadata of *ppDataCell*
- **.pxProperties**:

    - Metadata of *pxtDataCell*
- **.stateMapping**:
    - [1 x N_STATES+1] corresponding to the edges of the state bins.
    - Populated by *.import* from *pxtDataCell*
- **.px0_duraMs**:
    - [double]. Duration of intervals sampled in the prespike event-wise distributions.
    - Populated by *.import* from *pxtDataCell* (0).
- **.px1_duraMs**:
    - [double]. Duration of intervals sampled in the postspike event-wise distributions.
    - Populated by *.import* from *pxtDataCell* (1).
- **.nShuffles**:
    - [int] Number of shuffles used.
    - Populated by *.import* from *pxtDataCell* (0)
- **.sigPVal**:
    - [double] P-value for significance.
    - Default = 0.05;
- **.zScore**:
    - [boolean]. Whether to z-score (normalize) distributions of test-statistic prior to determining significance.
    - Default = false
- **.sdo**:
    - [N_STATES, N_STATES] SDO (differential) matrix corresponding to unit x xtData channel
    - Populated by *.import*
- **.sdoJoint**:
    - [N_STATES, N_STATES] SDO (joint) matrix corresponding to unit x xtData channel
    - Populated by *.import*
- **.sdoBkgrnd**:
    - [N_STATES, N_STATES] SDO (differential) matrix corresponding to the background xtDataChannel
    - Populated by *.import*
- **.sdoBkgrndJoint**:
    - [N_STATES, N_STATES] SDO (differential) matrix corresponding to the background xtDataChannel
    - Populated by *.import*
- **.shuffles**:
    - Populated by *.import*
- **.stats**:
    - Populated by *.import*
- **.markovMatrix**:
    - Populated by *.import* from *pxtDataCell* (0)
- **.transitionMat**:
    - Populated by *.makeTransitionMatrices*
- **markovType**:
    - {‘px0’/’px1’} Whether to use the Markov Matrix from *pxtDataCell* (0) or *pxtDataCell* (1).

(Selected) Methods:
- **.import**():
    - If used with *sdoStruct*, rebungles the struct data into *sdoMat* format.
    - If used with to import 2 *pxtDataCell* classes; the SDO is calculated as the difference between them.

- **.computeSdo**(pxt_0, pxt_1):
    - Used to populate *sdoMat* from two *pxtDataCell* structures (pxt_0, pxt_1).
- **.performStats**():
    - Calculates matrix statistic values using the *obj.sigPVal* and *obj.zScore* properties.
    - Populates the *obj.stats* field.
- **.makeTransitionMatrices**():
    - Generates the 7 Transition matrices, using the SDO structure.
- **.getPredictionPxt**():
    - Uses the transition matrices in the *sdoMat* class to generate a *pxtDataCell* (predicted) class.
- **.plot**():
    - Plots the SDO visualization methods, as described in *SAT.plotSDO.m*

---

sdoMultiMat()

Utility: Custom *Handle* class used to compute SDOs across numerous point process and timeseries data combinations using a common set of parameters.

Properties:

- **.nXtChannels**:
    - [int] Number of xtChannels used from *xtDataCell*
    - Populated by *.compute*
- **.nPpChannels**:
    - [int] Number of ppChannels used from *ppDataCell*
    - Populated by *.compute*
- **.px0DuraMs**:
    - *As described in **sdoMat**.*
- **.px1DuraMs**:
    - *As described in **sdoMat**.*
- **.zDelay**:
    - *As described in **pxtDataCell**.*
- **.nShift**:
    - *As described in **pxtDataCell**.*
- **.filterWid**:
    - *As described in **pxtDataCell**.*
- **.filterStd**:
    - *As described in **pxtDataCell**.*
- **.sigPVal**:
    - *As described in **sdoMat**.*
- **.nSigValues**:
    - Threshold for number of significant sdo matrix tests to flag for follow-up
- **.zScore**:
    - *As described in **sdoMat**.*
- **.sigMat**:
    - Structure which contains SDO combinations were found to be significant (using *.sigPVal*) on >= *.nSigValues* tests.
- **.sdoMatCell**:
    - Holder for *sdoMat* classes.
- **.sdoStruct**:

        - Holder for the *sdoStruct* data. See SAT.*computeSDO.m*
- **.xtProperties**:
    - Metadata from *xtDataCell*.
- **.ppProperties**:
    - Metadata from *ppDataCell*.

Methods:

- **.compute**(xtdc, ppdc, useXtChannels, usePpChannels):
    - Populates *sdoMultiMat* using an *xtDataCell* and *ppDataCell*.
    - If *useXtChannels* is provided, only use provided subset of channels from *xtDataCell*.
    - If *usePpChannels* is provided, only use provided subset of channels from *ppDataCell*.
- **.findSigSdos**():
    - Populates .*sigMat* using the number of provided tests.
- **.extract**(XT_CH_NO, PP_CH_NO):
    - Extracts an *sdoMat* class from the *sdoMultiMat*, as indexed within the reference *sdoMultiMat* as provided by XT_CH_NO, PP_CH_NO.

These are the steps taken by *sdoAnalysis_demo_OOP*.m

OOP Steps:

1. Generate an *xtDataCell* from emgCell.m
    a.
    ```
    $ xtdc = xtDataCell;
    $ xtdc.import(emgCell); %original xtData format;
    ```
2. Assign *xtDataCell* state-mapping parameters, if necessary;
    a.
    ```
    $ xtdc.discretize()
    ```
    b. Qualitatively validate discretization schema.
    ```
    $ xtdc.plot(1:22, 1:11, [], 'stateSignal');
    %// plot trials 1-22, rows 1-11, using the ‘stateSignal’
    ```
    c. → Adjust parameters as necessary until discretized data captures phenomenon of interest (i.e. state definitions are sufficient to describe physiological signal)
3. Generate a *ppDataCell* class, and import from spikeTimeCell.m
    a.
    ```
    $ ppdc = ppDataCell;
    $ ppdc.import(spikeTimeCell); %original ppData format
    ```
4. Shuffle spike occurrences in *ppDataCell*
    a.
    ```
    $ ppdc = ppdc.shuffle
    ```
5. Generate a *pxtDataCell*, to capture the *pre-spike* interval.
    a. Assign the properties as desired
    b. Sample a particular xt and pp channel

```
$ XT_CH_NO = 8;  %// BI
$ PP_CH_NO = 12; %// Ch36u2

$ px0 = pxtDataCell();
$ px0.duraMs = -10; %// assign negative here to use prespike Interval
$ px0.import(xtdc, ppdc, XT_CH_NO, PP_CH_NO);
```

6. Generate a *pxtDataCell*, to capture the *post-spike* interval.
   a. Sample the same xt and pp channel as above

```
$ px1 = pxtDataCell(); %post-spike interval;
$ px1.import(xtdc, ppdc, XT_CH_NO, PP_CH_NO);
```

7. Compute SDO as the difference between the two distributions (*pxtDataCell* classes)

```
$ sdo_out = sdoMat();
$ sdo_out.computeSdo(px0, px1);
```

8. Compute Background SDO from the original *xtDataCell* using assignment properties in *sdoMat*.
   a. → This will replace the estimated background from the backgroundMkv of each *pxtDataCell* class
   b. Not necessary for prediction, but has improved accuracy.

```
$ sdo_out.computeBackgroundSdo(xtdc);
```

9. Assign SDO significance at an assigned P-Value Threshold (default = 0.05);

```
$ min_pval = 0.05;
$ sdo_out.performStats(min_pVal);
```

10. Plot SDO matrices
    a.

```
$ sdo_out.plot()
```

11. Generate Transition Matrices
    a. *Generate 7 Hypothesized transitions*

```
$ sdo_out.makeTransitionMatrices();
```

12. Generate a predicted *pxtDataCell* class from an initial *pxtDataCell*
    a.

```
%// px0 is the initial pxtDataCell (at time 0)
$ pdpxt = sdo_out.getPredictionPxt(px0)
```

13. Measure the distance between a predicted and observed *pxtDataCell*
    a.

```
$ pdpx.comparePxt(px1)
% // compare between the predicted px1 and observed px1
```

14. Plot the distance between *pxtDataCell* data classes;
    a.

```
$ pdpx.plotError
```

Note that multiple SDOs can be computed at once, using a single set of parameters, using the *sdoMultiMat* class.

NOTE: The SDO Analysis Toolkit is imported into MATLAB as a data package (SAT). Folders designated prefixed by '+' serve as sub-packages within the SAT Package. See MATLAB Documentation.

For example, the script "...\sat-master\+SAT\+predict\+plot.px_v_x.m", the appropriate function call within MATLAB is `SAT.predict.plot.px_v_x();`.

Programmatic Functions

Short Flow:

1) Generate *ppData* & *xtData*
2) *validate_dataCells*
3) *computeSDO.m*
4) *findSigSdos.m*
5) *plotSDO.m*
6) *predictSDO.m*

Generate and Populate Data holders *ppData* and *xtData*

Generate *ppData*:

SAT.ppDataHolder_new.m:

*SAT.ppDataHolder_new(**N_TRIALS, N_PP_CHANNELS**)*

Utility:
- Generates an empty *ppData* structure, a data holder for point process data.
- User data must be bungled to fit this format.

Prerequisites:
- None

Input Arguments:
- N_TRIALS
  - Integer value corresponding to the number of trials to use.
- N_PP_CHANNELS
  - Integer value corresponding to the number of point-process data channels to use.

Output:
- *ppData* of defined size.

Example Code

```
>> numberTrials = 20;
>> numberNeurons = 10;
ppData = SAT.ppDataHolder_new(numberTrials, numberNeurons);
```

ppData A {2 x N_TRIALS} Cell array containing point-process observations collected during recording trials. Each trial is represented as a column of the cell array. SDO Analysis assumes a level of stationarity to the signal; that different trials differ in time, but not in principle signal behavior.

- ***ppData*{1,:}** Each cell in the first row contains primary data within 1 x N_PP_CHANNELS struct. This struct contains the following fields:
    - **.electrode**: A string or character unique identifier corresponding to this point-process data channel.
    - **.time**: A [1 x N] (Row) Vector containing the event times for the given point-process. These time points should be in the same units and relative start time as the data in the *xtData* against which it will be compared.
    - **.counts**: An integer count of the number of elements in **.time**
- ***ppData*{2,:}**: Each cell in the second row contains metadata associated with each trial. This data will be passively appended to the *sdo* structure later, to keep track of associated pre-processing parameters associated with SDO generation. It is a structure composed of the following fields:
    - **.trialNumber**: [Integer]: A simple index of trial number by position. This value is not directly utilized by the *computeSDO* code, and may be overridden.

IMPORTANT NOTES:
- *ppData* is a homogeneous structure. During data population, the row index for each trial should be consistent across the entire data cell. (e.g. 'Neuron 3' on row 3 in trial 1 should also be on row 3 of trial 10).
- Events which do not occur during a trial should still be given a field (and 'time' field left empty) to thus maintain this homogenous data structure.
- Additional fields may be incorporated into the first row of *ppData* (primary data) array, however, these **will not** be used for SDO analysis, nor passed to the *sdo* structure.
- Additional fields may be incorporated into the second row of *ppData*(metadata) array. These fields **will** be passively passed to the *sdo* structure. This permits an investigator to pass trial-wise notes, annotation, and data pre-processing parameters into the *sdo* structure for later comparison or review.

Populate *ppData*:

- Due to the plurality of possible data formats and structures, a single method for populating *ppData* from user data structures is not defined. Users will need to write a solution which navigates this process. An example of one potential data format (cell array) into *ppData* is given below.

Example Code

```
%// load Nx1 cell array of spike times;
>> ourSpikeData = load('spikedata.mat');

>> numberTrials  = 20;
>> numberNeurons = 10;

for tr = 1:numberTrials
    for n = 1:numberNeurons
        %// populate ppData;
        neuronName = strcat('neuron', num2str(n));
        ppData{1,tr}(n).electrode = neuronName;
        ppData{1,tr}(n).time = ourSpikeData{n};
        ppData{1,tr}(n).counts = length(ppData{1,tr}(n).time);
    end
end
```

IMPORTANT NOTES:
- Ideally, sets of spike times passed to the **.time** field of the *ppData*field should be represented as a [1 x numberSpikes] row vector.

- The units of time used in the *ppData* should match those used in *xtData*.

Generate *xtData*:

SAT.xtDataHolder_new.m:

SAT.xtDataHolder_new(**N_TRIALS, N_XT_CHANNELS**)

Utility:
- Generates an empty *xtData* data structure.

Prerequisites:
- None

Input Arguments:
- N_TRIALS:
  - Integer value corresponding to the number of trials to use.
- N_XT_CHANNELS:
  - Integer value corresponding to the number of point-process data channels to use.

Output:
- *xtData* of defined size.

Example Code

```
>> numberTrials = 20;
>> numberNeurons = 10;
>> xtData = SAT.xtDataHolder_new(numberTrials, numberNeurons);
```

xtData: A {2 x N_TRIALS} Cell array containing analog time-series signals collected during recording trials. Each trial is represented as a column of the cell array. SDO Analysis assumes a level of stationarity to the signal; that different trials differ in time, but not in principle signal behavior.

- ***xtData*{1,:}** Each cell in the first row contains primary data within 1 x N_PP_CHANNELS struct. This struct contains the following fields:
  - **.electrode**: A string or character unique identifier corresponding to this time series data channel.
  - .**envelope**: A [1 x N] (Row) vector containing the time-varying amplitude signal (or representation of the signal). This is the primary data used for STA and SDO analysis.
  - **.fs**: The signal sampling frequency, used to align observations to time points.
  - **.times**: A [1 x N] (Row) Vector containing the matching time values, defined for each element of *.**envelope***. These time points should be in the same units and relative start time as the data in the *ppData* against which it will be compared.
- ***xtData*{2,:}**: Each cell in the second row contains metadata associated with each trial. This data will be appended to the *sdo* structure later, to keep track of associated pre-processing parameters associated with SDO generation. It is a structure composed of the following fields:
  - **.trialNumber**: [Integer]: A simple index of trial number by position. This value is not directly utilized by the *computeSDO* code, and may be overridden.

IMPORTANT NOTES:
- *xtData* is a homogeneous structure. During data population, the row index for each trial should be consistent across the entire data cell. (e.g. ‘signal 3’ on row 3 in trial 1 should also be on row 3 of trial 10).
- Events which do not occur during a trial should still be given a field (and ‘time’ field left empty) to thus maintain this homogenous data structure.

- Additional fields may be incorporated into the first row of *xtData* (primary data) array, however, these **will not** be used for SDO analysis, nor passed to the *sdo* structure.
- Additional fields may be incorporated into the second row of *xtData* (metadata) array. These fields **will** be passively passed to the *sdo* structure. This permits an investigator to pass trial-wise notes, annotation, and data pre-processing parameters into the *sdo* structure for later comparison or review.

Populate *xtData*:

- Due to the plurality of possible data formats and structures, a single method for populating *xtData* from user data structures is not defined. Users will need to write a solution which navigates this process. An example of one potential data format (structure array) into *xtData* is given below.

Example Code

```
%// load 1xN structure with fields 'signal', 'channelName', 'Hz'
>> ourEmgData = load('emgdata.mat');

>> numberTrials  = 20;
>> numberEmg = 8;

for tr = 1:numberTrials
    for m = 1:numberEmg
        %// populate xtData;
        emgName = ourEmgData(m).channelName;
        xtData{1,tr}(m).electrode = emgName;
        xtData{1,tr}(n).envelope  = abs(ourEmgData(m).signal);
        sigLen = length(xtData{1,tr}(m).envelope);
        fs = ourEmgData(m).Hz; %// signal frequency
        xtData{1,tr}(n).fs        = fs;
        %// Here, assume data signal is contiguous
        xtData{1,tr}(n).times     = [1/fs:1/fs:sigLen/fs]';
    end
end
```

IMPORTANT NOTES:

- Time series data passed to the **.envelope** field should be in a [1xN] (row vector) format.
- Time data contained in the **.times** field should be in a [1xN] (row vector) format.
- Currently, frequency data, **.fs**, is assumed to be constant over all trials and observations.

Validate Data Structures

The SDO analysis package has a limited capacity to validate and conform *xtData* and *ppData* structures before running *computeSDO.m*.

It may be worthwhile to save *xtData* and *ppData* after the validation stage, depending on the effort used in their creation..

SAT.validateDataCells.m:

SAT.validateDataCells(**xtData, ppData**, FIX_FLAG)

Utility:

- Scans *ppData* and *xtData* structures to ensure the structure arrays are homogeneous, and that all fields are populated.

Prerequisites:

- *SAT.ppDataHolder_new()*
- *SAT.xtDataHolder_new()*

Input Arguments:

- *xtData*:
    - Populated xtData structure
- *ppData*
    - Populated ppData structure
- FIX_FLAG
    - 1 or 0. If 1, attempt to conform data struct holders to permit *computeSDO.m* to execute, but this may result in errors.

Output:

- *xtData*:
    - The repaired *xtData* if FIX_FLAG = 1, else the original xtData Structure
- *ppData*:
    - The repaired *ppData* if FIX_FLAG = 1, else the original ppData Structure
- exitFlag
    - 0 = No Warnings or Errors Detected
    - 1 = Warnings: *computeSDO.m* may execute, but there may be inaccuracies in construction.
    - 2 = Critical Warnings. *computeSDO.m* will fail with unrepaired data structures; repaired data structures should be validated by user prior.

Example Code

Successful Validation

```
>> xtdc = xtData;
>> ppdc = ppData;
%// test validation without repair
>> SAT.validateDataCells(xtdc, ppdc);
DataCells passed validation!
```

Error Detection

```
>> xtdc = xtData;
%// remove terminal trial to mismatch number of observations between trials
>> xtdc2 = xtData(:,1:end-1);

>> ppdc = ppData;

%// trial lengths are not the same size
>> length(xtdc2) == length(ppdc)
ans =
    logical
    0

%// test validation without repair
>> SAT.validateDataCells(xtdc2, ppdc);
WARNING!: Mismatch in number of trials between structures
```

Limited Error Correction

```
>> xtdc = xtData;
%// remove terminal trial, to mismatch number of observations between trials
>> xtdc2 = xtData(:,1:end-1);
```

```
>> ppdc = ppData;

%// trial lengths are not the same size
>> length(xtdc2) == length(ppdc)
ans =
    logical
    0

>> FIX_FLAG = 1;

%// test validation without repair
>> [xtdc_out, ppdc_out] = SAT.validateDataCells(xtdc2, ppdc, FIX_FLAG);
WARNING!: Mismatch in number of trials between structures

%// trial lengths are now the same size;
>> length(xtdc_out) == length(pdc_out)
ans =
    Logical
    1

%// DataCells now pass validation
>> SAT.validateDataCells(xtdc_out, ppdc_out);
DataCells passed validation!
```

IMPORTANT NOTES:

- This script checks that the number of rows (channels) in each trial is consistent across the entire data Structure, and that each utilized field is populated. It will give a false positive to validation if the correct number of channels is passed to a trial, but these are in inconsistent order.
- If one data structure contains more trials than the other, dummy trial data will be appended to the end of the smaller data structure. Depending on which trials were missing on the smaller data structure, this may result in a mismatching of trial data in the *xtData* and *ppData*.
- Dummied data structure trials pull information from the 1st trial (cell) of *xtData* and *ppData*. This carries the implicit assumption that sampling frequency is consistent.
- Frequency data, **.fs**, must be consistent within a trial field.
    - There is currently no support for having trials at differing frequencies in different trials. This may result in errors or false-positive validations.
- If any warnings are detected, data structures should be corrected by the user.
- Vector data (e.g. ***.envelope***) which is present in a column vector will be transposed to a row vector without raising an error.

Compute SDO Matrix Structure

The primary SDO Generation script, given a *xtData* and *ppData*. Because extensive parameters may be tuned for SDO generation, the execution parameters are contained within the script header, and must be separately set there. Parameters used in computeSDO are appended to the *sdo* structure, along with metadata (row 2) of *xtData* and *ppData.*

computeSDO will display the amount of time necessary to complete on xtDataChannel, for all ppData units provided within the chunk. This may be used to estimate the expected duration for code execution.

The effects of different parameters on state distributions can be tested prior to running *computeSDO.m* by the utility script *sdoAnalysis_testPxParams.m*.

SAT.computeSDO.m

SAT.computeSDO()

Utility:
- Generates and populates the *sdo* structure.

Prerequisites:
- *xtData*
- *ppData*

Dependencies:
- pxTools Library

Input Arguments:

xtData (read from memory)
ppData (read from memory)
[Header Arguments]

Header Arguments:

xtDataCellName :
- A String/Char matching to a *xtData* structure loaded into memory/workspace. (This allows for a *xtData* to be named uniquely, but evaluated commonly).
- Default = 'xtDataCell'

ppDataCellName :
- A String/Char matching to a *ppDataCell* structure loaded into memory/workspace. (This allows for a *ppData* to be named uniquely, but evaluated commonly).
- Default = 'ppDataCell'

XT_DATA_FIELD :
- A String/Char matching the fieldname of the *xtData* trial structure (row 1 cell element) containing the time series data. If *xtData* is populated as above, this may be left as default.
- Default = 'envelope';

XT_ID_FIELD :
- A String/Char matching the fieldname of the *xtData* trial structure (row 1 cell element) containing the unique channel identifier (name/ID) for the time series data. If *xtData* is populated as above, this may be left as default.
- Default = 'electrode'

PP_ID_FIELD :
- A String/Char matching the fieldname of the *ppData* trial structure (row 1 cell element) containing the unique channel identifier (name/ID). If *ppData* is populated as above, this may be left as default.
- Default = 'electrode'

XT_MAP_METHOD :
- A String/Char matching a scripted method for determining the maximum value for state for *xtData* signals.
- Currently only 'xTrialxSeg' is supported.
    - ‘xTrialxSeg’: Use the maximum of signal amplitude as observed over all trials.

XT_MAP_MODE :
- A String/Char matching a scripted method for the derivation of signal states in *xtData* from signal amplitudes measured in *xtData*.
- May be one of the following values:

- ‘log’ :
    - States are defined from equal intervals of log-transformed signal amplitude. (Default)
- 'Linear':
    - States are defined from equal intervals of non-transformed signal amplitude

N_BINS :

- Integer. The number of signal bins/ states to break up signal amplitude into. The SDO is a square matrix of size (N_BINS x N_BINS). Increasing N_BINS increases memory consumption and decreases sampling per state transition from observed data. Decreasing N_BINS decreases resolution of SDO matrix.
    - Default = 20

PX0_DURA_MS:

- Double. Duration of pre-spike interval to draw from *xtData*. Should be an integer multiple of xtData sample period. (e.g. at a sample frequency of 2000Hz, the minimum duration is (1/2000*1000) 0.5 ms; Durations may be 0.5, 1, 1.5 …)
- A pre-spike interval corresponding to a single bin will result in an SDO completely in accordance with Sanger, 2011 [1].
- Longer pre-spike intervals may be used for analysis and prediction, but resultant SDO matrices may violate assumptions of linearity.
    - Default = 0.5; (at our signal sample frequency of 2000Hz, 1 bin).

PX1_DURA_MS:

- Double. Duration of post-spike interval to draw from *xtData*. Should be an integer multiple of xtData sample period. (e.g. at a sample frequency of 2000Hz, the minimum duration is (1/2000*1000) 0.5 ms; Durations may be 0.5, 1, 1.5 …)
    - Default = 10; (at our signal sample frequency of 2000Hz, 20 bins)
- Post-spike intervals have no limitations on their size to satisfy assumptions for SDO linearity.
- If pres-spike and post-spike interval durations are of 1 bin, then the joint and differential SDO matrices will comprise a first order Markov matrix and change-of-Markov matrix.

PX_FSM_WID:

- Integer. Width of the exponential smoothing kernel (numerator) of drawn probability distributions. Effectively, the number of states over which to distribute a pulse bidirectionally. (Check the below notes for the equation used.)
    - Default = 1
- If PX_FSM_WID = 0, no smoothing;

PX_FSM_STD:

- Double: Normalization for exponential smoothing kernel (denominator) of drawn probability distributions. Rate of exponential decay. The higher the value, the smaller the difference between the center of the pulse and the adjacent affected states, as designated by PX_FSM_WID. (Check the below notes for the equation used.)
    - Default = 1,
- If PX_FSM_STD = 0, no smoothing;

PX_NSHIFT:

- Integer: Bin position of the start of the px1 distribution relative to spike position. Horizontally translates the bins collected for px0 and px1 bins in time. (Equivalent to a delay of BOTH pre-spike and post-spike distributions relative to spike).
    - Default = 1;

PX_ZDELAY:

- Integer: Number of bins between the end of the collected pre-spike distribution and start of post-spike distribution.
- Default = 0;

N_SHUFF:
- Integer: Number of shuffles (random draws) of observed interspike intervals to generate for each neuron. Used to test the significance of the spike-triggered SDO against non-specific 'shuffled' SDOs. Increasing this value will increase memory consumption.
- Default = 1000;

SHUFF_METHOD:
- {'ISI'/'CIF'}: Method for resampling spiketimes for each unit to determine spike-triggered significance.
- ISI shuffles interspike intervals. (Test significance relative to number of spiking observations)
- CIF resamples from an estimated conditional intensity function (CIF). (Test significance of spike position relative to jitter).
- Default = 'ISI'

CIF_FIR _TYPE:
- {'sg', '-hg', 'expd', tb'}: Finite Impulse Response (FIR) of spike impulses for estimating the CIF, used in turn for reshuffling spike trains to test significance.
- 'sg' = Symmetrical gaussian: A gaussian distribution centered on spike impulses. Acausal.
- '-hg' = Negative half-gaussian: The negative/prespike half of a gaussian distribution originally centered on spike impulses. Anticausal.
- 'expd' = Exponential Decay: Spike response decays logarithmically in time. Causal.
- 'tb' = Trailing boxcar: Boxcar summing over future events. Anticausal.
- Only used if SHUFF_METHOD == 'CIF'
- Default = '-hg'

CIF_TAU_SEC:
- Double: Decay rate for the CIF_FIR_TYPE filter, in seconds.
- if CIF_FIR_TYPE = {'sg', '-hg'}; duration for 1 STD, in Sec.
- if CIF_FIR_TYPE = 'expd'; = 1/Lambda
- if CIF_FIR_TYPE = 'tb'; Duration for boxcar
- Default = 0.05 (50 ms).

SIG_PVAL:
- Double: P-Value Threshold to assign statistical significance.
    - Default = 0.05;

Z_TRANSFORM:
- Boolean: [0/1]
- If 1, apply z-score transform data elements prior to determining significance of spike-triggered effects.

MAX_PP_PER_CHUNK:
- Integer. Maximum number of ppData channels to populate *sdo* struct for at once. Reducing this number decreases memory consumption at the cost of increased computational efficiency. If memory issues occur, drop this value.
    - Default = 25;

MAX_XT_PER_CHUNK:
- Integer. Maximum number of xtData channels to populate *sdo* struct for at once. Reducing this number decreases memory consumption at the cost of increased computational efficiency. If memory issues occur, drop this value.
    - Default = 15;

REBUILD_ARRAY:
- Logical. Restart *sdo* chunkwise generation. If *computeSDO* fails to complete (due to an error of *xtData* or *ppData* structures, or a lack of memory resources),, when calculating the SDO chunkwise (e.g., number neurons > MAX_PP_PER_CHUNK), it will attempt to resume *sdo* struct generation from the last successful completed row. This permits the user to address the issue before resuming.

- REBUILD_ARRAY disables continuation after failure. Set to 1 if issues occur with resuming *computeSDO* after failure.
- Default = 0;

SAVE_SDO:
- Logical. After computing sdo matrix and running statistics, save the data structure. User will be queried for save directory.
- Default = 1;

STEPWISE_CALC:
- Logical. Estimate the SDO matrices using an algorithm for the stepwise (binary) representations of "prespike" and "postspike" state distributions. This estimation of the SDO ensures the resulting matrices are linearly compliant without ad-hoc conforming. Stepwise Calculation represents a ~3x increase in SDO runtime. Recommended to be set to 1.
- Default = 1

VERBOSE
- Logical. Display additional status update text to the UI during calculation.
- Default = 0

<u>Output</u>:
- *sdo* struct.

sdo: A [1 x N_XT_CHANNELS] consolidated SDO structure, containing all combinations of *xtData* v. *ppData* data types, shuffled null-hypothesis SDOs, and associated statistics. It is a structure containing the following fields:

- **.signalType**:
    - *xtData* ID name.
- **.neuronName**:
    - A {1 x N_XT_CHANNELS} cell containing the ID names for the *ppData* contents, in the same order as *ppData*.
- **.levels**:
    - The amplitude level bins for mapping state for the time series signal referenced in this row of the *sdo*.
    - Format is compatible with the MATLAB *discretize()* function; the number of signal bins (states) is one less than the number of levels.
- **.unit**:
    - Reserved field. Corresponds to the derivation of state (mapping method) from signal maximum and minimum amplitude.
- **.sdosJoint**:
    - A {1 x N_PP_CHANNELS} cell containing the joint SDO calculated for that xtChannel (*sdo* row) and ppChannel (cell number), calculated from pre-spike and post-spike distributions calculated from <u>observed spiking events</u>.
    - Each joint SDO is the average matrix product of the pre-spike and post-spike distributions, and is a matrix containing non-negative values. If each column is normalized to 1, the joint SDO matrix becomes a left transition matrix.
    - If post-multiplied by a column vector of probability of pre-spike state, the product will be a prediction of the conditional post-spike signal state.
- **.sdos**:
    - A {1 x N_PP_CHANNELS} cell containing the differential SDO calculated for that xtChannel (*sdo* row) and ppChannel (cell number), calculated from pre-spike and post-spike distributions calculated from <u>observed spiking events</u>.

- Each differential SDO is the matrix product of 1) the *difference* in post-spike & pre-spike distributions and 2) the pre-spike distribution.
- If the differential SDO is perfectly compliant with Sanger:
    - 1) Each column sums to 0
    - 2) The Main diagonal contains non-positive elements
    - 3) The Off-diagonal elements are non-negative
- If more than one bin is used in the pre-spike distributions, or if the distribution is filtered, assumptions 2 and 3 may be violated. Such an SDO may still be used to accurately predict the *change* of post-spike signal state.
- If post-multiplied by a column vector of probability of pre-spike state, the product will be a prediction of the *change* in conditional post-spike signal state.

- **.bkgrndJointSDO**:
    - A [N_BINS x N_BINS] array containing the background joint SDO, calculated from every discrete time point in *xtData* for the reference xtData channel.
    - Prior assumptions of the joint SDO hold, but are not specific to any ppData channel.
- **.bkgrndSDO**:
    - A [N_BINS x N_BINS] array containing the background differential SDO, calculated from every discrete time point in *xtData* for the reference xtData channel.
    - Prior assumptions of the differential SDO hold, but are not specific to any ppData channel.
- **.shuffles**:
    - A {1 x N_XT_CHANNELS} cell containing a struct. This struct contains fields:
        - **.SDOShuff**:
            - A [N_BINS x N_BINS x N_SHUFF] doubles array, contains the calculated differential SDO for every shuffled spike train of that particular channel in *ppData*.
        - **.SDOJointShuff**:
            - A [N_BINS x N_BINS x N_SHUFF] doubles array, contains the calculated joint SDO for every shuffled spike train of that particular channel in *ppData*.
- **.stats**:
    - A {1 x N_XT_CHANNELS} cell containing a struct. This struct contains fields:
        - **.changeMeasureContSDO**:
            - The column-wise difference in the above-diagonal and below-diagonal elements of the spike-triggered differential SDO matrix.
        - **.changeMeasureShuffContSDO**:
            - The column-wise difference in the above-diagonal and below-diagonal elements for each shuffled differential SDO matrix.
        - **.KL2D_neuron_bk**:
            - The KLD between the spike-triggered SDO and the background, summed over all matrix elements.
        - **.KL2D_neuron_bk**:
            - The KLD between each shuffled SDO and the background, summed over all matrix elements.
        - **.KLcurr_neuron_bk**:
            - The KLD between the spike-triggered and background pre-spike distributions.
        - **.KLcurr_shuff_bk**:
            - The KLD between each shuffled-spike and background pre-spike distributions.
        - **KLcond_neuron_bk**:
            - The KLD between the spike-triggered and background post-spike distributions.
        - **KLcond_shuff_bk**:
            - The KLD between each shuffled-spike and background post-spike distributions.

        - **KL2D_neuron_meanshuff**:
            - The KLD between the spike-triggered SDO and the mean of the spike-shuffled SDOs, summed over all matrix elements.
        - **KL2D_shuff_meanshuff**:
            - The KLD between each spike-shuffled SDO and the mean of the spike-shuffled SDOs, summed over all matrix elements.
        - **KLcurr_neuron_meanshuff**:
            - The KLD between the spike-triggered and mean-shuffled pre-spike distributions.
        - **KLcurr_shuff_meanshuff**:
            - The KLD between each shuffled and mean-shuffled pre-spike distributions.
        - **KLcond_neuron_meanshuff**:
            - The KLD between the spike-triggered and mean-shuffled post-spike distributions.
        - **KLcond_shuff_meanshuff**:
            - The KLD between each shuffled and mean-shuffled post-spike distributions.
- **.params**:
    - A [1 x 3] Struct of SDO generation parameters containing the following fields
        - **.xt**
            - A [1x1] struct containing time series data parameters, with the following fields:
                - **.xtDataCellName**:
                    - xtDataCellName
                - **.DataFieldname**:
                    - XT_DATA_FIELD
                - **.IDFieldName**
                    - XT_ID_FIELD
                - **.MapMethod**:
                    - XT_MAP_METHOD
                - **.MapMode**:
                    - XT_MAP_MODE
                - **.trialwiseMetatdata**:
                    - Row 2 of *xtDataCell*
        - **.pp**
            - A [1x1] struct containing point-process data parameters, with the following fields:
                - **.ppDataCellName**:
                    - ppDataCellName
                - **.IDFieldname**:
                    - PP_ID_FIELD
                - **.trialwiseMetadata**:
                    - Row 2 of *ppData*
        - **.px**
            - A [1x1] struct containing methods for drawing probability distributions, with the following fields:
                - **.px0DurationMs**:
                    - PX0_DURA_MS
                - **.px1DurationMs**:
                    - PX1_DURA_MS
                - **.smoothingFilterWidth**:
                    - PX_FSM_WID
                - **.smoothingFilterStd**:
                    - PX_FSM_STD

- **x1StartShift**:
    - PX_NSHIFT
- **x0x1Delay**:
    - PX_ZDELAY

Example Code

```
%// load processed 11-channel emg as a time series
>> xtData = load('emgCell.mat');
%// load spike times as a  point process
>> ppData = load('spikeTimeCell.mat');

%// run computeSDO with default parameters;
>> SAT.computeSDO
Elapsed time is 14.306293 seconds.
Elapsed time is 14.227698 seconds.
Elapsed time is 14.247260 seconds.
Elapsed time is 14.113447 seconds.
Elapsed time is 14.765843 seconds.
Elapsed time is 14.047517 seconds.
Elapsed time is 14.021728 seconds.
Elapsed time is 14.037978 seconds.
Elapsed time is 14.298157 seconds.
Elapsed time is 14.518542 seconds.
Elapsed time is 14.791165 seconds.

>>
```

IMPORTANT NOTES:

- Different parameters for state assignment can be tested with the utility script *SAT.testPxParams.m* prior to running SAT.*computeSDO*.
- There are four supported parameters for tuning intervals from which signal is sampled, relative to a time index (spike, shuffled-spike, or random point). The relationships between these parameters is diagrammed as:
    - PX0_DURA_MS is the width/interval of the prespike interval (indexed backwards from time of spike)
    - PX1_DURA_MS is the width/interval of the postspike interval (indexed forward from teh time of spike)
    - PX_ZDELAY is the duration between the end of the pre-spike interval and the start of the post-spike interval. (i.e. a delay between the measured effect and response)
    - PX_NSHIFT is a homogenous shift of the prespike/postspike intervals relative to the spiking event. (i.e. a delay between the spike and pre-spike baseline ; equivalent delay between the spike and the post-spike effect)

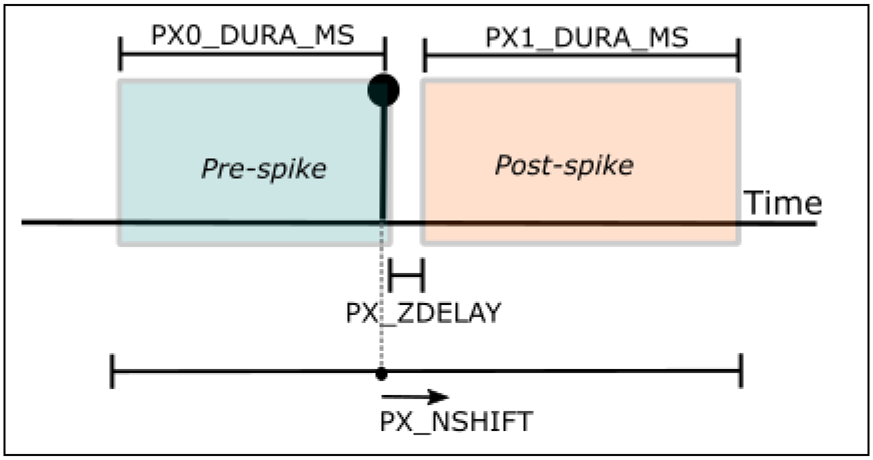

Relationships between variables governing the draws of the peri-spike state distributions.

- The if smoothing of perispike probability distributions is enabled (PX_FSM_WID > 1) and (PX_FSM_STD > 1), then filtering coefficients will be calculated for a zerophase (*filtfilt*) data filtering using an exponential smoothing kernel. Kernel weights are normalized to 1 to ensure that summed probability distributions also sum to 1.
  - $filter\ coefficients\ =\ exp(-\frac{[-wid:wid]^2}{2*std^2})$
    - 'wid' = PX_FSM_WID;
    - 'std' = PX_FSM_STD;
    -
- There are currently 4 supported methods of estimating the conditional intensity function from the spike events for shuffling spikes to evaluate the null hypothesis ('sg', '-hg', 'expd', 'tb'). The finite impulse responses (FIR) to spike impulses is diagrammed below:

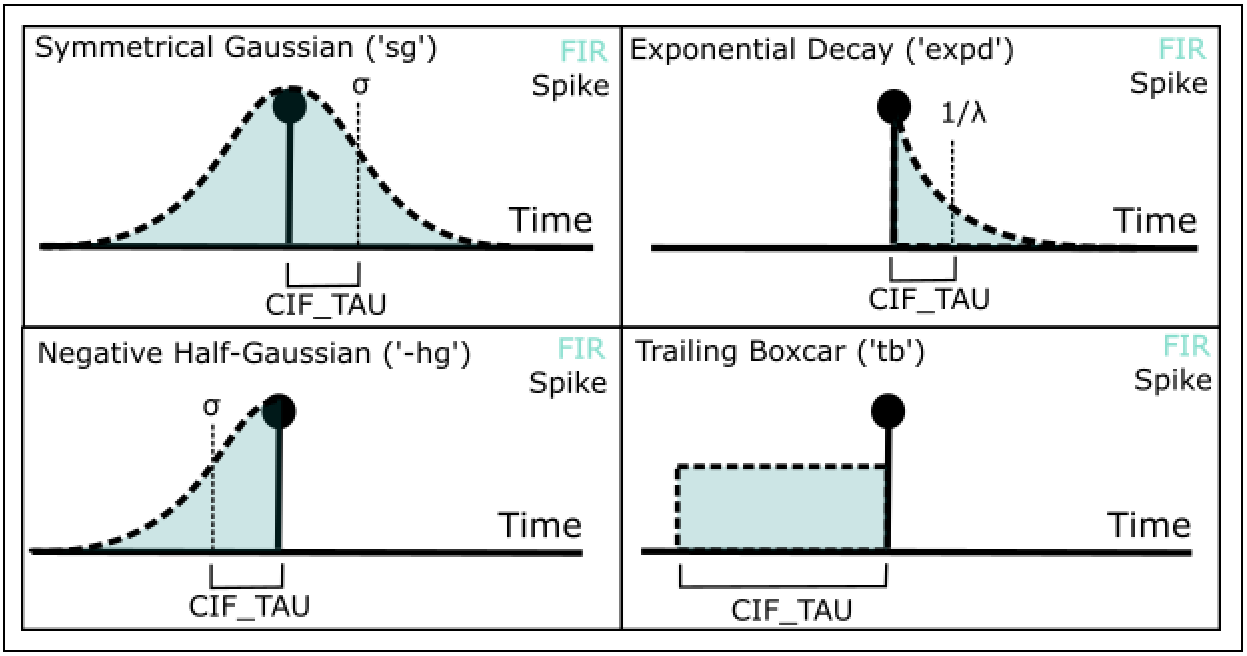

  - The support variable 'CIF_TAU' reflects the characteristic time decay of each response, provided in seconds.
  - The 'trailing boxcar' and 'negative half-gaussian' concentrate shuffle spikes in the pre-spike interval, testing a null hypothesis of no change in dynamics local to observed spike events.
  - The 'symmetrical gaussian' jitters observed spikes around their current interval, testing the specificity of spike effects in both directions.
- The *sdo* data structure may become quite large when querying over a large number of xtDataChannels and ppDataChannels. For reference, the *sdo* struct generated from the demo data, comprised of:
  - 11 xtChannels
  - 13 ppChannels
  - 20 States
  - 1000 Shuffles

  Corresponds to a ~950 mB *sdo* structure.
  - If the number of units tested becomes prohibitive, *sdo* may be generated and saved against a single time series dataset (i.e. one row of the *sdo* struct).

        - *sdo* ‘chunks’ when *computeSDO* operates ‘chunkwise’ may also be treated as miniaturized version of *sdo*. Significant SDOs may then be screened against these smaller *sdo* structures, and *ppData* and *xtData* trimmed to only include these combinations, if desired.

- Generation of the *sdo* struct, calculation of SDO statistics, and analysis of significance are handled as three separate steps (scripts) within *computeSDO*. Segregation of these steps may be optimal in some data exploration. For instance, the same *sdo* structure can be tested for significance at different P-Values without requiring it to be rebuilt by calling *computeSDO_testStatSig*.

Screen for Significant Spike-Triggered SDOs

Having built the *sdo* structure, identify SDOs which are statistically significant. Further SDO analysis requires honing in on a particular SDO, of which only a few may be present within all potential combinations of *xtData* and *ppData*.

SAT.sdoUtils.findSigSdos.m:

SAT.sdoUtils.findSigSdos(**sdo**, SIG_THRESH)

Utility:
- Identify spike-triggered SDOs which are at or above a chosen significance threshold.

Prerequisites:
- *computeSDO.m* or *computeSDO_performStats*.m

Input Arguments:
- *sdo*
    - The *sdo* structure containing the **.stats** field.
- SIG_THRESH
    - Integer [0-3]. Number of SDO features (at earlier established p-value) considered significant to screen SDO.

Output:
- *lookupStruct*
    - [1 x N] structure of screened SDOs containing fields:
        - **.xtChannelNo**:
            - Index number of xtChannel within *sdo* (sdo row index)
        - **.ppChannelNo**:
            - Index number of ppChannel within *sdo* (sdo(x).sdos row index)
        - **.xtChannelID**:
            - string/char. Fieldname/ID for xtChannel
        - **.ppChannelID**:
            - string/char. Fieldname/ID for ppChannel
        - **.nSig**:
            - Integer. Number of significant features of SDO.

Example Code:

```
%// load sdo
>> sdo = load('sdo.mat');

>> nXtChannels = length(sdo)

ans =
    11

>> nPpChannels = length(sdo(1).sdos)
```

```
ans =
     13

%// total number of SDOs
>> nXtChannels * nPpChannels

ans =

    143

%// only interested in sdos with 2+ significant features
>> sigThresh = 2;

%// Get reduced list
>> sigSdos = SAT.sdoUtils.findSigSdos(sdo, sigThresh);

%// Find number of sdos with 2+ significant features
>> length(sigSdos)

ans =

    56

%// find lookup information of first significant sdo
>> sigSdos(1)

ans =

    struct with fields:

        xtChannelNo: 1
        ppChannelNo: 6
        xtChannelID: 'AD'
        ppChannelID: 'channel20_unit2'
        nSig: 2
```

Plot SDOs and Visualize Statistics

Visualize the SDO matrix using a variety of methods. Unless a particular combination of xtDataChannel and ppDataChannel is of interest *a priori*, it may be helpful to first identify SDOs with statistically significant features, as above.

NOTE: If it is desired to save the generated plots as scalar vector graphics figures for later modification, we recommend setting the 'saveFig' flag to 1, rather than using a manual save in the MATLAB user interface. When using a manual save from the UI, MATLAB may automatically simplify .svg figures, which will limit customization of these plots in an svg program, such as Inskcape.

SAT.plotSDO.m:

SAT.plotSDO(**sdo**, **XT_SDO_CH_NO, PP_SDO_CH_NO,** saveFig, saveFormat, outputDirectory)

Utility:

- Header script for running the entire plotSDO library on the target *sdo*. This allows a single line of code for generating (and saving) figures. Similarly, if saving figures, this permits the user-variables for saving, save directory, and save format to only be set once.

Prerequisites:

- *computeSDO*.m

Input Arguments:

- *sdo*:
    - The *sdo* structure
- XT_SDO_CH_NO:
    - The row index of the *sdo* struct to use for SDO Analysis
    - Matches sdo(XT_SDO_CH_NO)
- PP_SDO_CH_NO:
    - The subindex of the *sdo* struct to use for SDO Analysis
    - Matches sdo(x).neuronNames{PP_SDO_CH_NO}
- **Optional Name-Value Pair Arguments**:
    - filter
        - Logical [0/1]
        - If 1, apply a smoothing filter along SDO matrix diagonals before plotting the displays.
        - Default = 1
    - saveFig:
        - Logical [0/1].
        - If 1, save plotted figure.
    - saveFormat:
        - String/Char [‘png’/’svg’]
        - File format to save figures, if saved.
    - outputDirectory:
        - String [“”]
        - Full file directory for saving figures to. If not passed and figures are saved, will query the user for a save directory.

Output:
- None
- (Figures are produced, and may be saved)

Example Code

Preview SDO Figures

```
%// load sdo
>> sdo = load('sdo.mat');

%// select indices for plotting
>> xt_index = 8;
>> pp_index = 4;

%// plot sdo figures without saving
>> SAT.plotSDO(sdo, xt_index, pp_index);
Plotting BI x channel17_unit3
```

Plot SDO figures to svg for later manipulation

```
%// load sdo
>> sdo = load('sdo.mat');

%// select indices for plotting
>> xt_index = 8;
>> pp_index = 4;

>> saveFigs = 1;
>> saveFormat = 'svg';
%// plot sdo figures without saving
>> plotSDO(sdo, xt_index, pp_index, 'saveFigs', saveFigs, 'saveFormat', saveFormat);
Plotting BI x channel17_unit3
```

CALLED FUNCTIONS:
- ***SAT.plot.plotDiffSDO()***
- ***SAT.plot.plotJointDiffSDO()***
- ***SAT.plot.risingFallingState()***
- ***SAT.plot.arraySig()***
- ***SAT.plot.px0Sig()***
- ***SAT.plot.quiverSDO()***
- ***SAT.plot.shearSDO()***

Plot SDOs:

SAT.plot.plotDiffSDO.m:

SAT.plotSdo.plotDiffSDO(**sdo, XT_CH_NO, PP_CH_NO,** saveFig, saveFormat, outputDirectory)

Utility:
- Plots the differential SDOs of the spike-triggered, background, and mean of the spike-shuffled sets of point process data as heatmaps.

Inputs:
- sdo
  - *sdo* structure generated by *computeSDO.m*
- XT_CH_NO
  - [integer]
  - XT channel ID number, row position, in *sdo*.
- PP_CH_NO
  - - [integer]
  - PP channel ID number, column subindex, for a given row of *sdo*.
- Optional Name-Value Pairs:
  - filter
    - Logical [0/1]
    - If 1, apply a smoothing filter on the SDO matrix diagonals before plotting.
    - Default = 1
  - saveFig
    - [0/1]
    - If 1, save generated figure to disk.
    - Default = 0
  - saveFormat
    - - ['png'/'svg']
    - File format for the saved file.
    - Default = ‘png’
  - outputDirectory
    - String containing path to the target save directory.
    - If not provided, and saveFig = 1, will query the user using a GUI.
    - Default = ‘’

Output:

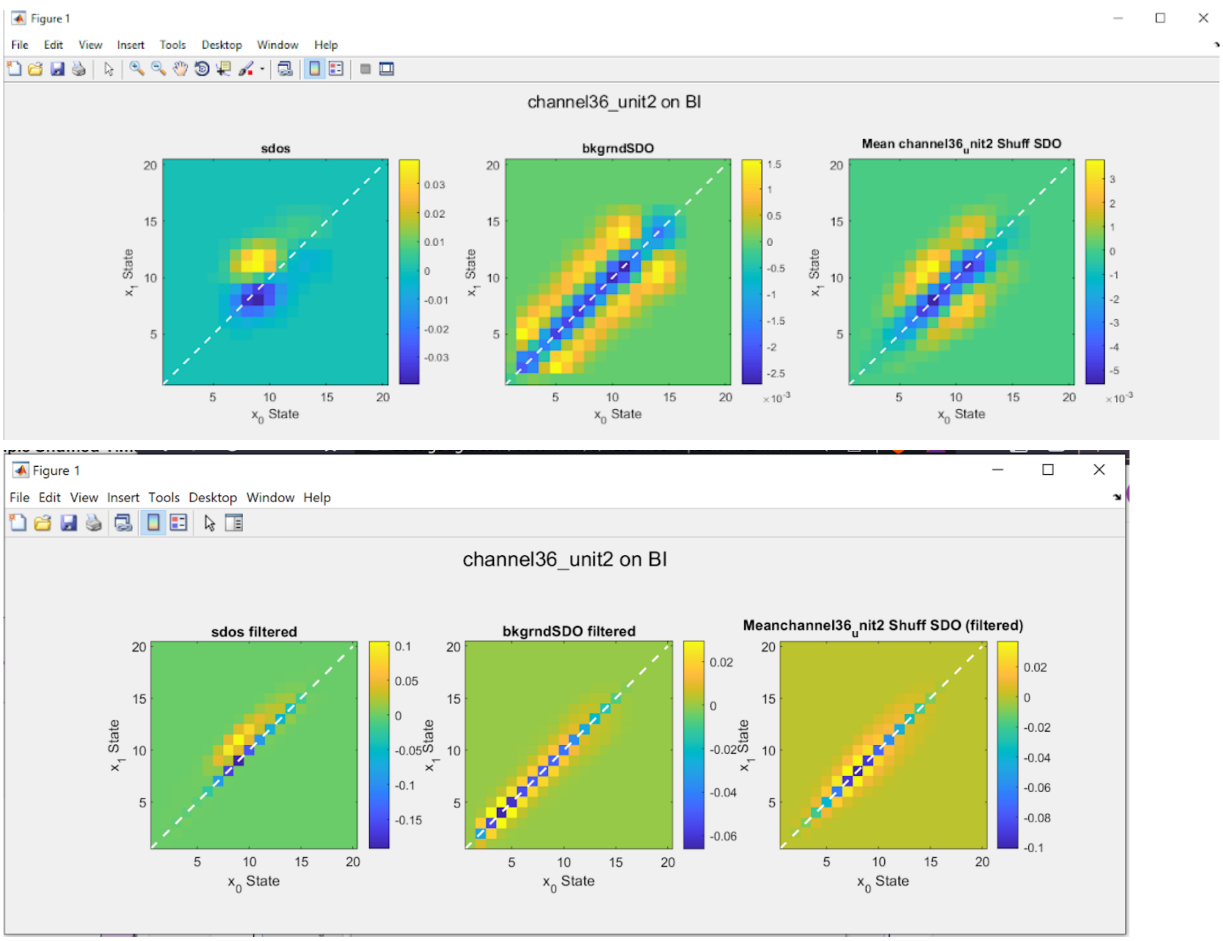

Heat maps of the *differential* SDO matrices for the spike-triggered (‘sdos’), background (‘bkgrndSDO’) and mean-shuffled units (‘Mean Shuff SDO’). Differential SDOs have columns which sum to zero, and thus have positive and negative elements. SDO motifs for the spike-triggered units vs. the null hypotheses can be interpreted by this plot. Here, the spike-triggered SDO resembles a ‘step-up’ motif.

Notes:

- Method of the plotSdo module, which is called by *plotSDO.m*, and may be called individually.

SAT.plot.plotJointDiffSDO.m:

plotSdo.plotJointDiffSDO(**sdo**, **XT_CH_NO, PP_CH_NO**, saveFig, saveFormat, outputDirectory):

Utility:

- Plots the(non-normalized) differential and joint SDOs for the spike-triggered and background spike-sets as heatmaps.

Inputs:

- sdo
    - sdo structure generated by *computeSDO.m*
- XT_CH_NO
    - [integer]
    - XT channel ID number, row position, in *sdo*.
- PP_CH_NO
    - - [integer]
    - PP channel ID number, column subindex, for a given row of *sdo*.
- Optional Name-Value Pairs:
    - filter
        - Logical [0/1]

- If 1, apply a smoothing filter on the SDO matrix diagonals before plotting.
- Default = 1
- saveFig
  - [0/1]
  - If 1, save generated figure to disk.
  - Default = 0
- saveFormat
  - - ['png'/'svg']
  - File format for the saved file.
  - Default = ‘png’
- outputDirectory
  - String containing path to the target save directory.
  - If not provided, and saveFig = 1, will query the user using a GUI.
  - Default = “”
- <u>Output</u>:

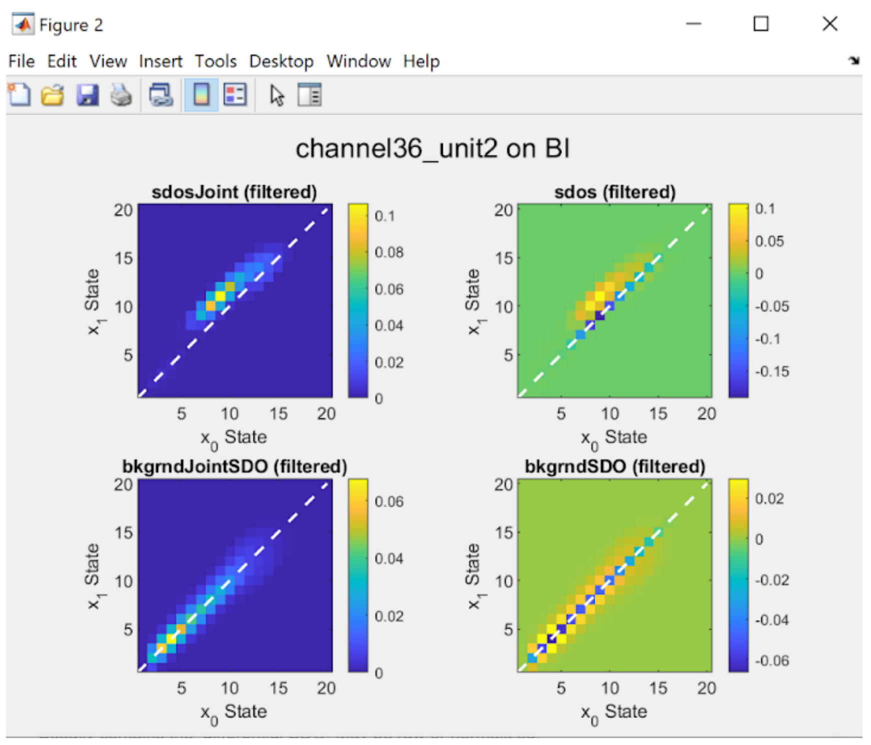

This plot shows both the spike-triggered and background *differential* SDO and the *joint* SDO. The joint SDO is the average convolution of the pre-spike and post-spike state distributions, and hence is the joint distribution of state. The *differential* SDO sums with the pre-spike covariance matrix (not pictured here) to produce the *joint* SDO. Here, we observe that the post-spike distribution of state is towards a higher state (above diagonal; top left). This is the region suggested to be stabilized by the *differential* SDO (top right). In contrast, there are no major asymmetries of the background *joint* SDO.

<u>Notes</u>:

- Method of the plotSdo module, which is called by *plotSDO.m*, and may be called individually.

Plot Alternative SDO Visualizations:

SAT.plot.quiverSDO.m:

SAT.plot.quiverSDO(**sdoMat**, saveFig, saveFormat, outputDirectory, normMat, headPos):

Utility:

- Plots the ‘quiverSDO’ of the provided *sdo* matrix, which highlights the direction of coarse state transition bias for every input state.

Inputs:

- sdoMat
    - A [N_STATES x N_STATES] matrix
    - Usually contains the *differential* SDO; May be raw or normalized.
- Optional Name-Value Pairs:
    - filter
        - Logical [0/1]
        - If 1, apply a smoothing filter on the SDO matrix diagonals before plotting.
        - Default = 1
    - saveFig
        - [0/1]
        - If 1, save the generated figure to ‘outputDirectory’
        - Default = 0
    - saveFormat
        - - ['png'/'svg']
        - File format for the saved file.
        - Default = ‘png’
    - outputDirectory
        - String containing path to the target save directory.
        - If not provided, and saveFig = 1, will query the user using a GUI.
        - Default = ‘’
    - ‘normMat’
        - a [N_STATES x N_STATES] array which may be passed.
        - Serves as an element-wise denominator for sdoMat.
        - Default = Array of ones;
    - ‘headPos’
        - {‘end’/ ‘center’}
        - Whether arrowheads should be placed at the ends of the vector stems, or in the middle.
        - Default = ‘end’.

Output:

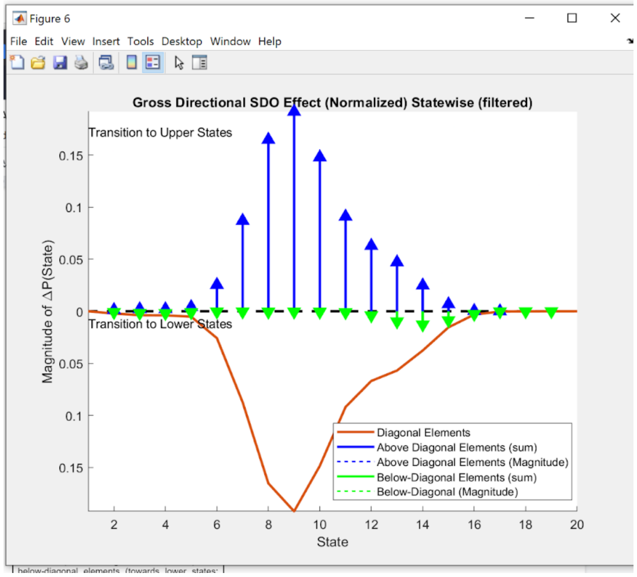

This is the 'quiver SDO' plot for the selected SDO. For each column of the SDO (input state), the summed magnitude of the above-diagonal elements (towards higher states; blue) and below-diagonal elements (towards lower states; green) is plotted as the stem length of a vector. If the value is positive, arrowheads point away from the azimuth, while these point towards the azimuth if the value is negative. The directional effect of the SDO on the output distribution for each input state can be inferred from the behavior of two arrows. (i.e. arrows pointing in the same direction indicate a shift of the post-spike distribution, arrows pointing together suggest a decrease in signal variance, while arrows pointing away suggest an increase in post-spike signal variance. Elements on the diagonal are given as an orange line plot. In this case, the spike-triggered SDO shows strong directional effects, transitioning towards higher states.

<u>Notes</u>:

- Method of the plotSDO module, which is called by *plotSDO.m*, and may be called individually.
- This function may be called for *any* SDO matrix, and for Differential or Joint SDOs. (e.g. Could be used to plot the background, or for the joint distribution of state). Hence, we do not pass the *sdo* structure, to this function, but rather whatever matrix we want ot plot.

SAT.plot.shearSDO.m

SAT.plot.shearSDO(**sdoMat**, saveFig, saveFormat, outputDirectory, N_BANDS)

<u>Utility</u>:

- Used to plot the 'shear' SDO.

<u>Inputs</u>:

- sdoMat
  - A [N_STATES x N_STATES] matrix
  - containing the *differential* SDO; May be raw or normalized.
- <u>Optional Name-Value Pairs</u>:
  - filter
    - Logical [0/1]
    - If 1, apply a smoothing filter on the SDO matrix diagonals before plotting.
    - Default = 1
  - saveFig

- [0/1]
- If 1, save generated figure to disk.
- Default = 0

- saveFormat
  - - ['png'/'svg']
  - File format for the saved file.
  - Default = 'png'
- outputDirectory
  - String containing path to the target save directory.
  - If not provided, and saveFig = 1, will query the user using a GUI.
  - Default = ''
- N_BANDS
  - [Integer]:
  - How many off-diagonal bands to use on either side of the main diagonal for plotting.
  - Default = Number of states / 8

Outputs:

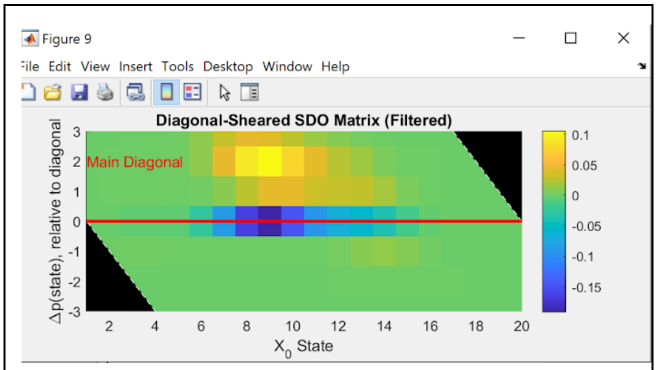

This is the sheared SDO matrix. As SDO matrices often show most of their behavior around the diagonal, and effects may be interpreted relative to the diagonal, shearing the matrix to place the diagonal on the horizontal may be helpful for discerning effects and representing SDO matrices compactly. Here, the spike-triggered SDO shows positive elements above the diagonal, and negative elements below the diagonal.

Notes:

- Method of the plotSdo module, which is called by *plotSDO.m*, and may be called individually.
- This function may be called for *any* SDO matrix, and for Differential or Joint SDOs. (e.g. Could be used to plot the background, or for the joint distribution of state). Hence, we do not pass the *sdo* structure, to this function, but rather whatever matrix we want ot plot.

Plot SDO Significance Statistics:

SAT.plot.risingFallingState.m:

SAT.plot.risingFallingState(**sdo, XT_CH_NO, PP_CH_NO**, saveFig, saveFormat, outputDirectory)

Utility:

- Plot the difference between the above-diagonal (off-diagonal) and below-diagonal (off-diagonal) components (which should all be positive values for a fully-conforming linear operator [1]). This difference is thus the measure of 'coarse bias' in the state transition; the relative shift in the mean of the post-spike distribution, relative to each pre-spike state.

- Co-plotted with this figure is the significance of this overall (i.e. magnitude, over all columns/states), relative to the shuffled SDOs.

Inputs:

- sdo
    - sdo structure generated by *computeSDO.m*
- XT_CH_NO
    - [integer]
    - XT channel ID number, row position, in *sdo*.
- PP_CH_NO
    - - [integer]
    - PP channel ID number, column subindex, for a given row of *sdo*.
- Optional Name-Value Pairs:
    - saveFig
        - [0/1]
        - If 1, save generated figure to disk.
        - Default = 0
    - saveFormat
        - - ['png'/'svg']
        - File format for the saved file.
        - Default = ‘png’
    - outputDirectory
        - String containing path to the target save directory.
        - If not provided, and saveFig = 1, will query the user using a GUI.
        - Default = ‘’

Output:

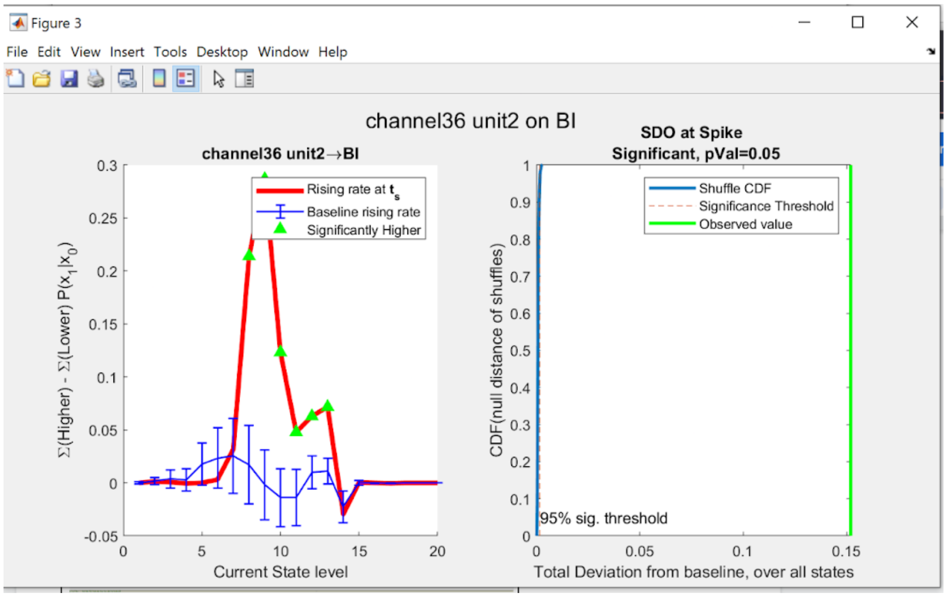

This plot shows the significance of the direction of off-diagonal effects of the *differential* SDO matrix. For each pre-spike state (column) the off-diagonal values supporting an increase in state (positive elements above diagonal; negative elements below diagonal) are compared against the off-diagonal values supporting a decrease in state (positive elements below diagonal; negative elements above diagonal). Thus, the measure on the left is the ‘coarse directional bias’ of the output state, given the input state (red). This bias is compared against the similar metric for spike-shuffled SDOs (means + significance thresholds indicated by blue line + error bars). Here, the spike-shuffled SDO has a significant state bias towards increased states (green triangles) over input states 6-15. On the right is a cumulative distribution function of the ‘coarse state bias’ calculated for

the spike-shuffled SDOs (blue). The threshold for significance was treated as the top 5% of this CDF (orange dotted bar). The metric for the spike-triggered SDO is given in green, to the right of the threshold, indicating the spike-triggered SDO has significant coarse bias.

Notes**:**
- Method of the plotSDO module, which is called by *plotSDO.m*, and may be called individually.
- If N_SHUFF used to evaluate to test significance of the SDO is set to 0, this figure will still plot, but a single black line will be given at 0 on the left, and no CDF will be generated on the right (although this may still be displayed as 'significant')

SAT.plot.arraySig.m:

SAT.plot.arraySig(**sdo, XT_CH_NO, PP_CH_NO**, zTransform, saveFig, saveFormat, outputDirectory):

Utility:
- Used to visualize significant elements/pixels of the SDO, relative to the distribution of shuffled-spike SDOs.
- Co-plotted with this figure is the significance of this overall (i.e. magnitude, over all elements), relative to the shuffled SDOs.

Inputs:
- sdo
  - sdo structure generated by *computeSDO.m*
- XT_CH_NO
  - [integer]
  - XT channel ID number, row position, in *sdo*.
- PP_CH_NO
  - - [integer]
  - PP channel ID number, column subindex, for a given row of *sdo*.
- zTransform
  - [0/1] (Optional, positional)
  - If 1, z-transform array elements across the shuffles prior to calculating significance.
- Optional Name-Value Pairs:
  - saveFig
    - [0/1]
    - If 1, save generated figure to disk.
    - Default = 0
  - saveFormat
    - - ['png'/'svg']
    - File format for the saved file.
    - Default = 'png'
  - outputDirectory
    - String containing path to the target save directory.
    - If not provided, and saveFig = 1, will query the user using a GUI.
    - Default = ''

Output:

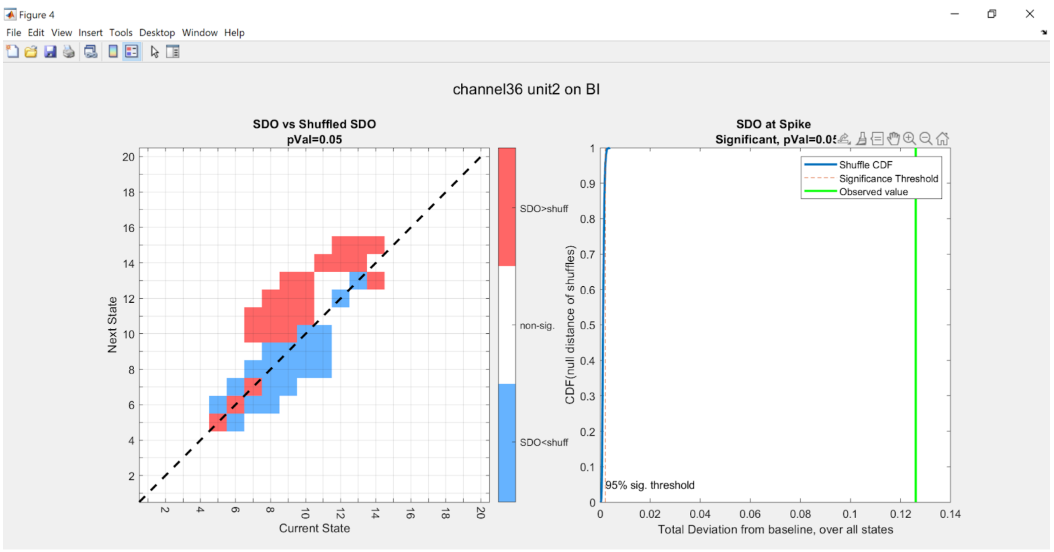

This plot shows the significance of individual pixels/elements of the *differential* SDO matrix, relative to the shuffled SDOs. Here, each spike-triggered SDO element value is compared against the distribution of the 1000 shuffled SDOs. Elements may be significantly greater (red) or lesser (blue) value than the shuffled SDO value. (Here, the spike-triggered *differential* SDO has many regions above the diagonal significantly greater than shuffled, and many regions below diagonal significantly less than the shuffled values). On the right is a plot of the cumulative distribution function of the total distance between each shuffled array and the mean of the shuffles (square of the distance between elements, summed over all values). The significance threshold is plotted as an orange dashed bar. The statistic calculated for this unit is given as a green bar, to the right of the threshold, indicating significance for this metric.

<u>Notes</u>:

- Method of the plotSDO module, which is called by *plotSDO.m*, and may be called individually.
- If N_SHUFF used to evaluate to test significance of the SDO is set to 0, this figure will still plot, but will give a plain white matrix be on the left, and no CDF will be generated on the right (although this may still be displayed as ‘significant’)

SAT.plot.px0Sig.m:

SAT.plot.px0Sig(**sdo, XT_CH_NO, PP_CH_NO**, saveFig, saveFormat, outputDirectory):

<u>Utility</u>:

- Used to plot the average pre-spike state distribution for the SDO.
- Co-plotted with this figure is the significance of the spike-triggered vs. average shuffled pre-spike distributions (measured as the KLD), relative to the shuffled SDOs.
- The Kullback-Leibler Divergence (KLD) is a premetric used to categorize the ‘distance’ between two probability distributions.

<u>Inputs</u>:

- sdo
    - sdo structure generated by *computeSDO.m*
- XT_CH_NO
    - [integer]
    - XT channel ID number, row position, in *sdo*.
- PP_CH_NO
    - - [integer]
    - PP channel ID number, column subindex, for a given row of *sdo*.
- <u>Optional Name-Value Pairs</u>:
    - saveFig
        - [0/1]

        - If 1, save generated figure to disk.
        - Default = 0
    - saveFormat
        - - ['png'/'svg']
        - File format for the saved file.
        - Default = ‘png’
    - outputDirectory
        - String containing path to the target save directory.
        - If not provided, and saveFig = 1, will query the user using a GUI.
        - Default = ‘’
-

Output:

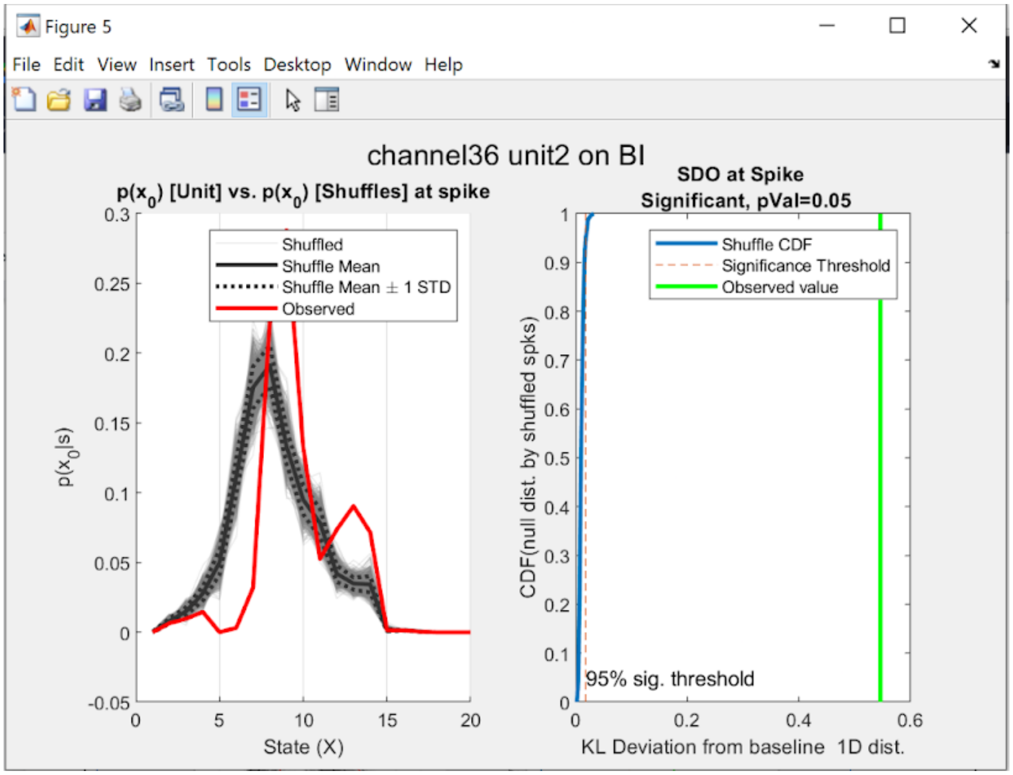

This plot shows the average pre-spike distribution of the unit(red), relative to the equivalent distributions for the spike-shuffled points (gray). The mean and standard deviation of the shuffles is given in black. On the right is a cumulative distribution function of the Kullback-Leibler Divergence (KLD) between each shuffled distribution, and the mean of the shuffled distributions. The threshold for significance is given in orange. The KLD between the spike-triggered pre-spike distribution and the mean-shuffled distribution is given in green. In this case, the spike-observed value is to the right of the threshold, indicating significance.

Notes:

- Method of the plotSDO module, which is called by *plotSDO.m*, and may be called individually.
- If N_SHUFF used to evaluate to test significance of the SDO is set to 0, this figure will still plot, but a single black line at 0 will be given on the left, and no CDF will be generated on the right (although this may still be displayed as ‘significant’)

Use SDO to predict probability distributions of state

Use the selected spike-triggered SDO matrix to generate predicted probability distributions from supplied probability distributions.

Evaluate the performance of different predictive methods using the supplied dataset.
Hypotheses:

1) **x(t0)=x(t1)**: (‘t0t1’)
    a) Hypothesis that there is no change in the post-spike distribution relative to the pre-spike distribution.
    b) *Effected by multiplying the pre-spike distribution with the identity matrix*.
2) **Gaussian H0**:
    a) Hypothesis that the post-spike distribution is an increase in the distribution variance without a change in the mean.
    b) *Effected by convolving the identity matrix with the exponential decay kernel filter used in the generation of smoothing filters for px*. *This matrix is thus used as the transition matrix*.
3) **Spike-triggered Average**:
    a) Hypothesis that the post-spike signal distribution is the average post-spike distribution.
    b) *Effected by using the row-sum of the spike-triggered joint SDO, replicated into a square matrix, to use as a transition matrix*.
4) **Background Dynamics**:
    a) Hypothesis that post-spike signal distribution may be accurately predicted using only the background joint SDO.
    b) *Effected by using the column-normalized background joint SDO as a transition matrix*.
5) **1st Order Markov**:
    a) Hypothesis that distribution of states in the post-spike interval may be predicted from the appropriate convolution of the Markov matrix constructed from all points in the post-spike interval.
    b) *Effected by capturing the signal states in the post-spike interval, and calculating the state-state transitions between adjacent points, over all points. The 1st-Order Markov is then multiplied by the number of states in the post-spike interval, to equalize the expected number of bins*.
6) **Spike-triggered dpx + Background Dynamics**:
    a) Hypothesis that the post-spike signal distribution is the combination of the background joint SDO + the row-sum of the
7) **Spike-Triggered SDO**:
    a) Hypothesis that the post-spike signal distribution is the post-spike conditional distributions.
    b) *Effected by using the joint spike-triggered SDO distribution, column-normalized, as the transition matrix.*

The script *predictSDO* may be used as an all-in-one script for generating a prediction using the SDO, and plotting this prediction. Probability distributions may also be predicted using the functions within the *predict* module, or manipulated using *pxTools* library.

SAT.predictSDO.m

SAT.predictSDO():

<u>Utility</u>:

- Header script for calling all predictSdo methods.
- Different parameters may be set within the script header
- Generates predictions of the post-spike state distributions, using the given *sdo* and *xtData* and *ppData*.
    - These may be the same *xtData* and *ppData* used in the original generation of *sdo*.

        - Thes may be different *xtData* or *ppData*s,(but of course, these new data must have ID values which match the same units and channels used in *sdo* for analysis).

Prerequisites:
- *computeSDO()*

Input Arguments:
- {None}

Header Arguments:
- XT_DC_CH_NO
    - Integer
    - Index number of the time series data within the *xtData*.
- PP_DC_CH_NO
    - Integer
    - Index number of the point process data within the *ppData*.
- STATE_ASSIGNMENT
    - {‘max’/’mean’/’median’}
    - How to assign a single best ‘state’ to a predicted state distribution.
    - Default = ‘max’
- N_DT_INTERVALS
    - How many intervals (of length dt, the length used to predict the SDO) forward with.
    - Assumes that the spike-triggered dynamics are constantly maintained throughout the intervals.
    - Default = 1;
- PLOT_ON
    - Logical [0/1]
    - Whether to plot predicted probability distributions/state after predictions.
    - Default = 1;
- SAVE_FIG
    - Logical [0/1]
    - Whether to save plotted figures
    - Default = 0;
- SAVE_FMT
    - {‘png’/’svg’}
    - Save format type, if saving figures.
    - Default = ‘png’
- SAVE_DIR
    - {string/char}
    - Save directory for output figures, if SAVE_FIG = 1;
    - Default = “”

Output:
- {None}

CALLED FUNCTIONS:
- ***SAT.predict.predictPx()***
- ***pxTools.getXFromPx()***
- ***SAT.predict.assignPlotterProperties()***
- ***SAT.predict.plotter()***

SAT.predict.predictPx.m:

SAT.predict.predictPx(**sdo, xtData, ppData, XT_DC_CH_NO, PP_DC_CH_NO**, xtID, ppID, xtIDField, ppIDField, xtDataField):

Utility:
- Generates predicted distributions of state from observed data (*xtData* and *ppData*) and the *sdo* struct.

Prerequisite:
- *computeSDO()*

Reliancies:
- pxTools Library

Input Arguments:
- sdo
  - *sdo* structure containing the stochastic dynamic operators, both differential and joint matrices.
- xtData
  - *xtData* containing observations of time-series data over which to generate a predicted distribution of signal state.
  - → This may be the same data used to generate *sdo*, or it can be different. If different, the value in the field **.electrode** in *xtData* must match the value for **.signalType** in *sdo.*
- ppData
  - *ppData* containing observations of point-process data over which to generate a predicted distribution of signal state.
  - → This may be the same data used to generate *sdo*, or it can be different. If different, the value in the field **.electrode** in *ppData* must match the value for **.neuronNames** in *sdo.*
- XT_DC_CH_NO
  - Integer. Channel number (row) index for the supplied *xtData* to use in the predictions of post-event state distributions.
  - If not provided, “xtID” (below) must be defined to specify a reference time series data channel.
- PP_DC_CH_NO
  - Integer. Channel number (row) index for the supplied *ppData* to use in the predictions of post-event state distributions.
  - If not provided, “ppID” (below) must be defined to specify a reference time series data channel.
- Optional Name-Value Arguments
  - “xtID” : [string/char]
    - The value matching the **.electrode** fieldname in *xtData* to use.
    - → Allows string-matching instead of use of row-indices for specifying data for predictions.
  - “ppID” : [string/char]
    - The value matching the **.electrode** fieldname in *ppData* to use.
    - → Allows string-matching instead of use of row-indices for specifying data for predictions.
  - “xtIDField” : [string/char]
    - If reference ID field in *xtData* is not mapped to **.electrode**, it must be defined here.
  - “ppIDField” : [string/char]

        - If reference ID field in *ppData* is not mapped to **.electrode**, it must be defined here.
    - “xtDataField”: [string/char]
        - The field containing the time series data in *ppData*, if not mapped to **.envelope**
    - “staType”: { ’avg_px’ / ‘px_sta’}. Switch for how to consider the spike-triggered average in the probabilistic context.
        - If ‘avg_px’; Take the average post-spike state probability distribution as the STA.
        - If ‘px_sta’; Take the probability distribution of the state-transformed impulse response.
        - Default = ‘avg_px’;
    - ‘mkvDist’: {‘px0’ / ‘px1’}: Switch for which distribution to use when estimating the 1-step markov transition matrix.
        - If ‘px0’, generate a 1-step markov matrix between sequential observations in the *pre-spike* distribution.
        - If ‘px1’, generate a 1-step markov matrix between sequential observations in the *post-spike* distribution.
        - Default = ‘px0’
    -

Output:

- predicted_px:
    - Struct of predicted state distributions, by method. Contains the following fields:
        - **.t0t1**: No change prediction.
            - [N_STATES x N_SPIKES] Doubles Array
        - **.gaussH0**: Random drift prediction
            - [N_STATES x N_SPIKES] Doubles Array
        - **.mkv**: 1st-Order Markov prediction
            - [N_STATES x N_SPIKES] Doubles Array
            - Influenced by ‘mkvDist’ argument, above;
        - **.bck**: Background SDO Dynamics Prediction
            - [N_STATES x N_SPIKES] Doubles Array
        - **.STA** Spike-triggered averaged state prediction.
            - [N_STATES x N_SPIKES] Doubles Array
            - Influenced by ‘mkvDist’ argument, above
        - **.staBck** Spike-triggered Constant offset + Background array
            - Influenced by STA arguments
        - **.SDO** Spike-triggered SDO prediction.
            - [N_STATES x N_SPIKES] Doubles Array
- observed_px
    - Struct of observed perispike state distributions. Contains the following fields:
        - **.t0_actual**: Observed pre-spike state distributions
            - [N_STATES x N_SPIKES] Doubles Array
        - **.t1_actual**: Observed post-spiek state distributions
            - [N_STATES x N_SPIKES] Doubles Array
    - Only returned if 2+ outputs are requested.
- observed_at
    - Struct of observed reference signal amplitude collected around spike, prior to state transformation.
        - **.t0_actual**: Observed pre-spike signal amplitude
            - [(dt-s,s) x N_SPIKES] Doubles Array

- **.t1_actual**: Observed post-spike signal amplitude
    - [(s,s+dt) x N_SPIKES Doubles array
- Only returned if three outputs are requested.

Example Code:

```
>>xtData = load('emgCell.mat');
>>ppData = load('spikeTimeCell.mat');

>>xtChNo = 8;
>>ppChNo = 4;

%// using standard dataCells, run default prediction parameters
>>[pd_px, obs_px] = SAT.predict.predictPx(sdo, xtData, ppData, xtChNo, ppChNo);

%// show fields
pd_px =

    struct with fields:

    t0t1: [20x703 double]
   gauss: [20x703 double]
     sta: [20x703 double]
     bck: [20x703 double]
     mkv: [20x703 double]
  staBck: [20x703 double]
     sdo: [20x703 double]
```

IMPORTANT NOTES:
- *ppData* and *xtData* used here follow the same assumptions as earlier in the pipeline (e.g. homogeneity of size).
- *ppData* and *xtData* may be a subset of the original data, the original data, or novel data, provided the fieldname used for the Datas match those used in *sdo*.
- The same parameters used to treat the state probability distributions used in the construction of *sdo* are used in the prediction of post-spike state.

Generate Predicted States from Distributions

States may be assigned to a distribution by one of three methods:
- 'max' : Take the peak of the state distribution as the single state.
- 'Mean': Take the mean value (rounded) of the distribution as the single state
- 'Median': Take the median value (rounded) of the distribution as the single state.

Single state predictions may be quickly generated from observed/predicted probability distribution structures by *pxTools.getXfromPx.m*.

pxTools.getXfromPx.m

pxTools.getXfromPx(**px**, method):
- Utility:
    - Get state assignment from observed probability distributions or structure containing these distributions
- Prerequisites:
    - None
- Input:
    - px

        - A [N_STATES x N_OBSERVATIONS] doubles array, or a structure whose fields are such doubles arrays.
    - method
        - {‘max’, ‘mean’, ‘mode’}: String/char. One of three potential assignment methods.
- Output:
    - X
        - If *px* is a doubles array, X is a [1 x N_OBSERVATIONS] vector.
        - If *px* is a struct, X is a struct with matching fields, containing such vectors.

Example Code

```
pd_px =

    struct with fields:

    t0t1: [20x703 double]
   gauss: [20x703 double]
     sta: [20x703 double]
     bck: [20x703 double]
     mkv: [20x703 double]
  staBck: [20x703 double]
     sdo: [20x703 double]

>>stateAssignment = ‘max’;

%// assign single state to predicted distributions
>> pd_x = pxTools.getxfromPx(pd_px, stateAssignment);

pd_x =

    struct with fields:

    t0t1: [1x703 double]
   gauss: [1x703 double]
     sta: [1x703 double]
     bck: [1x703 double]
     mkv: [1x703 double]
  staBck: [1x703 double]
     sdo: [1x703 double]
```

Measure Prediction Errors Between Predicted and observed states and distributions

SAT.predict.calcPredictionError.m

SAT.predict.calcPredictionError(**x0StateArr, x1StateArr, pdStateArr, x1PxArr, pdPxArr**, N_BINS, refName)

Utility: Generate ‘errorStruct’, a data structure containing the prediction errors for states and distributions

Inputs:

- x0StateArr:
    - A [1xN] vector containing the observed pre-spike states.
    - May also be a {N_XT_CH, N_PP_CH} cell array, of similar [1xN] vectors.
- x1StateArr:
    - A [1xN] vector containing the observed post-spike states.
    - May also be a {N_XT_CH, N_PP_CH} cell array, of similar [1xN] vectors.
- pdStateArr:

        - A struct containing fields whose values are [1xN] vectors of predicted post-spike states. May also be a {N_XT_CH, N_PP_CH} cell array of similar structs.
- x1PxArr:
    - A [N_BINS x N] doubles array containing column vectors of probability distributions of post-spike state
    - May also be a {N_XT_CH, N_PP_CH} cell array, of similar [N_BINSxN] vectors.
- pdPxArr:
    - A struct containing fields whose values are [N_BINS x N] column vectors of probability distributions of post-spike state
    - May also be a {N_XT_CH, N_PP_CH} cell array, of structs whose fields are similar [N_BINSxN] vectors.
- N_BINS:
    - Integer, or called empty.
    - Number of states used, used for calculating state-wise error, and ensuring all states are accounted for (even if not observed).
    - If not provided, will use the maximum observed value as the number of states.
- OPTIONAL NAME-VALUE PAIRS:
    - 'refName'
        - String. Name of the field which is treated as the 'ground truth' for post-spike prediction. Used as a simple identifier in errorStruct. Default = 't1_actual'

<u>Outputs</u>:

- errorArr:
    - 1 x 6 structure (for each hypothesis used) containing the following fields:
        - **.fieldname**:
            - String/char: Char representation of the hypothesis name.
        - **.reference**:
            - String/char: Char representation of the ground-truth name.
        - **.x0States**:
            - Observed (single) prespike signal state.
        - **.L0_running**:
            - [1/0] error for predicted states vs. ground truth, calculated over all spiking events (and indexed chronologically)
                - 1 = Mismatch between prediction and ground truth
                - 0 = Coherence between prediction and ground truth.
        - **.L1_running**:
            - Magnitude of error between predicted states and ground truths, calculated for all spiking events
        - **.L2_running**:
            - Squared magnitude of error between predicted states and ground truths, calculated for all spiking events
        - **.L_inf**:
            - The maximum magnitude of L1 error ever observed between hypothesized and predicted states.
        - **.L0_running_x_state**:
            - Running L0 error, by prespike state
        - **.L0_x_state**:
            - Cumulative L0 error by prespike state.
        - **.L1_running_x_state**:
            - Running L1 error, by prespike state

- **.L1_x_state**:
    - Cumulative L1 error by prespike state.
- **.L2_running_x_state**:
    - Running L2 error, by prespike state
- **.L2_x_state**:
    - Cumulative L2 error by prespike state.
- **.KLD**:
    - The KLD between each observed and predicted post-spike distribution.

Example Code

```
x0_actual = obs_x.t0_actual; %// prespike states
x1_actual = obs_x.t1_actual; %// postspike states

pd_x;

px1_actual = obs_px.t1_actual;

pd_px;

errorStruct = SAT.predict.calcPredictionError(...
    x0_actual, ...
    x1_actual,...
    pd_x1,...
    px1_actual, ... ...
    pd_px );


errorStruct =

  1×6 struct array with fields:

    fieldname
    reference
    x0States
    L0_running
    L1_running
    L2_running
    L_inf
    L0_running_x_state
    L0_x_state
    L1_running_x_state
    L1_x_state
    L2_running_x_state
    L2_x_state
    KLD
```

Visualize Predicted Distributions and States

Visual depiction of the SDO predictions of state and distributions may be accessed through the predictSDO_plotter library. Individual plotters may be called for a single function, or *predictSpike_plotter.m* may be executed, to plot using all methods simultaneously.

pxTools.plot.sta_v_x.m

pxTools.plot.sta_v_x(**at0, at1,** xs, saveFig, saveFormat, outputDirectory, binMS):
Utility:
- Used to plot the standard spike-triggered average signal amplitude, and the state-dependent spike-triggered average

Input:

- at0
    - [N_X0PTS x N_OBS Array]
    - Pre-spike Signal Amplitude
- at1
    - [N_X1PTS x N_OBS Array]
    - Post-spike Signal Amplitude
- xs
    - [1 x N_OBS] Vector, of state-at-spike
    - If xs is not provided, STA will be calculated across all events.
- <u>Optional Name-Value Pairs</u>:
    - saveFig
        - [0/1]
        - whether to trigger the save module
    - saveFormat
        - ['png'/'svg']
    - outputDirectory
        - String containing path. If not provided, will query user.
    - binMS
        - Numeric
        - Relationship between time interval and bin size.

<u>Output</u>:

xs not provided.

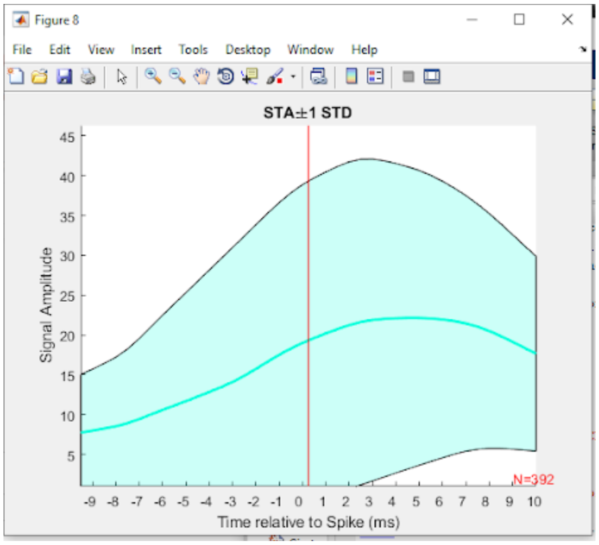

This is the spike-triggered average signal waveform, over a region ± 10 ms relative to spike. The mean waveform is plotted with an envelope ± 1 standard deviation. The total number of spikes used in the STA is given in the bottom right. The time of spike is indicated as a red vertical line. Because the standard deviation is so large (wide envelope on either side of spike), no significance would be assigned to this STA.

xs provided.

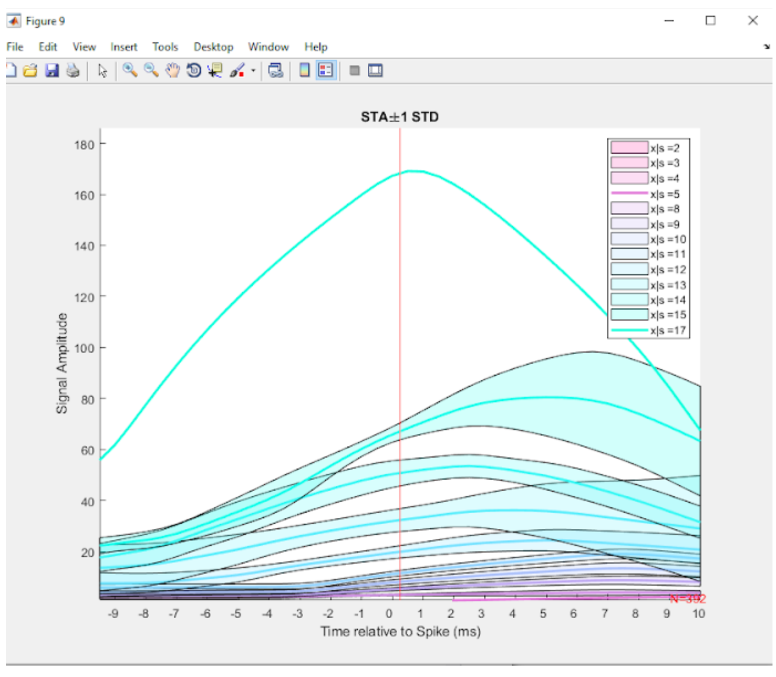

This is a spike-triggered average of signal waveform, segregated by signal state at time of spike (state-dependent STA). The mean waveform of each group is given as a solid line, with the standard deviation (if N > 1) is presented as a translucent envelope. The total number of spikes used is printed in the bottom right corner. If a state-at-spike is not observed, then it will not be plotted (and hence missing on the legend). Here, there are state-dependent effects of the STA.

IMPORTANT NOTES:

- px0, px1, and xs should all match the number of spiking elements.
- The order of elements in px0, px1 and xs should be conserved.

pxTools.plot.px_v_x_waterfall.m:

pxTools.plot.px_v_x_waterfall(**px0, px1, xs**, saveFig, saveFormat, outputDirectory)

Utility:

Plots waterfall/ ribbon figure of the pre-spike, post-spike, and delta probability state distributions, by state. Colors are randomly generated along a smooth continuum.

Input:

- px0
  - [N_BINS x N_OBS] array of pre-spike state distributions, by observation.
- px1
  - [N_BINS x N_OBS] array of post-spike state distributions, by observation.
- xs
  - [1 x N_OBS] row vector.
  - State of signal at spike (in order of observations in px0/px1).
- OPTIONAL NAME-VALUE PAIRS:
  - 'saveFig' : [0/1]. If 1, save plotted figure
  - 'saveFormat' : ['png'/'svg']. Save format for the figure;
  - 'outputDirectory': string/char. If not passed here, query user for save position

Output:

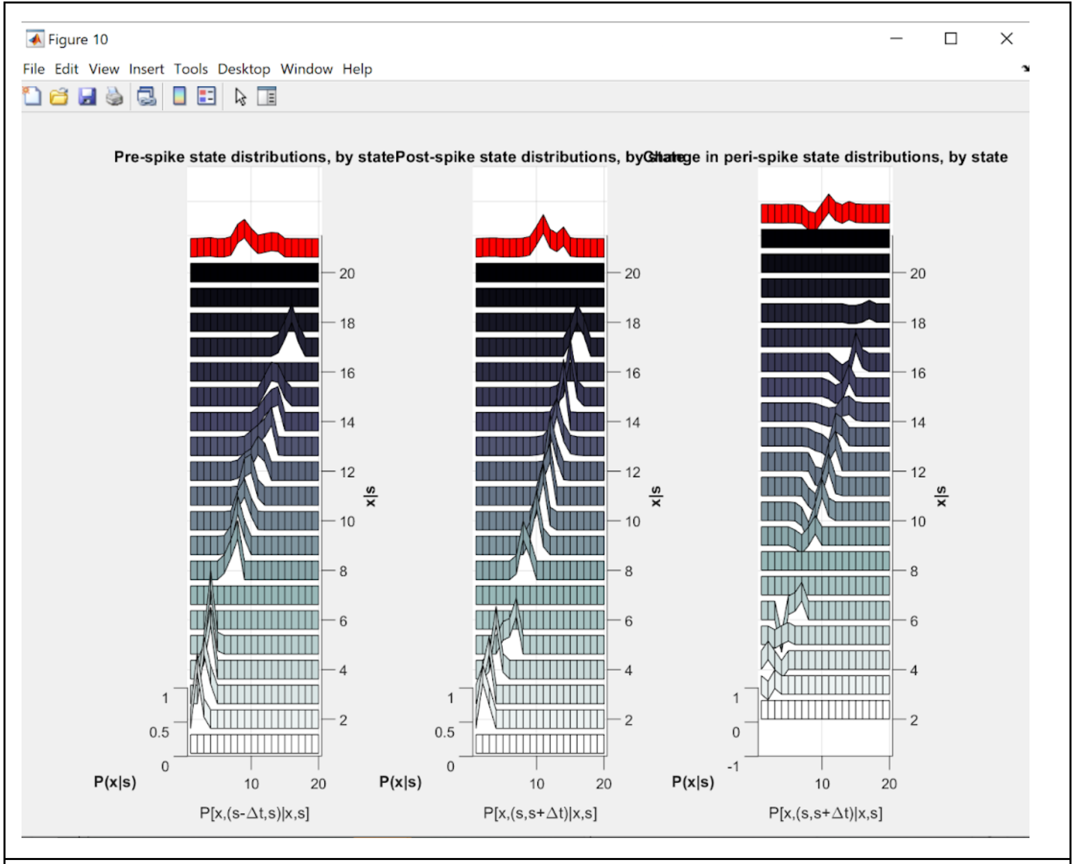

This is the average state-dependent pre-spike (column 1) and post-spike (column 2) distributions observed for the spike-triggered unit. The average state-dependent change in these two distributions is given in column 3. State at time of spike is given on the vertical bar (row of horizontal ribbon), while states composing the state-dependent distributions are indexed on the horizontal. The overall average pre-spike , post-spike, and change of distributions are given as the red ribbon at the top of the plots. Here, there are two sets of 'missing' states (5-7; 16) which are not observed in the prespike or post-spike distributions.

IMPORTANT NOTES:

- px0, px1, and xs should all match the number of spiking elements.
- The order of elements in px0, px1 and xs should be conserved.

pxTools.plot.staPxt.m

pxTools.plot.staPxt(**x0, x1**, N_STATES, BY_STATE, saveFig, saveFormat, outputDirectory):

Utility:

- Used to plot the P(x,t) peri-spike state heatmaps.
- Can be used over all states, or segregated by state-at-spike.

Inputs:

- X0: A [x0 Points x nSpikes] array containing pre-spike states.
- X1: A [x1 Points x nSpikes] array containing post-spike states.
- N_STATES: [Optional], integer. Maximum number of states used for statemapping.
- Optional Name-Value Pairs:
    - BY_STATE
        - Logical [0/1].
        - Flag for whether to average over all points, or to make separate heat map for each set of states at spikes.
        - Default = 0
    - saveFig

- Logical [0/1];
- whether to trigger the save module
- Default = 0

- saveFormat
    - {'png'/'svg'}
    - Default = ‘png’
- outputDirectory
    - - String containing path.
    - If not provided, will query user.

Output:

BY_STATE = 0;

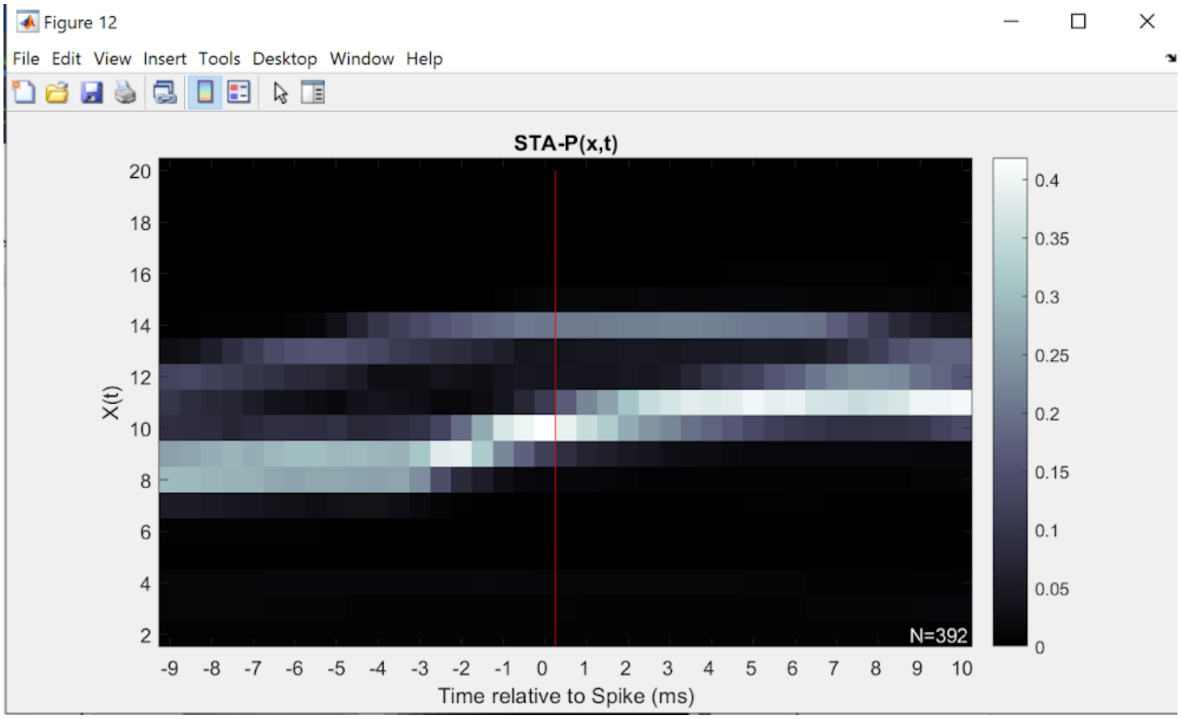

This image is a heatmap of the spike-triggered average probability of state for every indexed time. Each column is a probability distribution of state observed for the specific time point, relative to spike, across all experimental observations. The total number of spikes used to generate the heatmap is given in the bottom right corner. Time of spike is indicated as a red line. This plot is useful for identifying gross perispike signal behavior. Here, we see what appears to be two major trajectories for the time series signal, with post-spike signal behavior predictable from signal state at spiketime.

BY_STATE = 1;

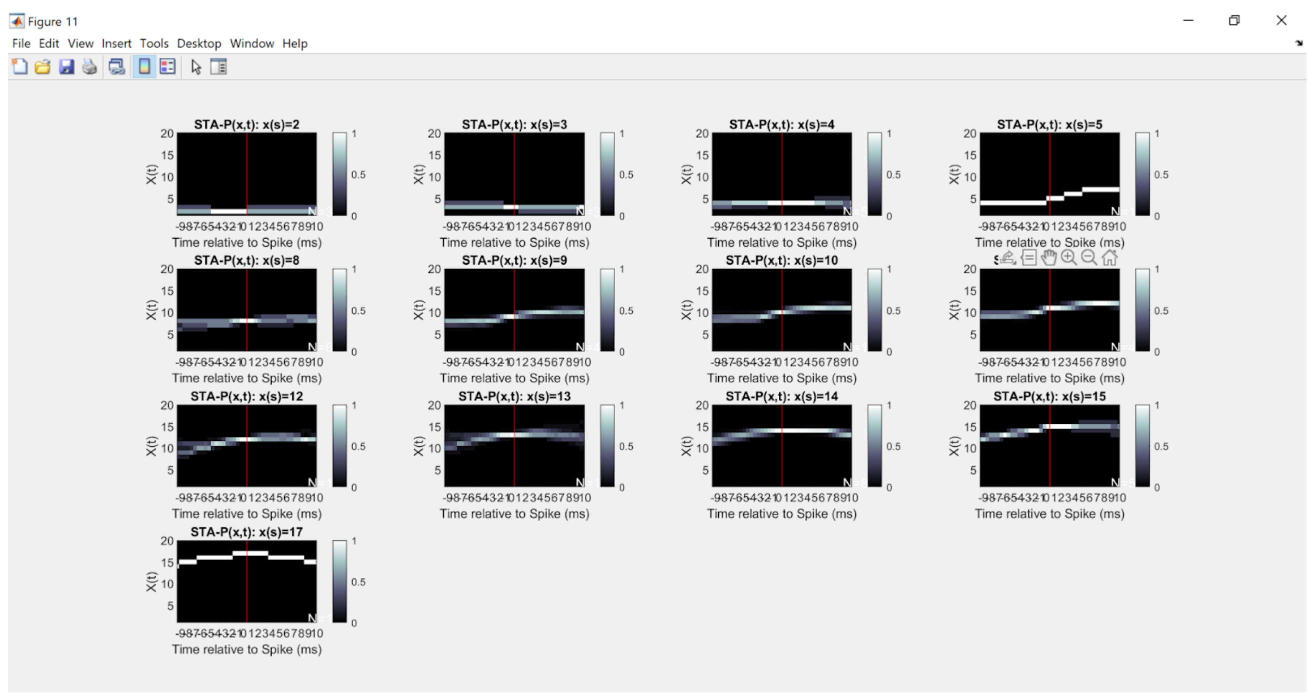

This is a heatmap of the spike-triggered state probability distribution for every time bin relative to spike, segregated by state at spiketime. The number of spikes used in each bin is given in the bottom right of each figure. The heatmaps are standardized between different states, such that the colors are comparable. Here, we observe that the pre-spike signal behavior varies by signal state at spiketime.

IMPORTANT NOTES:
- If a state is not observed at time of spike, it will be missing on the 'BY_STATE = 1' heatmap.

Plot State Predictions and Errors:

SAT.predict.plot.px_v_x.m

SAT.predict.plot.px_v_x(**pdPx1, x1**, subIndex, saveFig, saveFormat, outputDirectory):

Utility:
- Used to overlay the predicted post-spike distribution with the observed post-spike state.

Inputs:
- 'pdPx1' - Predicted Probability Distribution (Organized as a Mx1 column vectors, over N events)
- 'x1' - Observed post-spike state, organized as a [1xN] vector
- Optional Name-Value Pairs:
    - 'subIndex' - A subset of column indices to use for plotter.
    - saveFig - [0/1]; whether to trigger the save module
    - saveFormat - ['png'/'svg']
    - outputDirectory- String containing path. If not provided, will query user

Output:

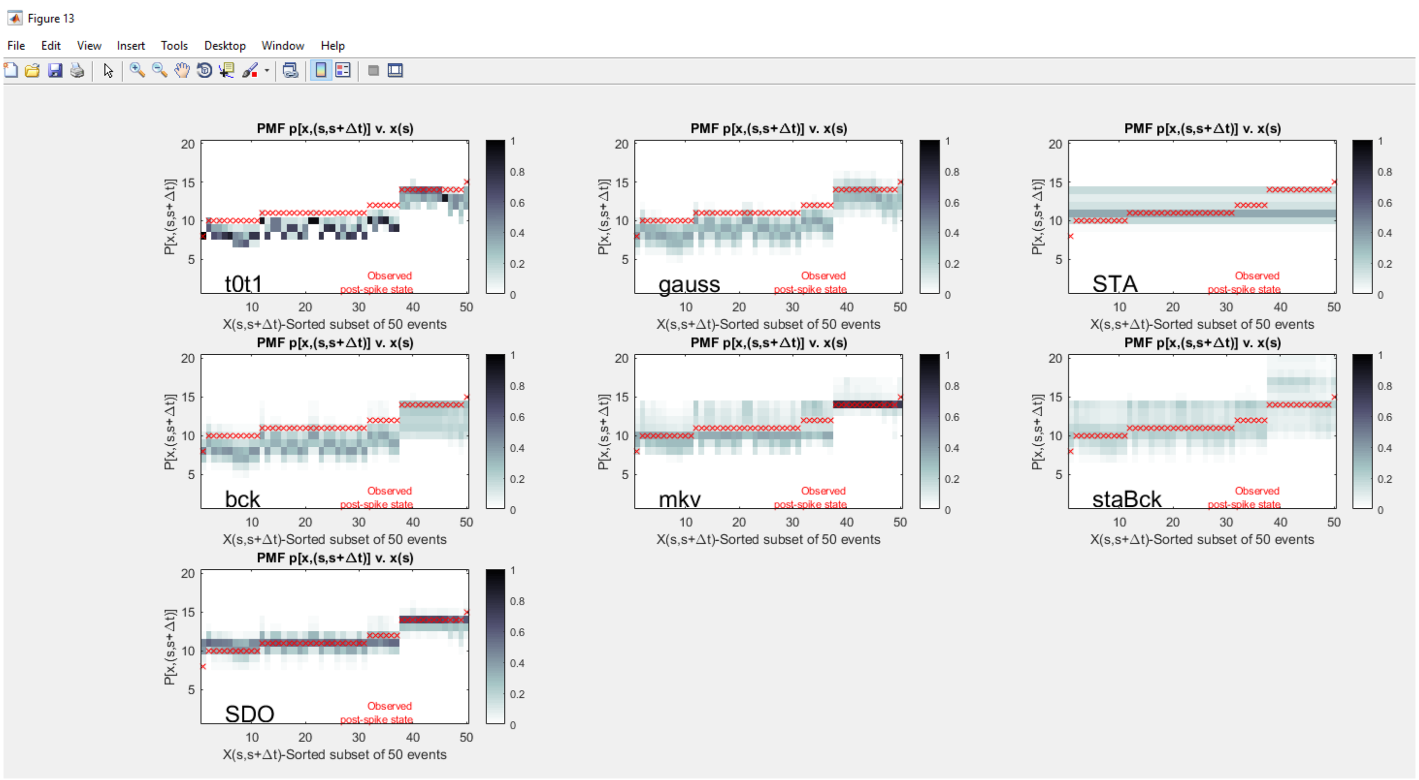

This plot visually demonstrates the coherence between state distribution predictions, generated from different hypotheses (gray heatmap), compared to the observed post-spike signal state (red x). Each column is a predicted distribution. Independent columns were sorted by pre-spike signal state, then horizontally concatenated to produce the resultant heatmap. This figure is useful for determining how well each prediction tracks dynamic signal behavior around spike. Here, most predictions fail to accurately predict post-spike signal state (i.e. the heat map is not concentrated around the red x), or these predictions are only accurate over a range of values (e.g. markov, STA). Only the SDO-based predictions remain accurate over all randomly-sampled predictions. T0t1 = No change hypothesis [H1]; gaussH0 = gaussian convolution [H2]; STA = spike-triggered average Px [H3], bck = background SDO [H4]; mkv = First-order markov [H5]; staBck = Background SDO w/ STA-derived offset [H6]. SDO [H7].

SAT.predict.plot.error_v_state.m

predictSdo.plot_error_v_state(**errorStruct**, saveFig, saveFormat, outputDirectory, plotProp, method):

Utility:

- Plot the stacked barchart of average error rate, by state, for each hypothesis.

Inputs:

- errorStruct: The common error prediction array generated by predictSdo.calcPredictError.m
- Optional Name-Value Pairs:
  - ‘saveFig’
    - [0/1] Flag.
    - Whether to automatically save the figure;
  - ‘saveFormat’
    - - [‘png’/’svg’].
    - Format for saving figure.
  - ‘outputDirectory’
    - - Where to save the figure; if not provided, will query the user for input.
  - ‘plotProp’
    - - The standard plotting properties structure, for color and line properties. If not provided will default to standard MATLAB colors
  - ‘method’
    - - {‘mean’, ‘median’, ‘mode’}: Definition of average to use for the plotter.

- Default = ‘mean’

<u>Output</u>:

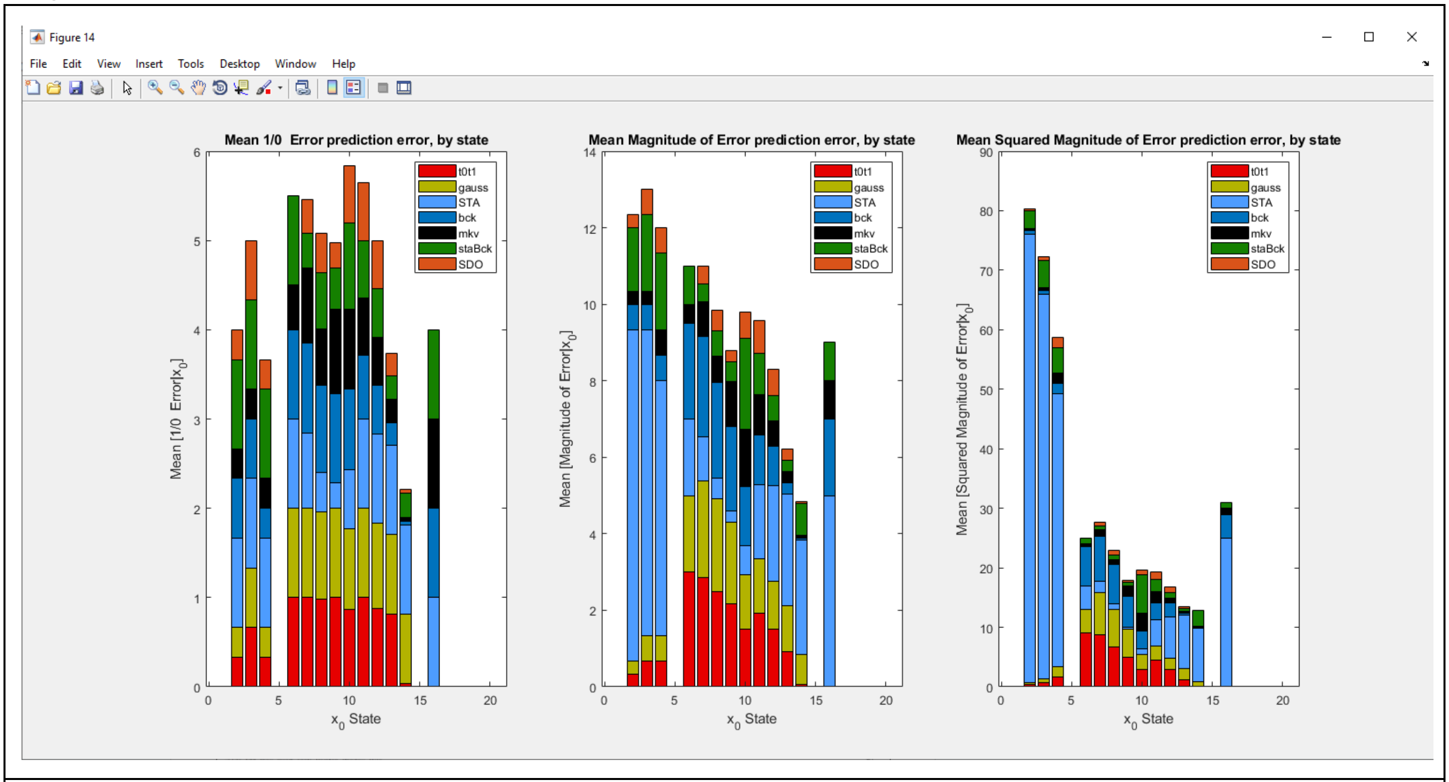

This stacked barchart shows the average prediction error of each prediction hypothesis, by pre-spike signal state. ‘Mean’, ‘median’, or ‘mode’ average error can be predicted. If no spikes are observed in a pre-spike signal state, the bar will be missing. T0t1 = No change hypothesis [H1]; gaussH0 = gaussian convolution [H2]; STA = spike-triggered average Px [H3], bck = background SDO [H4]; mkv = First-order markov [H5]; staBck = Background SDO w/ STA-derived offset [H6]. SDO [H7].

SAT.predict.plot.error_rates.m

SAT.predict.plot.error_rates(***errorStruct***, saveFig, saveFormat, outputDirectory, plotProp):

<u>Utility</u>:

- Plot the Accumulating L0, L1, and L2 errors to evaluate state-dependent behavior between different hypotheses

<u>Inputs</u>:

- errorStruct: The common error prediction array generated by predictSdo.calcPredictError.m

- <u>Optional Name-Value Pairs</u>:
  - ‘saveFig’ - [0/1] Flag. Whether to automatically save the figure;
  - ‘saveFormat’ - [‘png’/’svg’]. Format for saving figure.
  - ‘outputDirectory’ - Where to save the figure; if not provided, will query the user for input.
  - ‘plotProp’ - The standard plotting properties structure, for color and line properties. If not provided will default to standard MATLAB colors

<u>Output</u>:

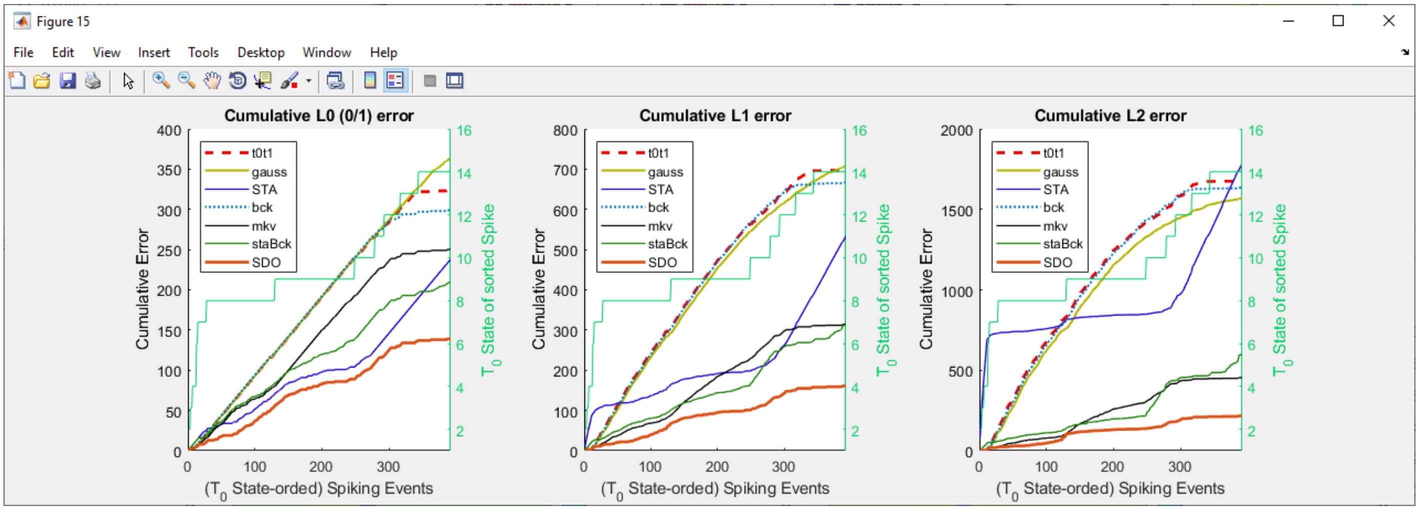

This figure shows the accumulating error between predicted and observed post-spike state, for each prediction hypothesis. Prediction errors are sorted by prespike signal state. This prespike state is given by the green step function, indexed on the right. Here, the SDO hypothesis outperforms the other hypotheses in all three metrics. The STA matches the SDO over the immediate states, but underperforms at higher and lower states. T0t1 = No change hypothesis [H1]; gaussH0 = gaussian convolution [H2]; STA = spike-triggered average Px [H3], bck = background SDO [H4]; mkv = First-order markov [H5]; staBck = Background SDO w/ STA-derived offset [H6]. SDO [H7].

SAT.predict.plot.relative_error_rates.m

SAT.predict.plot.relative_error_rates(**errorStruct, compCell**, saveFig, saveFormat, outputDirectory):

Utility:

Plot the delta-cumulative error rates between selected hypotheses.

Inputs:

- errorStruct:
  - Standard error structure
- compCell:
  - A {Mx2} cell containing strings/char matching the hypothesis names used in errorStruct; Each row (M) is a tested combination to plot.
- Optional Name-Value Pairs:
  - 'saveFig' -
    - [0/1] Flag.
    - Whether to automatically save the figure;
    - Default = 0
  - 'saveFormat' -
    - ['png'/'svg'].
    - Format for saving figure.
    - Default = 'png'
  - 'outputDirectory' -
    - Where to save the figure; if not provided, will query the user for input.
    - Default = “”

Output:

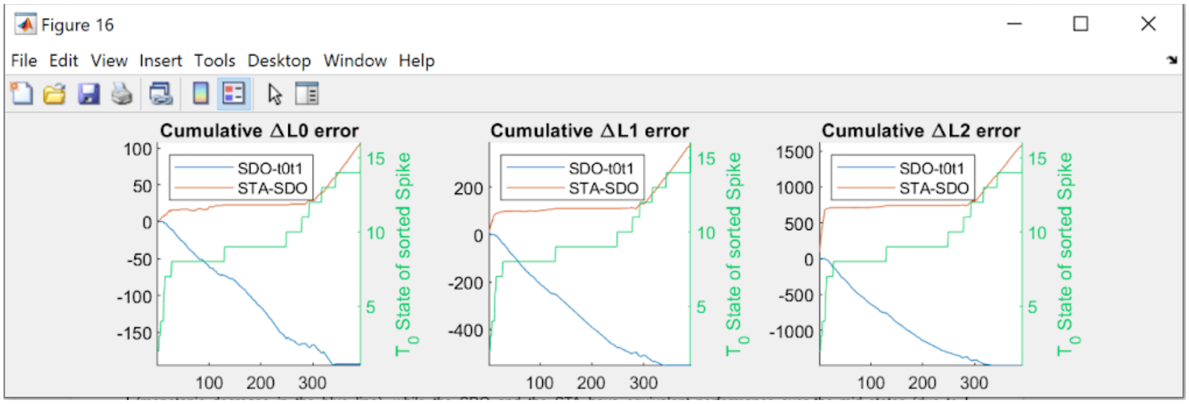

This figure shows the difference in the accumulating prediction error between selected hypotheses (here, the SDO vs. the STA, and SDO vs. the t0t1). As with the accumulating error plots, error rates are sorted by prespike state. Prespike state is plotted on as a green step function, and indexed on the right axis. Here, the SDO outperforms the t0t1 null hypothesis at virtually all states (monotonic decrease in the blue line), while the SDO and the STA have equivalent performance over the mid states (due to horizontal slope to the orange line).

SAT.predict.plot.pxDistance.m

SAT.predict.plot.pxDistance(**errorStruct, PXFIELD**, N_DRAWS, saveFig, saveFormat, outputDirectory, plotProp)

Utility:

- Plot violin plots of distributions of error states

Inputs:

- errorStruct: Standard error structure
- PXFIELD: Field within the error structure to plot
- Optional Name-Value Pairs:
    - N_DRAWS:
        - [Int].
        - Number of draws to use when bootstrapping the standard error
    - 'saveFig' -
        - [0/1] Flag.
        - Whether to automatically save the figure;
        - Default = 0
    - 'saveFormat' -
        - ['png'/'svg'].
        - Format for saving figure.
        - Default = 'png'
    - 'outputDirectory' -
        - Where to save the figure; if not provided, will query the user for input.
        - Default = “”
    - 'plotProp'
        - - The standard plotting properties structure, for color and line properties. If not provided will default to standard MATLAB colors

Output:

Plotting using the 'KLD' field.

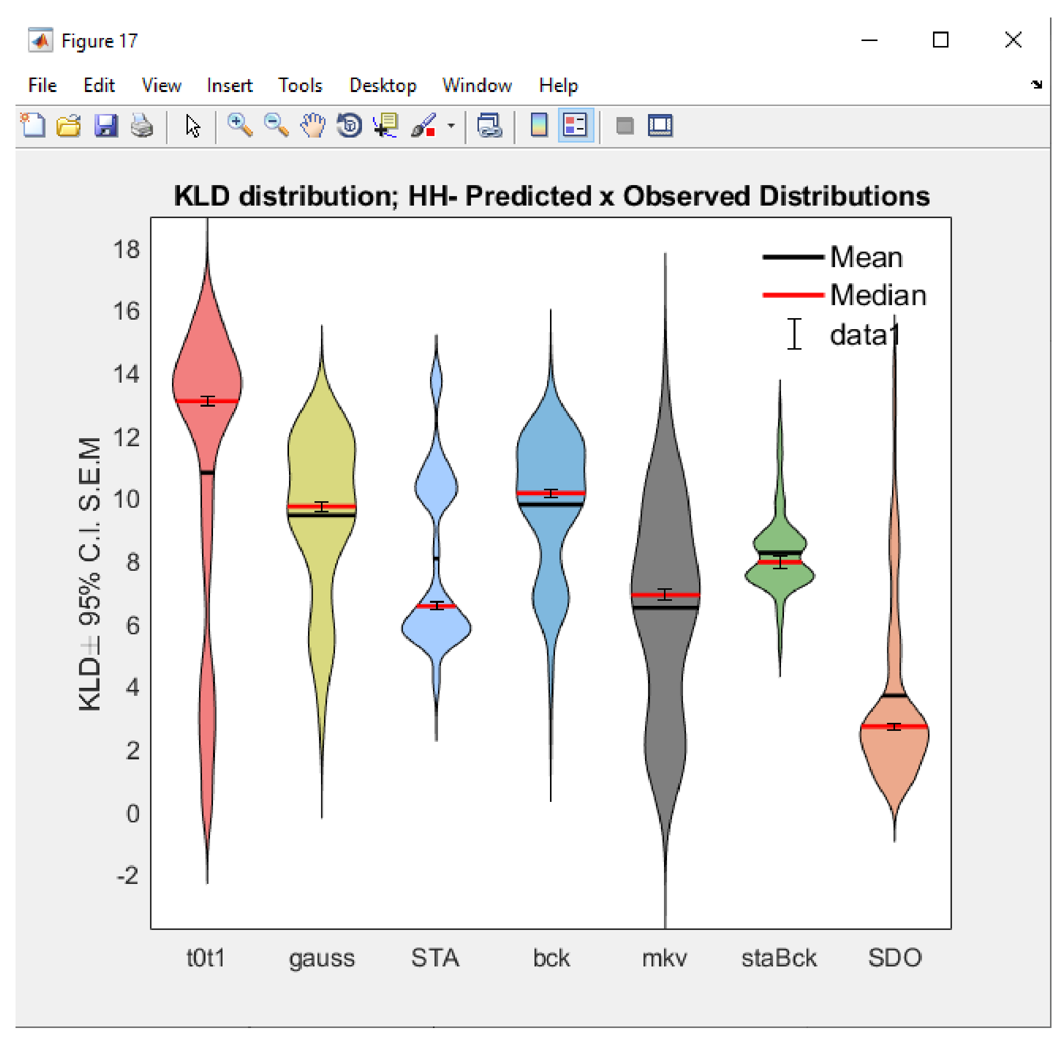

This is a violin plot of the KLD between the predicted and observed post-spike distributions, calculated over all spiking events as independent distributions. The mean (black) and median (red) of each distribution is given as a line. The 95% confidence interval is given as an error bar. Because the KLD is a measure of distance between two distributions, a smaller value indicates more similarity to the reference distribution. Here, the mean and median SDO prediction had less distance to the observed distribution, suggesting outperformance vs. the other hypotheses. T0t1 = No change hypothesis [H1]; gaussH0 = gaussian convolution [H2]; STA = spike-triggered average Px [H3], bck = background SDO [H4]; mkv = First-order markov [H5]; staBck = Background SDO w/ STA-derived offset [H6]. SDO [H7].

Plotting using the 'loglikelihood' field.

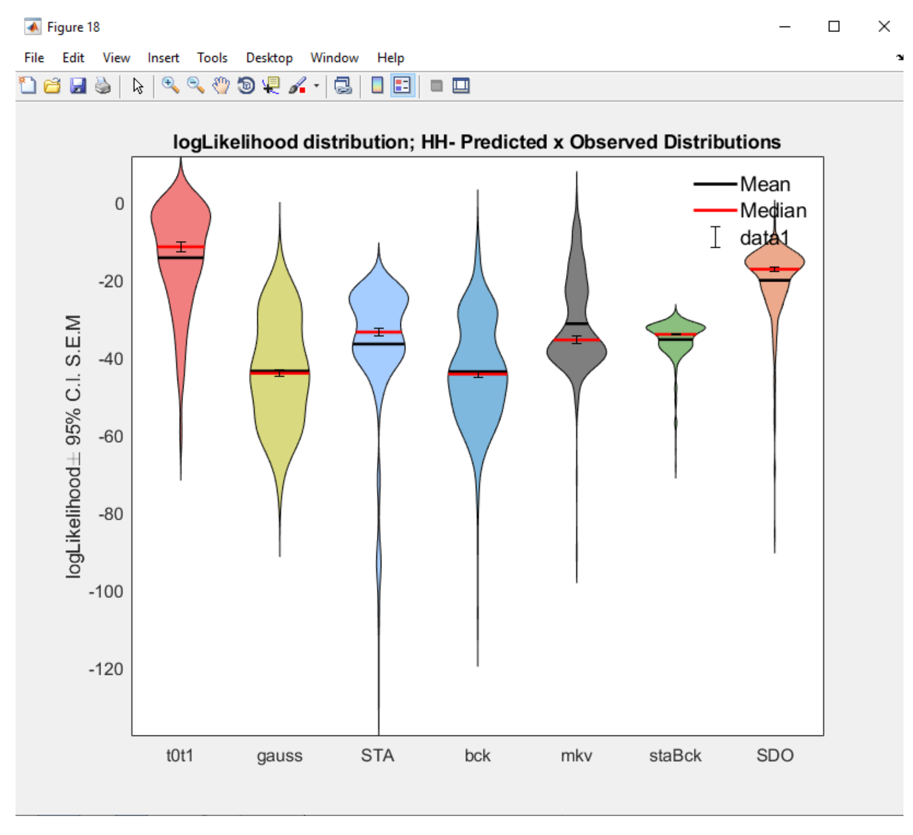

This violin plot shows the log-likelihood of the observed distribution, given each model (hypothesis) parameter estimations of probability of state. T0t1 = No change hypothesis [H1]; gaussH0 = gaussian convolution [H2]; STA = spike-triggered average Px [H3], bck = background SDO [H4]; mkv = First-order markov [H5]; staBck = Background SDO w/ STA-derived offset [H6]. SDO [H7].

IMPORTANT NOTES: The violin plots created here correspond to the <u>distribution of spike-wise error</u> statistics. (That is, the distribution of errors in the observed dataset). This is useful for determining if errors are normally distributed, or clustered. This is not the same as the significance of the means or medians *of* those distributions (tested below).

SAT.predict.testSig.m

SAT.predict.testSig(**errorStruct,** nShuffles,compRows,dataField,statType, saveFig, saveFormat, outputDirectory, plotProp)

<u>Utility</u>:

Tests the significance of the prediction-errors relative to each hypothesis. Performs *nShuffles* random paired-draws. Generates a statistic across each shuffle (i.e. sum, mean, median), then creates a distribution of these statistics. Tests the null hypothesis that the means of each distribution are the same. Significant differences in (any) means are tested by ANOVA, then followed up with multiple comparisons tests with P-value correction. Significant differences are often extreme, hence the empirical distribution functions and distributions of test statistics with confidence intervals are also plotted for intuition.

<u>Inputs</u>:

- errorStruct: Standard error structure
- <u>Optional Name-Value Pairs</u>:
  - ‘pVal’
    - Minimum threshold for significance.
    - Default = 0.05;
  - nShuffles’
    - Number of shuffles to use for establishing confidence intervals and significance.

        - Default = 1000
    - compRows
        - Row indices for comparing from the error structure, passed as a vector.
        - Default = 1: length(errorStruct);
    - dataField
        - Which test stat (field of *errorStruct*) to test.
        - Default = 'L1_running';
    - statType
        - How to derive a metric from each shuffle for building a distribution.
        - {'sum'/'mean'/'median'}
            - Summed error across the shuffled-error
            - Mean error across the shuffled-error
            - Median error across the shuffled-error
        - Default = 'sum';
    - 'saveFig' -
        - [0/1] Flag.
        - Whether to automatically save the figure;
        - Default = 0
    - 'saveFormat' -
        - ['png'/'svg'].
        - Format for saving figure.
        - Default = 'png'
    - 'outputDirectory' -
        - Where to save the figure; if not provided, will query the user for input.
        - Default = ""
    - 'plotProp'
        - - The standard plotting properties structure, for color and line properties. If not provided will default to standard MATLAB colors

Outputs:

- anova_cll
    - {1 x N Hypotheses} Cell array containing the tabular output of a 1-way ANOVA between shuffled distributions.
- Multcomp_cll
    - {1 x N_Hypotheses} Cell array containing the post-hoc multiple comparisons tabular output data.

Plotting using the 'L1_running' field

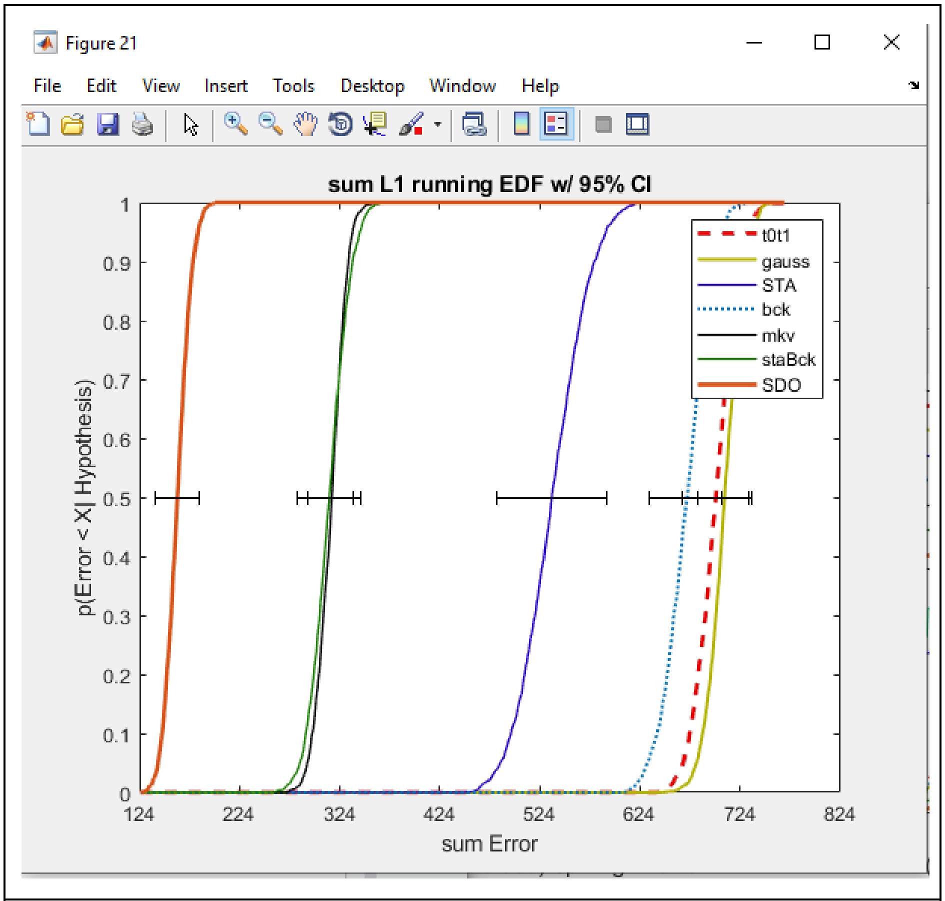

Plotted Empirical Distribution Functions (EDF) for the distribution of test statistics (here, sum) across all shuffles, using the 7 hypotheses. 95% Confidence Intervals are plotted as horizontal bars around the means of the respective EDFs. (Non-overlapping 95% CI bars indicate significant differences in using a pValue of 0.95). Here, the SDO has a significantly lower summed error. H6 vs. H5 hypotheses do not have significantly different summed errors. T0t1 = No change hypothesis [H1]; gaussH0 = gaussian convolution [H2]; STA = spike-triggered average Px [H3], bck = background SDO [H4]; mkv = First-order markov [H5]; staBck = Background SDO w/ STA-derived offset [H6]. SDO [H7].

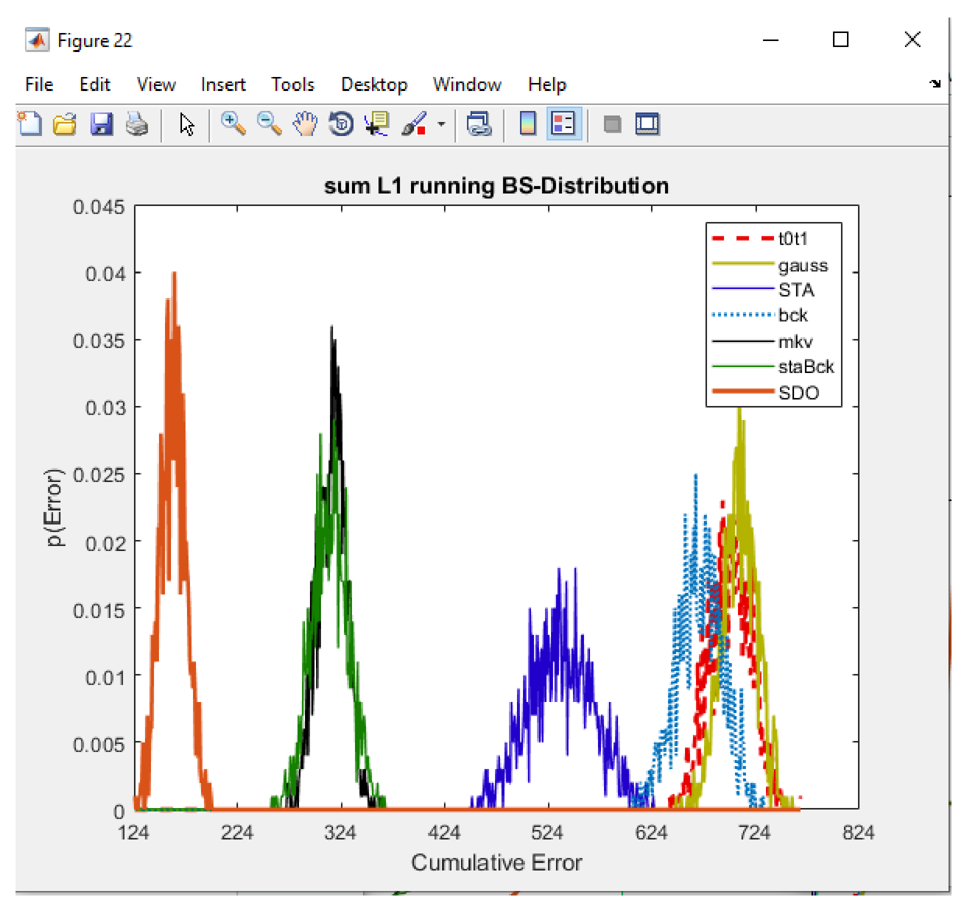

Error probability distributions by hypothesis (effectively the difference of the EDF). Due to 1000 bootstraps, error statistics distributions are roughly normal and significant differences in the may be assessed using standard tests (i.e. t-test). P-values of differences of the mean correspond to the overlap between the observed hypothesis error vs. the shuffled distribution of other hypotheses. (Generally the observed error is equivalent to the mean of the respective shuffled distribution.) Here, the SDO distribution is completely isolated from other hypothesis, indicating an extreme level of significance. (A P-value here will be ill defined, given how extreme along the tails the next hypothesis is.)T0t1 = No change hypothesis [H1]; gaussH0 = gaussian convolution [H2]; STA = spike-triggered average Px [H3], bck = background SDO [H4]; mkv = First-order markov [H5]; staBck = Background SDO w/ STA-derived offset [H6]. SDO [H7].

IMPORTANT NOTES:

- This method of deriving significance should be robust across different prediction metrics, including predictions to state and predictions to distributions. However, it is important to remember that the shuffled distributions displayed here correspond to the metric across the shuffled distribution, not the elements of the shuffle directly. I.e. While distributions of spike-wise error may overlap, the distributions of the *mean* of those spike-wise errors may not. (E.g. Overlapping distributions of spike-wise error shown by the violin plots do not mean that the distributions of the *means* of those distributions will overlap).

Supplemental Libraries for manipulating probability distributions and SDOs:

pxTools Library:

A small collection of scripts, functions, and methods for dealing with probability distributions, and collections of probability distributions, represented as column vectors.

Scripts contained within the pxTools Library are called throughout the *SDO Analysis Toolkit*, but may also be used as standalone scripts for gathering and manipulating probability distributions from *xtData* and *ppData* data holder structures.

pxTools.getXtStateMap.m

pxTools.getXtStateMap(**xtData**, **N_BINS**, fieldname, mapMethod, maxMode)

Utility:
- Assigns the edge vector used for binning state, for each channel of the provided *xtData*.
- Generates and populates the **.signalLevels** field within *xtData*.

Input:
- xtData
  - An *xtData* data holder, containing trialwise observations of signal amplitude.
- N_BINS
  - [integer]
  - The number of signal states to use.
- Optional Name-Value Pairs:
  - ‘fieldname’
    - The fieldname within *xtData* to use as signal amplitude.
    - Default = ‘envelope’
  - ‘maxMode’
    - {‘pTrial’, ‘pTrialxSeg’, ‘‘xTrialxSeg’}
    - The method for determining the maximal value of signal amplitude for defining signal state. pTrial methods define state independently for each trial (i.e. signal amplitude state definitions may vary across trials).
    - Default = ‘xTrialxSeg’
  - ‘mapMethod’
    - {‘linear’, ‘log’}
    - The method for assigning state bins from minimum and maximum observed signal amplitudes. ‘Log’ distributes state bins in log space.
    - Default = ‘log’

Output:
- sigLevelCell
  - A {N_XT_CHANNELS x N_TRIALS} cell array, which contains the definition of state (a [1xN_BINS+1] vector) for each time series data channel, for each trial.
- xtData
  - The input *xtData* structure, with new field
    - **.signalLevels**: The [1 x N_BINS+1] edge vector containing the definition of signal state.

IMPORTANT NOTES:

- If only a single output argument is defined, only sigLevelCell will be passed.
- 'xTrialxSeg' is the recommended method for determining states from signal amplitude.
- The edge vectors defined by this function can be directly passed into MATLABs *discretize* function to build a rasterized state-signal from the matching time-series data.

pxTools.getPxtFromXt.m

pxTools.getPxtFromXt(**xt, st, signalLevels**, navg, smoothwid, smoothstd, n_shift, z_delay)

<u>Utility</u>:

- Core method to convert time series data and positional indices (event times) into column vector probability distributions of state.

<u>Input</u>:

- xt:
  - [1 x M] vector
  - Contains signal amplitude
- st
  - [1 x N] vector
  - Contains positional indices to query xt at. In the SDO analysis framework, these correspond to spiking/event times.
  - May also be set to 'all', which will return a probability vector for the set properties for *every* possible time index of xt.
- signalLevels
  - [1 x N_BINS+1] Vector
  - Contains the edge vector defining N_BINS states for the signal in xt.

<u>Optional Name-Value Pairs</u>:

- 'navg'
  - [1x 2] Vector (of integers)
  - The first element is the number of points prior to event time (-1) to use for the pre-spike distribution.
  - The second element is the number of points after event time to use for the post-spike distribution.
  - Note that these points are in the dimension/duration of xt.
  - Default = [20, 20];
- 'smoothwid'
  - [int]
  - The number of states to calculate the smoothing exponential kernel over for filtering the observed state distributions.
  - NOTE: This is the number of points (states) over which the kernel is calculated relative to the mean, but is not directly used in the calculation of the gaussian sigma (but because probability distributions are summed to 1 after filtering, this may indirectly influence p(x) amplitude.
  - Default = 0
- 'smoothstd'
  - Double > = 0
  - Standard deviation of the *filtfilt* gaussian smoothing kernel.
  - Default = 0
- 'n_shift'
  - [int]

- The anchoring point for drawing pre-spike/post-spike distributions relative to xs; defined from the relative position of the start of the post-spike interval relative to the time bin containing spike.
    - Default = 1;
- ‘z_delay’
    - [int]
    - The number of time bins to skip between the end of the prespike interval and the start of the post-spike interval.
    - Default = 0;

Outputs:
- px_t0
    - A [N_BINS x N_SPIKES] doubles vector, containing the pre-spike probability column vectors indexed by the order of elements in xs.
- px_t1
    - A [N_BINS x N_SPIKES] doubles vector, containing the post-spike probability column vectors indexed by the order of elements in xs.
- ind_t0
    - A [N_X0_POINTS x N_SPIKES] integer vector, containing the positional indices of the components of xs used in the pre-spike distribution.
- ind_t1
    - A [N_X1_POINTS x N_SPIKES] integer vector containing the positional indices of the components of xs used in the post-spike distribution.

IMPORTANT NOTES:
- If only 2 outputs are defined, only px_t0 and px_t1 will be passed.
- Raw probability distributions of observed data (no filtering) can be used by setting ‘smoothwid’ and ‘smoothstd’ to 0.
- To capture a single pre/post-spike state, an element ‘navg’ can be set to 0. If both navg = [1,1], the returned distribution is effectively a markov process.

pxTools.getXfromPx.m

pxTools.getXfromPx(**px**, method)

Utility:
- Assigns a single state to the queried column-wise probability distributions (or a structure containing these columnwise distributions as fields)

Inputs:
- px
    - [N_BINS x N_SPIKES] doubles array, containing column-wise probability vectors.
    - If px is a structure, define a single state for all fields.
- method
    - {‘max’, ‘mean’, ‘median’}
    - How to assign a single state to the distribution. In all cases, the returned value is a single state (integer) matching the closest metric.
    - Default = ‘max’

Outputs:
- X
    - [1 x N_SPIKES] integer vector, containing the assigned state value.
    - If px is a structure, X is a structure with matching fields.

pxTools.getTrialwisePxt.m:

pxTools.getTrialwisePxt(**xtData, ppData**, trList, xtList, ppList, xtDataField, pxNPoints, pxFilter, pxShift, pxDelay)

<u>Utility</u>:

- Using provided *xtData* and *ppData*, get probability distributions for each combination of xtDataChannel and ppDataChannel, over all trials.
- Used to quickly extract all combinations of spike/signal within *computeSDO.m*

<u>Inputs</u>:

- xtData
    - Standardized xtData containing the time series data
- ppData
    - Standardized ppData containing the point-process data
- OPTIONAL POSITIONAL PARAMETERS
    - trList
        - Vector/list
        - Contains the list of trials to use (column indices of xtData and ppData)
        - If not provided, defaults to all trials.
    - xtList
        - Vector/list
        - Contains the list of xtDataChannels to use (row indices of each trial of xtData).
        - If not provided, defaults to all rows.
    - ppList
        - Vector/list
        - Contains the list of ppDataChannels to use (row indices of each trial of ppData).
        - If not provided, defaults to all rows.
- OPTIONAL NAME-VALUE PAIR ARGUMENTS
    - xtDataField
        - String/Character
        - The name of the field containing the time series data in *xtData*, for each trial used.
        - If not provided, it defaults to ‘envelope’.
    - pxNPoints
        - [1xN] Integer
        - First component is the width of the prespike interval, second component is the width of the post-spike interval, in bins/indexed positions.
        - Default = [20,20]
    - pxFilter
        - [1xN] Double
        - First component is the number of bins to estimate gaussian smoothing kernel over relative to the reference bin; PX_FSM_WID
        - Second component is the standard deviation of the gaussian smoothing kernel, in states.
        - Default = [0,0]; (No filtering)
    - pxShift
        - Integer
        - The starting position of the starting position for splitting the pre-spike/post-spike bins relative to the occurrence of spike.
        - Default =1 (Spike is the last element of pre-spike interval).
    - pxDelay

    - Integer
    - The duration between the end of the pre-spike interval and the start of the post-spike interval, in bins.
    - Default = 0

Outputs:
- pxt0Cell
  - {1 x NTrials} Cell matrix.
  - Each element contains a {nXtChannels x nPpChannels} cell, containing observed column vectors of probability distributions of the prespike state, collected for the observed spikes, as indexed by the xtDataChannel and ppDataChannel.
- pxt1Cell
  - {1 x NTrials} Cell matrix.
  - Each element contains a {nXtChannels x nPpChannels} cell, containing observed column vectors of probability distributions of the postspike state, collected for the observed spikes, as indexed by the xtDataChannel and ppDataChannel.
- iXt0Cell
  - {1 x NTrials} Cell matrix.
  - Each element contains a {nXtChannels x nPpChannels} cell, containing observed column vectors of indexed prespike position within the time series data.
- iXt1Cell
  - {1 x NTrials} Cell matrix.
  - Each element contains a {nXtChannels x nPpChannels} cell, containing observed column vectors of indexed postspike position within the time series data.
- at0Cell
  - {1 x NTrials} Cell Matrix
  - Each element contains a {nXtChannels x nPpChannels} cell, containing the observed column vectors of (raw) signal amplitude within the indexed pre-spike amplitude within the time series data.
- at1Cell
  - {1 x NTrials} Cell Matrix
  - Each element contains a {nXtChannels x nPpChannels} cell, containing the observed column vectors of (raw) signal amplitude within the indexed post-spike amplitude within the time series data.

pxTools.getCovArrFromJointArr.m

pxTools.getCovArrFromJointArr(**arr**, OP_DIM)

Utility:
- Converts an input joint array (p(x1|x0) to either p(x0|x0) or p(x1|x1)
- Provides an approximation of the expected covariance array

Inputs:
- arr
  - The joint SDO.
- OP_DIM
  - Operational dimension for extracting the probability distribution.
  - Default = 1; (Post-spike distributions are arranged column-wise)

Output:
- covArr
  - The predicted output covariance matrix.

IMPORTANT NOTES:

- The predicted covariance array from the joint SDO (itself calculated from the average of spike-averaged effects) is NOT the joint SDO which would result from the pre-spike state distributions.

pxTools.getSdoFromJointArr.m

pxTools.getSdoFromJointArr(**arr**)

Utility:
- Converts an input joint array into dp(x1|x0), a gross approximation of the differential SDO matrix.

Inputs:
- arr
  - The joint SDO matrix to estimate from.

Output:
- sdoMat
  - The approximated differential SDO matrix.

IMPORTANT NOTES:
- The predicted differential SDO array from the joint SDO (itself calculated from the average of spike-averaged effects) is NOT the differential SDO which would result from the pre-spike state distributions.
- Thus, the result is only an approximation of the true SDO.

pxTools.getH0Array.m

pxTools.getH0Array(**N_BINS**, **PX_FSM_WID, PX_FSM_STD**, ‘type’)

Utility: Produce an identity matrix or filtered identity matrix as a transition matrix; used for the null hypotheses.

Inputs:
- N_BINS
  - Integer
  - Number of state bins to use
- PX_FSM_WID
  - *Not required*.
- PX_FSM_STD
  - Integer
  - The size of the filtering bin used for the H0 array.
  - If 0, the returned result will be an identity matrix.
  - If > 0, the returned matrix will be a filtered identity matrix.
- ‘type’
  - {‘M’/’L’}
  - Switch between the Markov-like transition matrix (M) or the change-of-probability matrix, the linear operator (L).
  - $M - 1 = L$
  - Default = M.

Outputs:
- h0Array
  - A [N_BINS x N_BINS] transition matrix, generating the specified parameters.
  - If ‘type’ == M; each column sums to 1.
  - If ‘type’ == L; each column sums to 0.

pxTools.getMarkovFromXt.m

pxTools.getMarkovFromXt(**xtArr, N_BINS**, 'conform', 'order', 'Normalization')

Utility: Calculate the first-order Markov matrix from subsequent observations of state-state transitions.

Inputs:

- xtArr
    - A NxM matrix of integer values (corresponding to state ID.
    - State-state transitions are observed between rows. Independent sets of observations are arranged as different columns (as may be collected by *pxTools_getTrialwisePxt.m*). If using this method, columns must be of equal size.
    - Alternatively, this may be a 1xN timeseries envelope.
- N_BINS
    - Integer
    - Maximum number of states to use; order of the output Markov Matrix
    - If not provided, the maximum observed value in the xtArr will be used.
- Optional Name-Value Pairs:
    - 'conform'
        - [0/1]
        - Whether the output Markov Matrix should be fully defined for all states (including non-observed), or not.
    - 'order'
        - [0/1]
        - Order of the Markov Matrix Chain (i.e. how many steps back to incorporate into memory).
        - Reserved for future use, no current function.
    - 'Normalization'
        - {'array', 'column', 'none'}
        - How to normalize the recovered matrix.
            - 'array' : The whole matrix will be summed to 1.
            - 'column' : Each column will be summed to 1.
            - 'none': No normalization used; matrix will reflect transition frequency.
- Output:
    - mkv = Resultant Markov Matrix

pxTools.KLDiv.m

pxTools.KLDiv(**p1, p2**, flag)

Utility: Returns the Kullback-Leibler Divergence (KLD) calculated between two probability distributions. The KLD is a parameterized distance between a reference and query distribution.

Inputs:

- p1
    - Query Probability distribution.
    - Can be the SDO matrices, or column-vectors of observed distributions.
- p2
    - Reference Probability distribution.
    - Can be the SDO matrices, or column-vectors of observed distributions.
- flag
    - {empty},Int
    - Dimensional Direction for calculating the KLD, if P1 and P2 are multidimensional.

- If not defined, p1 and p2 are vectorized as single distributions (a single KLD is calculated).

Output:

- KL
  - A measure of the KLD, calculated over the dimensions, as provided.

IMPORTANT NOTES:

- The KLD is not symmetrical in both directions; $KLD(A, B) \neq KLD(B, A)$.
- Thus, comparisons between different hypotheses/methods need to be made against the same reference distribution to be meaningful.

pxTools.matrixRandomWalk.m

pxTools.matrixRandomWalk(**mat, N_STEPS, N_SIM**, ‘startVal’)

Utility: Simulate a specified number of random walk steps, for a given number of simulations, given a transition matrix.

Inputs:

- mat
  - The first-order square transition matrix containing the probability of consecutive state.
  - This should be column-normalized to 1.
- N_STEPS
  - Integer
  - The number of subsequent observations to draw.
- N_SIM
  - The number of simulations to make N_STEPS observations of.
- ‘startVal’
  - If supplied as an integer, treated as the single starting state for each simulation.
  - If supplied as a vector/distribution, the starting state for each simulation is drawn from this distribution.

Outputs:

- randWalkMat:
  - A [N_SIM x N_STEPS] array of integers, corresponding to subsequent state draws, under the assumption of a random walk (Events are independent).

pxTools.splinePx.m

pxTools.splinePx(**pxIn**, OP_DIM)

Utility: Splines a probability distribution (or array of distributions), to ensure that there are no occurrences of ‘0’ in the overall state-state transitions.

Inputs:

- pxIn
  - [M x N] array containing a probability distribution.
  - Probability vectors within the array may be oriented either direction, but are defaulted as column vectors, appended together.
- OP_DIM
  - The operational dimension along which probability vectors are considered.
  - Default = 1 (probability vectors are columnwise).

Outputs:

- pxOut
  - [M x N] array containing the splined probability distribution.

IMPORTANT NOTES:

- Uses the MATLAB ‘pchip’ function to spline the resultant probability distributions, which may result in sub-optimal fitting.

pxTools.predictPxtFromPx0.m

pxTools.predictPxtFromPx0(**mat, px0,** N_INTERVALS)

Utility: Predicts a post-spike distribution over some defined interval after spike, given an input distribution.

Inputs:

- mat
    - The transition matrix used for prediction.
    - May be either the ‘markov’ transition format (M) or the linear operator format (L)
- px0
    - [N_BINS x N_Observations] matrix containing the observed (pre-spike) probability distributions to predict from.
- N_INTERVALS
    - The number of steps forward to predict, given the supplied matrix.
    - Default = 1.

Outputs:

- pxt
    - [N_BINS x N_Observations] matrix containing the predicted post-spike distributions N_INTERVALS steps forward.

IMPORTANT NOTES:

- This is the core method for predicting probability distributions, given a transition matrix and the probability vector.

pxTools.getPxTE.m

pxTools.getPxTE(**px0**, **L**, **N_STEPS**, scaleArr)

Utility: Utility to generate a prediction of Px over N_STEPS using an initial probability distribution and a linear (L) update operator (SDO) under the framework px1 = x0 + dx(0), where dx0 is calculated by an update matrix.

Inputs:

- pxt0
    - A [Nx1] Initial probability distribution.
    - May be binary.
    - Must sum to 1.
- L
    - Either a:
        - 1) [NxN] scaled/normalized SDO matrix.
        - 2) {Mx1} cell array containing M [NxN] SDO matrices
- N_STEPS
    - Int.
    - Number of steps to make (predict), including point px0.
- scaleArr
    - A [MxN_STEPS] doubles array containing non-negative real-valued scaling amplitudes for sdo matrix at each time step.
    - If omitted, or Dimension 2 < N_STEPS, missing values will be treated as 1 (SDO active).

Outputs:

        - pxTE
            - A [N x N_STEPS] doubles array containing the projected probability distribution for each time step.

IMPORTANT NOTES:
- If the supplied SDOs are non-conforming, the predicted probability distributions are not guaranteed to sum to 1.
- scaleArr can handle any non-negative value. It used to directly scale SDO effects at the given time point. Negative values will cause the SDO to no longer remain compliant.

pxTools.xvect2px.m

pxTools.xvect2px(**xt**, MAX_X)

Utility: Converts a state (integer) signal into a binary probability vector for each time point.

Inputs:
- xt
    - [1xN] doubles vector, composed of integer values.
- MAX_X
    - The maximum state number to evaluate probability distributions for.
    - If not provided, will default to the maximum observed state (value)

Outputs:
- px
    - An [MAX_X x N] output binary vector, for every indexed state position observed in xt.

Auxiliary Functions:

SAT.sdoUtils.quicksdo.m

SAT.sdoUtils.quicksdo(**px0**, **px1**)

- Utility:
    - Quickly estimate the SDO matrix and pre/post spike covariance given the pre/post spike state distribution, without the rest of the *sdo* structure.
- Prerequisites:
    - None
    - (Probability distributions may be drawn from *xtData* and *ppData* using the pxTools library)
- Dependencies:
    - None
- Input Arguments:
    - *px0*:
        - A [nStates x nObservations] array, corresponding to pre-spike probability state distributions as column vectors.
    - *px1*
        - A [nStates x nObservations] array, corresponding to the post-spike probability state distributions as column vectors.
- Output Arguments:
    - *sdoMat*:
        - The [nStates x nStates] array, corresponding to the pre/post-spike SDO matrix.
        - If only a single output is queried, only *sdoMat* is returned.
    - *covMat*:
        - The [nStates x nStates] array, corresponding to the px0→ px1 transition matrix.

SAT.sdoUtils.islinearsdo.m

SAT.sdoUtils.islinearsdo(**L**)

- Utility:
    - Quick test for if the matrix satisfices the definition of a linear operator, L
- Prerequisites:
    - None
- Dependencies:
    - None
- Input Arguments:
    - L
        - Square matrix to test for linearity.
- Output Arguments:
    - Flag
        - Boolean [0/1].
        - 0 = L fails to suffice requirements for linearity
        - 1 = L satisfies requirements for linearity

SAT.sdoUtils.normsdo.m

SAT.sdoUtils.normsdo(**dSDO, jSDO,** dNSdo, jNSdo, normMat, method)

- Utility:
    - Normalizes the differential SDO using the joint SDO, a prerequisite to combination and forward prediction.
- Prerequisites:
    - None
- Dependencies:
    - *Normpdfcol2unity.m*
- Input Arguments:
    - dSDO:
        - [NSTATES x NSTATES] doubles array
        - The differential SDO to normalize
    - jSDO
        - [NSTATES x NSTATES] doubles array
        - The joint SDO to use for normalization.
    - Optional:
        - dNSdo
            - [NSTATES x NSTATES] doubles array
            - The normalized differential SDO
        - jNSdo
            - [NSTATES x NSTATES] doubles array
            - The Normalized joint SDO
        - normMat
            - A [NSTATES x NSTATES] array, which served as the denominator of element-wise division of the jSDo and dSDO matrices.
        - method: {'px0', 'px1px0', 'unity'}
            - Normalization Method;
            - Default = 'px0'

SAT.sdoUtils.conformsdo.m

SAT.sdoUtils.conformsdo(**sdoMat**):

Utility:
- Conforms an input differential SDO matrix to fit the requirements necessary for the SDO to behave as a linear operator.

Prerequisites:
- None

Dependencies:
- None

Input Arguments:
- sdoMat
    - A differential SDO matrix.
    - → In principle, any square matrix could be input and ‘conformed’ thusly (although what this represents would be questionable).

Output:
- L
    - A Linear Operator, fully compliant with the assumptions of Sanger.

SAT.estPxParams.m:

SAT.testPxParams()

Utility: A simple utility script to test different parameters on the resultant background joint probability distribution of state, prior to complete SDO Analysis.

Prerequisites:
- None

Dependencies:
- None

Input Arguments:
- {None}

Output:
- {None}

IMPORTANT NOTES:
- The header variables used in this script (intentionally) match the same header variables used in the *computeSDO*.m. This permits different parameters to be tested prior to doing the complete SDO Analysis.

trimdatacell.m:

trimdatacell(**dc**, TRIAL_LIST, CHANNEL_LIST)

Utility: Trim a provided data structure holder to a specified list of trials to use, and channels to use. May be used to carve down given data structure holder into a subset of interesting data structure holder for performing analysis.

Prerequisites:
- None

Dependencies:
- None

Input Arguments:
- dc
    - An input *dataCell*, which may be an *xtData*or *ppData*

- TRIAL_LIST
    - Integer/Integer Vector
    - List of trials (dataCell columns) to retain.
    - Must be defined, or left as an empty set (‘[]’)
    - If left empty, all trials are retained.
- CHANNEL_LIST
    - Integer/Integer Vector
    - List of Channels (dataCell rows, within the top row of the *dataCell* element, structure arrays) to retain.
    - If not defined, all channels are retained.

ssta_vs_sdo.m:

ssta_vs_sdo()

Utility: A standalone script which demonstrates the need for the SDO by showing the STA, mean-split STA and the distributions associated w/ it.

Prerequisites:

- None

Dependencies:

- PxTools_Library

Input_Arguments:

- {None}

Header Arguments:

- USE_TRIALS:
    - [1 x N] Vector of Integer
    - Corresponds to the Trial Indexed positions within the xtData
- XT_CH_NO:
    - Integer.
    - Trial Index position for time series data to use.
- PP_CH_NO:
    - Integer.
    - Trial Index position for point-process data to use.
- N_T0_PTS:
    - Integer.
    - Number of points to use on either side of spike for drawing pre/post spike intervals.
    - Default = 20
- N_STATES:
    - Integer.
    - Number of signal ‘states’ to define from time series signal amplitude
    - Default = 20
- MAX_MODE:
    - ‘xTrialxSeg’
    - Method to determine max/min signal amplitude across datasets for determining states
- MAP_METHOD:
    - {‘log’/’linear’}
    - Default ‘log’
    - How to build state intervals from min/max signal amplitude
    - Default = ‘log’
- DATA_FIELD:
    - Structure field name containing time series data within *xtData*.

- Default = ‘envelope’
- COMPOSITE:
  - Logical [0/1]
  - If 1; Re-assemble plotted figures as subplots on a new figure.
  - Note that legends are not redrawn in the subplots, which may lead to ambiguity in interpretation.
  - Default = 1

Output:

- 2x Figures, corresponding to the spike-triggered and shuffled-time figures

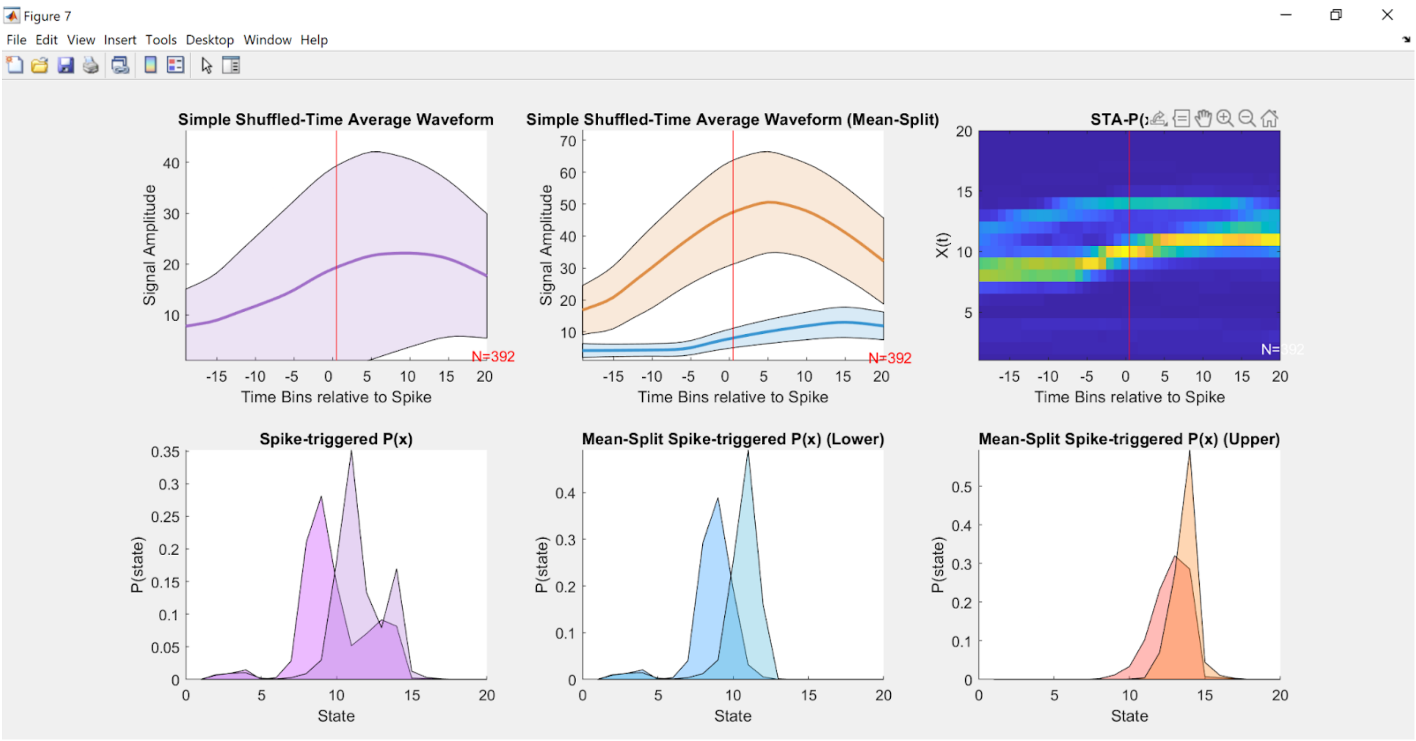

Composite figure demonstrating different plotted relationships between spikes and signals. Top Left: An overall spike-triggered average waveform between spike and signal, +/- 1 standard deviation. Top Middle: The STA waveform from signal collected above/below the mean of the signal at time of spike. Top Right: The spike-triggered impulse response probability distribution of state. Bottom left: The prespike/postspike probability distribution of state, averaged over all waveforms. Bottom Middle: The prespike/postpike distribution of state from spiking events which occur in the lower half of observed spiking signal states. Bottom Right: The prespike/postpike distribution of state from spiking events which occur in the upper half of observed spiking signal states.

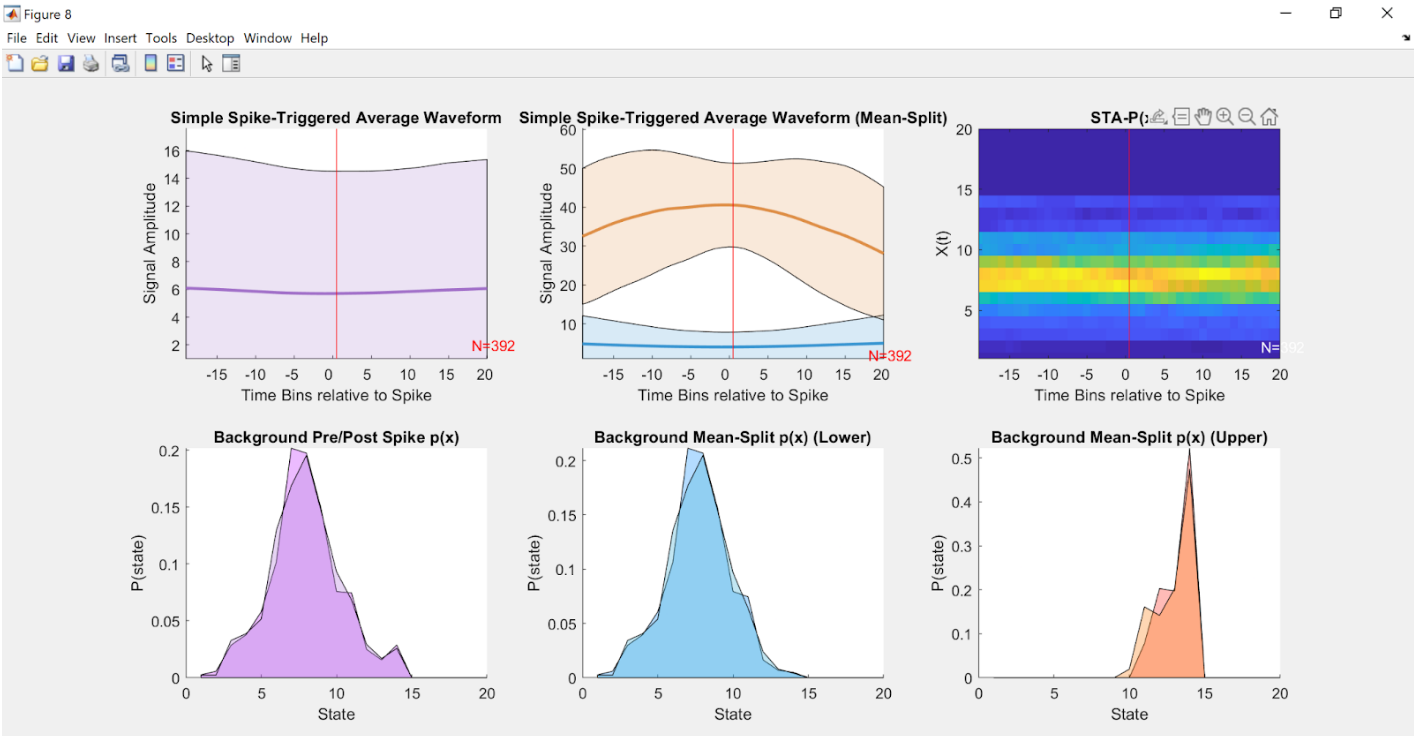

Equivalent plots for an equivalent number of randomly-drawn spikes. These figures indicate that there are signal dynamics inherent in the signal, uncorrelated to spiking events.

REFERENCES:

1. Sanger TD (2010) Controlling variability. J Mot Behav. 42(6)
2. Sanger TD (2011) Distributed control of uncertain systems using superpositions of linear operators. Neural Comput 23(8):1911-34
3. Sanger TD (2014) Risk-aware control. Neural Comput. 26(12)
