# Supplementary material for: A Stochastic Dynamic Operator Framework That Improves the Precision of Analysis and Prediction Relative to the Classical Spike-Triggered Average Method, Extending the Toolkit
Source: eNeuro. 2024 Nov 5;11(11):ENEURO.0512-23.2024. doi: 10.1523/ENEURO.0512-23.2024 (PMC11552545; doi:10.1523/ENEURO.0512-23.2024)
Supplement: SDO Analysis Toolkit — Download SDO Analysis Toolkit, ZIP file. [file eneuro-11-ENEURO.0512-23.2024-s001.zip › sdoAnalysis_20240821/sat-master/external/hex2rgb/rgb2hex_and_hex2rgb_documentation/html/rgb2hex_hex2rgb_demo.html]

rgb2hex and hex2rgb 

# rgb2hex and hex2rgb

These functions convert RGB color triplets to hexadecimal format, or hexadecimal format to RGB color triplets.

## Contents

- Syntax
- Description
- Examples of rgb2hex
- Examples of hex2rgb
- Author Info

## Syntax

```
rgb2hex(rgb)
hex2rgb(hex)
hex2rgb(hex,range)
```

## Description

rgb2hex(rgb) converts RGB color triplets to hexadecimal format. If no value in the rgb matrix exceeds unity, values are assumed to be scaled from 0 to 1. If any value in rgb exceeds unity, values are assumed to be scaled from 0 to 255.

hex2rgb(hex) converts hexadecimal to rgb color triplets scale from 0 to 1.

hex2rgb(hex,range) specifies a range as 1 or 256 for scaling output RGB values. A previous version of this function required a range of 255 when scaling from 0 to 255, but letting range equal 256 may be more intuitive, so either input will work now. Whether you enter a range of 255 or 256, RGB values will be scaled from 0 to 255. Default range is 1 to Matlab syntax, meaning values are scaled from 0 to 1.

## Examples of rgb2hex

What is the hexadecimal value of pure green?

```
greenHex = rgb2hex([0 1 0])
```

```
greenHex =

#00FF00
```

What if the rgb values are scaled from 0 to 255?

```
greenHexIsStill = rgb2hex([0 255 0])
```

```
greenHexIsStill =

#00FF00
```

This function works for multiple entries too:

```
myrgbvalues = [.2 .3 .4;
               .5 .6 .7;
               .8 .6 .2;
               .2 .2 .9];
myhexvalues = rgb2hex(myrgbvalues)
```

```
myhexvalues =

#334D66
#8099B3
#CC9933
#3333E6
```

Or similarly,

```
rgb2hex(jet(5))
```

```
ans =

#0080FF
#00FFFF
#80FF80
#FFFF00
#FF8000
```

## Examples of hex2rgb

Say some online color program gives you some hex value that you'd like to use in your next Matlab plot. The number '#334D66' is something that Matlab can understand, so we use hex2rgb:

```
hex2rgb('#334D66')
```

```
ans =

    0.2000    0.3020    0.4000
```

The hex2rgb function may also be used inside a plot command:

```
plot(1:10,(1:10).^2,'color',hex2rgb('#334D66'),'linewidth',5)
```

The pound sign is optional:

```
myrgbvalue = hex2rgb('334D66')
```

```
myrgbvalue =

    0.2000    0.3020    0.4000
```

Values may be scaled from 0 to 255:

```
myRGBvalue = hex2rgb('#334D66',256)
```

```
myRGBvalue =

    51    77   102
```

Input hex values can be in a matrix:

```
myhexvalues = ['#334D66';'#8099B3';'#CC9933';'#3333E6'];
myrgbvalues = hex2rgb(myhexvalues)
```

```
myrgbvalues =

    0.2000    0.3020    0.4000
    0.5020    0.6000    0.7020
    0.8000    0.6000    0.2000
    0.2000    0.2000    0.9020
```

Input hex values may also be in a character array:

```
HexValsAsACharacterArray = {'#334D66';'#8099B3';'#CC9933';'#3333E6'};
rgbvals = hex2rgb(HexValsAsACharacterArray)
```

```
rgbvals =

    0.2000    0.3020    0.4000
    0.5020    0.6000    0.7020
    0.8000    0.6000    0.2000
    0.2000    0.2000    0.9020
```

Character arrays can be useful when plotting and labelling:

```
x = 1:4;
y = -x;
scatter(x,y,1e3,rgbvals,'filled')
axis([0 5 -5 0])
text(x,y,HexValsAsACharacterArray,'horizontalalignment','center')
```

## Author Info

These functions were written by Chad A. Greene of the University of Texas at Austin's Institute for Geophysics (UTIG) in April of 2014. On advice from Stephen Cobeldick, some changes were made in August 2014. Functionality has not changed with these updates, but the functions are now faster and more robust. Thanks Stephen.

Published with MATLAB® R2012b
