# Supplementary figures and images for: A Stochastic Dynamic Operator Framework That Improves the Precision of Analysis and Prediction Relative to the Classical Spike-Triggered Average Method, Extending the Toolkit
Source: eNeuro. 2024 Nov 5;11(11):ENEURO.0512-23.2024. doi: 10.1523/ENEURO.0512-23.2024 (PMC11552545; doi:10.1523/ENEURO.0512-23.2024)

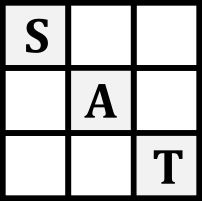

Supplement: SDO Analysis Toolkit — Download SDO Analysis Toolkit, ZIP file. [file eneuro-11-ENEURO.0512-23.2024-s001.zip › sdoAnalysis_20240821/SAT_Logo.png]

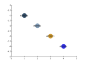

Supplement: SDO Analysis Toolkit — Download SDO Analysis Toolkit, ZIP file. [file eneuro-11-ENEURO.0512-23.2024-s001.zip › sdoAnalysis_20240821/sat-master/external/hex2rgb/rgb2hex_and_hex2rgb_documentation/html/rgb2hex_hex2rgb_demo.png]

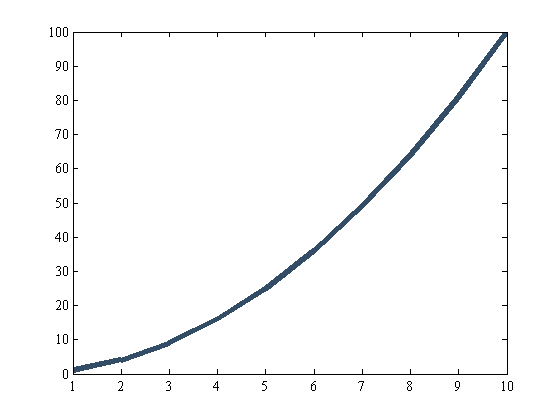

Supplement: SDO Analysis Toolkit — Download SDO Analysis Toolkit, ZIP file. [file eneuro-11-ENEURO.0512-23.2024-s001.zip › sdoAnalysis_20240821/sat-master/external/hex2rgb/rgb2hex_and_hex2rgb_documentation/html/rgb2hex_hex2rgb_demo_01.png]

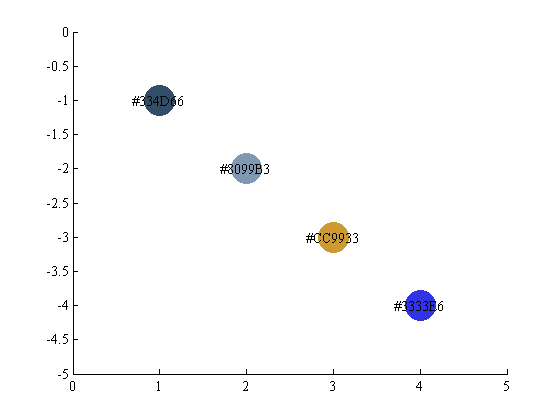

Supplement: SDO Analysis Toolkit — Download SDO Analysis Toolkit, ZIP file. [file eneuro-11-ENEURO.0512-23.2024-s001.zip › sdoAnalysis_20240821/sat-master/external/hex2rgb/rgb2hex_and_hex2rgb_documentation/html/rgb2hex_hex2rgb_demo_02.png]
